# Chiral coronene diimide assemblies with strong chiroptical properties in solution and thin-films

**DOI:** 10.1039/d6sc06148f

**Published:** 2026-10-13

**Authors:** Denis Hartmann, Artemijs Krimovs, Oussama Errida, Tiberiu-M. Gianga, Giuliano Siligardi, Robert Pal, Timothy A. Barendt

**Affiliations:** a University of Birmingham, School of Chemistry Edgbaston Campus Birmingham B15 2TT UK t.a.barendt@bham.ac.uk; b University of Sheffield, School of Mathematical and Physical Sciences Dainton Building, Brook Hill S3 7HF Sheffield UK d.hartmann@sheffield.ac.uk; c University of Durham, Department of Chemistry South Road Durham DH1 3LE UK; d Diamond Light Source, Harwell Science & Innovation Campus Didcot OX11 0DE UK

## Abstract

The self-assembly of chiral organic dyes provides an effective route to strongly chiral optical materials. Herein, we report a novel coronene diimide derivative with point-chiral imide groups derived from l/d-valinol. This chiral chromophore shows solvent-dependent aggregation and readily self-assembles *via* π-stacking into strongly chiroptically active materials, effectively translating point chirality into supramolecular chirality in the assembly. These properties are retained in the solid state, with thin films of these molecules showing strong circular dichroism (CD) and circularly polarised luminescence (CPL), realising easily accessible point chirality as a structural element for chiroptical materials.

## Abstract 2

A coronene diimide with α-chiral imide groups was synthesised. This molecule self-assembles into strongly CD and CPL active assemblies, which retain activity in thin films.

## Graphical abstract

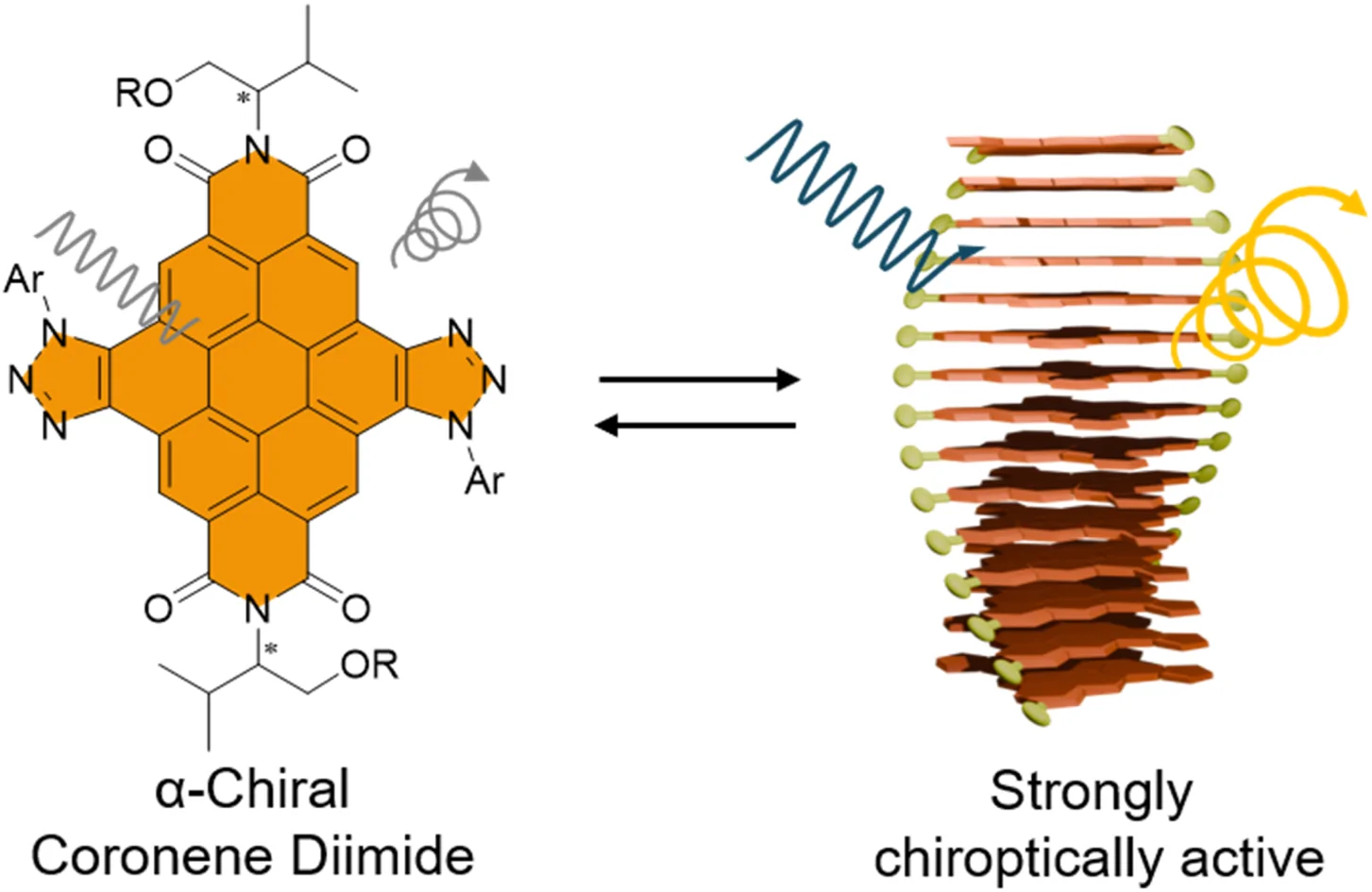

## Introduction

Self-assembly is the fundamental supramolecular process of small molecular building blocks assembling into larger structures *via* non-covalent interactions.^1^ Molecules that can self-assemble are under heavy investigation for their unique properties, such as self-healing,^2^ encapsulation,^3^ formation of defined nanostructures,^4,5^ and electrical^6^ and optical^7^ properties, and therefore find use in drug delivery^8^ and next-generation optoelectronic materials.^9–11^ Among such systems, self-assembly into chiral nanostructures is especially desirable, as it can afford materials with strong chiroptical properties,^12–14^ quantified by their dissymmetry factor (g), due to amplification of chirality,^15^ and therefore can find use in chiroptical applications such as sensing,^16^ spintronics^17,18^ and security inks.^19^

Perylene diimides (PDIs) are organic dyes with outstanding photophysical, electrochemical and chiroptical properties and a wealth of supramolecular chemistry.^20–23^ Lateral core-extension of PDIs yields coronene diimides (CDIs), affording larger π-surfaces, hypsochromic absorption and variation in solubility and aggregation properties compared to their PDI counterparts.^24,25^ In contrast to PDIs, however, CDIs are heavily underexplored in terms of their supramolecular chemistry^26–32^ and (chir)optical properties.^25,27,32–37^

Due to their planarity, CDIs do not have the intrinsic chirality of twisted, bay-substituted PDIs,^21^ and therefore instalment of chirality has to occur on its periphery. The use of point-chiral side chains to afford chiroptical materials has been realised for many achiral organic molecules,^13,36,38–40^ including PDIs.^16,41–56^ In this context, α-chiral, amino acid-derived imide groups are robust, economic and widely available as both enantiomers, making them an excellent source of point chirality. However, we have found that certain planar PDI derivatives functionalised with such imide groups do not undergo chiral self-assembly in solution.^56,57^ Therefore, we targeted novel CDIs bearing α-chiral imide substituents. Owing to their lateral π-extension, these molecules may exhibit a stronger tendency to self-assemble than PDIs, thereby enhancing their chiroptical properties in both solution and the solid state.

Here, we have synthesised the first CDI with α-chiral imide groups derived from d- and l-valinol through π-extension of PDI (Fig. 1). We also functionalised the CDI at the core, as these groups may provide a further handle for self-assembly and is in contrast to a previously explored chiral CDI,^36^ which was only functionalised at the imide positions. In this regard, we have chosen to install biphenyl arms, which may assist self-assembly through additional π–π stacking interactions.^58^ In contrast to analogous PDIs,^56,57,59^ this CDI showed rich self-assembly behaviour with strong CD (|*ε*_max_| = 2500 M^−1^ cm^−1^). We investigated the structure–property relationships responsible for the self-assembly, including the importance of planarity and judicious choice of solubilising side chains at the imide positions. The CDI showed promising application in thin films, with large CD and CPL dissymmetry factors (*g* = 10^−2^) being retained in the solid state.

**Fig. 1:**
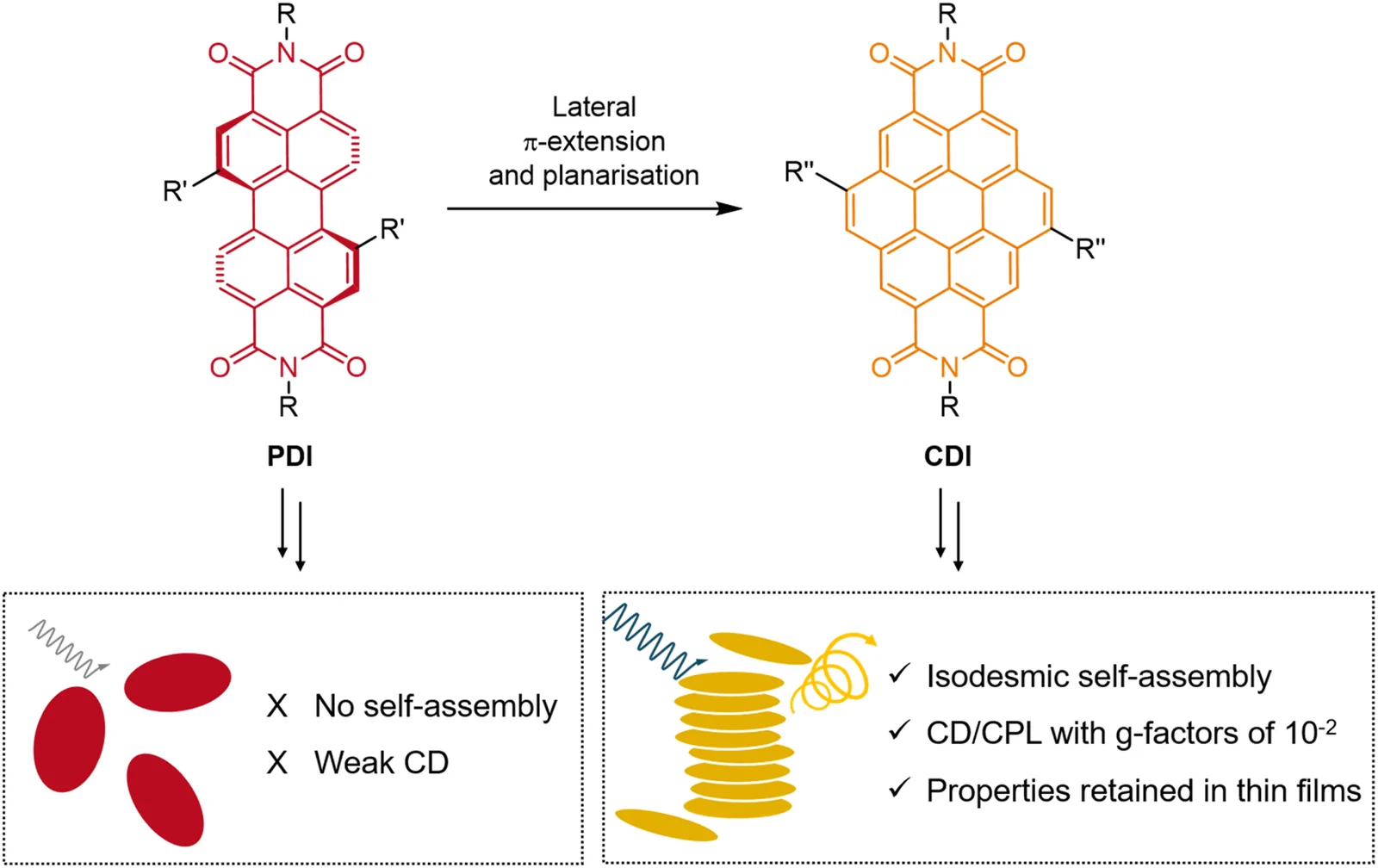
Lateral π-extension of twisted PDIs to afford planar CDIs and the consequences on supramolecular self-assembly and chiroptical properties.

## Results and discussion

### Synthesis and characterisation

We began our synthesis (Fig. 2a) from the TBS-protected l- (**a**-series) or d- (**b**-series) valinol-derived 1,7-dibromo PDI previously reported by us.^60^ Sonogashira cross-coupling with TMS acetylene afforded bis-alkynes 1ab in excellent yield. Deprotection of the TMS group with K_2_CO_3_, followed by Cu-catalysed alkyne–azide cycloaddition with biphenyl azide yielded bistriazole-PDIs 2ab in further excellent yields. We were able to obtain single crystals of 2b (Fig. 2b), which confirmed the successful installation of the biphenyl arms onto the perylene core. This PDI was then subjected to a Mallory photocyclisation using green light,^59,61^ producing the desired CDI scaffold 3ab. We were able to grow single crystals of both 3a and 3b by slow evaporation of a solution from CHCl_3_ and CH_2_Cl_2_ respectively, allowing us to confirm the structure and point chirality of this CDI and the planarity of the coronene core (Fig. 2c).

**Fig. 2:**
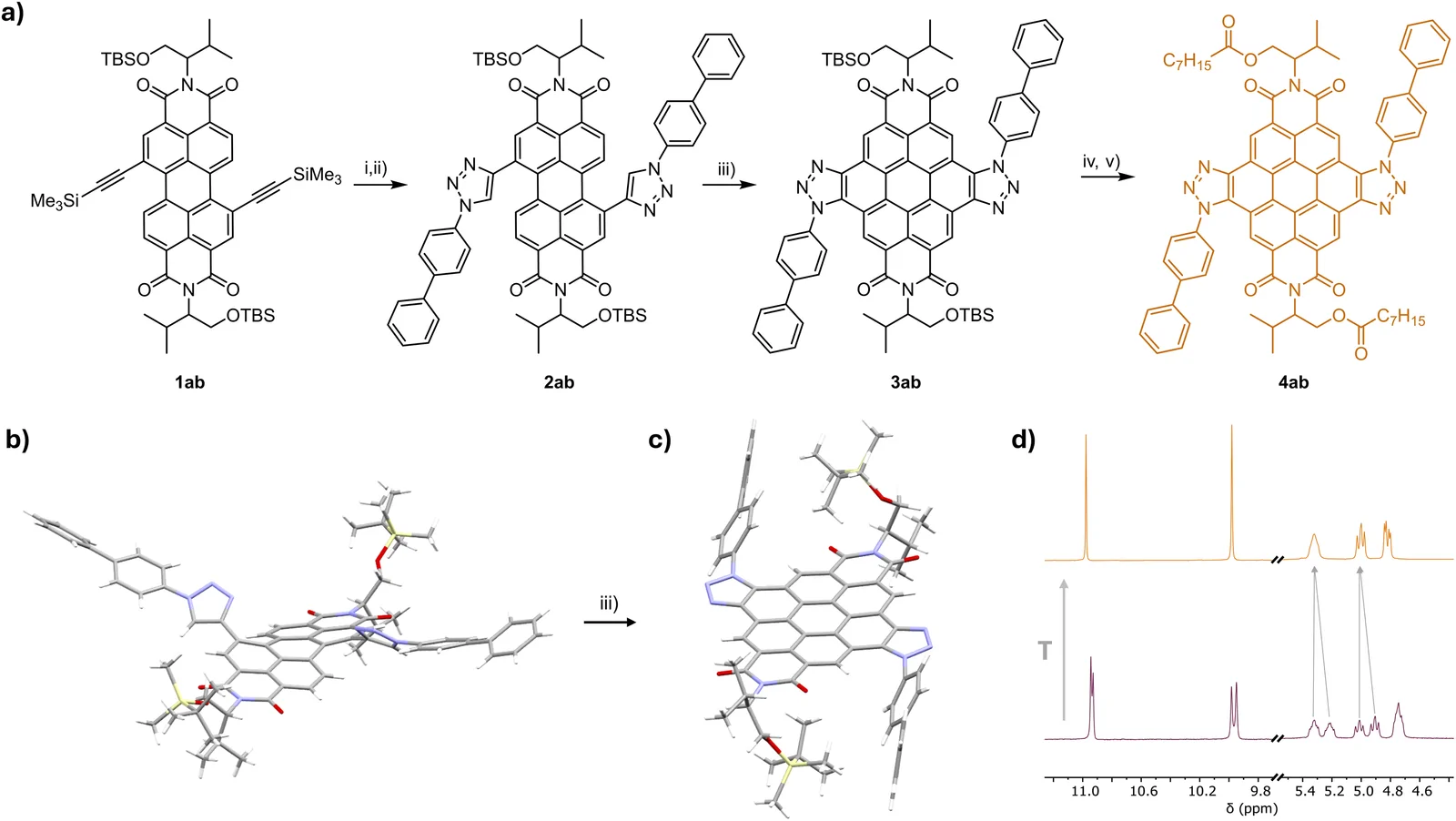
Synthesis and characterisation of the CDIs investigated in this study. (a) Synthetic procedures of the l- (**a**-series) and d-valinol (**b**-series)-derived CDIs 4ab. (i) K_2_CO_3_, MeOH, CH_2_Cl_2_. (ii) biphenyl azide, Cu(MeCN)_4_PF_6_, TBTA, CH_2_Cl_2_. (iii) 525 nm LED, CHCl_3_. (iv) HCl, Et_2_O, CH_2_Cl_2_. (v) Octanoyl chloride, DMAP, pyridine, CH_2_Cl_2_. (b) Single crystal X-ray diffraction structure of 2b. Solvent molecules were omitted for clarity. (c) Single crystal X-ray diffraction structure of 3b. Solvent molecules were omitted for clarity. (d) Partial ^1^H NMR spectra of CDI 4a in 1,1,2,2-tetrachloroethane-*d*_2_ at 25 °C and 100 °C.

The CDI 3a showed no appreciable self-assembly (*vide infra*), which we attributed to the bulky TBS groups. We therefore removed these protecting groups with HCl in Et_2_O/CH_2_Cl_2_ to yield the CDI diol, which was found to be very insoluble. To allow for chiral self-assembly in solution, we therefore installed linear alkyl chains by acylation of the valinol groups using octanoyl chloride, yielding soluble CDI derivatives 4ab for further investigation (Fig. 2a).

We noticed a doubling of peaks in the ^1^H NMR spectra of CDIs 3ab and 4ab during their synthesis, which we attribute to restricted rotation along the imide N–C bond.^62^ We therefore performed variable temperature ^1^H NMR spectroscopy in 1,1,2,2-tetrachloroethane-*d*_2_ (Fig. 2d and SI Fig. 9-1) on the most soluble CDI derivative 3a, which is consistently monomeric as seen by UV-vis spectroscopy (*vide infra*). Upon heating, the peaks coalesced, giving rise to a single set of signals (Fig. 2c). This allowed us to estimate the barrier of interconversion as Δ*G*^‡^ = 69 kJ mol^−1^ (SI Table 9-1), which is in line with theory (71 kJ mol^−1^).^62^ This barrier is high enough to give rise to restricted rotation, but low enough to allow for interconversion at room temperature.^63^ The resulting *syn*- and *anti*-rotamers of the valinol-groups are further evidenced by the crystals of 2b and 3b (Fig. 2b and c), with the former showing a *syn*-relationship of the imide substituents and the latter an *anti*-relationship in the solid state. From ^1^H NMR spectroscopy, the similar relative integrations of the rotamers at equilibrium (∼1.2 : 1) implies that they are close in energy (Δ*G* ≈ 0.5 kJ mol^−1^).

### Self-assembly in solution

We then went on to investigate the solvent-dependent self-assembly of the octanoylated valinol-derived CDIs 4ab. We measured the UV-vis spectra of 4a in several solvents (Fig. 3b, SI Fig. 2-1 and 2-2). In low to intermediate polarity solvents such as PhMe or CHCl_3_ the spectrum looked monomeric and resembled the spectrum of 3a and for a previously synthesised CDI.^59^ We further confirmed the monomeric nature of the CDI absorption spectrum in CHCl_3_ by computational methods. Prediction of the absorption spectrum of a CDI monomer by TD-DFT (TD-ωB97x,^64^ def2-SVP,^65^ SI Fig. 7-1 and 7-2) with implicit solvation in CHCl_3_ showed a close match with the observed spectrum.

**Fig. 3:**
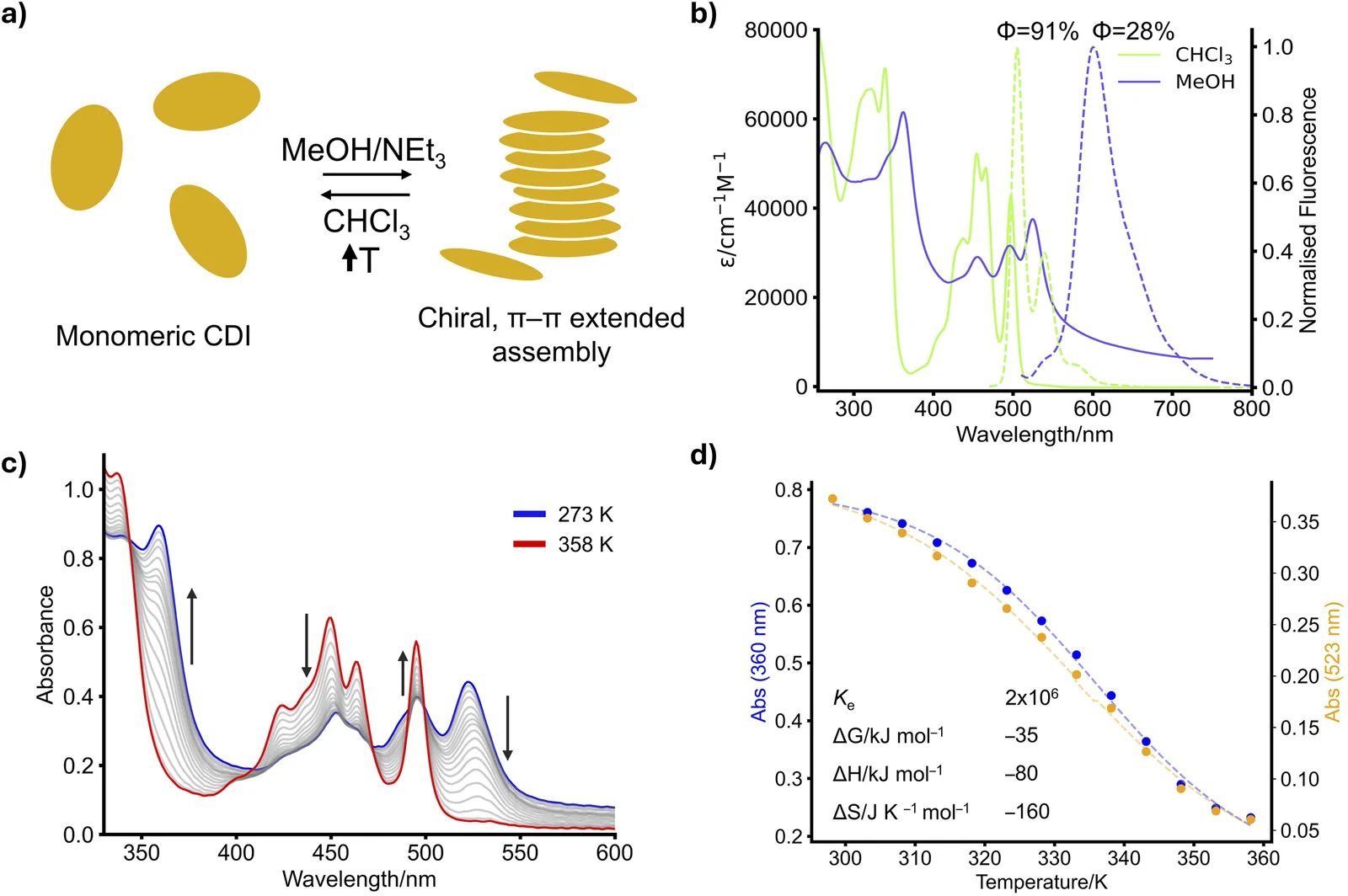
Self-assembly of CDI 4ab. (a) MeOH or NEt_3_ induced self-assembly of CDI and disaggregation with CHCl_3_ or heat. (b) UV-vis absorption spectra, normalised fluorescence spectra and quantum yields (*Φ*) of 4a in CHCl_3_ (*λ*_ex_ = 450 nm) and MeOH (*λ*_ex_ = 490 nm) at 10 µM concentration. (c) Changes in absorption spectrum of 4a (20 µM) in 9 : 1 NEt_3_ : CHCl_3_ upon heating. (d) The change of the 365 nm and 528 nm absorption peaks of 4a (20 µM) in (9 : 1 NEt_3_ : CHCl_3_) in as a function of temperature, fitted to an isodesmic model.

In low polarity/apolar solvents (NEt_3_/cyclohexane) or those of high polarity (MeOH (Fig. 3b and SI Fig. 2-2–2-5), however, the molecule showed a distinct absorption spectrum with a new, red-shifted band and a change in the vibrational progression, indicating self-assembly in the respective solvents, with similar spectral features to a related coronene aggregate).^30^

We further investigated this self-assembly behaviour by fluorescence spectroscopy (Fig. 3b, SI Fig. 2-6 and 2-7). Whereas in CHCl_3_ CDI 4ab showed a monomeric emission spectrum with distinct vibrational progression, a small (8 nm) Stokes shift and high quantum yield (91%), the spectrum in MeOH was broad and featureless, with a large Stokes shift (75 nm) and a lower quantum yield (28%). The latter is characteristic of H-type electronic coupling between chromophores upon their self-assembly, and consistent with a co-facial, π-stacked arrangement.^66,67^ This also matches previously reported spectral changes of a coronene derivative upon aggregation *via* co-facial π-stacking.^30^

The thermodynamics of CDI self-assembly were studied in more detail by UV-vis absorption spectroscopy. Initially, a solvent-into-solvent titration of 4a in CHCl_3_ into MeOH (SI Fig. 3-1 and 3-2) was performed and revealed a decrease in intensity of the absorption band at ∼530 nm, and the reemergence of monomer transitions; thereby showing the reversibility of aggregation; with full disassembly observed at 40% CHCl_3_. An analogous solvent-into-solvent titration of CHCl_3_ into a solution of 4a in NEt_3_ (SI Fig. 3-3 and 3-4) also showed disaggregation with increasing % of CHCl_3_, with full disassembly at 30% CHCl_3_. The amount of CHCl_3_ for full disaggregation is smaller than for MeOH, and indeed a comparison of the ratio of aggregate (∼530 nm) to monomer (496 nm) absorption peaks in both solvents shows the molecule to be more aggregated in MeOH (*A*_530_/*A*_496_ = 1.10) compared to NEt_3_ (*A*_526_/*A*_496_ = 0.94). Similarly, a comparison of hydrodynamic radius by dynamic light scattering (DLS) revealed the aggregate in NEt_3_ to be smaller (SI Fig. 4-1 and 4-2).

We also wanted to establish the mode of self-assembly and quantify it. We therefore prepared a solution of 4a in 3 : 1 MeOH : CHCl_3_ (10 µM), where the molecule is ∼50% aggregated at room temperature, and recorded UV-vis absorption spectra at different temperatures from 0 to 65 °C (SI Fig. 3-5).^32^ Upon heating, the spectra reveal disaggregation with increasing temperature, with the disappearance of the characteristic absorption band at 528 nm. Fitting this wavelength (as well as another distinct peak at 365 nm) to a temperature-dependent isodesmic model^68^ (SI Fig. 3-7, 3-8 and Table 3-1) gave an excellent fit and allowed us to estimate an equilibrium constant, *K* = 3 × 10^5^ M^−1^ at 298 K. This value is in line with previously reported CDIs.^36,59^ Isodesmic assembly is expected, given the free interconversion of the *syn*-/*anti*-rotamers (*vide supra*). Dimer and cooperative assembly models^69^ were also tested but did not fit the recorded data. DLS measurements on the aggregates of 4ab also revealed a decrease in hydrodynamic radius upon heating, as anticipated, as the aggregate disassembles (SI Fig. 4-3).

To further assess the self-assembly, we repeated this temperature-dependent disaggregation in 9 : 1 NEt_3_ : CHCl_3_ (10 µM), where it is partially disaggregated at room temperature (SI Fig. 3-11–3-13). The data again showed full disassembly upon heating, with fitting to the same isodesmic model (SI Fig. 3-13 and Table 3-2) yielding an equilibrium constant *K* = 2 × 10^6^ M^−1^ at 298 K, which may reflect the lower %vol CHCl_3_ used in comparison with the previous experiment in 3 : 1 MeOH : CHCl_3_. Self-assembly was further confirmed at higher concentration (Fig. 3c and d and SI Fig. 3-16, 20 µM), with a matching equilibrium constant after fitting to the same isodesmic model (Fig. 3d and SI Fig. 3-18, 3-19 and Table 3-3). The self-assembly was also investigated by concentration-dependent UV-vis measurements (SI Fig. 3-24 and 3-25), which support the isodesmic aggregation model identified from the variable-temperature studies. Although the equilibrium constants extracted from the two methods differ, such discrepancies are reported for self-assembling systems because concentration- and temperature-dependent experiments probe the aggregation equilibrium in different ways and are influenced by different spectroscopic assumptions.^70^

To probe the reversibility of the aggregate formation the heated solutions were also slowly cooled and their absorbance and CD spectra recorded (SI Fig. 3-6, 3-9, 3-10, 3-12, 3-16–3-23). At 20 µM in 9 : 1 NEt_3_ : CHCl_3_, a different aggregate was observed upon cooling, which was also transiently observed at 10 µM and in methanolic solution. DLS revealed it to be larger than the starting aggregate (1162 *vs.* 597 nm, SI Fig. 4-4), and it did not revert back to the starting aggregate over the time period measured (SI Fig. 3-26 and 3-27). This new aggregate showed a main absorbance peak redshifted from the starting aggregate (537 *vs.* 523 nm), with an inverted CD spectrum in the region of 320–420 nm and weaker *ε*_max_, showing a diastereomeric relationship between the two aggregates. Such chirality inversion has previously been reported in diastereomeric aggregates of a chiral CDI derivative.^37^ We were able to fit the absorbance data obtained upon cooling to an isodesmic model (SI Fig. 3-20 and 3-21) and found *K* = 1 × 10^6^ M^−1^ at 298 K (SI Table 3-4), lower than for the starting aggregate observed in NEt_3_, which explains why the prior aggregate is formed preferentially in MeOH or NEt_3_, as well as in thin films (*vide infra*).

The temperature-dependent aggregation experiments revealed that the self-assembly is driven by enthalpy, with a sensible decrease in entropy as the molecules assemble. This indicates that dispersion interactions likely provide a stronger driving force for π–π self-assembly^71^ than CDI desolvation, the π-surfaces of which are expected to be poorly solvated in polar MeOH or weakly polar NEt_3_.

We note that in both solvents light scattering was observed upon self-assembly, likely arising from the formation of higher-order aggregates. To assess the influence of scattering on the quantitative analysis, we performed a scattering correction to the absorption spectra and refitted the data (SI Fig. 3-28–3-39). The resulting equilibrium constants differ only modestly from the original values and remain within the same order of magnitude across all datasets (SI Table 3-9), leading to identical mechanistic conclusions. These results indicate that light scattering has only a limited effect on the extracted *K* values under the conditions employed. Nevertheless, because the onset of scattering is consistent with the formation of poorly soluble higher-order aggregates, the reported aggregation parameters should be regarded as estimates of the thermodynamics.

### Chiroptical properties in solution

We performed CD measurements of CDIs 4ab in both CHCl_3_ and MeOH (Fig. 4b). While both enantiomers are CD silent as monomers in CHCl_3_, arising from the fact there is minimal coupling between the chiral centre and the CDI core,^72–74^ we observed intense CD in MeOH (|*ε*_max_| = 2500 M^−1^ cm^−1^), with the three lowest energy transitions giving |*g*_abs_| = 0.07 (*λ* = 371 nm), 0.02 (464 nm) and 0.01 (528 nm) (SI Fig. 2-8). These values are substantially higher than those typically observed for point-chiral organic chromophores (10^−5^–10^−3^);^74^ moreover, the |*g*_abs_| value at 371 nm is excellent amongst chiral supramolecular polymers,^75^ including other CDI-based assemblies.^18,37,76^ We further measured the linear dichroism (LD) of the molecule in solution to rule out any distortions of the CD signal, and found the LD to be negligible (SI Fig. 2-9).

**Fig. 4:**
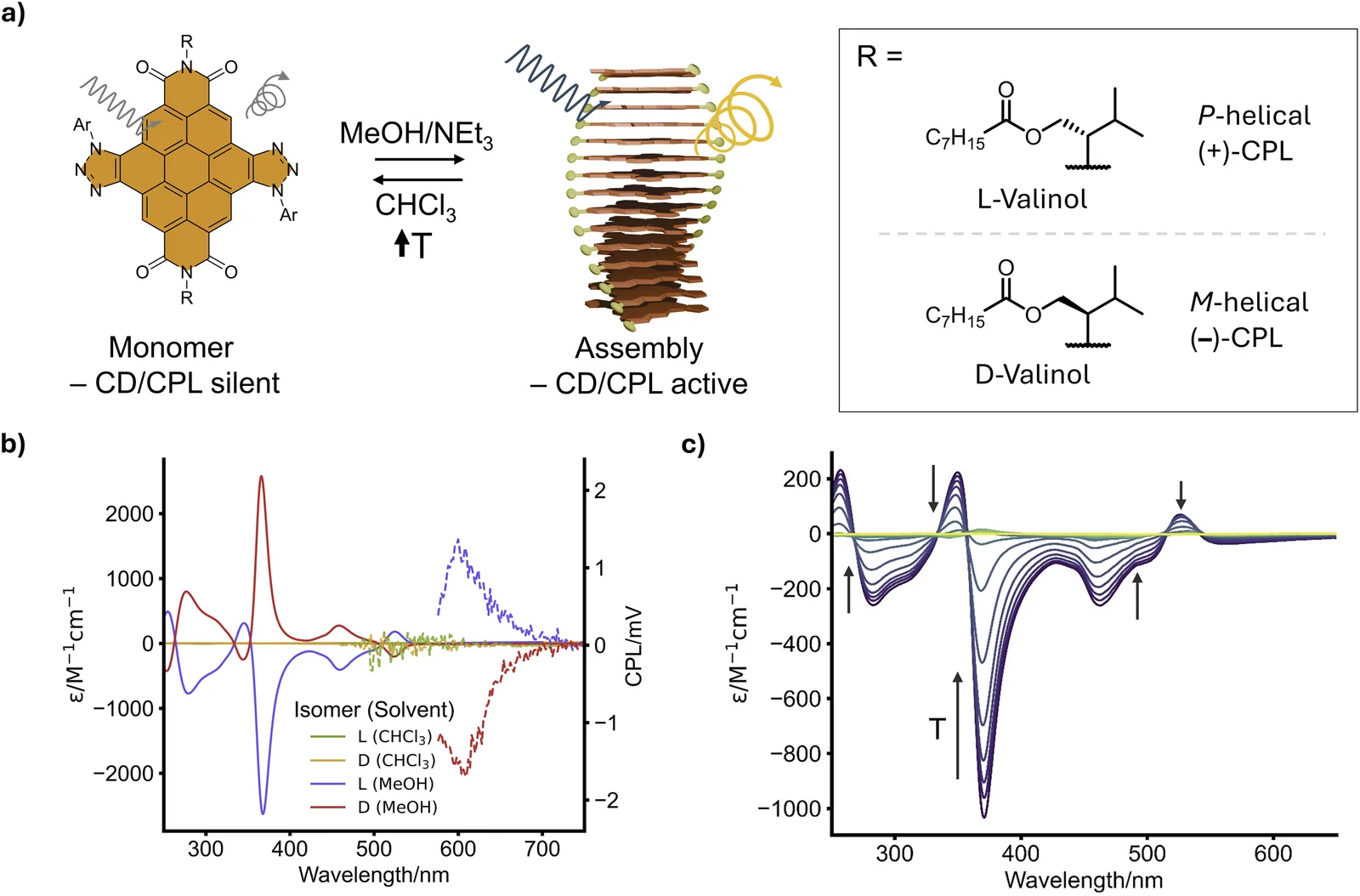
Chiroptical properties of CDI 4ab in solution. (a) Assembly of aggregate in MeOH or NEt_3_, and disassembly of aggregate with increasing temperature or CHCl_3_. (b) CD and CPL spectra (*λ*_ex_ = 450 nm) of 4a (l-isomer) and 4b (d-isomer) in CHCl_3_ and MeOH (10 µM). (c) Loss of CD of 4a in 25% CHCl_3_ in MeOH upon heating.

Due to the negligible CD of CDI 4ab in its monomeric state, the high dissymmetry factors in MeOH arise from (H-type) electronic coupling between chromophores in the chiral aggregate. This is evidenced by the bisignate nature of the lowest energy CD band at ∼500 nm. The observed Cotton effect also indicates a helical assembly, with a rotational displacement being sensible to accommodate the CDI's biphenyl arms, with its handedness directed by the l/d-valinol's *iPr* groups. The sign of this Cotton effect also allows us to assign the helicity of the observed H-aggregates using the exciton chirality rule,^77^ with the l-isomer giving a right-handed (*P*)-helix, and the corresponding d-isomer a left-handed (*M*)-helical arrangement. This is further consistent with the supramolecular chirality of a π-stacked bis-PDI macrocycle with l/d-valinol-based imide groups.^56^

We then went on to measure the CPL of the CDIs 4ab in monomeric and assembled states (Fig. 4b). The self-assembled structure in MeOH was found to be CPL active, with mirror image spectra for the enantiomers and a |*g*_lum_| of 0.01, matching in both sign and magnitude with the lowest energy transition observed by CD. This |*g*_lum_| is amongst the highest reported for organic supramolecular polymers in solution, including another chiral coronene assembly,^36^ and translates into a significant CPL brightness^78^ (*B*_CPL_) of 40 M^−1^ cm^−1^ at 600 nm, which is competitive with chiral J-aggregates.^79^ In contrast, in CHCl_3_, we also did not observe any CPL, showing that the point-chiral CDI monomer is also CPL silent. Therefore, self-assembly, and the resulting supramolecular chirality, are crucial for chiroptical activity. Indeed, upon heating, we also observed a loss of CD in line with the absorbance spectrum change towards the monomer, with a full loss of CD upon complete disassembly in both 3 : 1 MeOH : CHCl_3_ (Fig. 4d) and 9 : 1 NEt_3_ : CHCl_3_ (SI Fig. 3-14 and 3-22).

While analogous planar PDIs are monomeric in solution,^56,57^ demonstrating the importance of lateral π-extension in CDIs, we sought further structure–property relationships by examining the aggregation of the twisted PDI 2a and the TBS-protected CDI 3a (SI Fig. 2-10–2-13). For both, there was only a minimal UV-vis spectral change in a range of solvents, including those considered to be ‘poor’ solvents for rylene diimides,^71^ such as MeOH and cyclohexane. As a result of the limited self-assembly, only very small CD was observed (|*ε*| < 10 cm^−1^ M^−1^, SI Fig. 2-10 and 2-13). Therefore, the result with twisted PDI 2a suggests that chromophore planarisation aids self-assembly. The single crystal structure of 2b (Fig. 2b and SI Fig. 6-1 and 6-2) also indicates that the position of the biphenyl side arms may make the PDI less amenable to self-assembly compared to the CDI because they hinder one face of the PDI and reduce the space between the core and imide substituents. Planar CDI 3a highlights the need for less bulky and flexible side chains (*i.e.*, octanoyl *vs.* TBS). Indeed, the single crystal structure of 3a shows the TBS-groups above and below the plane of the CDI (Fig. 2c and SI Fig. 6-4) hindering close approach of individual CDI molecules and resulting in a relatively large π–π distance of 8.9 Å.^80^

### Chiroptical properties in thin films

Lastly, we were interested in whether the large CD and CPL of the chiral CDIs in the self-assembled state could be translated into thin films. We therefore prepared films of both l- and d-valinol-derived CDI 4ab, as well as a racemic (1 : 1) mixture of both, by dropcasting from a solution in CHCl_3_. We found only weak CD at this point, but upon annealing of the films at 160 °C (SI Fig. 5-1 and 5-2), we observed a colour change from a light to a dark orange and strong CD for the films which closely resembled solution data in MeOH (Fig. 5b). We believe that the annealing allowed the molecules to arrange into similar π-extended assemblies to those observed in MeOH solution (Fig. 5a), while also removing residual CHCl_3_, which would disrupt such assembly. Polarised light microscopy (SI Fig. 5-7) confirmed the increased level of structural organisation, as small crystallites became visible under cross-polarisation. Powder X-ray diffraction experiments of samples of the film pre- and post-annealing (SI Fig. 6-7 and 6-8) further revealed an increase in crystallinity, with more defined peaks and a larger number of higher angle reflections observed in the latter. The film powder patterns also showed a close match with the pattern recorded for dried residue from MeOH solution (SI Fig. 6-9), indicating that the morphologies of the thin film and solution states are similar. We further measured films of 4a by atomic force microscopy (SI Fig. 8-1 and 8-2), which showed assemblies of similar size to those in solution. Attempts to form films from MeOH or NEt_3_ directly were unsuccessful (SI Fig. 5-8 and 5-9).

**Fig. 5:**
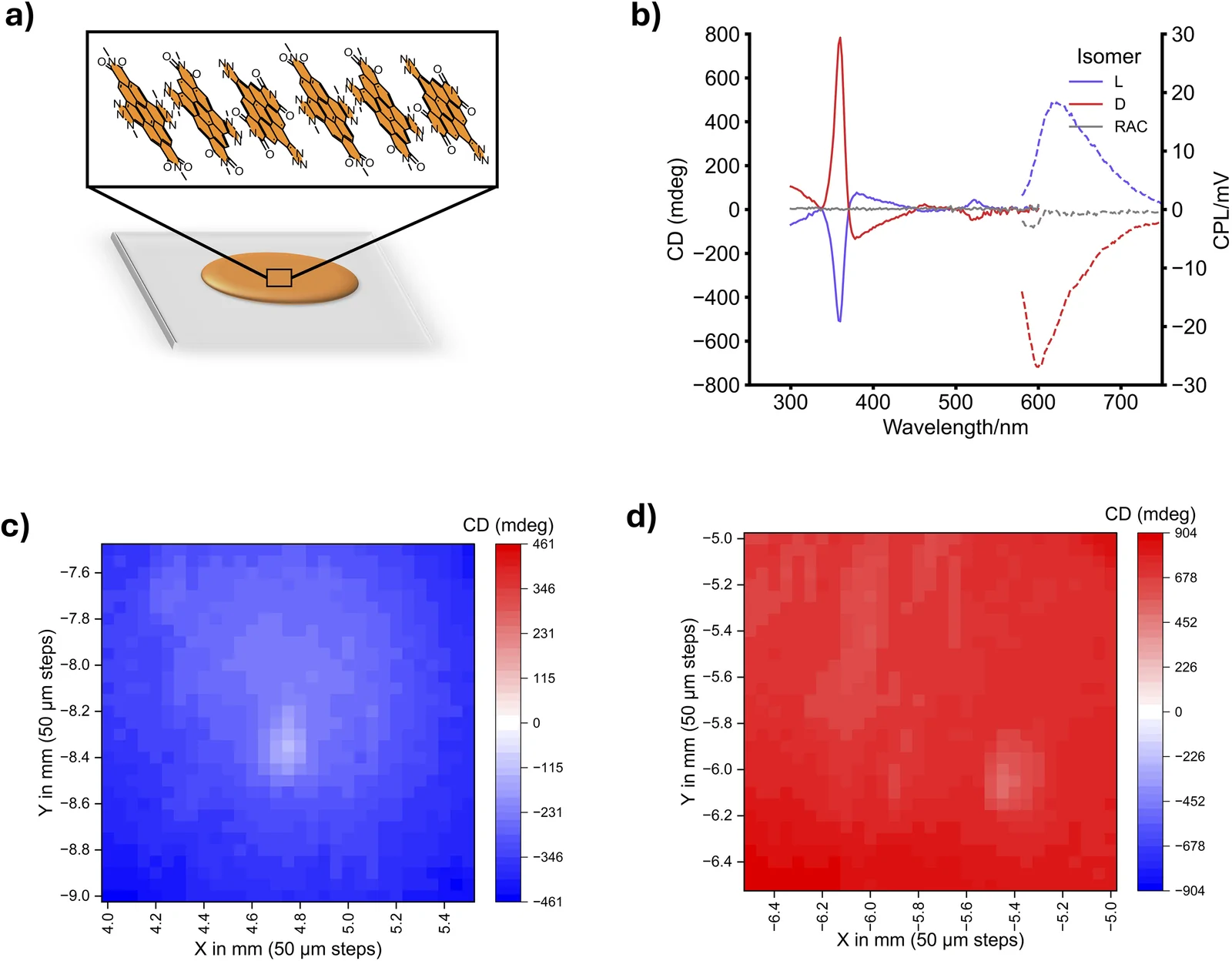
Thin film properties of CDIs 4ab. (a) The π-extended assemblies observed in annealed thin films of 4ab. (b) CD and CPL spectra (*λ*_ex_ = 450 nm) of dropcast thin films of 4ab. (c) MMP map (358 nm) of a film of CDI 4a. (d) MMP map (358 nm) of a film of CDI 4b.

We prepared four films of each enantiomer and measured them on a conventional benchtop CD spectrometer (SI Fig. 5-3–5-6) and found them to be reproducible. Profilometry further confirmed uniform thickness of the films (SI Fig. 5-10–5-12). The films were then analysed by Mueller Matrix Polarimetry (MMP, SI Fig. 5-13) at Diamond Light Source (Beamline B23) to get true CD spectra (Fig. 5b) as well as information on LD and birefringence properties (SI Fig. 5-14–5-19).^81^ Since the depolarisation index is above 0.5 and hence the CD is not affected by linear anisotropies (LD and LD'), the analytic inversion method was used to decompose the signals and obtain a true CD signal (Fig. 5b). We found that the apparent CD spectra recorded on a conventional benchtop spectrometer (SI Fig. 5-20) are a very close match to the true CD spectra of 4ab recorded by MMP (Fig. 5b and SI Fig. 5-21).

We calculated |*g*_abs_| for these films using the obtained MMP data, and it was found to be on the same order of magnitude as solution spectra (10^−2^). The strong CD of the films was found to be uniform for both 4a and 4b, showing little variation in sign and magnitude from film maps generated using MMP over a 1.5 mm^2^ area at 50 µm resolution (Fig. 5c and d and SI Fig. 5-22–5-27). Importantly, a racemic film showed no CD, and was also homogenous, indicating no domains of enantiomers, or artefacts arising from LD or birefringence. The lack of chiroptical activity displayed by the racemic films supports the intrinsic supramolecular chirality as the origin of the CD.

Finally, we found the films to be fluorescent and therefore measured their CPL spectra (Fig. 5b). These again matched solution data in MeOH, with a calculated |*g*_lum_| of 10^−2^, consistent in both sign and magnitude to the thin film CD. The quantum yield of the films is 28% (SI Fig. 5-28), consistent with the value obtained in solution.

## Conclusions

In summary, we have prepared novel CDI derivatives with α-chiral imide groups derived from d- and l-valinol. Compared to analogous chiral PDIs, these CDI derivatives showed strong self-assembly behaviour due to their lateral π-extension, controlled by solvent effects. In good solvents such as CHCl_3_, the CDIs were monomeric and thus CD and CPL silent. In contrast, in MeOH and NEt_3_, self-assembly was observed, with the point chirality of the imide group translating into supramolecular chirality, to afford strong CD and CPL with high dissymmetry factors on the order of 10^−2^ and large *B*_CPL_ (40 M^−1^ cm^−1^ at 600 nm).

Importantly, the choice of solubilising group on the imide (bulky TBS *vs.* flexible octanoyl) is crucial in enabling self-assembly of the CDI. The aggregation was found to be temperature-dependent, allowing for its quantification by variable temperature UV-vis absorption spectroscopy. This revealed the assembly behaviour to be isodesmic with a relatively large association constant, *K* = 10^6^ M^−1^. Importantly, thin films of the CDIs retained the strong chiroptical properties observed in solution and closely matched the observed solution data.

This work shows that through careful design of π-surface (laterally extended from PDI to CDI) and solubilising group (octanoyl), readily available α-chiral imide groups can provide an effective route to strong chiroptical materials *via* π–π self-assembly. Notably, the self-assembly of these CDIs was effectively translated into thin films, making them promising materials for chiroptical applications.

## Author contributions

D. H. and T. A. B. devised the project. D. H. performed the majority of the work, with contributions from O. E., T.-M. G. and G. S. on the MMP measurements. A. K. and R. P. performed the CPL measurements. D. H. and T. A. B. wrote the manuscript.

## Conflicts of interest

There are no conflicts to declare.

## Supplementary Material

SC-OLF-D6SC06148F-s001

SC-OLF-D6SC06148F-s002

## Data Availability

CCDC 2560269, 2560270 and 2560271 contain the supplementary crystallographic data for this paper.^82*a*–*b*^ All the data that support the findings of this study are available in this article and its supplementary information (SI). The raw data are available from the corresponding authors upon request. Supplementary information: the general methods, synthesis and characterisation of the molecules, additional photophysical spectra, MMP maps, MMP spectra, all methods for processing and analysing the MMP data, computational methods and crystallography data. See DOI: https://doi.org/10.1039/d6sc06148f.
