# Supplementary material for: Chiral coronene diimide assemblies with strong chiroptical properties in solution and thin-films
Source: Chem Sci. 2026 Oct 13. Online ahead of print. doi: 10.1039/d6sc06148f (PMC13679708; doi:10.1039/d6sc06148f)
Supplement: Supplementary file 1 [file SC-OLF-D6SC06148F-s002.pdf]

Supplementary Information (SI) for Chemical Science.
This journal is © The Royal Society of Chemistry

Supplementary Information

Chiral coronene diimide assemblies with strong chiroptical properties in solution and thin-films

Denis Hartmann,[a,b,*] Artemijs Krimovs,[c] Oussama Errida,[a] Tiberiu-M. Gianga,[d] Giuliano Siligardi,[d] Robert Pal,[c] Timothy A. Barendt[a,*]

[a]University of Birmingham, School of Chemistry, Edgbaston Campus, Birmingham, B15 2TT

[b]University of Sheffield, School of Mathematical and Physical Sciences, Dainton Building, Brook Hill, S3 7HF, Sheffield

[c]University of Durham, Department of Chemistry, South Road, Durham, DH1 3LE

[d]Diamond Light Source, Harwell Science & Innovation Campus, Didcot, OX11 0DE

Contents

Contents ... 1
1) Synthesis ... 2
2) Spectroscopy ... 16
3) Self-assembly ... 24
4) Dynamic Light Scattering ... 49
5) Thin Film Measurements ... 51
6) X-Ray Diffraction ... 68
7) Computational Chemistry ... 76
8) Atomic Force Microscopy ... 78
9) NMR Spectra ... 79
10) Mass Spectra ... 105
11) References ... 117

1) Synthesis

General Synthesis

Reagents were purchased from commercial sources (Merck, Acros Organics, Fluorochem and Alfa Aesar) and used without further purification. Solvents were used as supplied (analytical/HPLC-grade from Fisher or Sigma-Aldrich). If required, solvents were dried over molecular sieves (3 Å) overnight before use, and stored under N_2 atmosphere. Petroleum ether (PE) over a boiling point range of 40–60 °C was used. Eluent mixtures are reported in volume:volume or %vol. Column chromatography was carried out using Merck Silica Gel 60 Å, 230-400 mesh, 40-63 µm particle size. TLC was carried out on Merck silica gel 60 F254 Al plates. Preparative TLC was performed using 20 × 20 cm plates with 1 cm silica thickness. NMR spectroscopy measurements were recorded using a Bruker AVIII300, AVIII400, AV Neo 400 or AV Neo 500 instrument and peaks were referenced to the residual solvent peak. Electrospray Ionisation Mass Spectrometry (ESI-MS) measurements were carried out Waters Xevo G2-XS TOF Mass Spectrometer.

OH-D-Valinol-Br_2-PDI S1

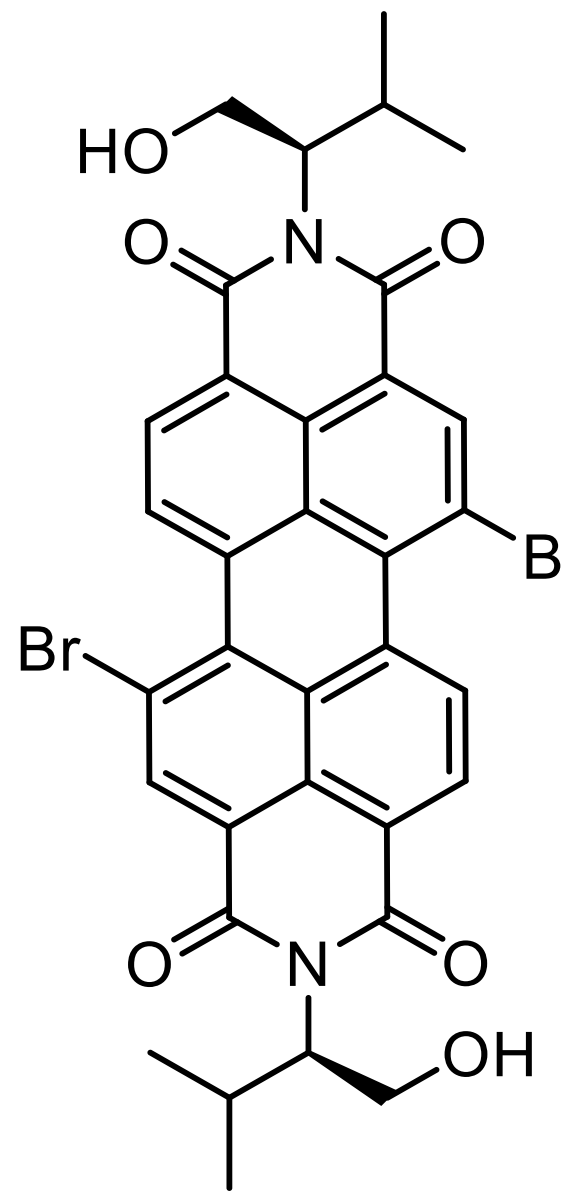

Following a published procedure:[1] To a round-bottom flask under N_2 atmosphere was added dibromo-perylene tetracarboxylic acid dianhydride (2.5 g, 4.54 mmol), D-valinol (984.55 mg, 9.54 mmol), 1,4-dioxane (20 mL) and dimethylacetamide (20 mL). The reaction mixture was stirred at 140 °C for 24 h. The mixture was then cooled, and the solvent reduced *in vacuo*. The resulting residue was purified by flash column chromatography (SiO_2, 5-15% Acetone/CH_2Cl_2) to yield a bright red solid as the title compound as a mixture of 1,7- and 1,6-brominated species (2.77 g, 3.85 mmol, 85%).

^{1}H NMR (400 MHz, $CDCl_3$) δ/ppm 9.42 (d, *J* = 8.2 Hz, 2H), 8.88 (d, *J* = 4.6 Hz, 2H), 8.64 (d, *J* = 8.2 Hz, 2H), 5.01 (ddd, *J* = 10.8, 7.9, 2.9 Hz, 2H), 4.33 (d, *J* = 8.9 Hz, 2H), 4.10 – 3.98 (m, 2H), 2.81 – 2.64 (m, 1H), 1.18 (d, *J* = 6.5 Hz, 6H), 0.85 (d, *J* = 6.8 Hz, 6H).

^{13}C NMR (101 MHz, $CDCl_3$) δ/ppm 164.32, 163.79, 138.61, 133.09, 132.97, 130.59, 129.20, 128.61, 127.21, 123.20, 122.82, 121.10, 62.65, 62.31, 26.34, 20.50, 20.04.

HRMS (ESI$^+$) found 721.0369 $[C_{34}H_{29}{}^{79}Br^{81}BrN_2O_6]^+$ requires 721.0366 (Error: 0.42 ppm).

TBSO-D-Valinol-Br_2-PDI S2

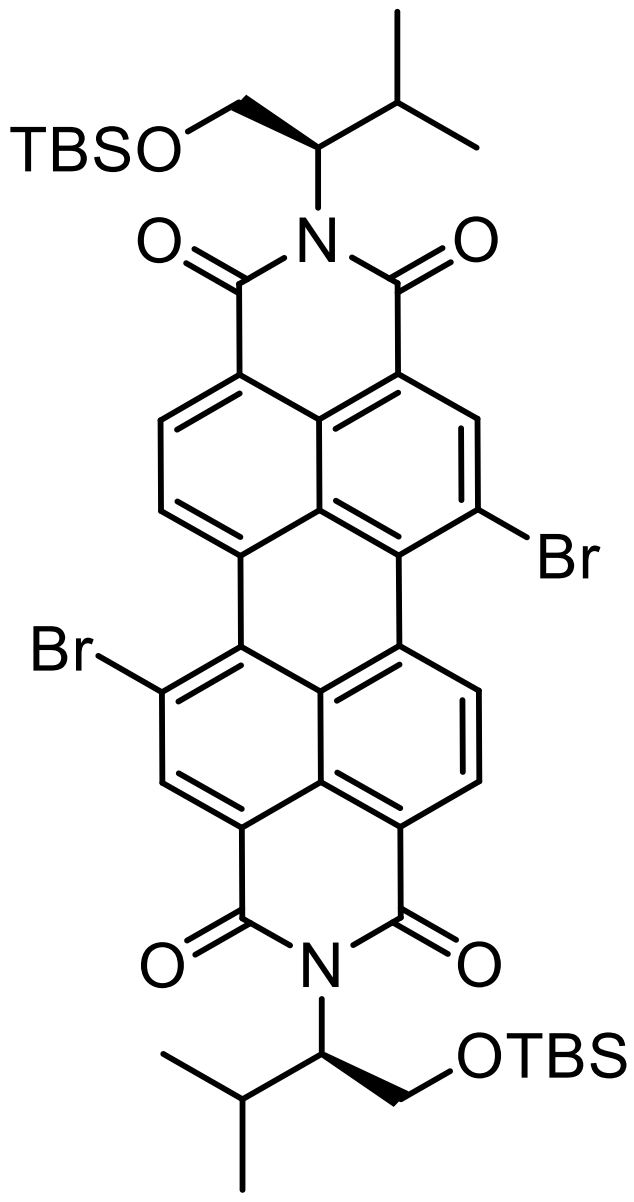

To a dry round-bottom flask under N_2 atmosphere was added **S1** (1 g, 1.39 mmol) as a mixture of 1,7- and 1,6-brominated species. To this was then added imidazole (567 mg, 8.33 mmol) and TBDMSCl (1.26 g, 8.33 mmol) and dry DMF (20 mL). The reaction was stirred at r.t. for 4 h, after which TLC showed complete conversion to the di-protected material. The solvent was removed *in vacuo* and the resulting residue purified by flash column chromatography (SiO_2, 5% EtOAc/Hexane), yielding 1.05 g of the desired compound as a mixture of isomers (80%). To separate the 1,7- from the 1,6-isomer, the resulting residue was the subjected to HPLC. The isomers were separated on a COSMOSIL Buckyprep column (28 mm x 250 mm) using a gradient of 20-38% DCM in Hexane over 20 minutes, with the first, and major species being the 1,7-isomer, which was collected (837 mg, 0.882 mmol, 64%).

^{1}H NMR (400 MHz, $CDCl_3$) δ/ppm 9.51 (d, *J* = 8.1 Hz, 2H), 8.90 (d, *J* = 22.4 Hz, 2H), 8.68 (dd, *J* = 22.7, 8.2 Hz, 2H), 5.00 (td, *J* = 10.0, 4.9 Hz, 2H), 4.33 (t, *J* = 9.9 Hz, 2H), 4.05 (dd, *J* = 10.2, 5.2 Hz, 2H), 2.65 (dp, *J* = 10.4, 6.6 Hz, 2H), 1.12 (d, *J* = 6.6 Hz, 6H), 0.88 (d, *J* = 6.7 Hz, 6H), 0.68 (s, 18H), -0.02 (s, 6H), -0.10 (s, 6H).

^{13}C NMR (101 MHz, $CDCl_3$) δ/ppm 164.41, 163.92, 163.28, 162.72, 138.51, 137.76, 133.05, 132.88, 130.55, 129.73, 129.42, 128.65, 127.29, 123.83, 123.41, 123.27, 122.85, 120.88, 62.50, 61.52, 27.53, 25.78, 20.92, 20.39, 18.08, -5.29, -5.40.

HRMS (ESI$^+$) found 947.2110 $[C_{46}H_{57}N_2Br_2O_6Si_2]^+$ requires 947.2116 (Error: -0.63 ppm).

TBSO-L-Valinol-bis-TMS-acetylene-PDI 1a

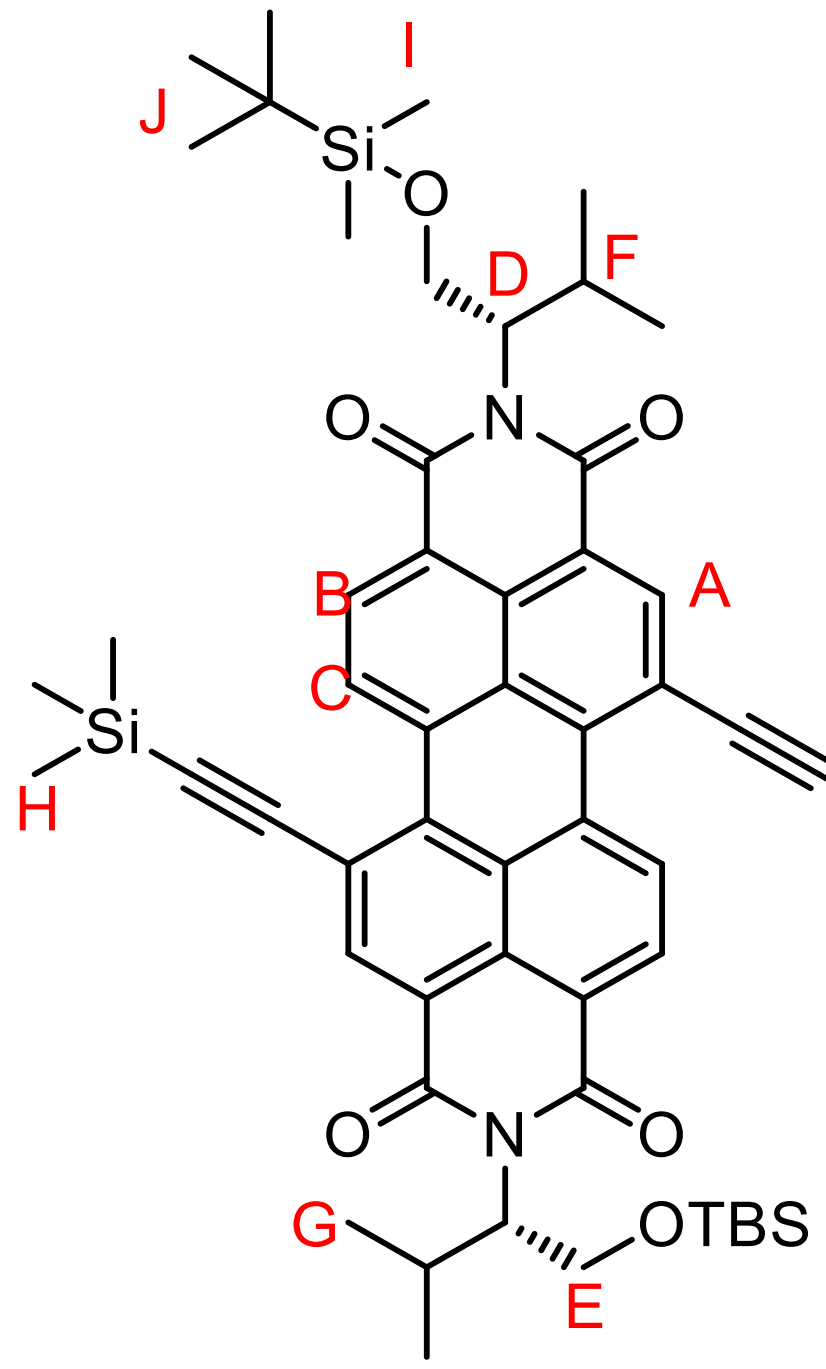

TBS Protected-LVal-PDI-Br_2 (437.5 mg, 0.461 mmol, prepared according to previously reported procedures[1]), $Pd(PPh)_2Cl_2$ (16.2 mg, 5 mol%) and CuI (8.8 mg, 10 mol%) were added to a round-bottom flask equipped with a stirrer bar and dissolved in 10 mL of a 1:1 mixture of PhMe and NEt_3. The mixture was then degassed by bubbling of N_2 for 30 minutes, before addition of TMS-acetylene (0.394 mL, 2.766 mmol, 6 eq.). The reaction was stirred at room temperature for 48 h. The solvent was then removed *in vacuo* and the resulting residue purified by flash column chromatography (SiO_2, 5% EtOAc/PE_{40-60}) to yield the title compound as a pink solid (447 mg, 0.454 mmol, 99%).

R_f (SiO_2, 5% EtOAc/PE_{40-60}) 0.33.

^{1}H-NMR (400 MHz, $CDCl_3$) δ/ppm 10.23 (d, *J* = 8.3 Hz, 2H, **C**), 8.82 (d, *J* = 21.3 Hz, 2H, **A**), 8.64 (dd, *J* = 20.1, 8.4 Hz, 2H, **B**), 5.02 (ddd, *J* = 10.5, 9.4, 5.1 Hz, 2H, **D**), 4.34 (dq, *J* = 10.4, 3.1 Hz, 2H, **E'**), 4.07 (dd, *J* = 10.2, 5.2 Hz, 2H, **E''**), 2.66 (dp, *J* = 12.5, 6.5 Hz, 2H, **F**), 1.13 (d, *J* = 6.6 Hz, 6H, **G'**), 0.90 (d, 6H, **G''**), 0.67 (s, 18H, **J**), 0.39 (s, 18H,**H**), -0.02 (s, 6H, **I'**), -0.11 (s, 6H, **I''**).

^{13}C-NMR (101 MHz, $CDCl_3$) δ/ppm 164.71, 164.43, 163.65, 163.30, 138.53, 137.80, 134.56, 134.17, 131.07, 130.23, 128.06, 127.83, 127.64, 123.89, 123.35, 122.72, 122.18, 119.96, 106.00, 105.92, 62.35, 61.60, 25.77, 21.00, 20.92, 20.41, -0.23, -5.30, -5.42.

HRMS (ESI$^+$) found 983.4671, $[C_{56}H_{75}N_2O_6Si_4]^+$ requires 983.4697 (Error: -2.59 ppm).

TBSO-D-Valinol-bis-TMS-acetylene-PDI 1b

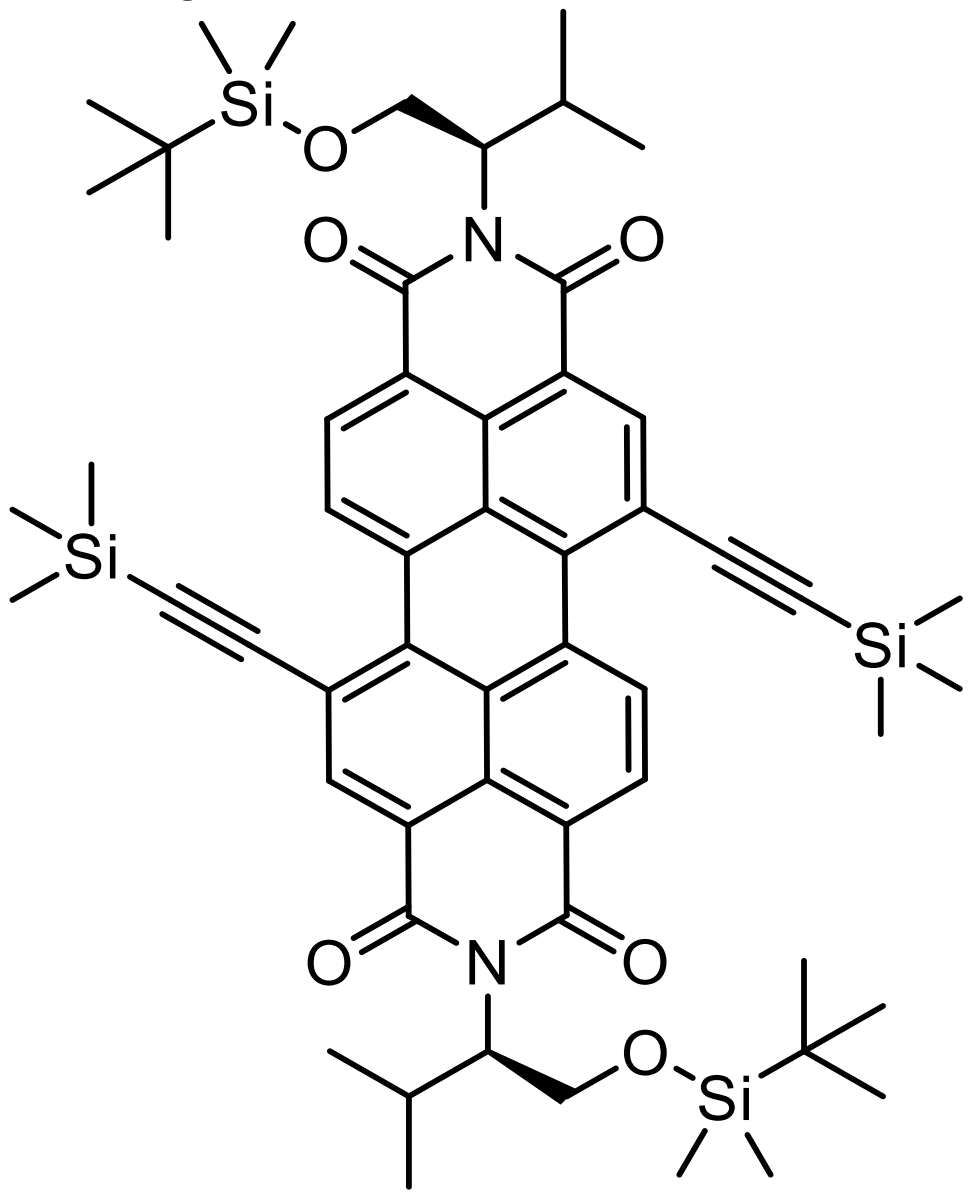

Synthesis analogous to L-isomer: TBSO-D-Val-dibromide **S2** (387 mg, 0.408 mmol), $Pd(PPh)_2Cl_2$ (14.3 mg, 5 mol%) and CuI (7.7 mg, 10 mol%) were added to a round-bottom flask equipped with a stirrer bar and dissolved in 10 mL of a 1:1 mixture of PhMe and NEt_3. The mixture was then degassed by bubbling of N_2 for 30 minutes, before addition of TMS-acetylene (0.394 mL, 2.766 mmol, 6 eq.). The reaction was stirred at room temperature for 4 h. The solvent was then removed *in vacuo* and the resulting residue purified by flash column chromatography (SiO_2, 5% EtOAc/PE_{40-60}) to yield the title compound as a pink solid (329 mg, 0.335 mmol, 82%).

^{1}H NMR (400 MHz, $CDCl_3$) δ/ppm 10.23 (d, *J* = 8.3 Hz, 2H), 8.81 (d, *J* = 21.3 Hz, 2H), 8.64 (dd, *J* = 20.3, 8.3 Hz, 2H), 5.02 (td, *J* = 9.9, 5.1 Hz, 2H), 4.34 (q, *J* = 6.7, 3.4 Hz, 2H), 4.07 (dd, *J* = 10.2, 5.2 Hz, 2H), 2.67 (dq, *J* = 12.0, 6.5 Hz, 2H), 1.13 (d, *J* = 6.6 Hz, 6H), 0.89 (d, *J* = 6.7 Hz, 6H), 0.67 (s, 18H), 0.39 (s, 17H), -0.02 (s, 6H), -0.11 (s, 6H).

^{13}C NMR (101 MHz, $CDCl_3$) δ/ppm 164.72, 164.43, 163.64, 163.30, 138.53, 137.80, 134.56, 134.17, 131.07, 130.22, 128.05, 127.83, 127.63, 123.88, 123.34, 122.72, 122.17, 119.94, 105.99, 105.91, 62.35, 61.59, 25.77, 21.00, 20.93, 20.41, 18.06, -0.23, -5.30, -5.42.

HRMS (ESI$^+$) found 983.4672, $[C_{56}H_{75}N_2O_6Si_4]+$ requires 983.4697 (Error: -2.43 ppm).

Biphenyl azide S3

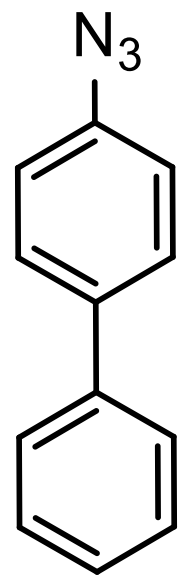

Following a literature procedure:[2] In a round-bottom flask, biphenyl bromide (1.17 g, 5 mmol), copper iodide (952 mg, 5 mmol), DMEDA (538 µL, 5 mmol) and sodium azide (650 mg, 10 mmol) were dissolved in EtOH (14 mL) and H_2O (6 mL). To this was then added sodium ascorbate (990 mg, 5 mmol) and the reaction heated to reflux for 3 h, when TLC indicated full conversion to the desired product. The mixture was then cooled to room temperature and diluted with ethyl acetate (50 mL) and water (50 mL). The layers were separated and the aqueous layer extracted with ethyl acetate (2x 20 mL), the organic layers combined, washed with water (1x 50 mL) ammonium chloride (2x 50 mL) and brine (1x 50 mL), dried ($MgSO_4$) and reduced *in vacuo* to yield a pale-yellow solid as the desired product, (1 g, 99%), with traces of ethyl acetate, which was used without further purification.

^{1}H NMR (300 MHz, $CDCl_3$) δ 7.64 – 7.50 (m, 4H), 7.50 – 7.29 (m, 4H), 7.15 – 7.06 (m, 1H). Spectral data matches literature data.[3]

TBSO-LVal-biphenyl triazole-PDI 2a

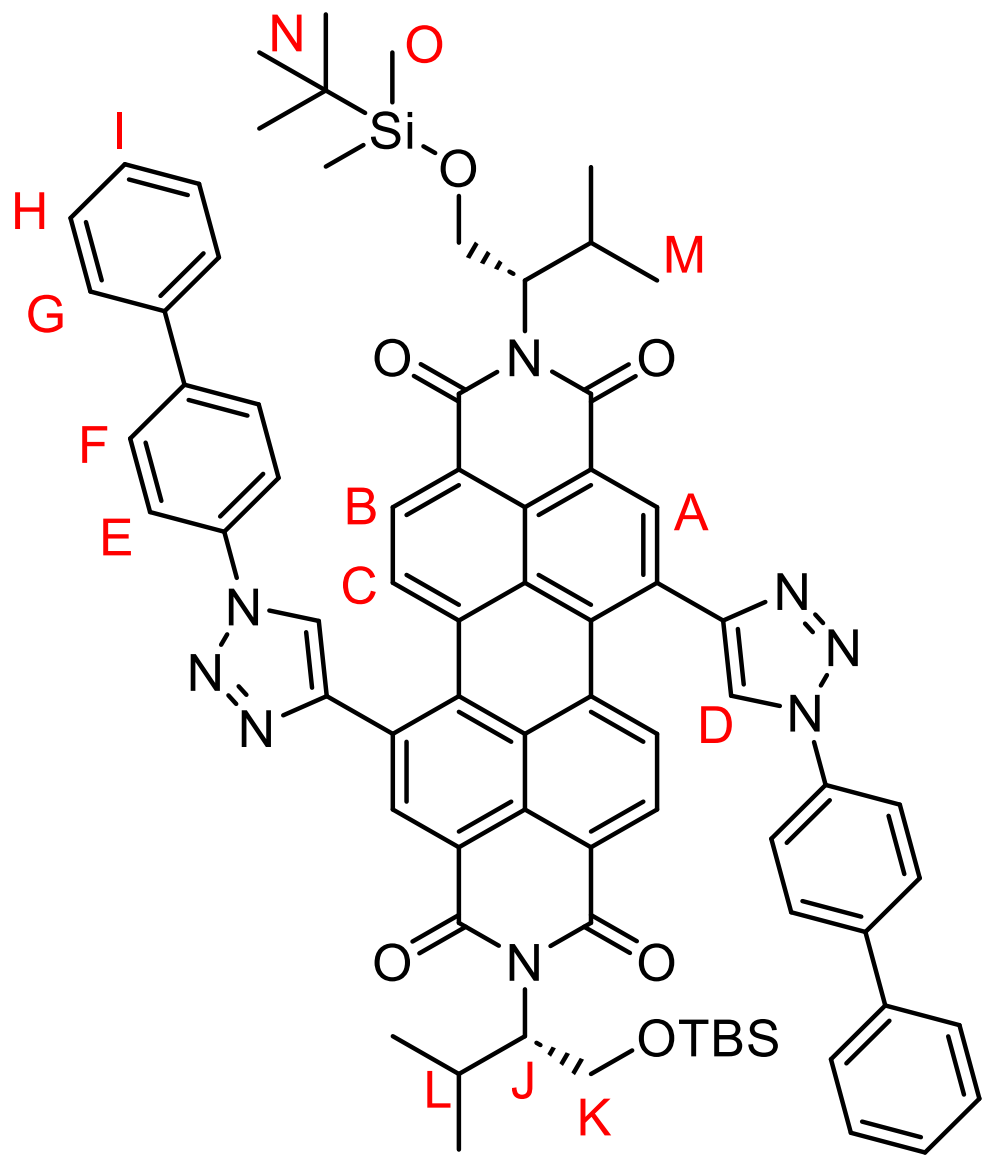

TBSO-L-Valinol-bis-TMS-acetylene-PDI **1a** (600 mg, 610 μmol) was dissolved in 2:1 DCM:MeOH (15 mL). To this was added K_2CO_3 (303 mg, 2.2 mmol, 3.6 eq.) and the reaction stirred for 10 minutes, when TLC indicated full deprotection. The reaction mixture was then diluted with DCM (20 mL) and washed with water (2x 50 mL) and brine (50 mL), dried (MgSO4) and reduced *in vacuo*. The residue was then redissolved in DCM (20 mL). To this was added biphenyl azide (714 mg, 3.66 mmol, 6 eq.) and the solution degassed with N_2 for 30 mins. TBTA (65 mg, 20 mol%) and $Cu(MeCN)_4PF_6$ (45 mg, 20 mol%) were then added and the reaction left stirring for 4 days. The solvent was subsequently removed and the residue purified by flash column chromatography (SiO_2, 1-2% Acetone/DCM) to yield the desired molecule as a purple solid (632 mg, 514 μmol, 84%).

^{1}H NMR (400 MHz, $CDCl_3$) δ/ppm 8.79 (d, *J* = 18.8 Hz, 2H, **A**), 8.40 – 8.24 (m, 4H, **B**, **C**), 8.12 (d, *J* = 8.1 Hz, 2H, **D**), 7.98 – 7.83 (m, 4H, **E**), 7.79 (d, *J* = 8.2 Hz, 4H, **F**), 7.64 (7.64 (d, *J* = 7.4 Hz, 4H, **G**), 7.49 (dd, *J* = 8.4, 6.9 Hz, 4H, **H**), 7.45 – 7.36 (m, 2H, **I**), 5.01 (dh, *J* = 9.4, 4.3 Hz, 2H, **J**), 4.33 (t, *J* = 9.9 Hz, 2H, **K'**), 4.06 (dd, *J* = 10.2, 5.2 Hz, 2H, **K''**), 2.65 (tq, *J* = 12.7, 6.5 Hz, 2H, **L**), 1.11 (d, *J* = 6.6 Hz, 6H, **M'**), 0.88 (d, *J* = 6.7 Hz, 6H, **M''**), 0.70 (d, *J* = 4.4 Hz, 18H, **N**), -0.02 (s, 6H, **O'**), -0.09 (s, 6H, **O''**).

^{13}C NMR (101 MHz, $CDCl_3$) δ/ppm 164.62, 163.52, 148.83, 142.38, 139.56, 135.81, 135.42, 134.64, 133.71, 130.55, 129.74, 129.51, 129.24, 129.15, 128.67, 128.62, 128.22, 127.22, 123.35, 123.26, 122.78, 122.72, 120.86, 120.02, 62.31, 61.74, 27.56, 25.83, 20.91, 20.37, 18.10, -5.28, -5.38.

HRMS (ESI$^+$) found 1229.5514, $[C_{74}H_{77}N_8O_6Si_2]^+$ needs 1229.5499 (Error: 1.48 ppm).

TBSO-DVal-biphenyl triazole-PDI 2b

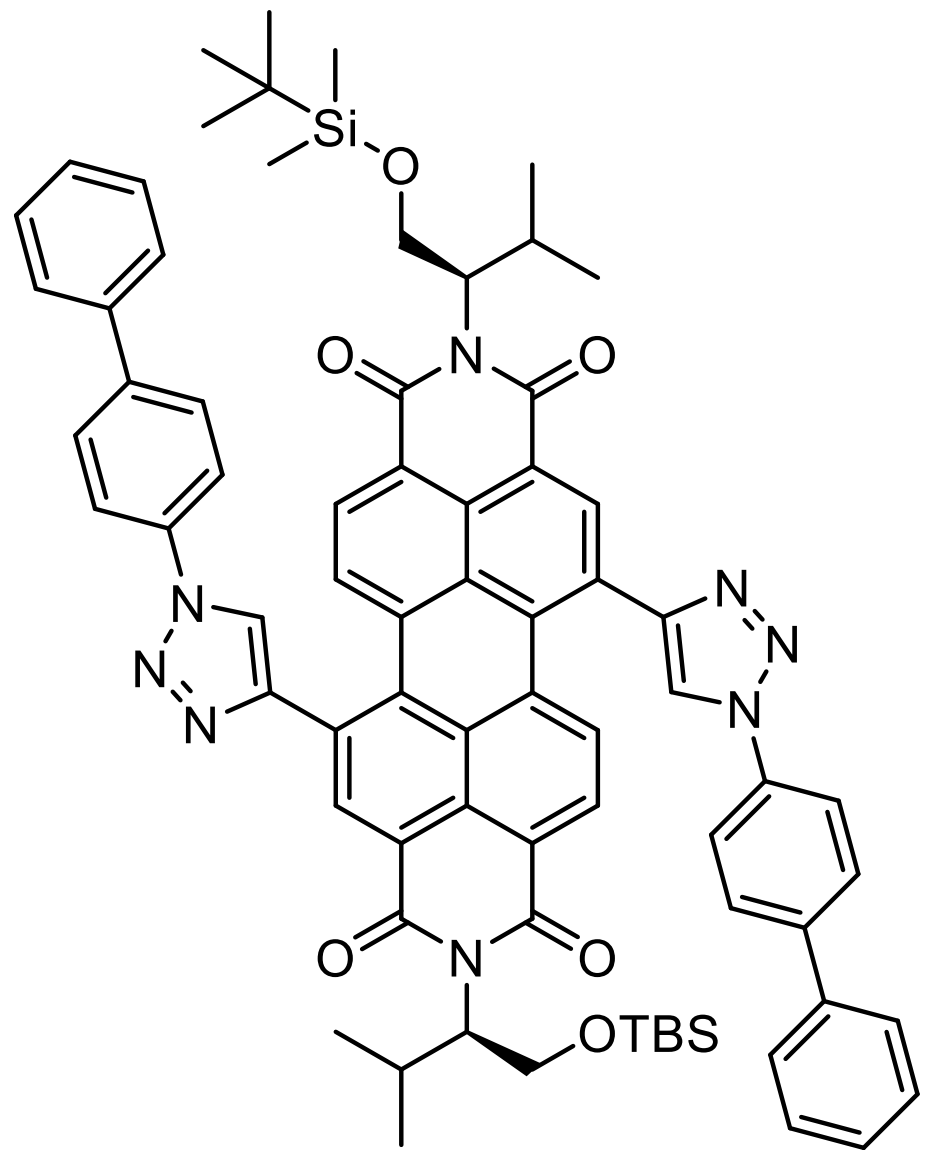

Analogous to L-isomer: TBSO-L-Valinol-bis-TMS-acetylene-PDI **1b** (200 mg, 203 μmol) was dissolved in 2:1 DCM:MeOH (15 mL). To this was added K_2CO_3 (101 mg, 0.73 mmol, 3.6 eq.) and the reaction stirred for 15 minutes, when TLC indicated full deprotection. The reaction mixture was then diluted with DCM (20 mL) and washed with water (2x 50 mL) and brine (50 mL), dried ($MgSO_4$) and reduced *in vacuo*. The residue was then redissolved in DCM (20 mL). To this was added biphenyl azide (238 mg, 1.22 mmol, 6 eq.) and the solution degassed with N_2 for 30 mins. TBTA (27 mg, 25 mol%) and $Cu(MeCN)_4PF_6$ (19 mg, 25 mol%) were then added and the reaction left stirring overnight. The solvent was subsequently removed and the residue purified by flash column chromatography (SiO_2, 1-2% Acetone/DCM) to yield the desired molecule as a purple solid (249 mg, 202 μmol, 99%).

^{1}H NMR (400 MHz, $CDCl_3$) δ/ppm 8.79 (d, *J* = 18.3 Hz, 2H), 8.46 – 8.21 (m, 4H), 8.12 (d, *J* = 8.1 Hz, 2H), 7.90 (d, *J* = 8.2 Hz, 4H), 7.84 – 7.74 (m, 4H), 7.64 (d, *J* = 7.5 Hz, 4H), 7.49 (t, *J* = 7.6 Hz, 4H), 5.01 (dq, *J* = 10.4, 5.6, 4.9 Hz, 2H), 4.34 (t, *J* = 9.8 Hz, 2H), 4.07 (dd, *J* = 10.3, 5.2 Hz, 2H), 2.66 (dd, *J* = 16.1, 8.5 Hz, 2H), 1.12 (d, *J* = 6.6 Hz, 6H), 0.89 (d, *J* = 6.7 Hz, 6H), 0.70 (d, *J* = 3.6 Hz, 18H), 0.06 (s, 6H), -0.01 (s, 6H), -0.08 (s, 6H).

^{13}C NMR (101 MHz, $CDCl_3$) δ/ppm 164.63, 163.53, 148.83, 142.40, 139.57, 135.82, 135.43, 134.64, 133.72, 130.55, 129.76, 129.52, 129.25, 129.16, 128.68, 128.62, 128.22, 127.23, 123.27, 122.78, 120.88, 120.03, 62.33, 61.75, 27.62, 25.84, 20.90, 20.37, 18.11, -5.27, -5.35.

HRMS (ESI$^+$) found 1229.5457, $[C_{74}H_{77}N_8O_6Si_2]^+$ needs 1229.5499
(Error: -3.42 ppm).

TBSO-LVal-biphenyl triazole-CDI 3a

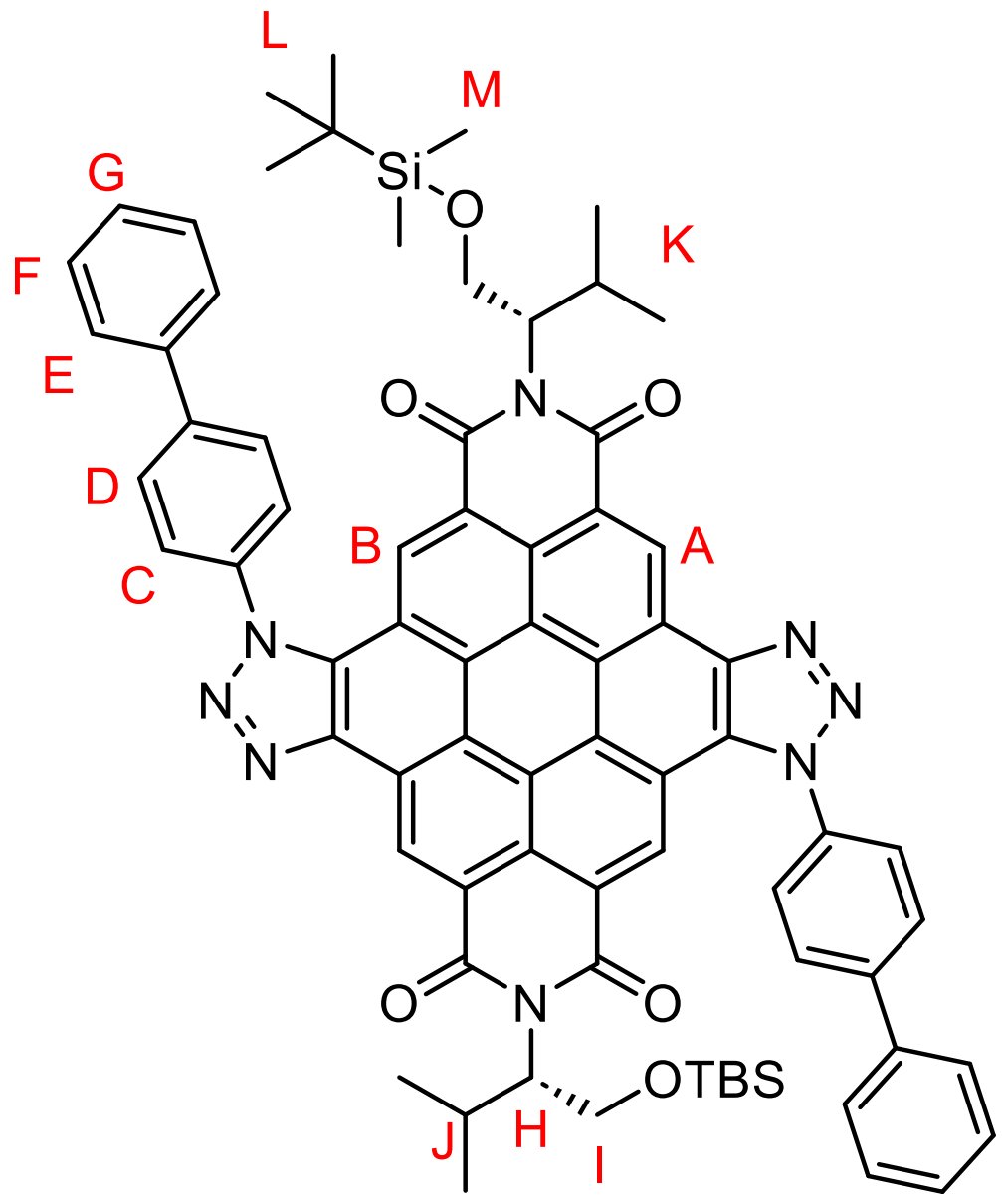

In a vial equipped with a stirrer bar, PDI **2a** (32 mg, 26 µmol) was dissolved in 35 mL of $CHCl_3$. The sample was then irradiated at 525 nm for 1 hour in a Photocube reactor (ThalesNano) at 35 °C and 1000 rpm stirring speed. The solvent was subsequently removed and the residue purified by flash column chromatography (SiO_2, CH_2Cl_2), followed by preparative thin layer chromatography (CH_2Cl_2) to yield the desired compound as an orange solid (13 mg, 10.6 µmol, 41%).

1H NMR (400 MHz, TCE-d_2, 373 K) δ/ppm 10.93 (s, 2H, **A**), 9.90 (s, 2H, **B**), 8.22 – 8.16 (m, 4H), 8.14 – 8.07 (m, 4H), 7.95 (dd, *J* = 8.2, 1.4 Hz, 4H), 7.68 (dd, *J* = 8.3, 6.9 Hz, 4H), 7.62 – 7.56 (m, 2H, **G**), 5.28 (td, *J* = 9.7, 5.1 Hz, 2H, **H**), 4.57 (t, *J* = 9.8 Hz, 2H, **I'**), 4.23 (dd, *J* = 10.2, 5.3 Hz, 2H, **I''**), 2.96 – 2.82 (m, 2H, **J**), 1.27 (d, *J* = 6.6 Hz, 6H, **K'**), 1.11 (d, *J* = 6.7 Hz, 6H, **K''**), 0.69 (d, *J* = 0.7 Hz, 18H, **L**), 0.07 (s, 6H, **M'**), -0.04 (s, 6H, **M''**).

^{1}H NMR (400 MHz, $CDCl_3$) δ/ppm 10.93 (d, *J* = 22.4 Hz, 2H), 9.91 (s, 2H), 8.25 – 8.00 (m, 8H), 7.88 (d, *J* = 7.4 Hz, 4H), 7.63 (t, *J* = 7.6 Hz, 4H), 7.54 (t, *J* = 7.5 Hz, 2H), 5.23 (d, *J* = 44.5 Hz, 2H), 4.48 (s, 2H), 4.15 (s, 2H), 2.84 (d, *J* = 61.1 Hz, 2H), 1.20 (s, 6H), 1.03 (dd, *J* = 6.0, 2.5 Hz, 6H), 0.56 (s, 9H), -0.01 (s, 6H), -0.14 (s, 6H).

^{13}C NMR (101 MHz, TCE-d_2, 373 K) δ/ppm 164.35, 164.09, 144.41, 142.05, 139.61, 136.37, 129.87, 129.01, 128.97, 128.25, 127.40, 126.76, 126.22, 124.63, 124.29, 124.22, 123.61, 123.55, 122.97, 122.76, 120.21, 117.94, 62.69, 61.59, 27.66, 25.47, 20.55, 20.26, 17.65, -5.58, -5.69.

HRMS (ESI^+) found 1247.4957, $[C_{74}H_{72}N_8O_6Si_2Na]^+$ needs 1247.5006 (Error: -4.90 ppm).

TBSO-DVal-biphenyl triazole-CDI 3b

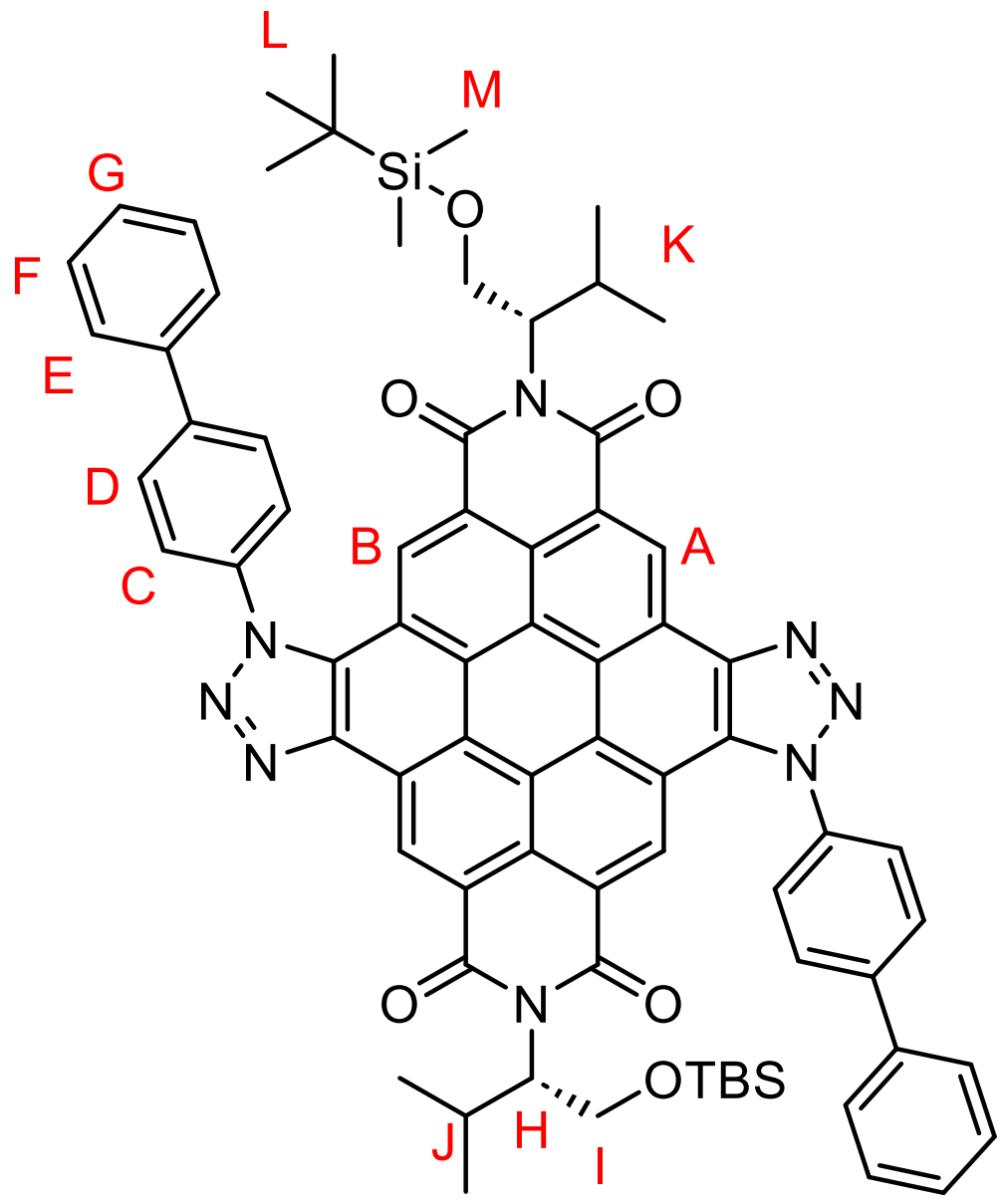

In a vial equipped with a stirrer bar, PDI **2b** (100 mg, 81 μmol) was dissolved in 100 mL of $CHCl_3$. The sample was then irradiated at 525 nm for 2 hours in a Photocube reactor (ThalesNano) at 35 °C and 1000 rpm stirring speed. The solvent was subsequently removed and the residue purified by flash column chromatography (SiO_2, CH_2Cl_2), to yield the desired compound as an orange solid (31 mg, 25 μmol, 31%).

^{13}C NMR (101 MHz, TCE-d_2, 298 K) δ/ppm 165.13, 164.79, 163.80, 163.23, 144.01, 141.93, 139.27, 136.11, 129.56, 129.17, 129.06, 128.47, 127.46, 126.78, 125.94, 124.53, 124.07, 123.24, 122.66, 122.21, 117.78, 62.58, 62.16, 61.07, 27.73 – 26.90 (m), 25.48, 20.66 (d, J = 35.3 Hz), 17.66, -5.49, -5.59.

^{1}H NMR (101 MHz, TCE-d_2, 298 K) δ/ppm 10.86 (d, J = 22.2 Hz, 2H), 9.97 – 9.72 (m, 2H), 8.17 (d, J = 7.8 Hz, 4H), 8.13 – 8.06 (m, 4H), 7.94 (d, J = 7.4 Hz, 4H), 7.67 (t, J = 7.4 Hz, 4H), 7.58 (t, J = 7.5 Hz, 2H), 5.36 – 5.02 (m, 2H), 4.60 – 4.41 (m, 2H), 4.18 (m, 2H), 2.92 (s, H), 2.76 (s, 1H), 1.23 (m, 6H), 0.88 (m, 6H), 0.55 (m, 18H), 0.05 – -0.05 (m, 6H), -0.05 – -0.22 (m, 6H).

HRMS (ESI$^+$) found 1247.4986, $[C_{74}H_{72}N_8O_6Si_2Na]^+$ needs 1247.5006 (Error: -1.97 ppm).

OH-LVal-biphenyl triazole-CDI S4a

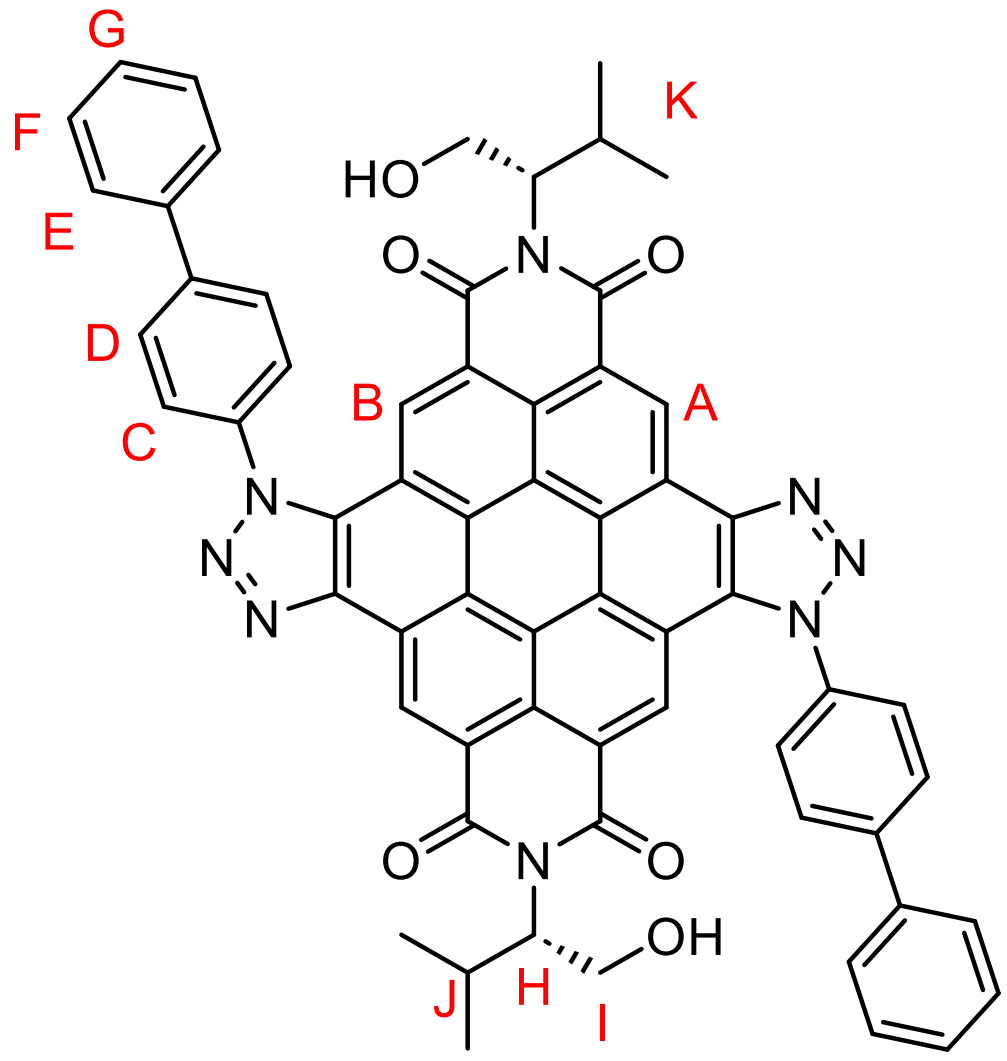

In a round-bottom flask under nitrogen atmosphere, CDI **3a** (51 mg, 41.6 µmol) was dissolved in $CHCl_3$ (2 mL). To this was added HCl in Et_2O (2 M, 2 mL) and the reaction stirred until TLC indicated complete conversion. The solvent was subsequently removed *in vacuo* and the residue purified by flash column chromatography (SiO_2, 0-10% MeOH/DCM) to yield a poorly soluble orange solid as the desired product (36.5 mg, 36.6 µmol, 88%).

HRMS (ESI^+) found 997.3431, $[C_{62}H_{45}N_8O_6]^+$ requires 997.3456 (Error: -2.51 ppm).

The compound was too insoluble to be characterised by NMR.

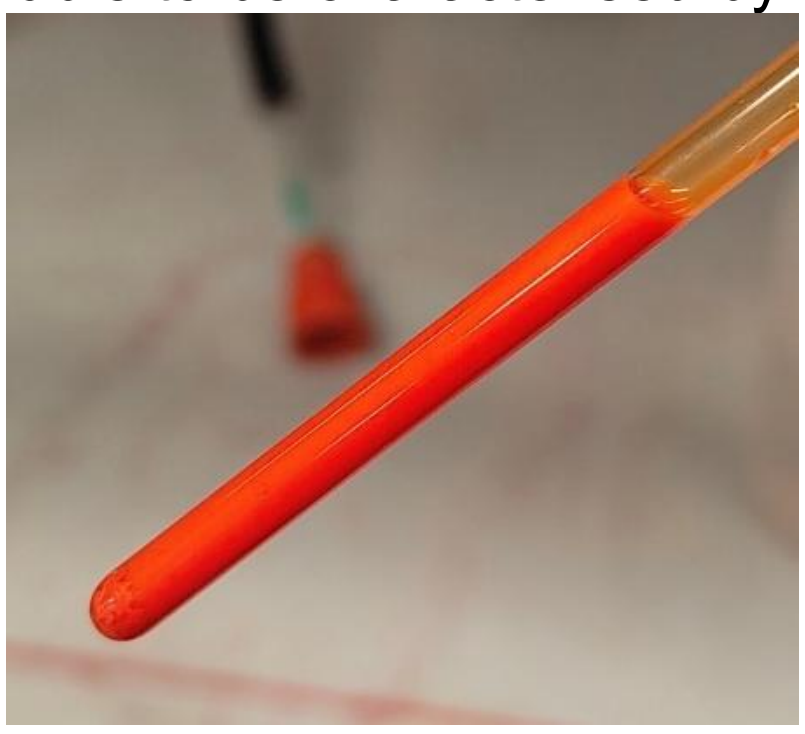

OH-DVal-biphenyl triazole-CDI S4b

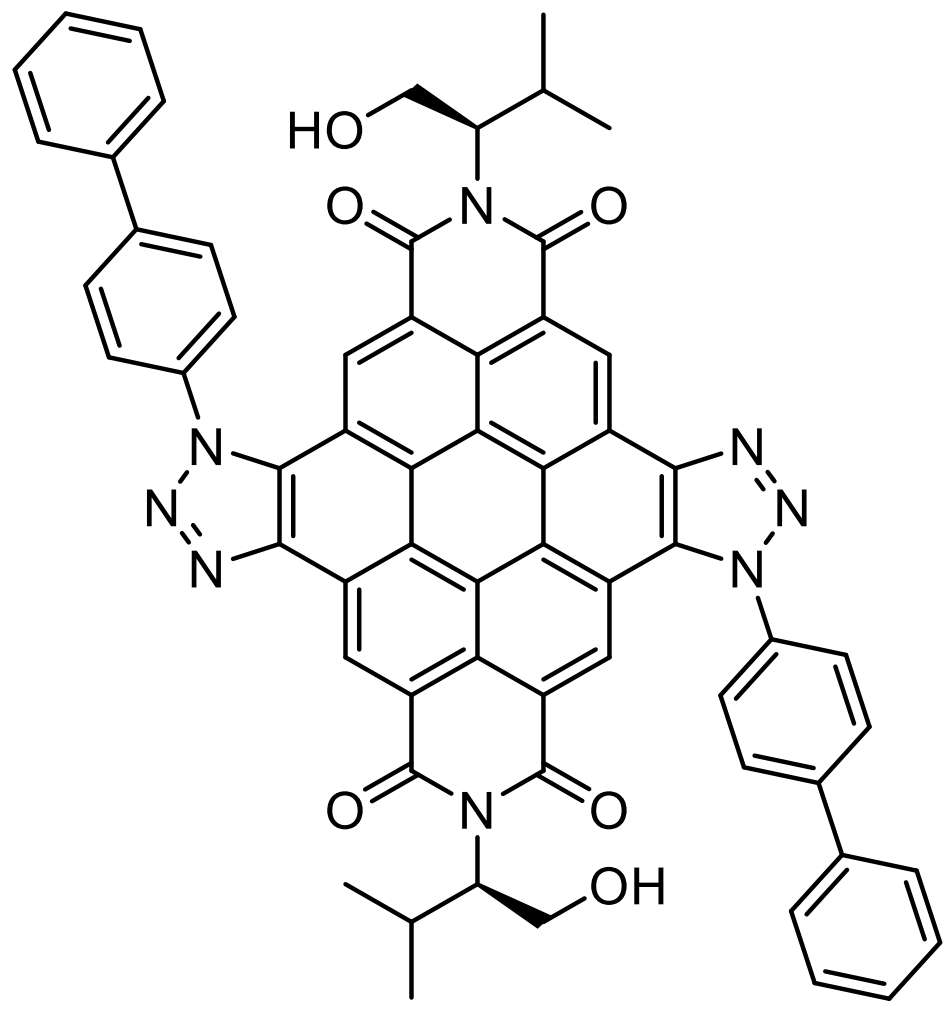

In a round-bottom flask under nitrogen atmosphere, CDI **3b** (31 mg, 25.29 μmol) was dissolved in $CHCl_3$ (3 mL). To this was added HCl in Et_2O (2 M, 3 mL) and the reaction stirred until TLC indicated essentially complete conversion. The solvent was subsequently in vacuo and the residue purified by flash column chromatography (SiO_2, 0-25% MeOH/DCM) to yield a poorly soluble orange solid as the desired product (25 mg, 25.03 μmol, 99%).

HRMS (ESI^+) found 997.3487, $[C_{62}H_{45}N_8O_6]^+$ requires 997.3456 (Error: 3.11 ppm).

The compound was too insoluble to be characterised by NMR.

Octanoyl-LVal-biphenyl triazole-CDI 4a

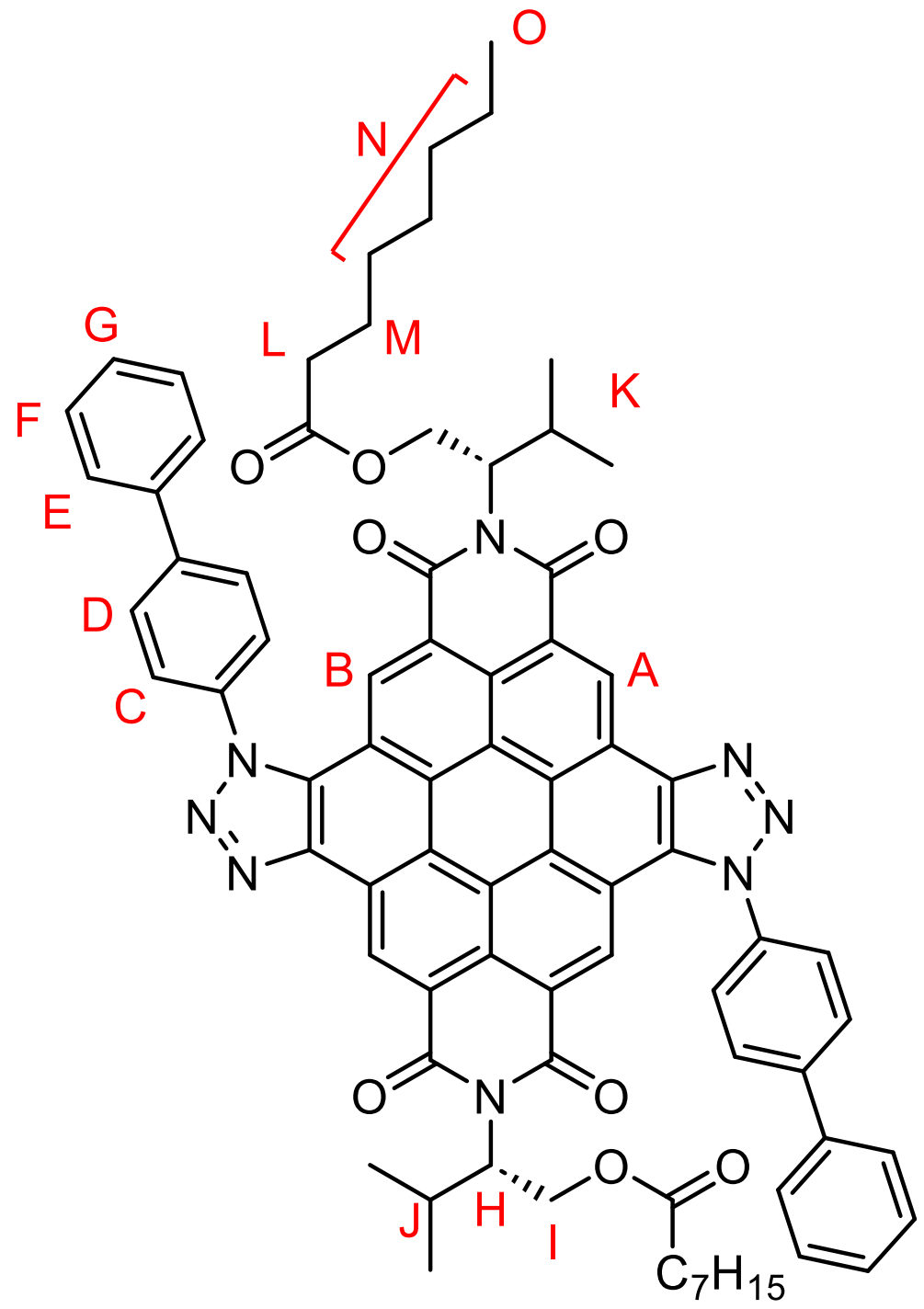

In a flame-dried round-bottom flask under nitrogen atmosphere, CDI-alcohol **S4a** (20 mg, 20 µmol) was suspended in dry CH_2Cl_2 (12 mL). To this was added dry pyridine (81 µL, 1 mmol), octanoyl chloride (86 µL, 502 µmol) and a small grain of DMAP. The reaction was left stirring for 16 hours, during which time the material became completely soluble and TLC indicated complete conversion. The solvent was subsequently removed and the residue purified by flash column chromatography (SiO_2, 0-5% EtOAc/ CH_2Cl_2) to yield a bright orange solid as the desired product (20.5 mg, 16.4 µmol, 82%).

^{1}H NMR (400 MHz, $CDCl_3$) δ/ppm 10.88 (s, 2H, **A**), 9.86 (apparent d, *J* = 23.8 Hz, 2H, **B**), 8.12 (d, *J* = 8.1 Hz, 4H, **C**), 8.06 (d, *J* = 8.1 Hz, 4H, **D**), 7.89 (d, *J* = 7.4 Hz, 4H, **E**), 7.64 (t, *J* = 7.5 Hz, 4H, **F**), 7.55 (t, *J* = 7.4 Hz, 2H, **G**), 5.34 and 5.21 (m, rotameric C-H, 2H. **H**), 4.98 (m, 2H, **I'**), 4.81 (m, 2H, **I''**), 2.96 and 2.86 (m, rotameric C-H, 2H, **J**), 2.24 and 2.17 (m, rotameric CH_2, 4H, **L**), 1.52 – 1.38 (m, 4H, **M**), 1.28 (d, *J* = 6.8 Hz, 6H, **K'**), 1.16 – 0.73 (m, 22H, **K''** and **N**), 0.53 (s, 6H, **O**).

^{13}C NMR (101 MHz, $CDCl_3$) δ/ppm 173.91, 173.60, 164.72, 164.55, 163.82, 163.47, 144.54, 142.10, 139.74, 136.35, 129.58, 129.37, 128.62, 127.73, 127.04, 126.59, 124.79, 124.50, 124.28, 123.54, 122.88, 122.56, 122.20, 118.03, 63.69, 59.92, 34.38, 31.53, 29.07, 28.91, 28.20, 27.76, 24.96, 22.47, 20.83, 20.33, 13.91.

HRMS (ESI$^+$) found 1249.5500, $[C_{78}H_{73}N_8O_8]^+$ requires 1249.5546 (Error: -3.7 ppm).

Octanoyl-DVal-biphenyl triazole-CDI 4b

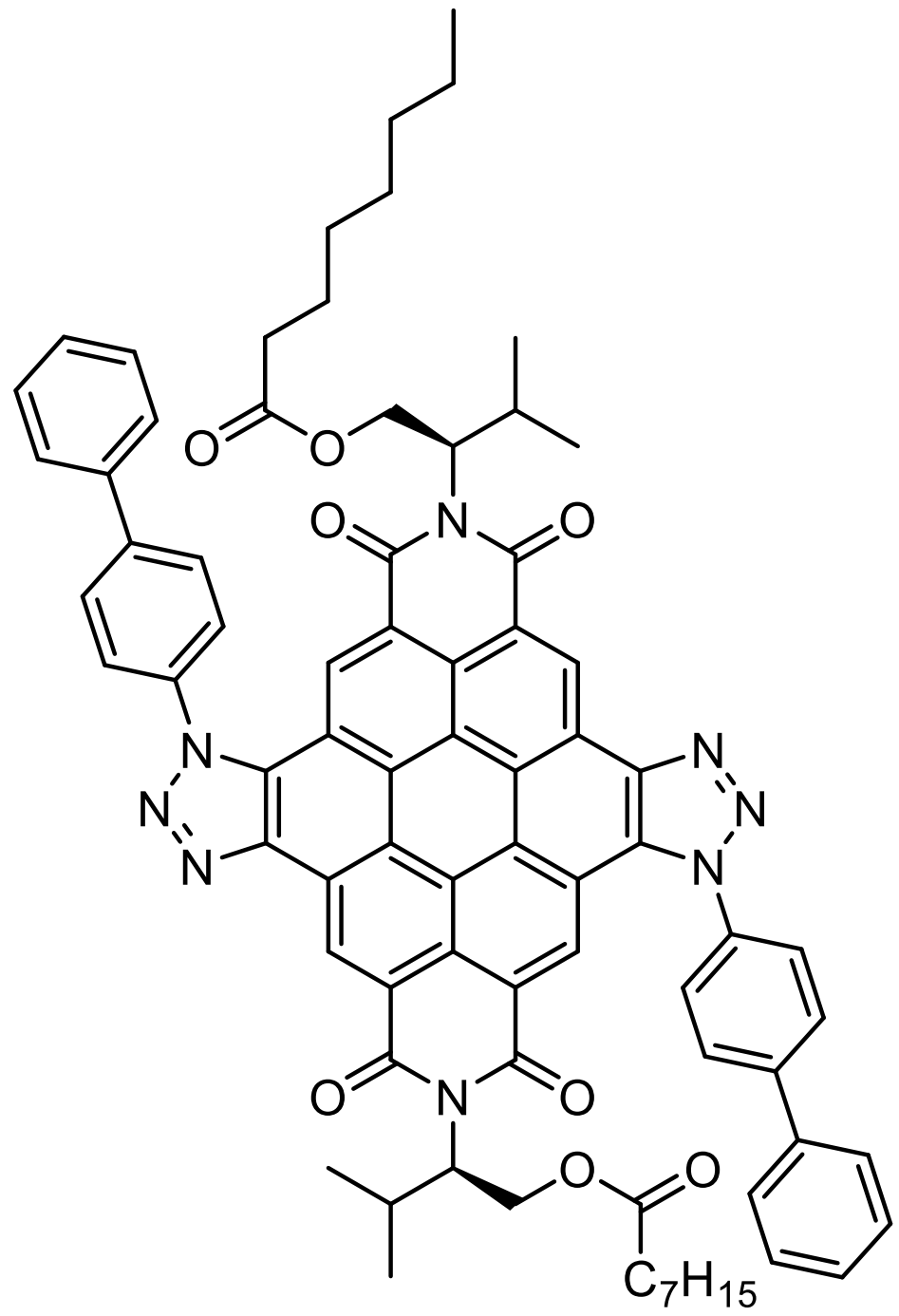

In a scintillation vial under nitrogen atmosphere, CDI-alcohol **S4b** (27 mg, 27 µmol) was suspended in dry CH_2Cl_2 (5 mL). To this was added dry pyridine (55 µL, 677 µmol), octanoyl chloride (116 µL, 677 µmol) and a small grain of DMAP. The reaction was left stirring for 4 hours, during which time the material became completely soluble and TLC indicated complete conversion. The solvent was subsequently removed and the residue purified by flash column chromatography (SiO_2, 0-5% EtOAc/$CHCl_3$) and further purified by precipitation with Et_2O to yield a bright orange solid as the desired product (22 mg, 17.61 µmol, 65%).

^{1}H NMR (400 MHz, $CDCl_3$) δ/ppm 10.87 (d, *J* = 15.6 Hz, 2H), 9.87 (d, *J* = 24.8 Hz, 2H), 8.12 (d, *J* = 8.2 Hz, 4H), 8.06 (d, *J* = 8.3 Hz, 4H), 7.89 (d, *J* = 7.4 Hz, 4H), 7.65 (t, *J* = 7.6 Hz, 4H), 7.59 – 7.52 (m, 2H), 5.42 – 5.12 (m, 2H), 4.97 (d, *J* = 11.4 Hz, 2H), 4.79 (d, *J* = 13.4 Hz, 2H), 2.91 (d, *J* = 39.4 Hz, 2H), 2.32 – 2.06 (m, 4H), 1.51 – 1.34 (m, 4H), 1.33 – 1.21 (m, 6H), 1.12 – 0.77 (m, 22H), 0.53 (d, *J* = 8.9 Hz, 6H).

^{13}C NMR (101 MHz, $CDCl_3$) δ/ppm 173.89, 173.59, 164.77, 164.60, 163.85, 163.49, 144.58, 142.15, 139.76, 136.37, 129.65, 129.37, 128.62, 127.75, 127.07, 126.64, 124.88, 124.58, 124.34, 123.60, 122.97, 122.65, 122.27, 118.09, 63.66, 59.90, 34.38, 31.53, 29.08, 28.91, 28.18, 27.75, 24.96, 22.47, 20.83, 20.32, 13.91.

HRMS (ESI$^+$) found 1249.5513, $[C_{78}H_{73}N_8O_8]^+$ requires 1249.5546 (Error: -3.34 ppm).

2) Spectroscopy

UV-Visible absorbance spectra were recorded on a Shimadzu UV-3600i Plus Spectrometer with a wavelength accuracy of ± 0.2 nm in the visible and UV region, a baseline flatness of ± 0.002 nm (200 – 3000 nm) and a noise level of <0.00008 Abs (900 nm) or a Cary50 UV-vis spectrometer.
Fluorescence spectra were recorded on a Cary Eclipse fluorescence spectrometer. Quantum yields were measured on an Edinburgh FLS1000 Spectrophotometer equipped with an integrating sphere using a Xe lamp and a PMT-980 detector. Slit widths were adjusted to yield ~10^6 counts at the excitation wavelength. Samples were measured at an OD of ~0.1 at the excitation wavelength and emission spectra recorded with 0.1 nm step size and 0.2 nm dwell time.
Circular Dichroism measurements were taken on a JASCO J-1500 CD Spectrophotometer with a wavelength accuracy ± 0.2 nm (250 to 500 nm), ± 0.5 nm (500 to 800 nm), a photometric accuracy of ± 0.01 Abs and a CD root mean square noise < 0.007 mdeg (500 nm) or a Chirascan V100. Recorded traces were then baseline corrected and smoothed using the provided software.
CPL was measured with a home-built (modular) spectrometer.[4] The excitation source was a broad band (200 – 1000 nm) laser- driven light source EQ 99 (Elliot Scientific). The excitation wavelength was selected by feeding the broadband light into an Acton SP-2155 monochromator (Princeton Instruments, 7 µm slit); the collimated light was focused into the sample cell (1 cm quartz cuvette, temperature controlled at 20 ºC). Sample PL emission was collected perpendicular to the excitation direction with a lens (f = 150 mm). The emission was fed through a photoelastic modulator (PEM) (Hinds Series II/FS42AA) and through a linear sheet polariser (Comar). The light was then focused into a second scanning monochromator (Acton SP-2155, 7 µm slit) and subsequently on to a photomultiplier tube (PMT) (Hamamatsu H10723 series). The detection of the CPL signal was achieved using the field modulation lock-in technique. The electronic signal from the PMT was fed into a lock-in amplifier (Hinds Instruments Signaloc Model 2100). The reference signal for the lock-in detection was provided by the PEM control unit. The monchromators, PEM control unit and lock-in amplifier were interfaced to a desktop PC and controlled by a custom-written Labview graphic user interface. The lock-in amplifier provided two signals, an AC signal corresponding to (I_L- I_R) and a DC signal corresponding to (I_L + I_R) after background subtraction. The emission dissymmetry factor was therefore readily obtained from the experimental data, as 2 AC/DC.
The emission spectra were recorded with 2 nm step size and the slits of the detection monochromator were set to a slit width corresponding to a spectral resolution of of 3.5 nm. CPL spectra (as well as total emission spectra) were obtained through an averaging procedure of several scans. Samples were prepared to an OD of 0.15 at the excitation wavelength (450 nm).

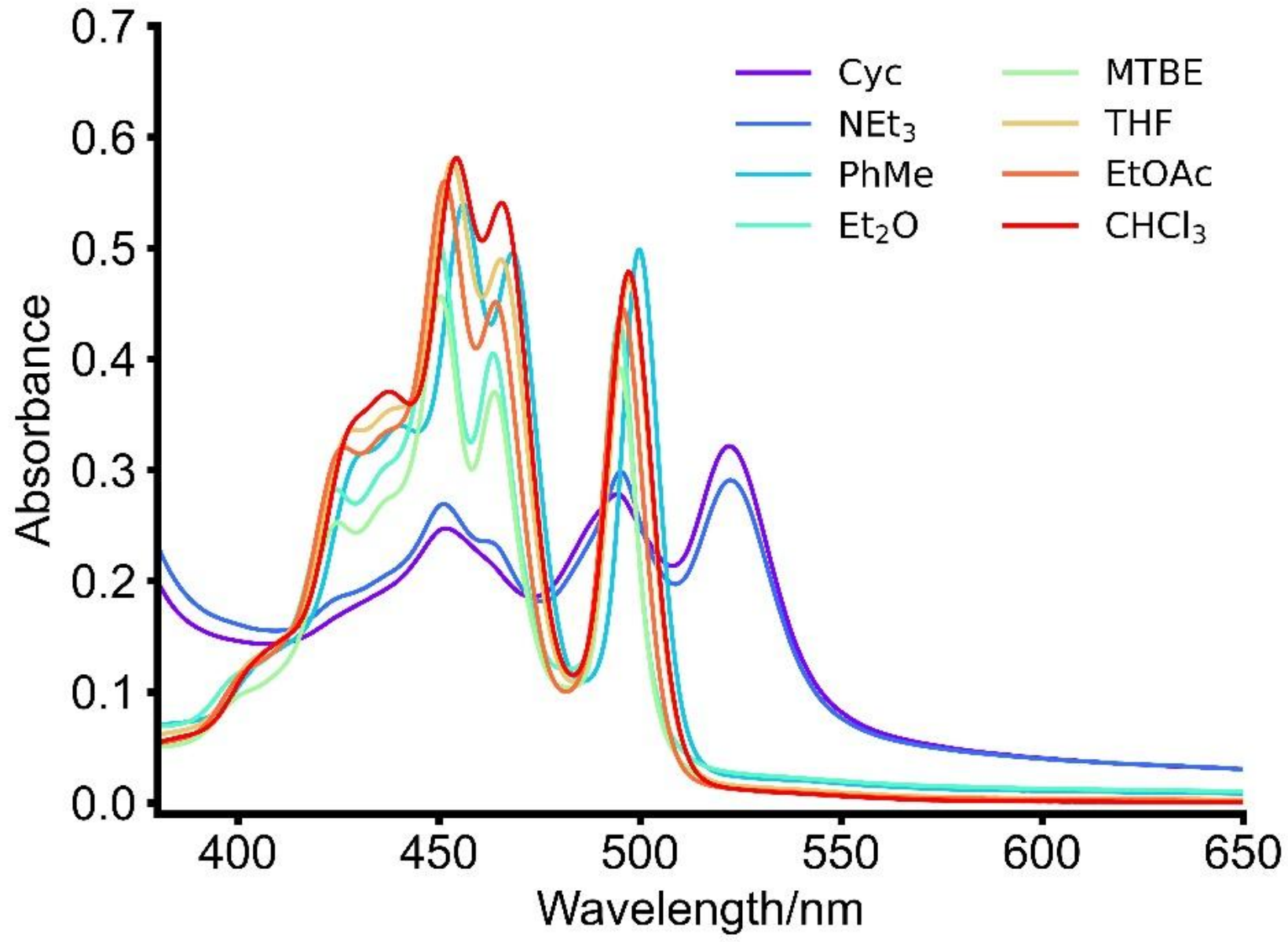

Supplementary Figure 2-1: UV-visible absorption spectra of CDI **4a** in various apolar solvents (Reichardt $E_T(30)$ <40) at 10 µM concentration. Samples were prepared from a 5 mM stock in $CHCl_3$, resulting in 0.2% $CHCl_3$ in each sample and measured immediately upon preparation at room temperature.

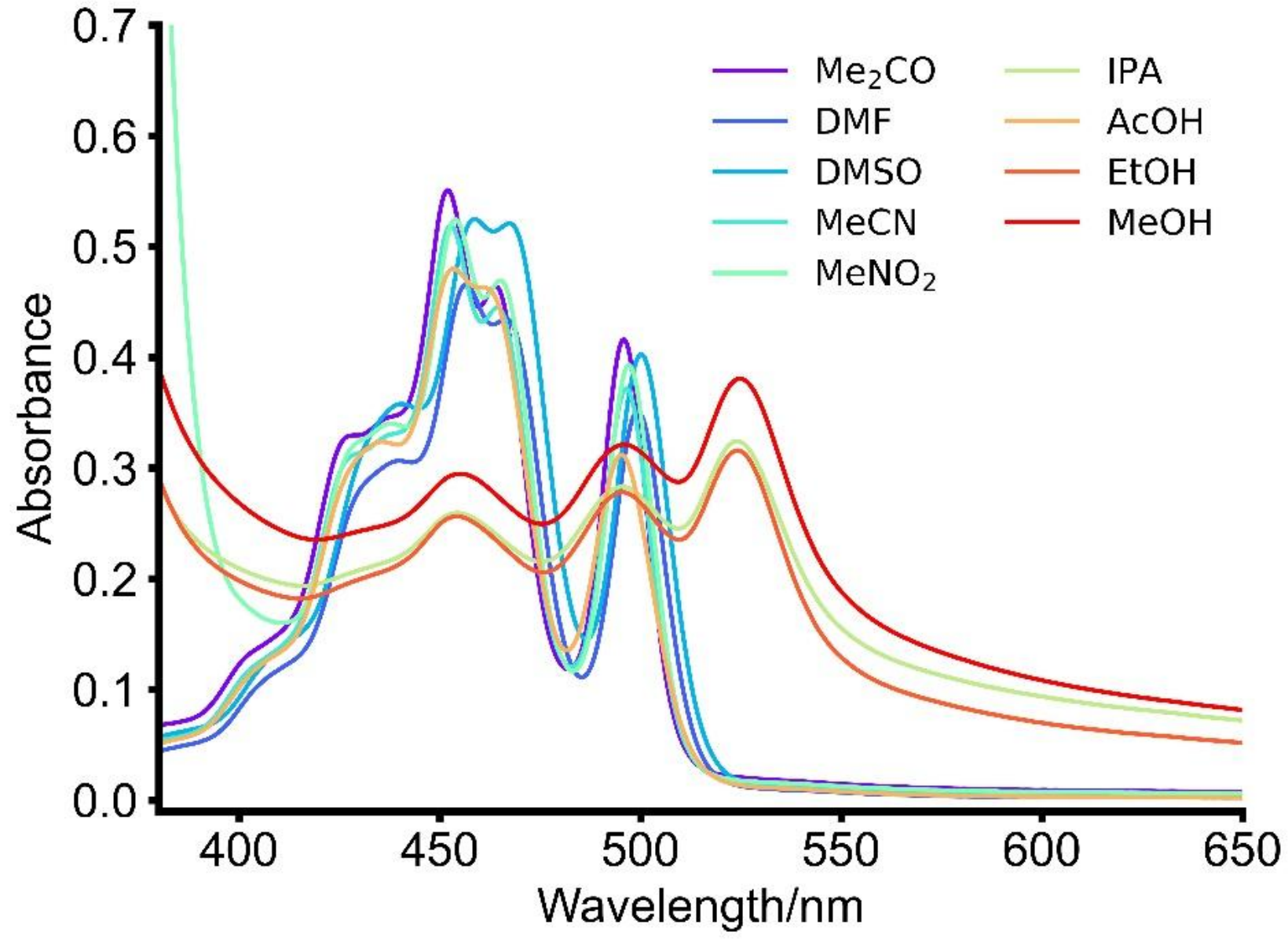

Supplementary Figure 2-2: UV-visible absorption spectra of CDI **4a** in various polar solvents (Reichardt $E_T(30)$ >40) at 10 µM concentration. Samples were prepared from a 5 mM stock in $CHCl_3$, resulting in 0.2% $CHCl_3$ in each sample and measured immediately upon preparation at room temperature.

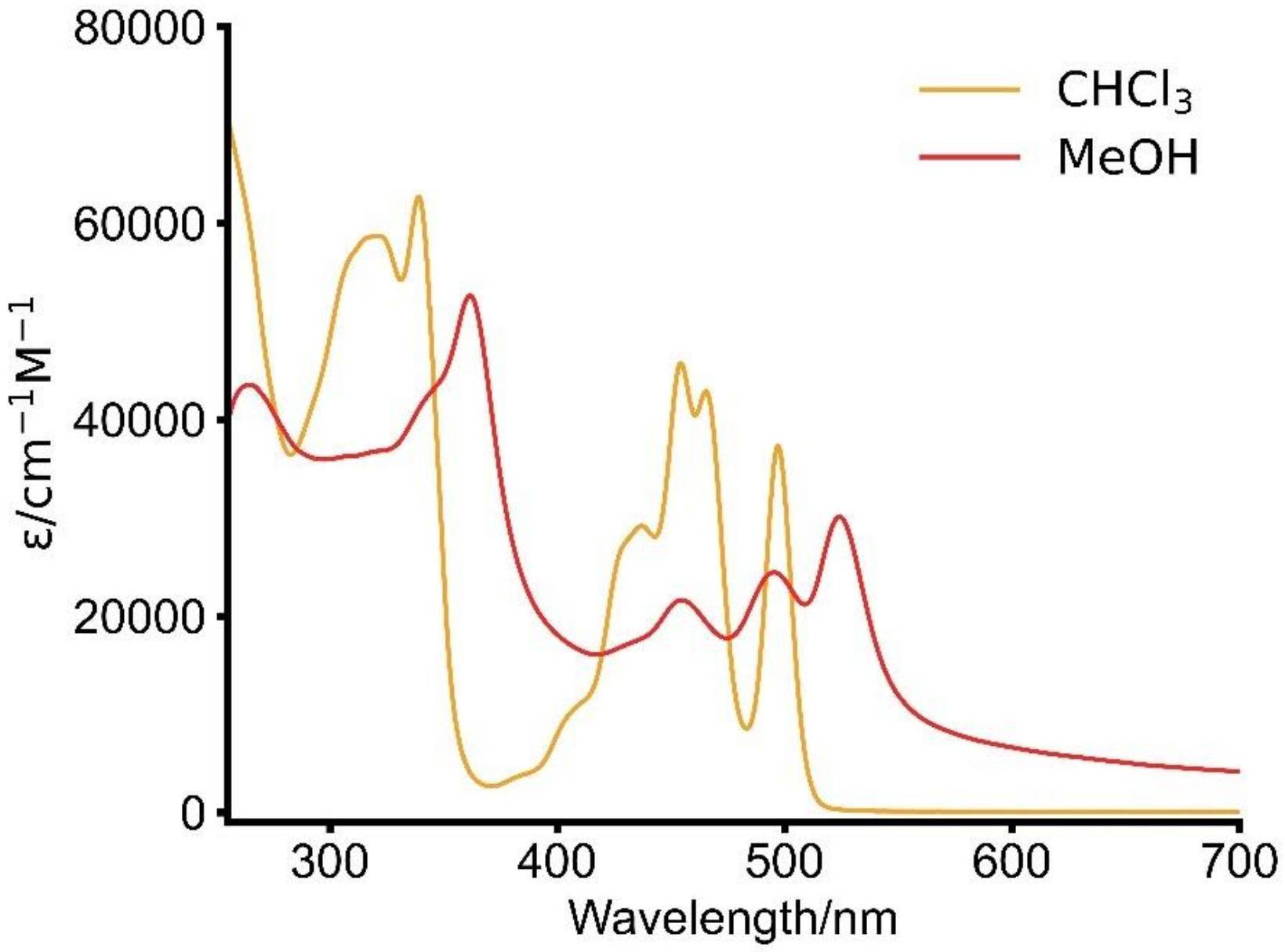

Supplementary Figure 2-3: UV-visible absorption spectra of CDI **4b** in $CHCl_3$ and MeOH (10 μM concentration).

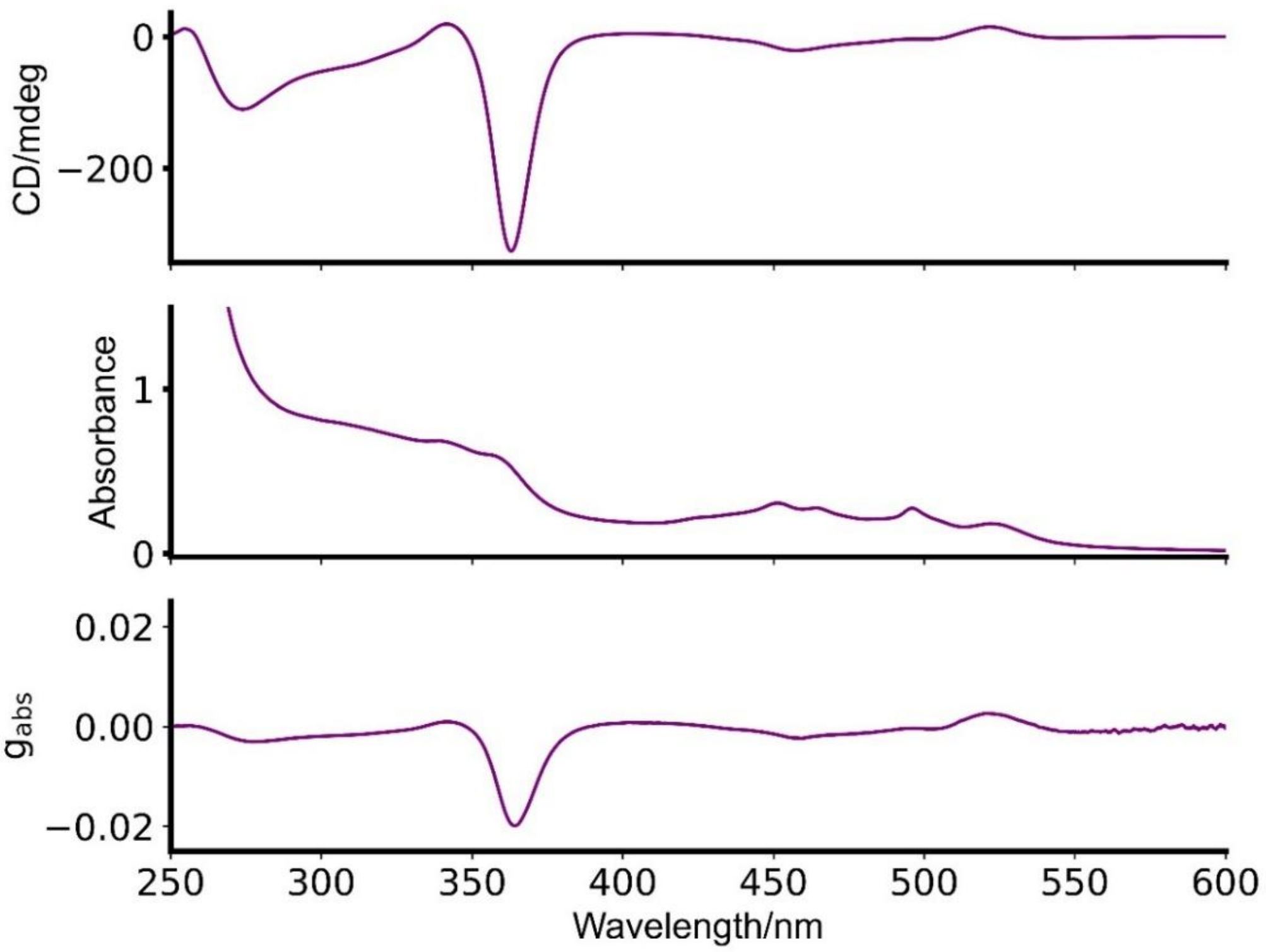

Supplementary Figure 2-4: CD, absorbance and g_{abs} of CDI **4a** in Cyclohexane (10 μM).

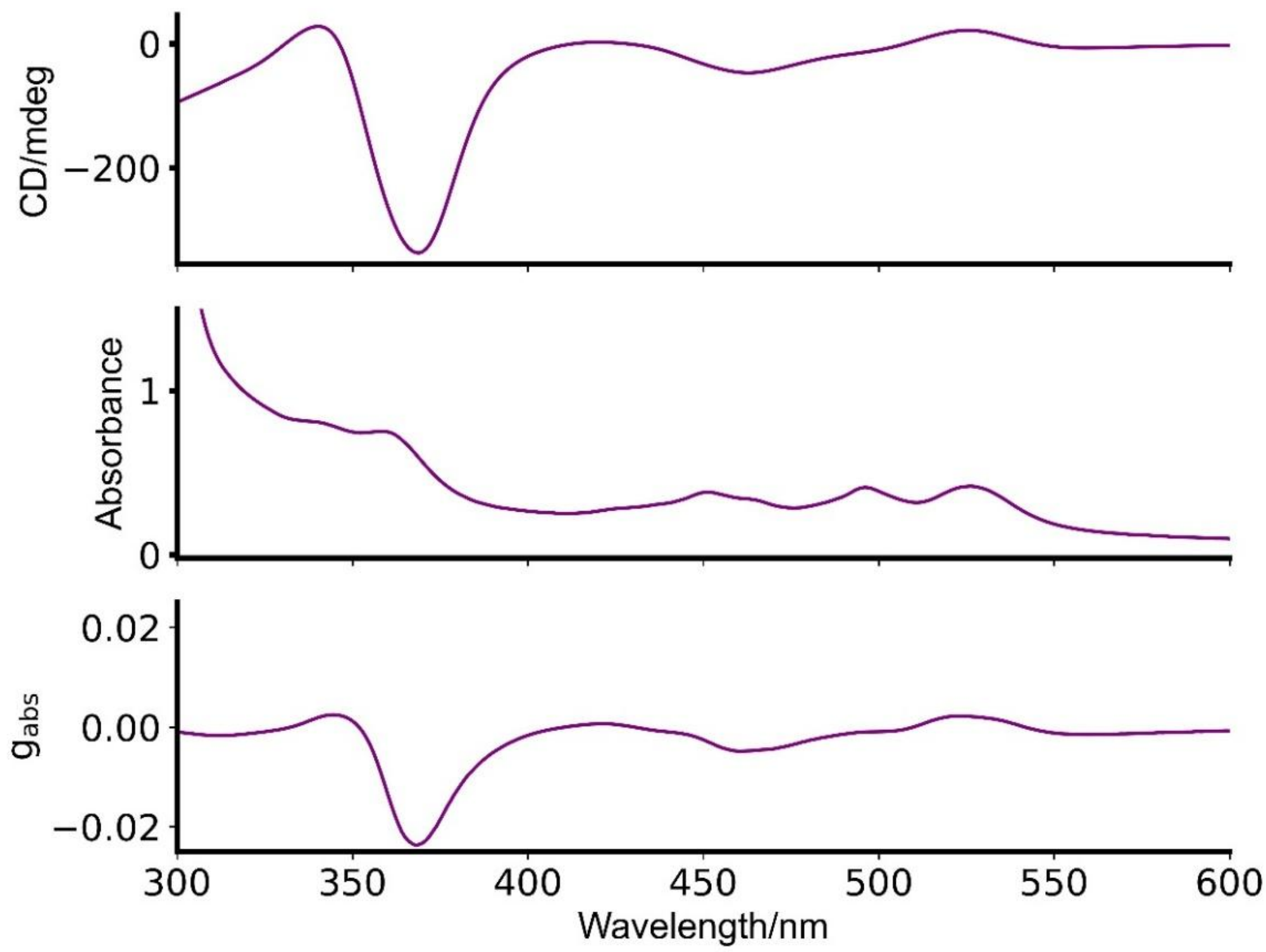

Supplementary Figure 2-5: CD, Absorbance and g_{abs} of CDI **4a** in NEt_3 (20 µM).

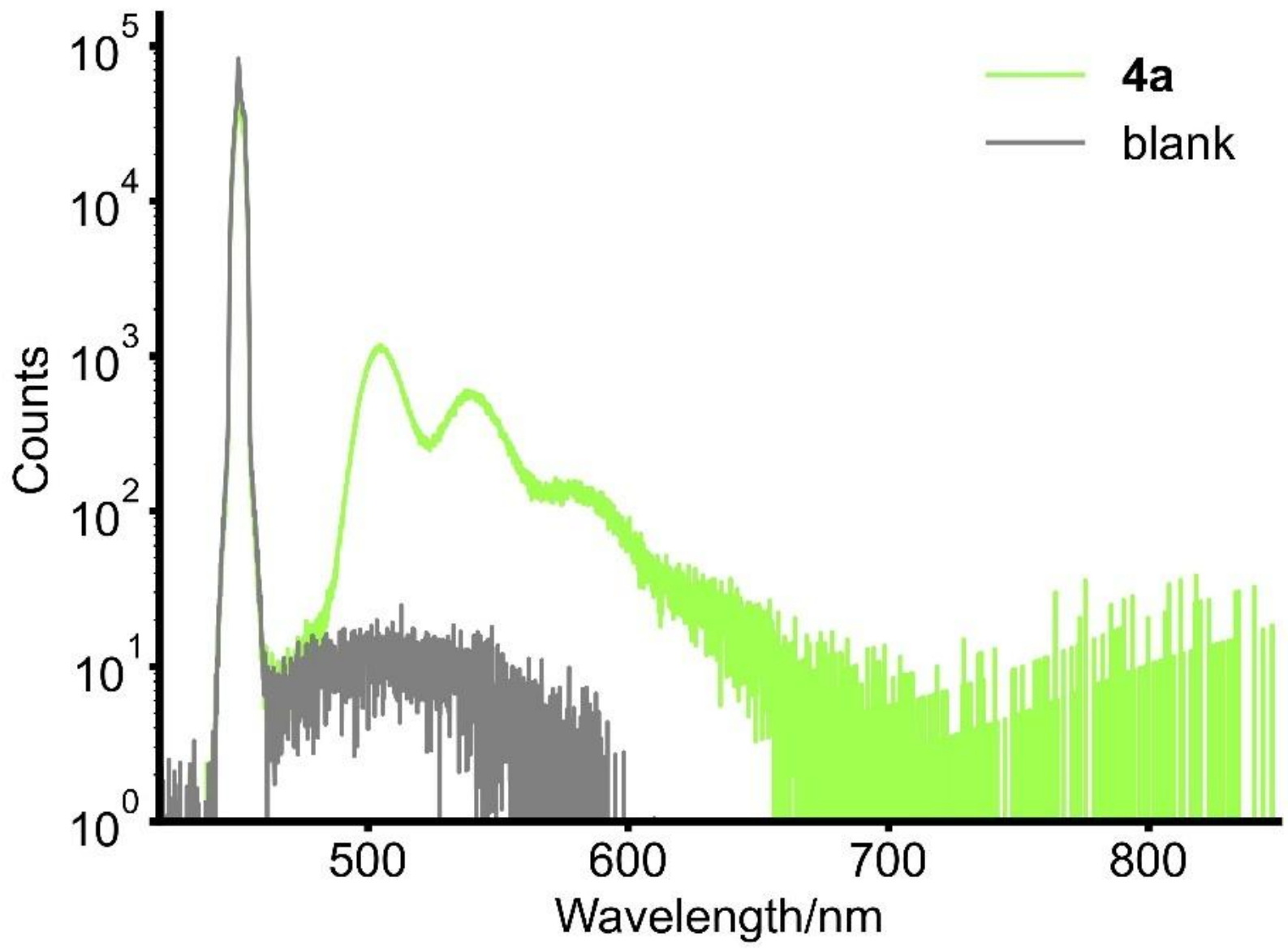

Supplementary Figure 2-6: Quantum Yield of CDI **4a** in $CHCl_3$ – λ_{ex} = 450 nm. Slit widths: λ_{ex} = 4 nm, λ_{em} = 0.8 nm. Φ_{obs} = 91%.

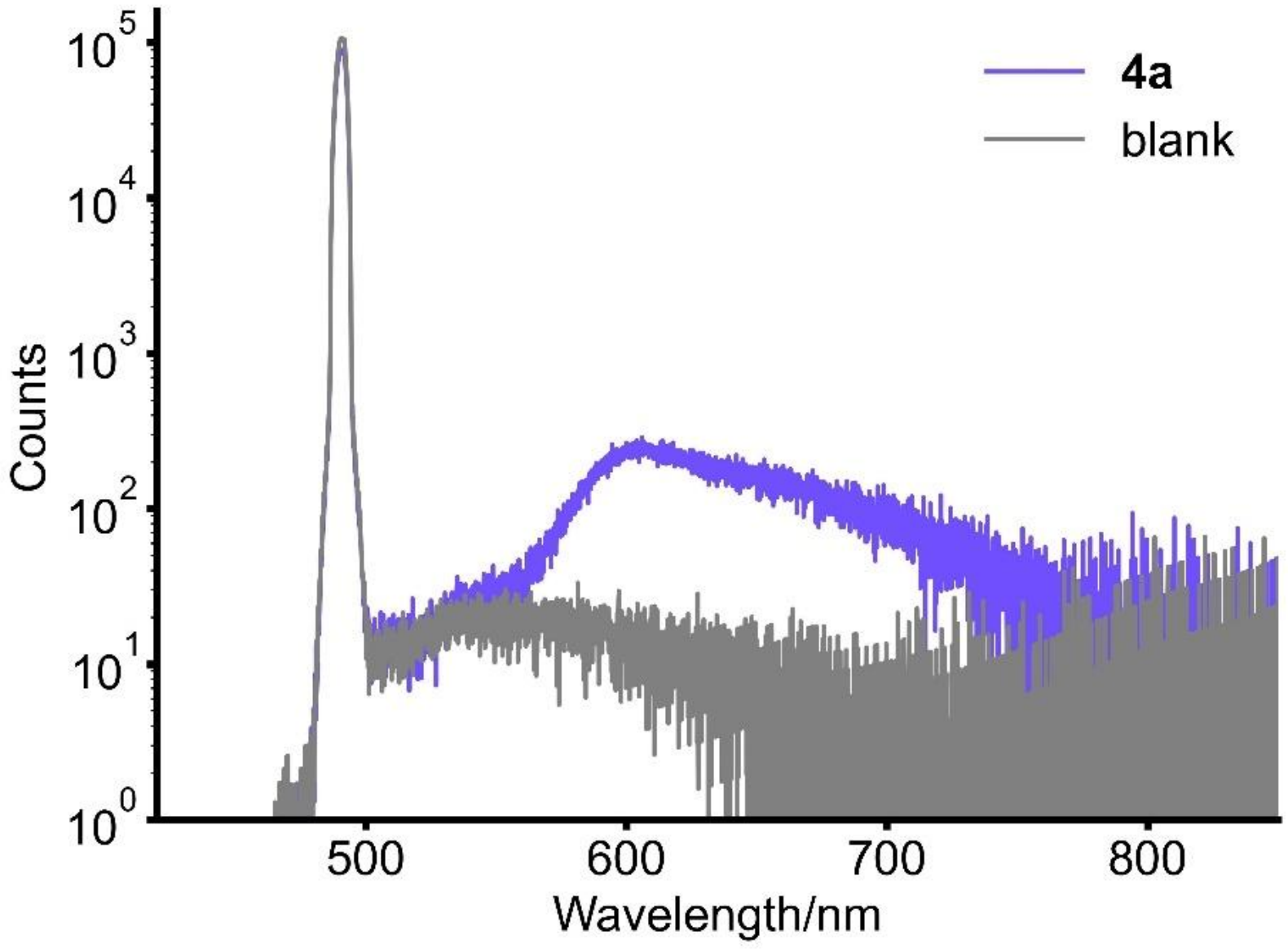

Supplementary Figure 2-7: Quantum Yield of CDI **4a** in MeOH – λ_{ex} = 490 nm. Slit widths: λ_{ex} = 4 nm, λ_{em} = 0.9 nm. Φ_{obs} = 28%.

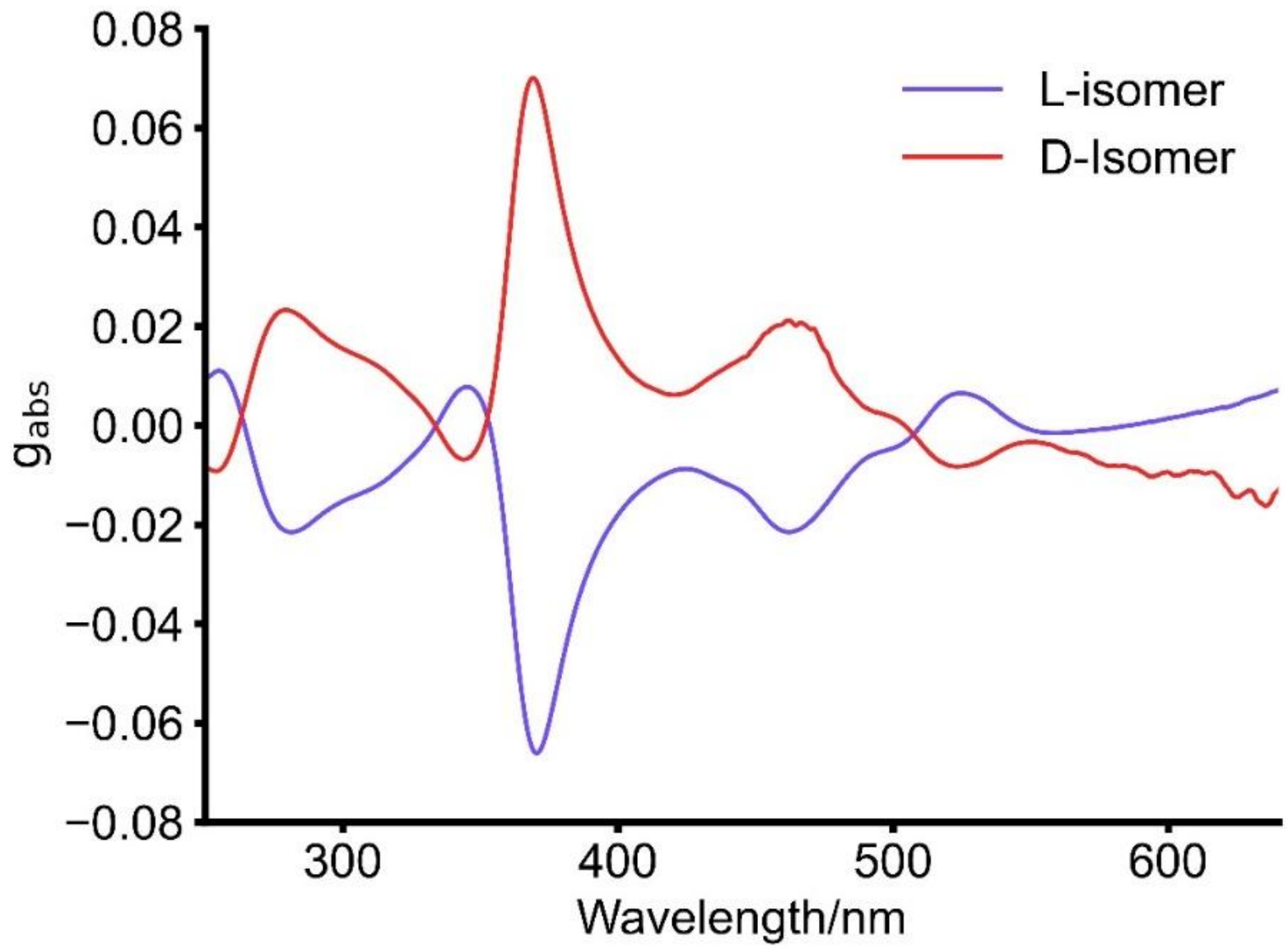

Supplementary Figure 2-8: Calculated g_{abs} for both L- and D-isomer of the CDI **4ab** in MeOH, with the maximum value being ±0.07 (λ = 371 nm).

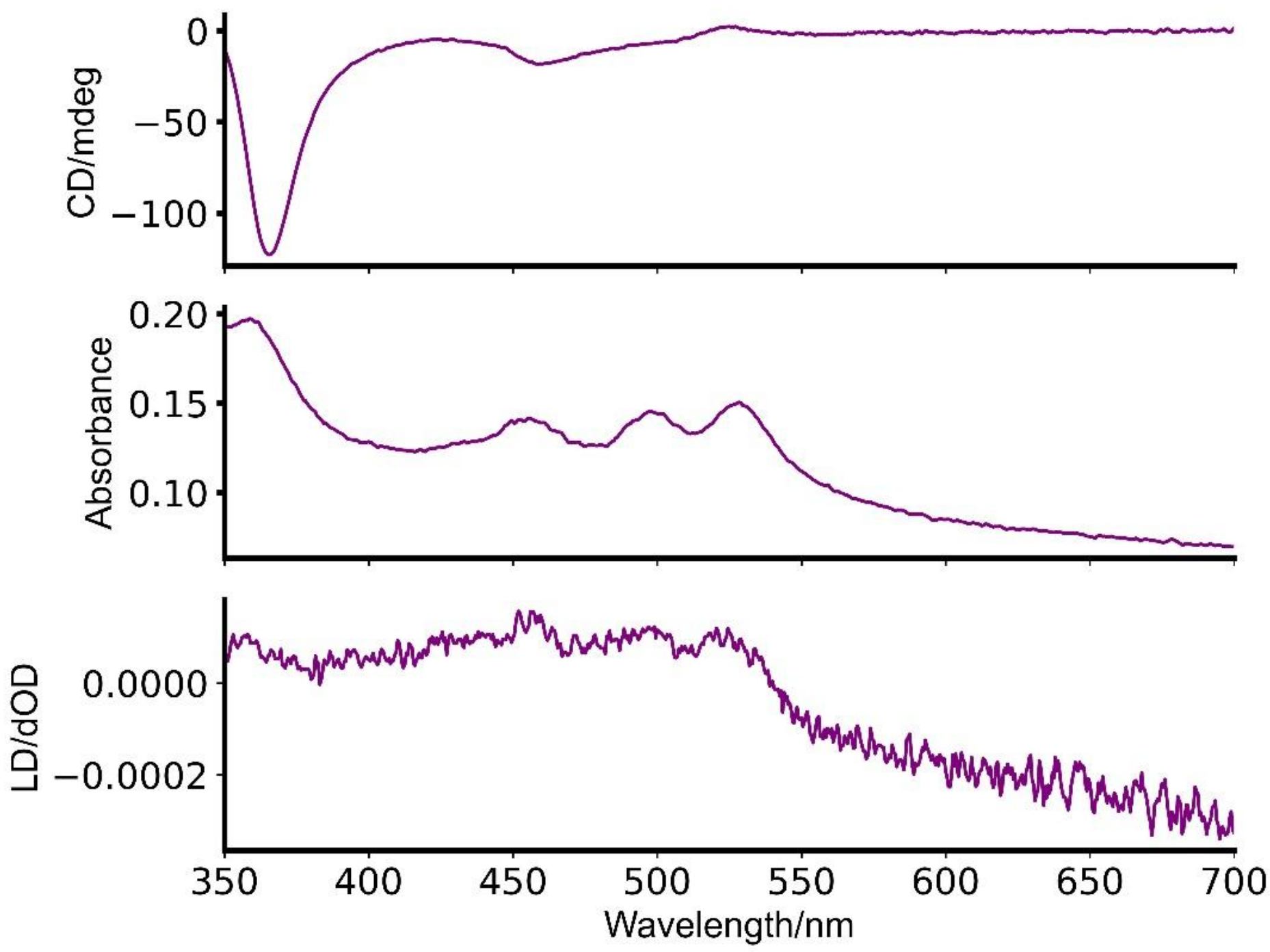

Supplementary Figure 2-9: Measured LD for L-Val-derived CDI **4a** in 5% $CHCl_3$ in MeOH (5 μM) on a JASCO J-1500 benchtop CD spectrophotometer. The measured LD is negligible compared to the intensity of the observed CD signal.

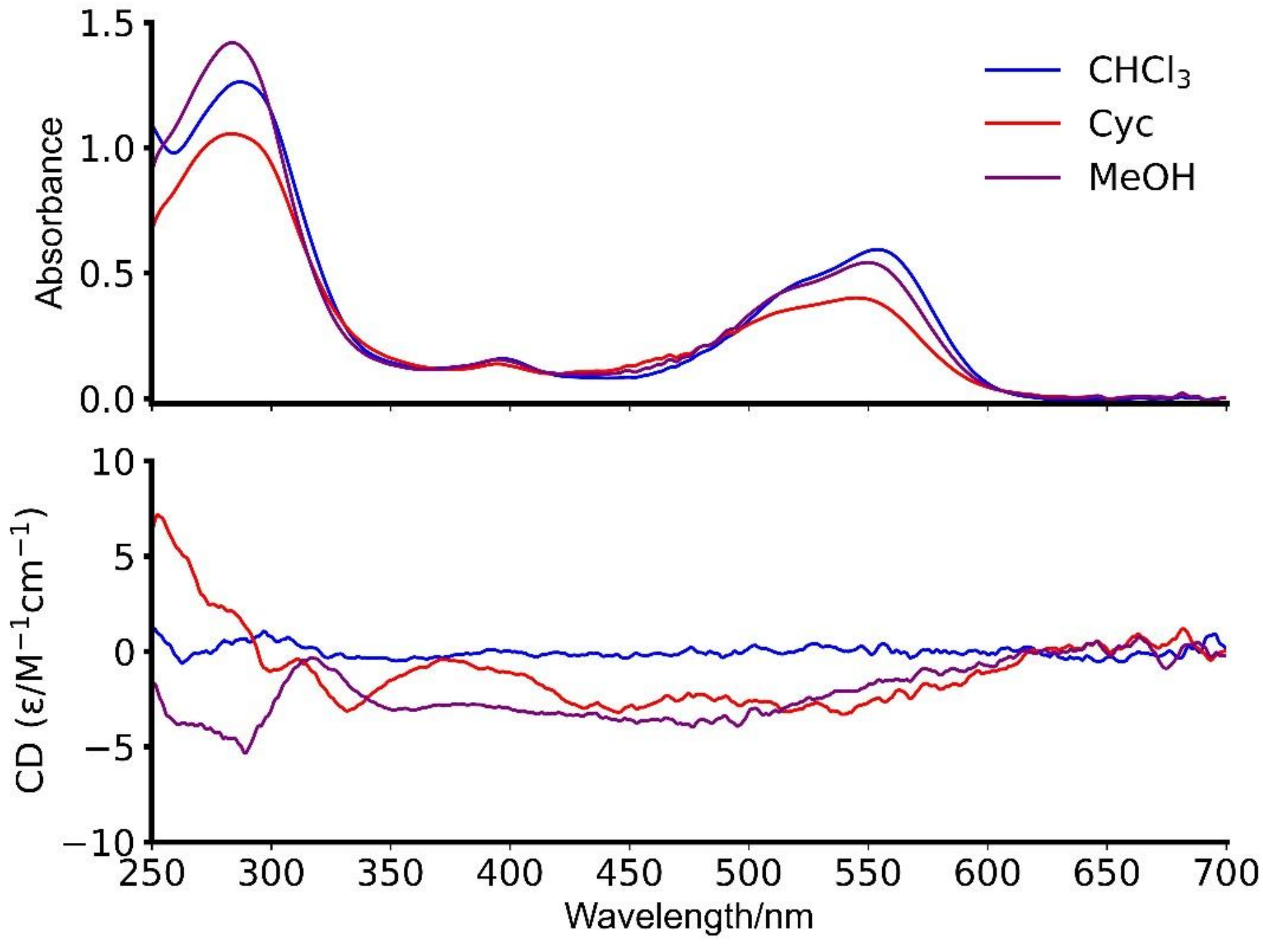

Supplementary Figure 2-10: Absorbance and CD spectra of **2a** at 20 μM concentration in different solvents. The molecule shows no appreciable aggregation or circular dichroism, even in generally considered 'poor' solvents for PDIs,[5] such as cyclohexane (Cyc) or MeOH.

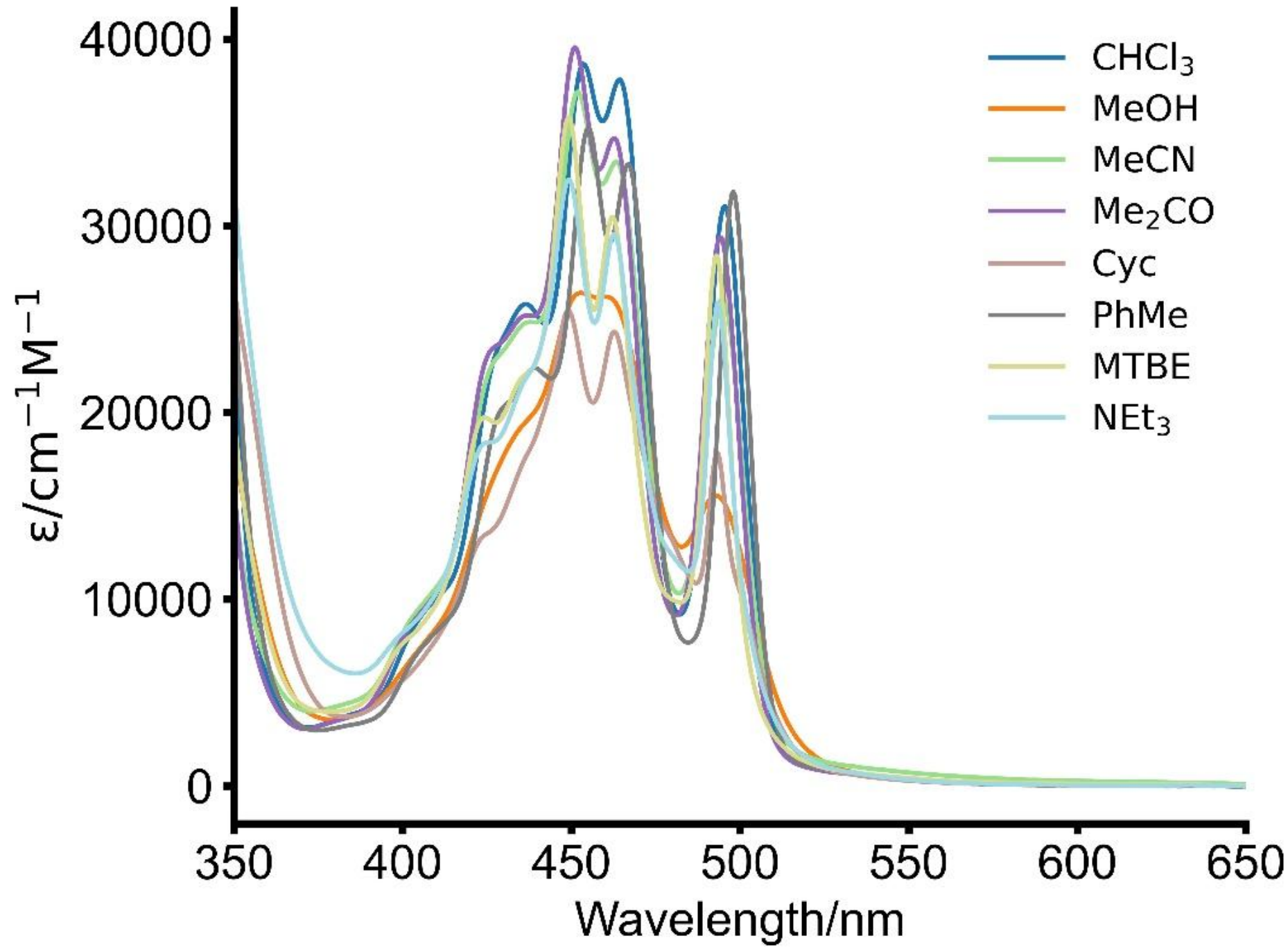

Supplementary Figure 2-11: UV-visible absorption spectra of CDI **3a** in different solvents (20 µM), prepared by addition of 12 µL of a 5 mM $CHCl_3$ stock into 3 mL of the respective solvent, resulting in 0.4% $CHCl_3$ in each sample.

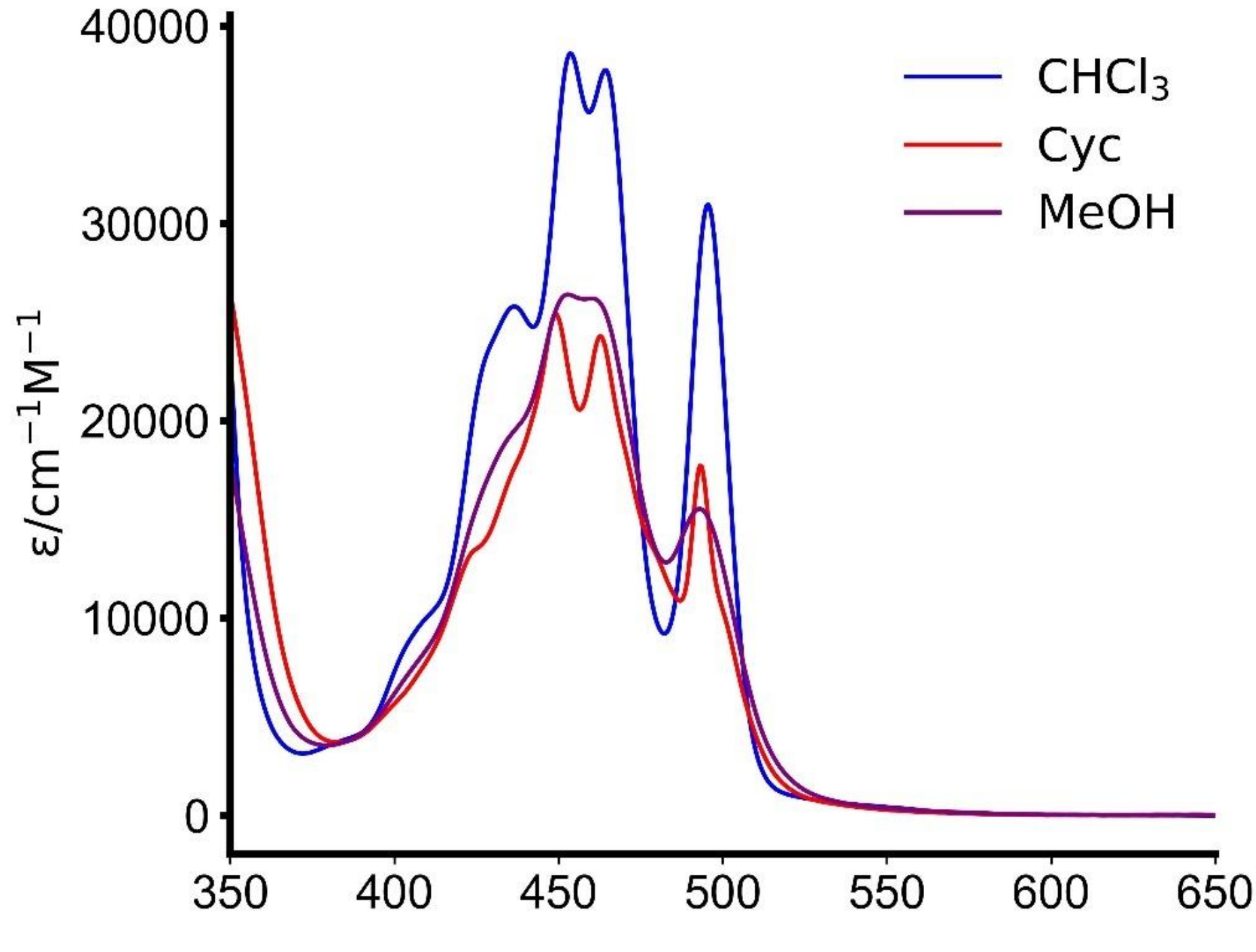

Supplementary Figure 2-12: UV-visible absorption spectra of CDI **3a** from **Supplementary Figure 2-11** in $CHCl_3$ as well as generally considered 'poor' solvents for PDIs,[5] namely cyclohexane (Cyc) and MeOH. No appreciable aggregation is observed.

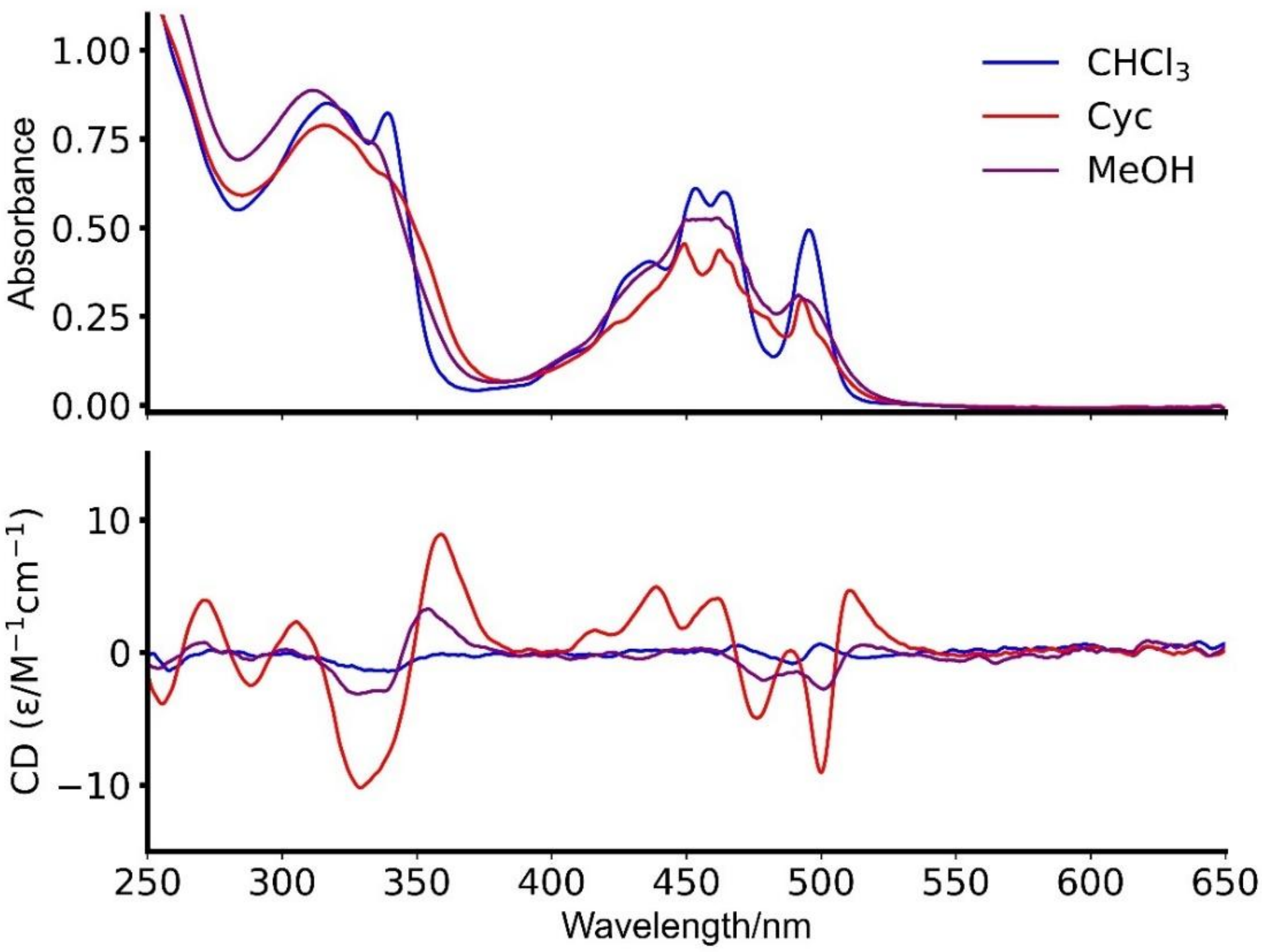

Supplementary Figure 2-13: Absorbance and CD traces of TBS-protected LVal-derived CDI **3a** in different solvents at 20 µM concentration. Only very weak CD is observed, even in generally considered 'poor' solvents for PDIs,[5] such as cyclohexane (Cyc) or MeOH, due to the lack of aggregation.

3) Self-assembly

Solvent-into-solvent titration MeOH/$CHCl_3$

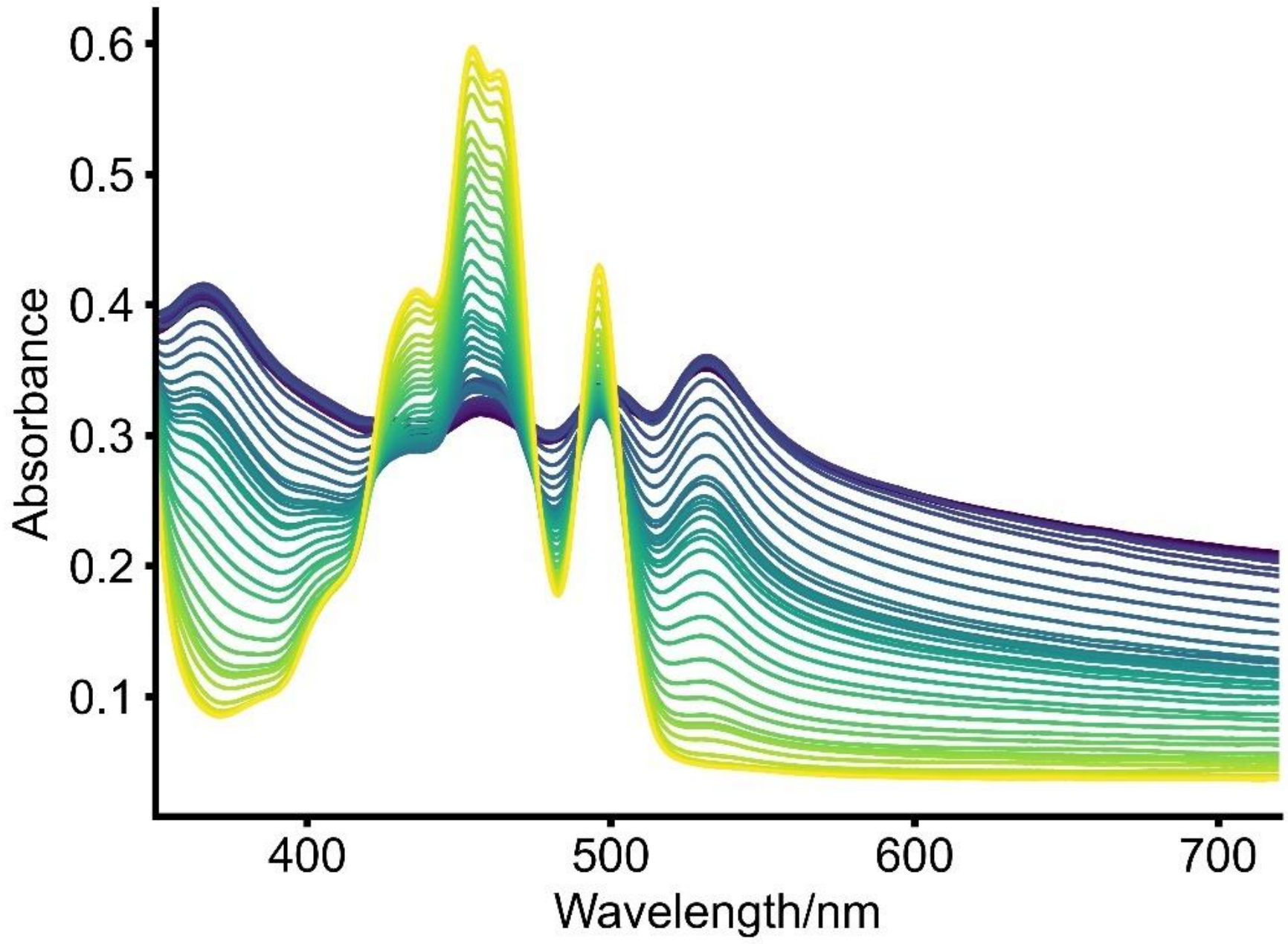

Supplementary Figure 3-1: Changes in absorbance upon addition of a solution of CDI **4a** in $CHCl_3$ (10 μM) to a solution of **4a** in MeOH (10 μM) from 0 (purple) to 50 (yellow) %vol. $CHCl_3$. Solutions were prepared through addition of 6 μL of a 5 mM solution of CDI **4a** in $CHCl_3$ into 3 mL of MeOH. 1.5 mL of this solution were placed into a cuvette and a solution of 10 μM **4a** in $CHCl_3$ was titrated in up to 50% $CHCl_3$ by volume.

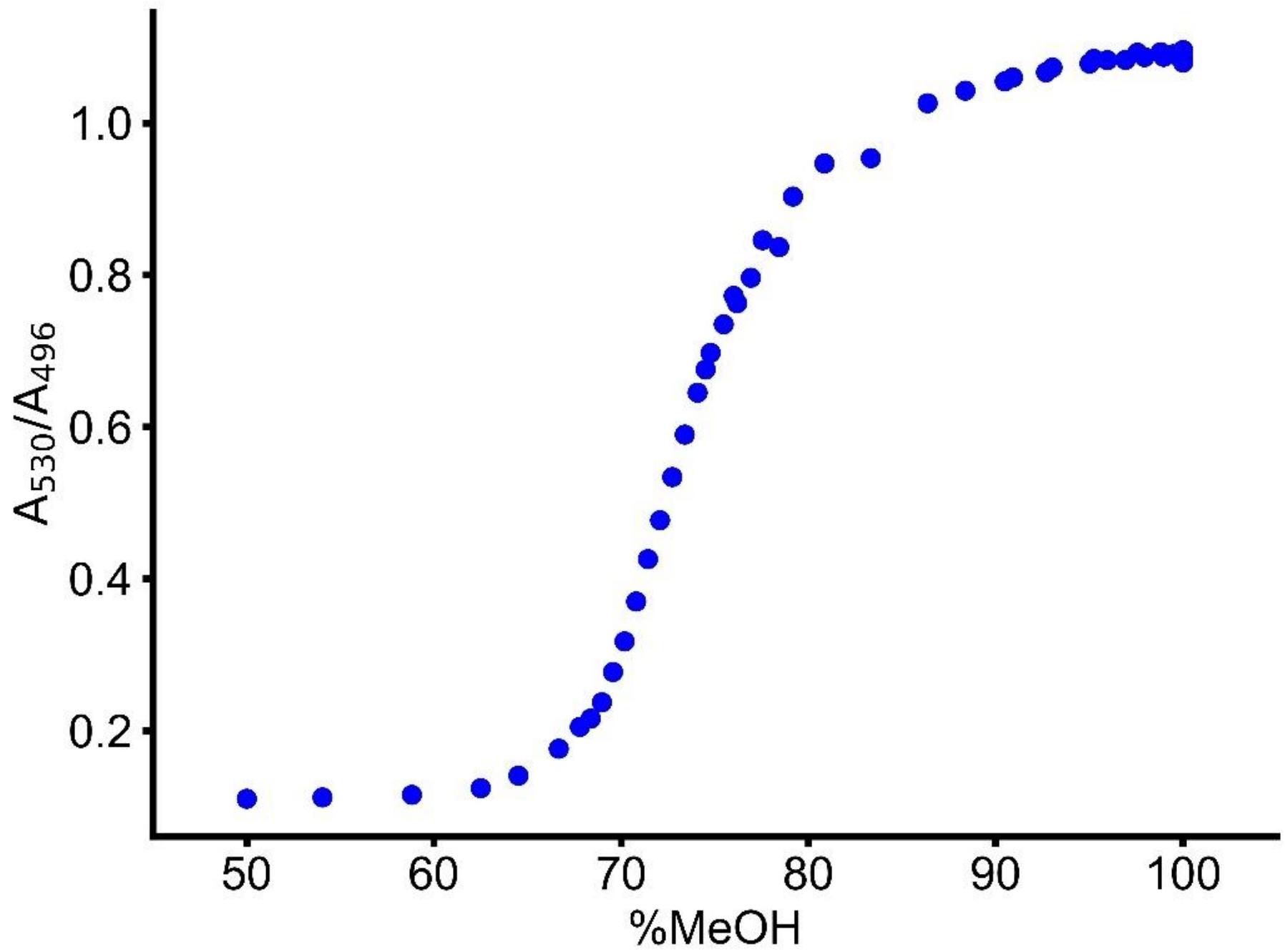

Supplementary Figure 3-2: Ratio of the lowest energy peaks of the aggregate (A_{530}) and monomer (A_{496}) from **Supplementary Figure 3-1** against %MeOH.

Solvent-into-solvent titration $NEt_3/CHCl_3$

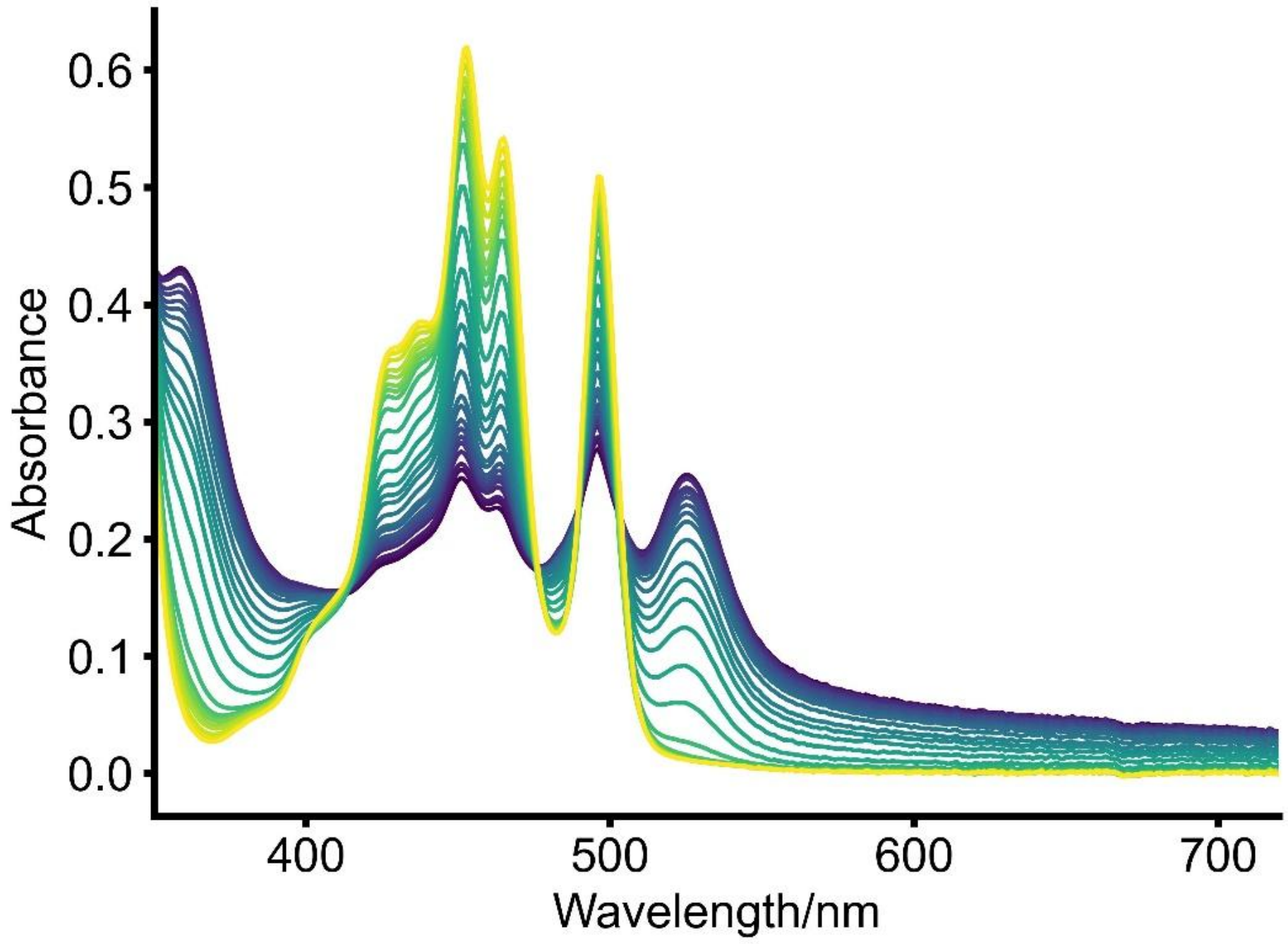

Supplementary Figure 3-3: Changes in absorbance upon addition of a solution of CDI **4a** in $CHCl_3$ (10 μM) to a solution of **4a** in NEt_3 (10 μM) from 0 (purple) to 50 (yellow) %vol. $CHCl_3$. Solutions were prepared through addition of 6 μL of a 5 mM solution of CDI **4a** in $CHCl_3$ into 3 mL of NEt_3. 1.5 mL of this solution were placed into a cuvette and a solution of 10 μM **4a** in $CHCl_3$ was titrated in up to 50% $CHCl_3$ by volume.

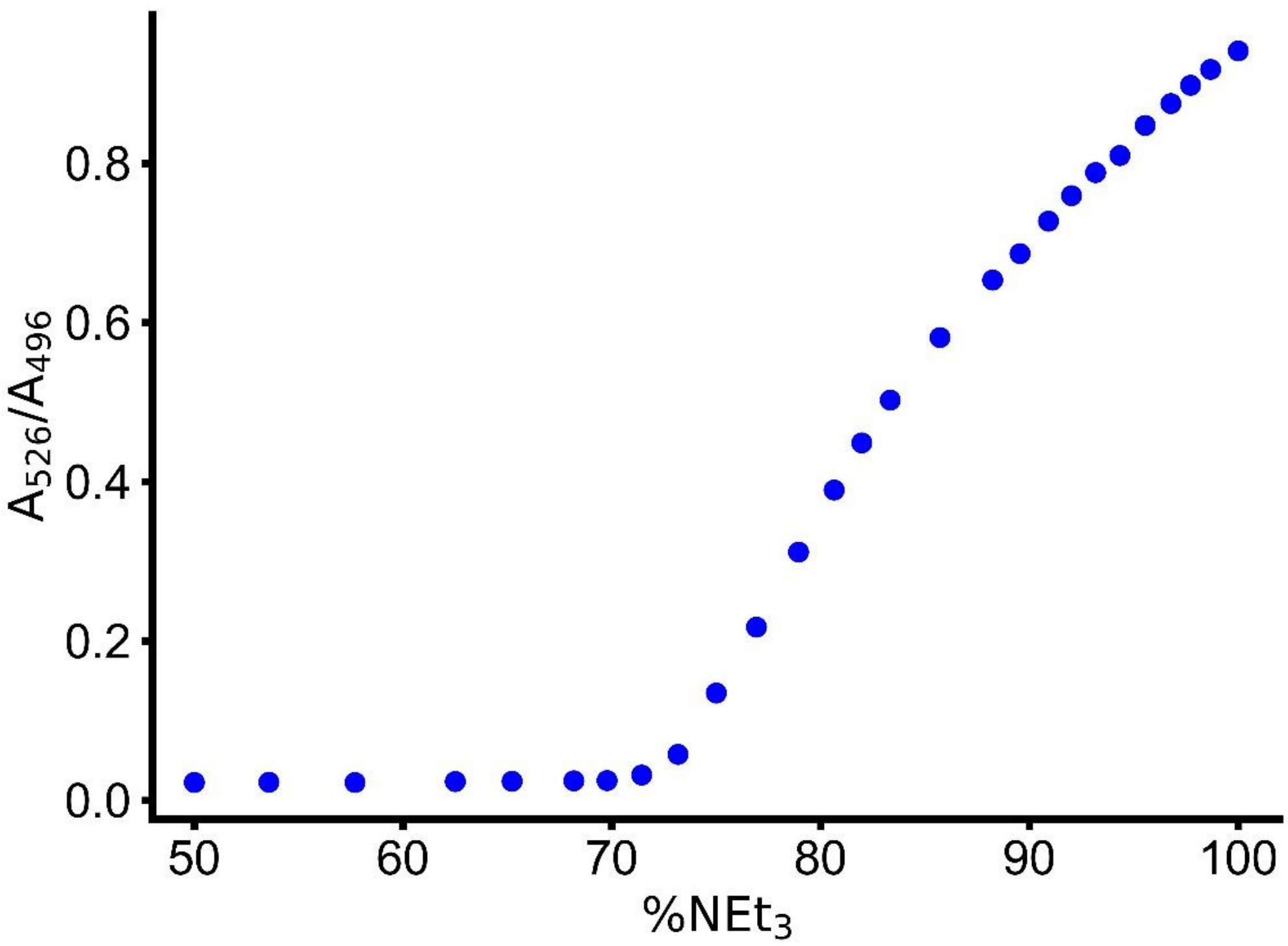

Supplementary Figure 3-4: Ratio of the lowest energy peaks of the aggregate (A_{526}) and monomer (A_{496}) from **Supplementary Figure 3-3** against $\%NEt_3$.

Temperature-dependent self-assembly in 3:1 MeOH:$CHCl_3$

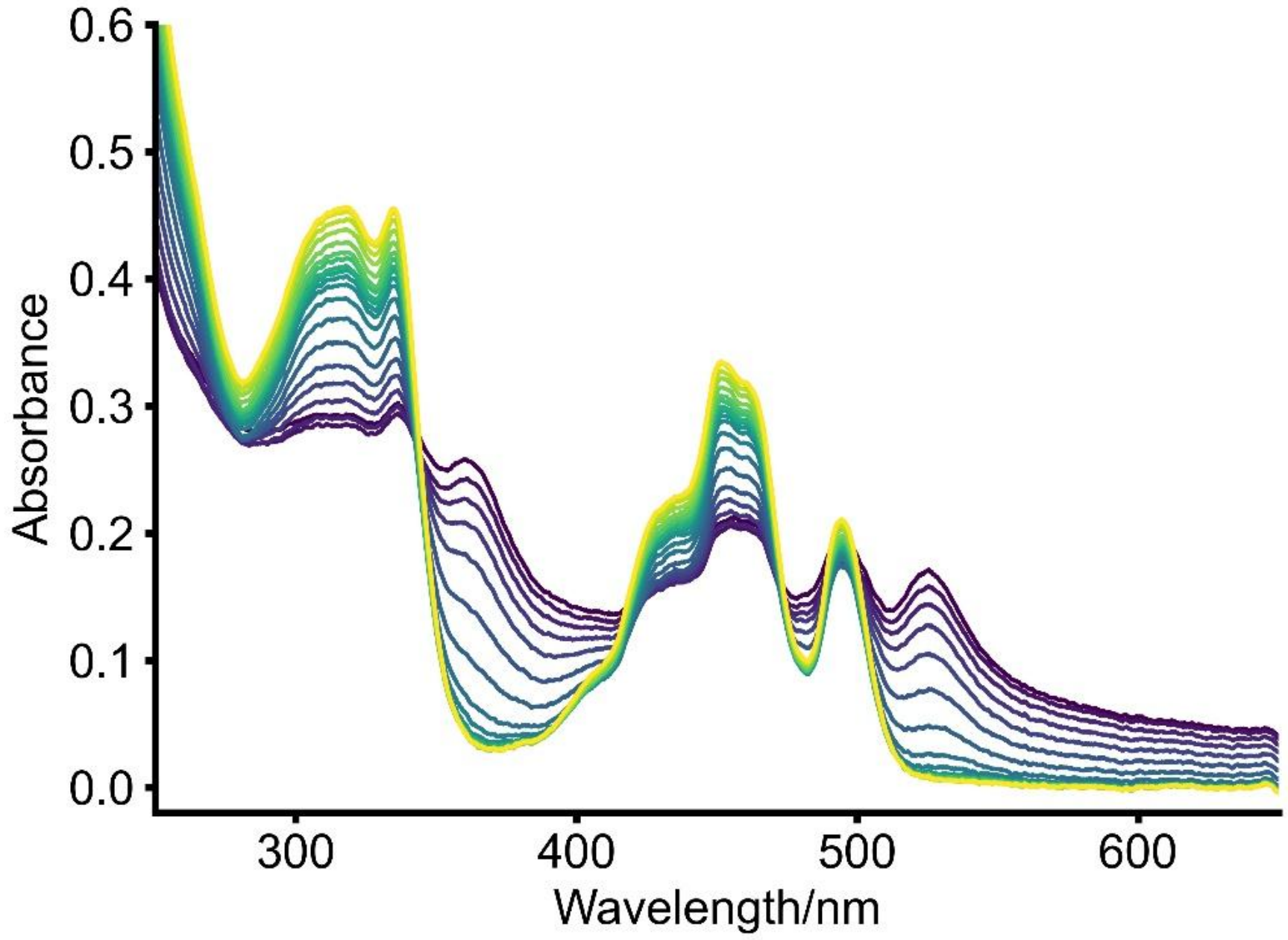

Supplementary Figure 3-5: Changes in absorbance of CDI **4a** in 3:1 MeOH:$CHCl_3$ (10 μM) with varying temperature from 0 °C (purple) to 65 °C (yellow) in 3:1 $CHCl_3$:MeOH (10 μM). Sample was prepared by addition of 6 μL of a 5 mM stock solution of compound **4a** in $CHCl_3$ to 2.25 mL of MeOH, then 0.75 mL $CHCl_3$ was added and the sample cooled to 0 °C for 30 minutes before starting the measurement. At each temperature, the sample was left to equilibrate for 2 minutes before measuring. Measurement was performed on a JASCO J-1500 CD spectrophotometer.

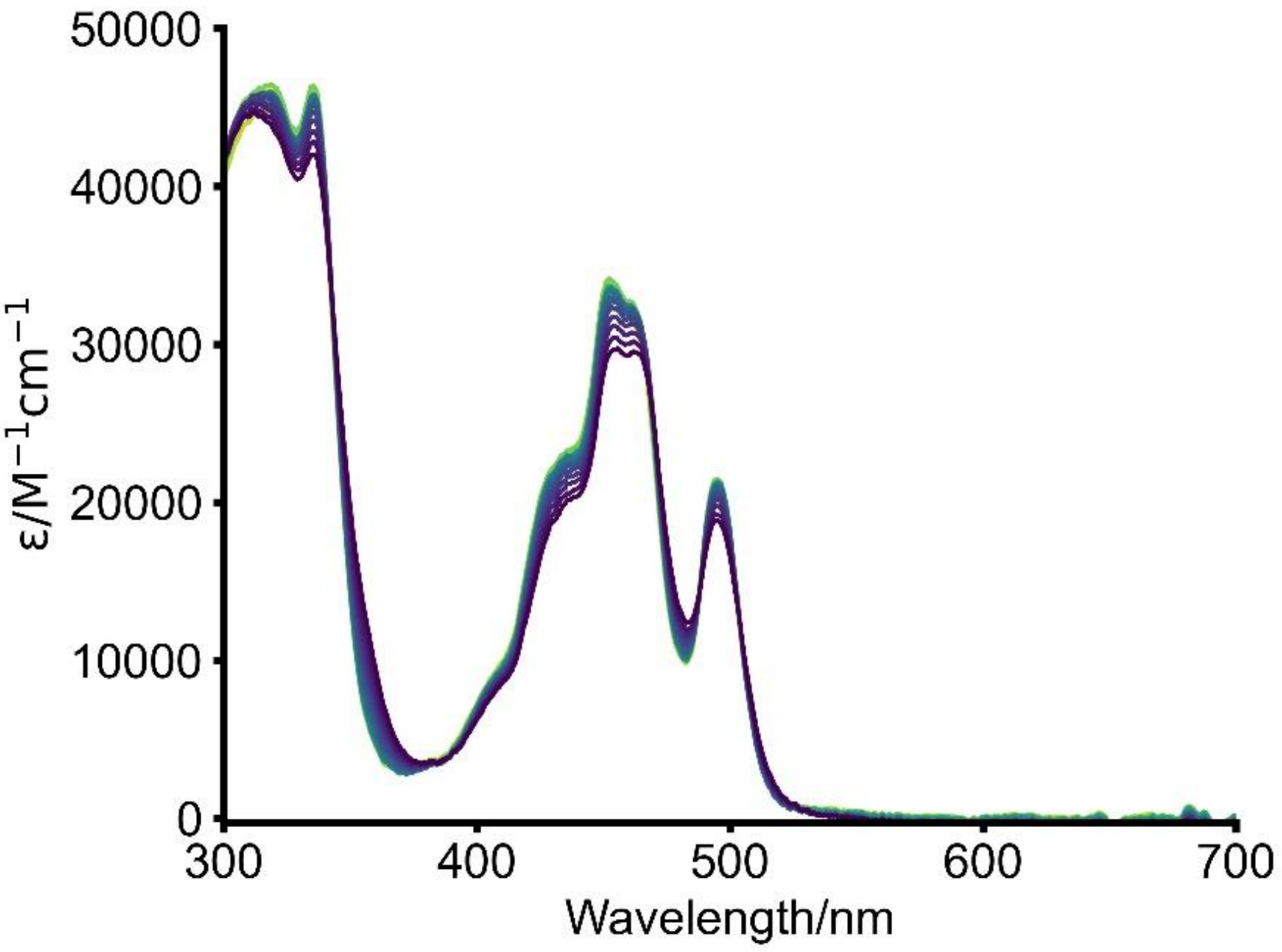

Supplementary Figure 3-6: Changes in absorbance of CDI **4a** in 3:1 MeOH:$CHCl_3$ (10 μM) after heating (**Supplementary Figure 3-5**) upon decreasing the temperature from 65 °C (yellow) to 0 °C (purple) in 2 °C increments to 45 °C, followed by 5 °C increments. At each temperature, the sample was left to equilibrate for 2 minutes before measuring. Measurement was performed on a JASCO J-1500 CD spectrophotometer.

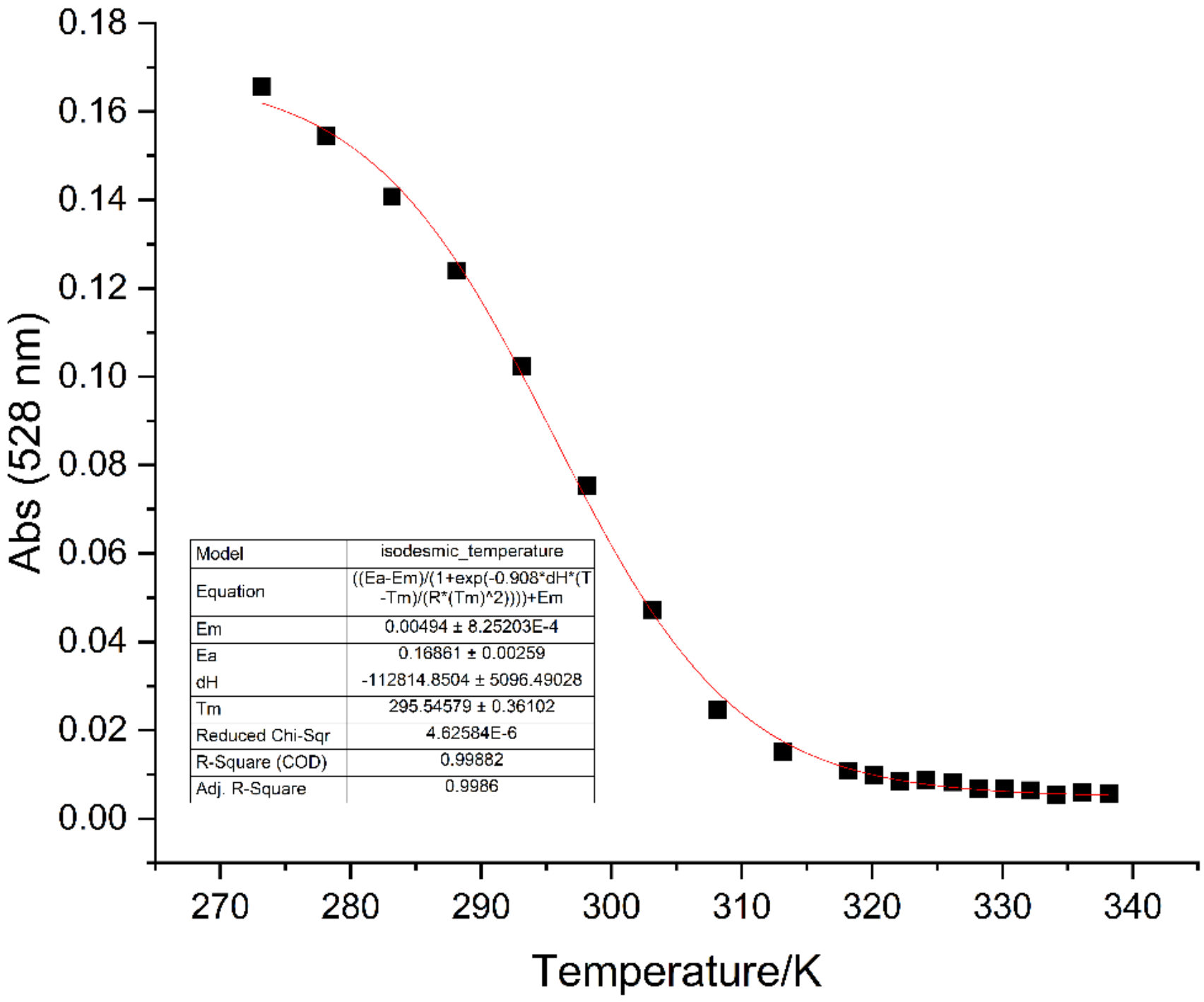

Supplementary Figure 3-7: Fitting of the absorbance at 528 nm from **Supplementary Figure 3-5** to a temperature-dependent isodesmic aggregation model.[6]

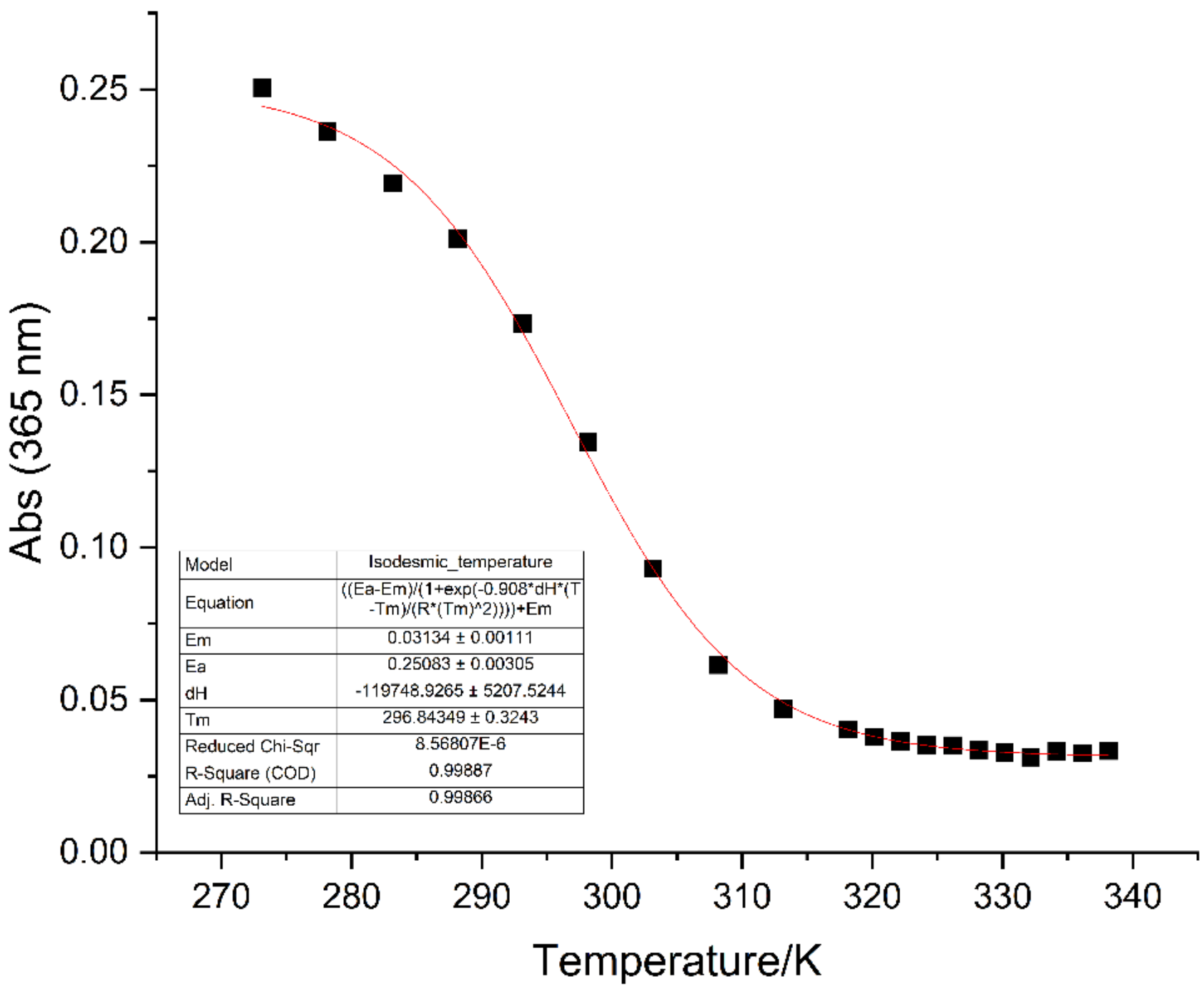

Supplementary Figure 3-8: Fitting of the absorbance at 365 nm from **Supplementary Figure 3-5** to a temperature-dependent isodesmic aggregation model.[6]

Supplementary Table 3-1: Thermodynamic parameters computed from the isodesmic fit to the absorbance data (**Supplementary Figures 3-7** and **3-8**). Data reported was calculated for 298 K.

**Index**	**Wavelength (nm)**	**ΔH (kJ mol^{-1})**	**ΔG (kJ mol^{-1})**	**K_e (x10^5 M^{-1})**	**ΔS (J K^{-1} mol^{-1})**	**T_m (K)**	**DP_N**
**1**	528	–112.81	-31.33	3.08	–296.1	295.5	1.32
**2**	365	–119.75	-31.47	3.26	–273.3	296.8	1.37
**3**	Average	–116.28	-31.40	3.17	–284.7	296.2	1.35

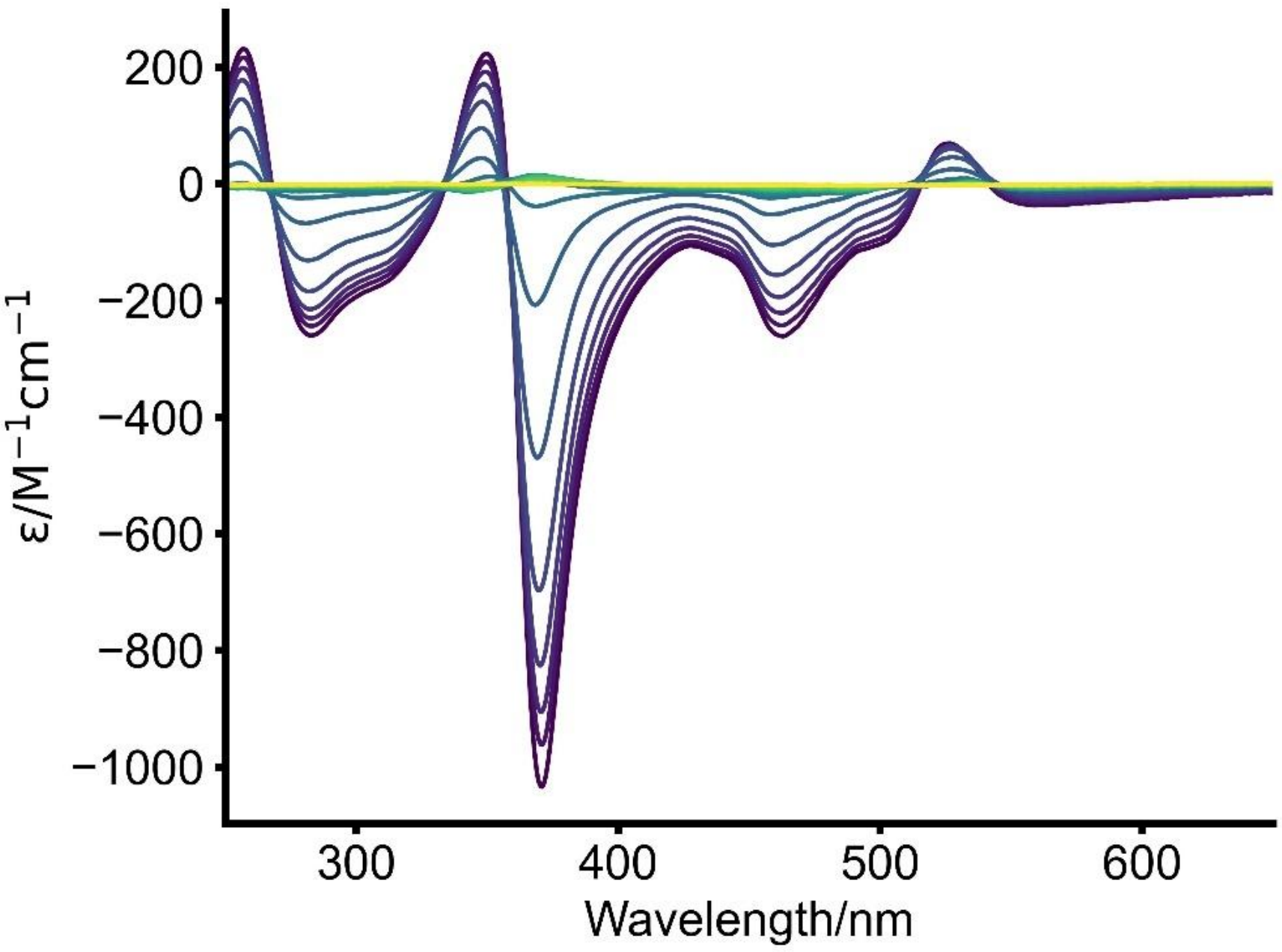

Supplementary Figure 3-9: Changes in circular dichroism of CDI **4a** in 3:1 MeOH:$CHCl_3$ (10 μM) (**Supplementary Figure 3-5**) upon increasing the temperature from 0 °C (purple) to 65 °C (yellow) in 5 °C increments (from 0 to 45 °C), followed by 2 °C increments. Sample was prepared by addition of 6 μL of a 5 mM stock solution of compound **4a** in $CHCl_3$ to 2.25 mL of MeOH, then 0.75 mL $CHCl_3$ was added and the sample cooled to 0 °C for 30 minutes before starting the measurement. At each temperature, the sample was left to equilibrate for 2 minutes before measuring. Measurement was performed on a JASCO J-1500 CD spectrophotometer.

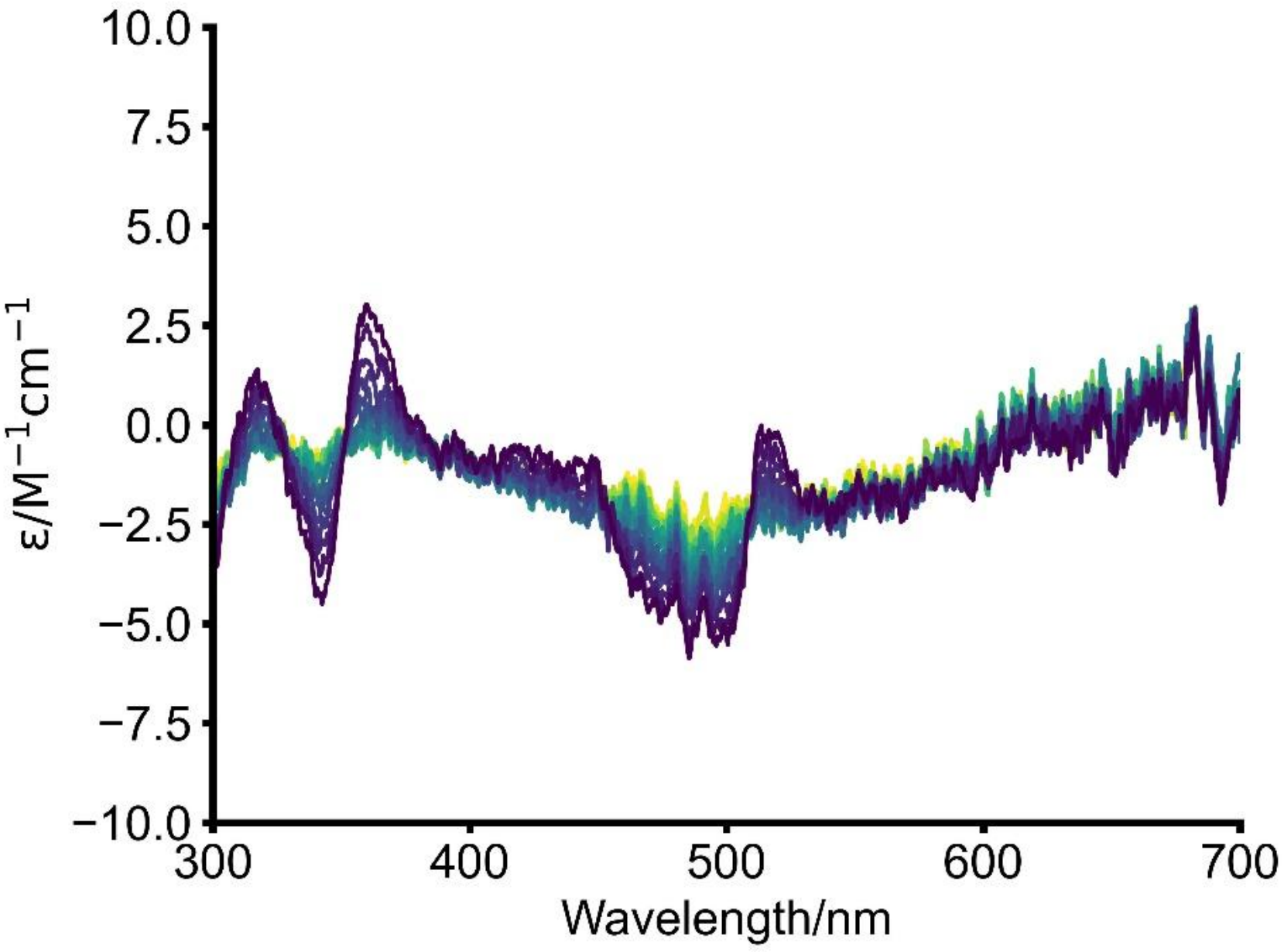

Supplementary Figure 3-10: Changes in circular dichroism of CDI **4a** in 3:1 MeOH:$CHCl_3$ (10 μM) after heating (**Supplementary Figure 3-9**) upon decreasing the temperature from 65 °C (yellow) to 0 °C (purple) in 2 °C increments to 45 °C, followed by 5 °C increments. At each temperature, the sample was left to equilibrate for 2 minutes before measuring. Measurement was performed on a JASCO J-1500 CD spectrophotometer.

Temperature-dependent self-assembly in 9:1 NEt_3:$CHCl_3$

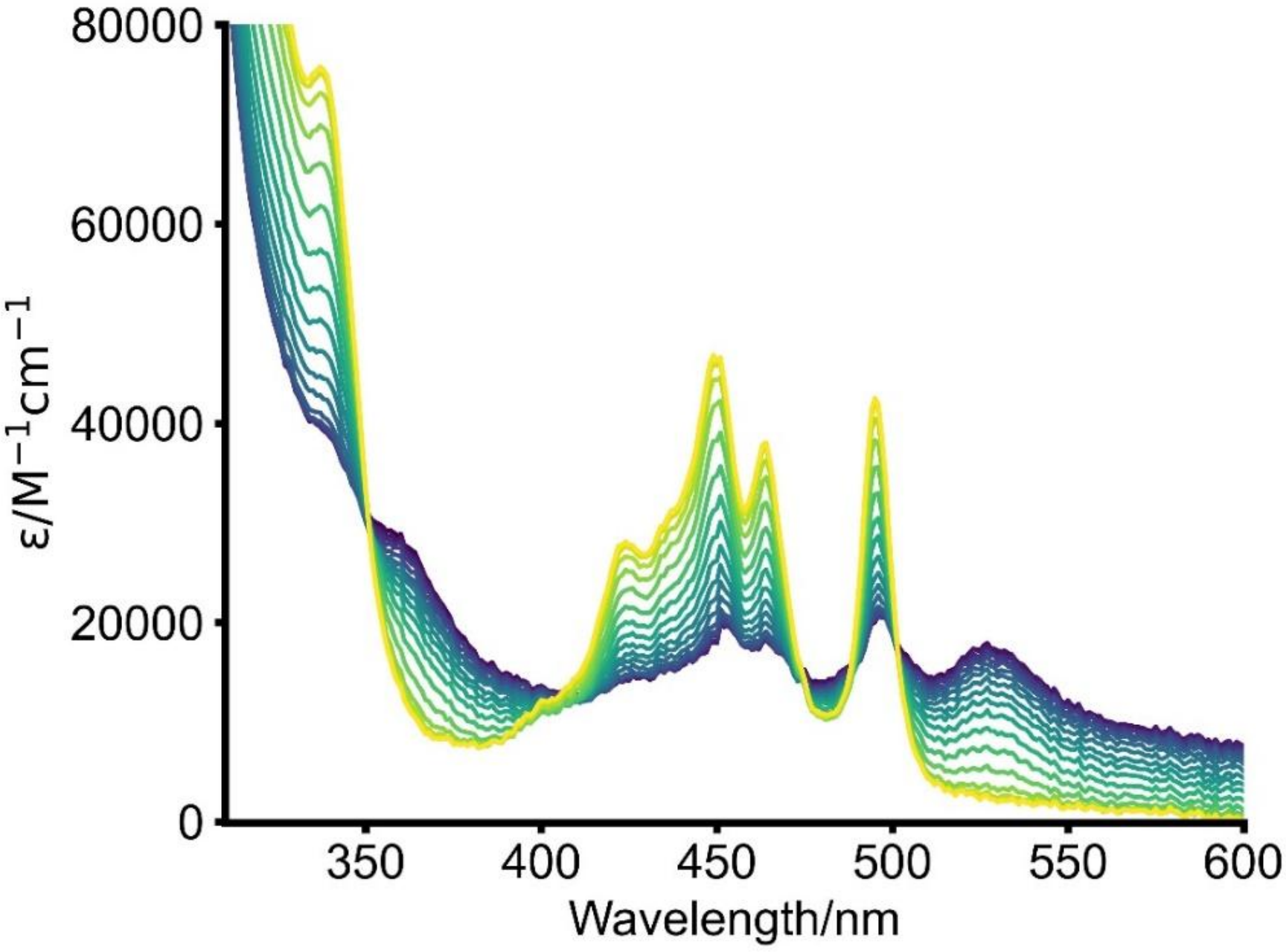

Supplementary Figure 3-11: Changes in absorbance of CDI **4a** in 9:1 NEt_3:$CHCl_3$ (10 μM) with varying temperature from 0 °C (purple) to 85 °C (yellow) in 5°C increments. Sample was prepared by addition of 6 μL of a 5 mM stock solution of compound **4a** in $CHCl_3$ to 2.7 mL of cold NEt_3, then 0.294 mL $CHCl_3$ was added and the sample cooled to 0 °C for 15 minutes before starting the measurement. At each temperature, the sample was left to equilibrate for 1 minute before measuring. Measurement was performed on a Chirascan V100 CD spectrophotometer.

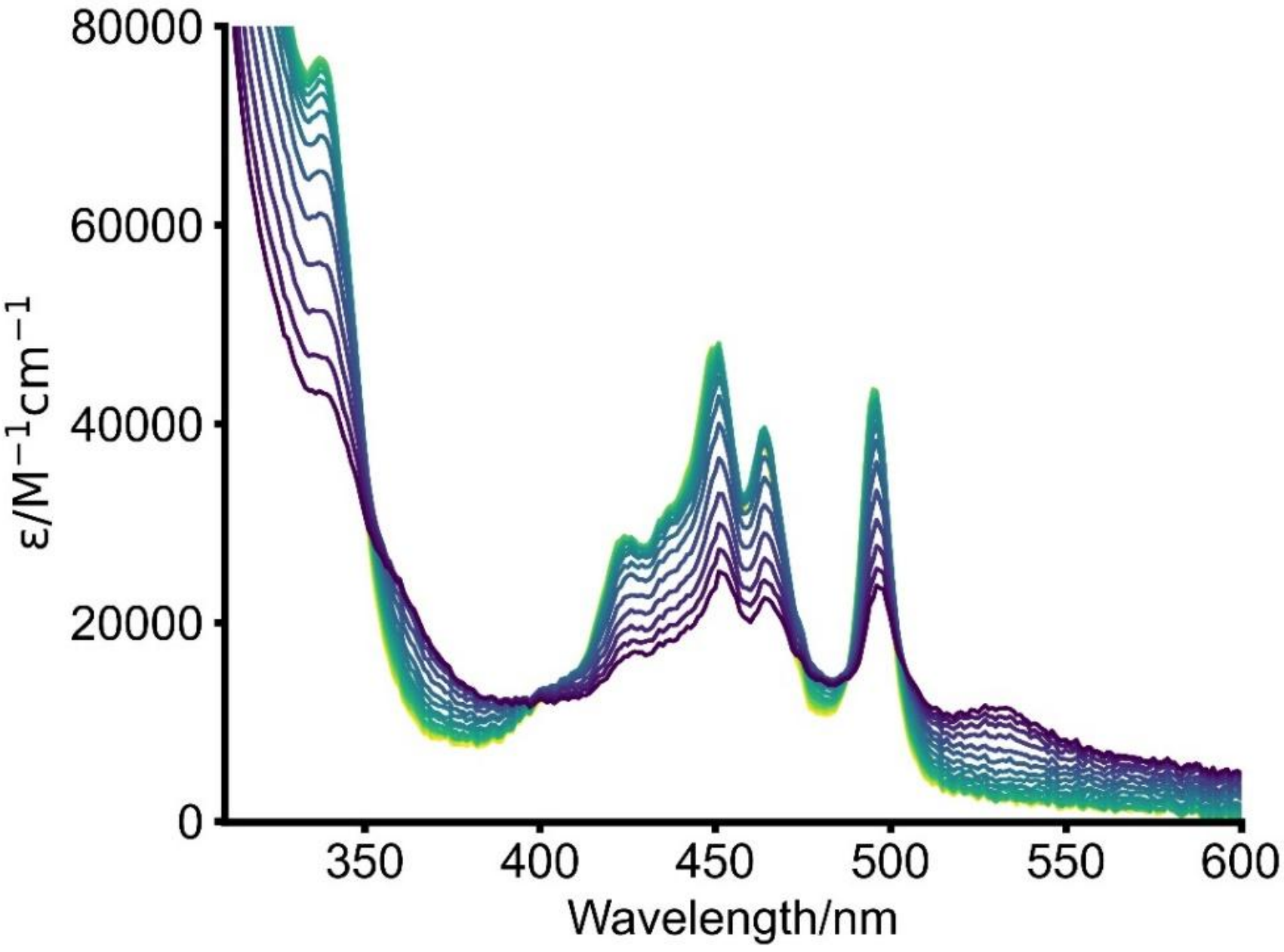

Supplementary Figure 3-12: Changes in absorbance of CDI **4a** in 9:1 NEt_3:$CHCl_3$ (10 μM) after heating (**Supplementary Figure 3-11**) upon decreasing the temperature from 85 °C (yellow) to 0 °C (purple) in 5 °C increments Sample was prepared by addition of 6 μL of a 5 mM stock solution of compound **4a** in $CHCl_3$ to 2.7 mL of cold NEt_3, then 0.294 mL$CHCl_3$ was added and the sample cooled to 0 °C for 20 minutes before starting the measurement. At each temperature, the sample was left to equilibrate for 1 minute before measuring. Measurement was performed on a Chirascan V100 CD spectrophotometer.

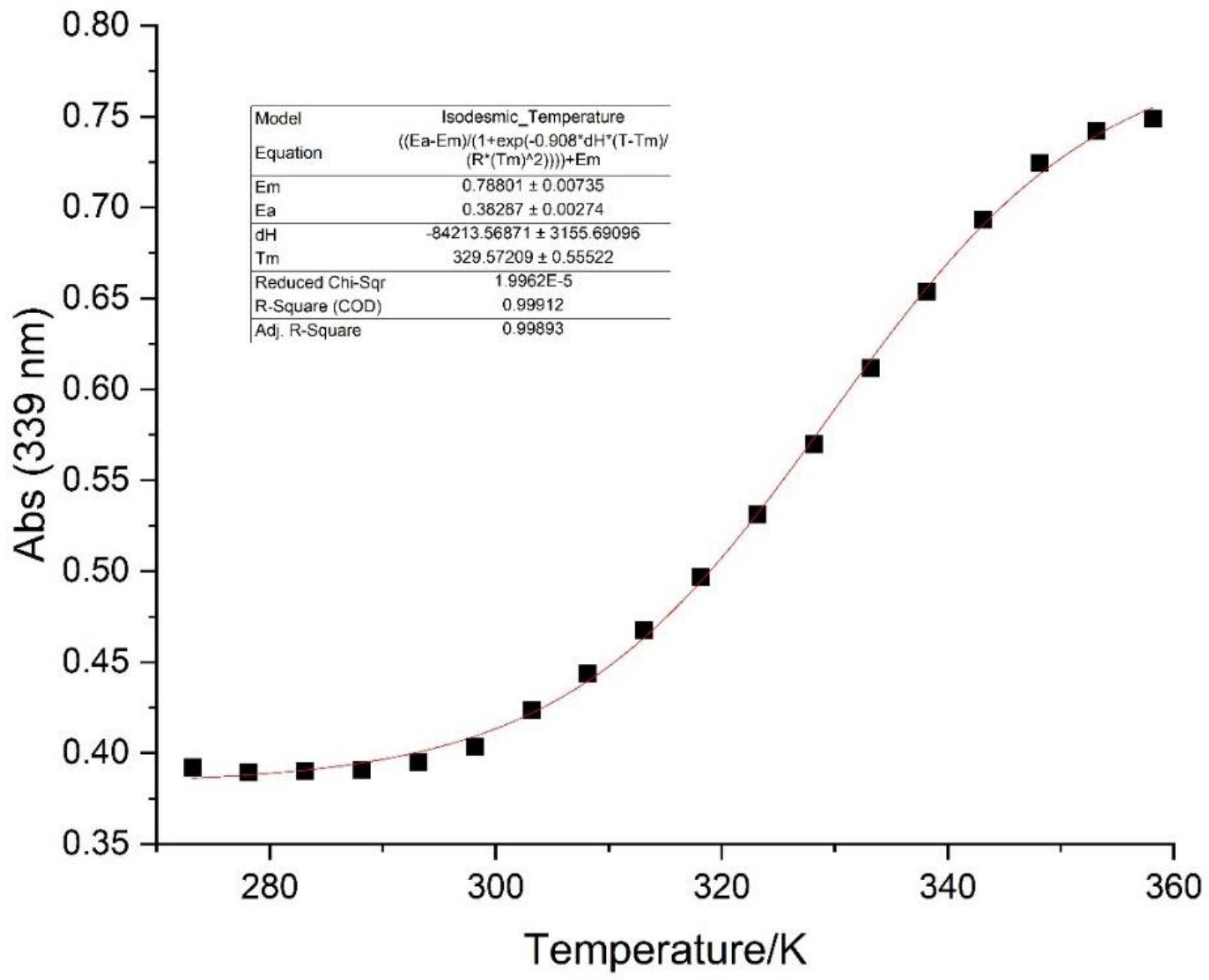

Supplementary Figure 3-13: Fitting of the absorbance at 339 nm from **Supplementary Figure 3-11** to a temperature-dependent isodesmic aggregation model.[6]

Supplementary Table 3-2: Thermodynamic parameters computed from the isodesmic fit to the absorbance data (**Supplementary Figure 3-13**). Data reported was calculated for 298 K.

**Index**	**Wavelength (nm)**	**ΔH (kJ mol^{-1})**	**ΔG (kJ mol^{-1})**	**K_e (x10^5 M^{-1})**	**ΔS (J K^{-1} mol^{-1})**	**T_m (K)**	**DP_N**
**1**	339	-84.21	-36.40	23.82	-160.36	329.57	4.41

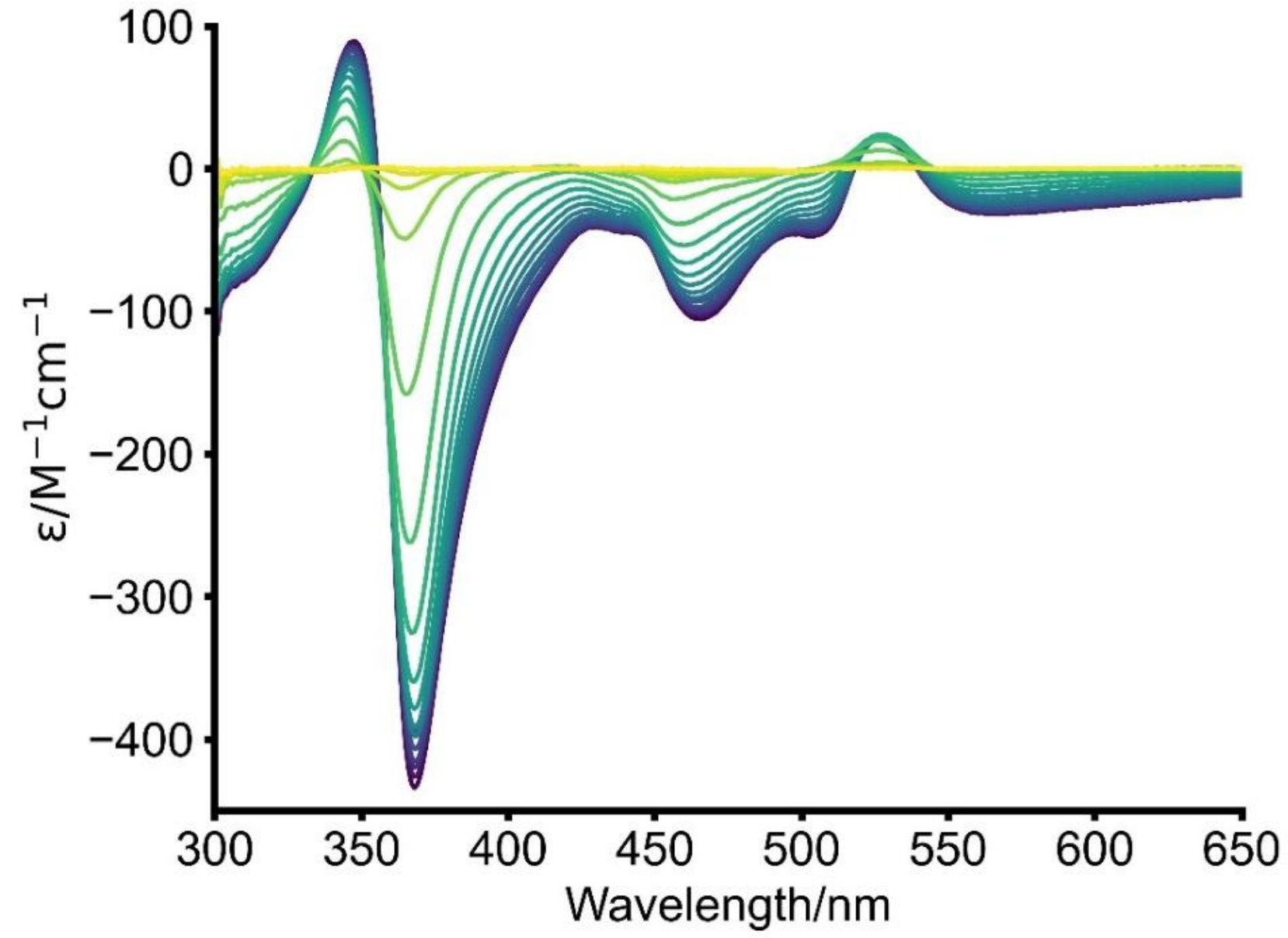

Supplementary Figure 3-14: Changes in circular dichroism of CDI **4a** in 9:1 NEt_3:$CHCl_3$ (10 μM) (**Supplementary Figure 3-11**) upon increasing the temperature from 0 °C (purple) to 85 °C (yellow) in 5 °C increments. Sample was prepared by addition of 6 μL of a 5 mM stock solution of compound **4a** in $CHCl_3$ to 2.7 mL of cold NEt_3, then 0.294 mL $CHCl_3$ was added and the sample cooled to 0 °C for 20 minutes before starting the measurement. At each temperature, the sample was left to equilibrate for 1 minute before measuring. Measurement was performed on a Chirascan V100 CD spectrophotometer.

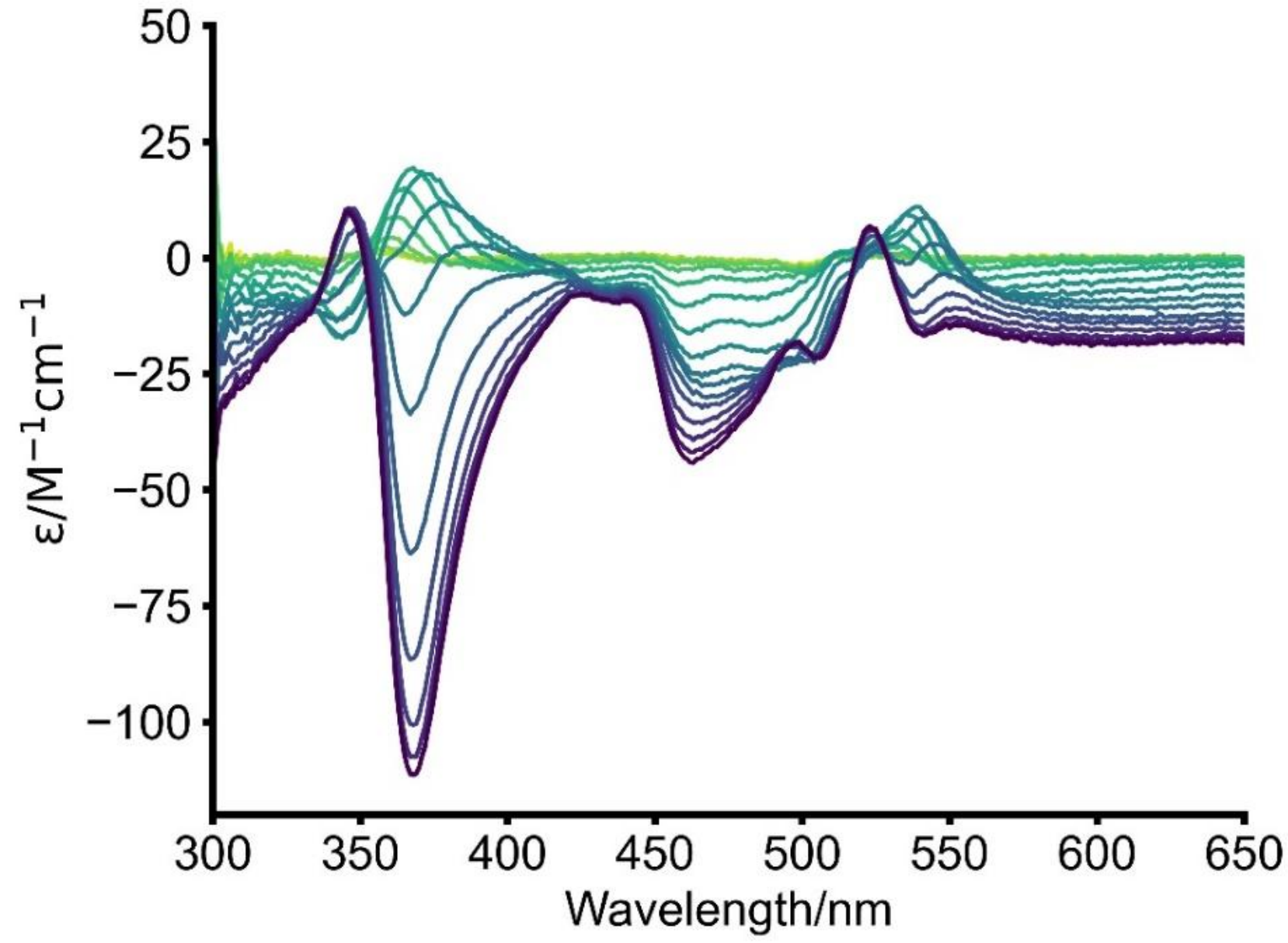

Supplementary Figure 3-15: Changes in circular dichroism of CDI **4a** in 9:1 NEt_3:$CHCl_3$ (10 μM) after heating (**Supplementary Figure 3-14**) upon decreasing the temperature from 85 °C (yellow) to 0 °C (purple) in 5 °C increments. Sample was prepared by addition of 6 μL of a 5 mM stock solution of compound **4a** in $CHCl_3$ to 2.7 mL of cold NEt_3, then 0.294 mL $CHCl_3$ was added and the sample cooled to 0 °C for 20 minutes before starting the measurement. At each temperature, the sample was left to equilibrate for 1 minute before measuring. Measurement was performed on a Chirascan V100 CD spectrophotometer.

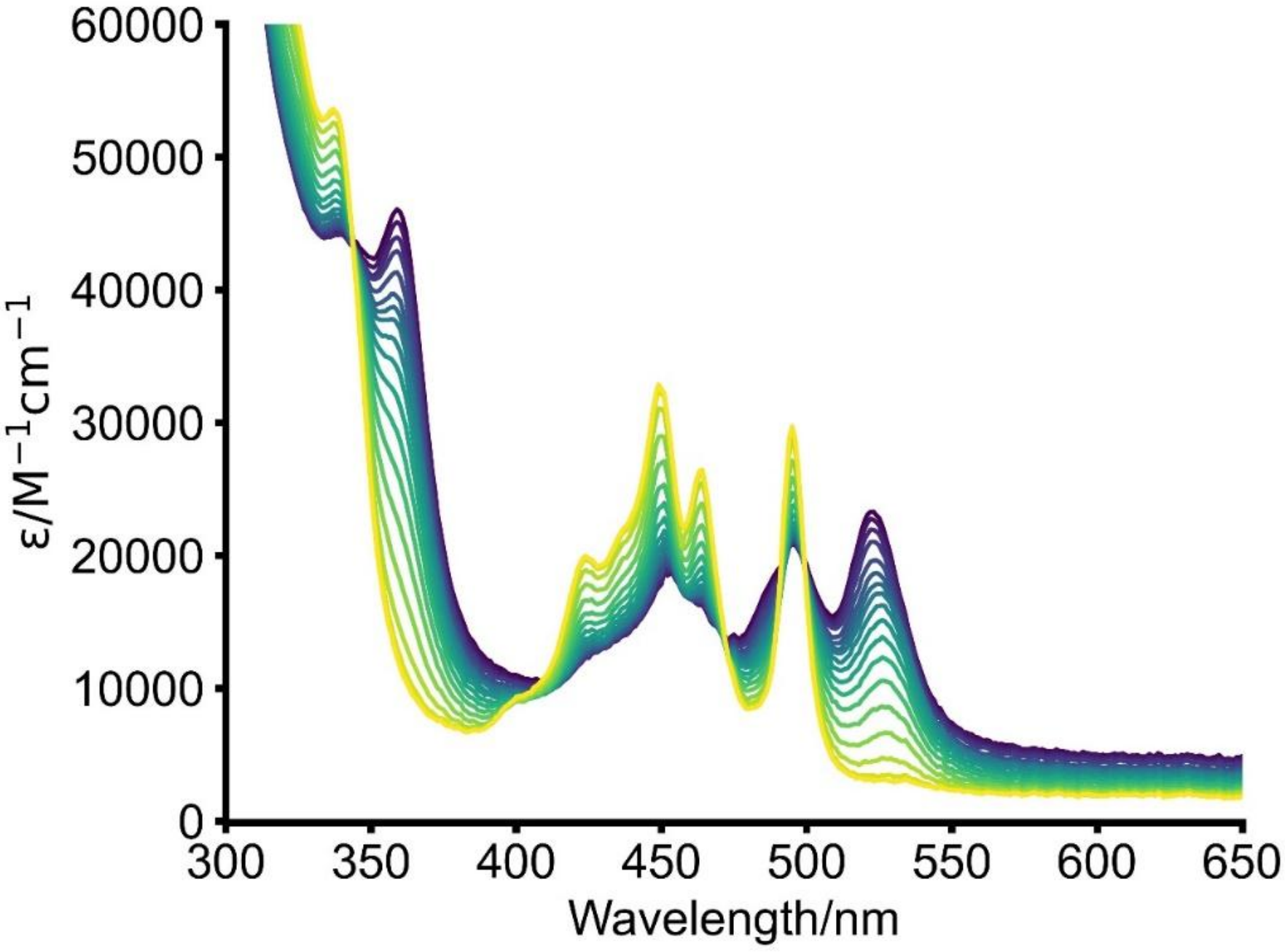

Supplementary Figure 3-16: Changes in absorbance of CDI **4a** in 9:1 NEt_3:$CHCl_3$ (20 μM) with varying temperature from 0 °C (purple) to 85 °C (yellow) in 5°C increments. Sample was prepared by addition of 12 μL of a 5 mM stock solution of compound **4a** in $CHCl_3$ to 2.7 mL of cold NEt_3, then 0.288 mL $CHCl_3$ was added and the sample cooled to 0 °C for 15 minutes before starting the measurement. At each temperature, the sample was left to equilibrate for 1 minute before measuring. Measurement was performed on a Chirascan V100 CD spectrophotometer.

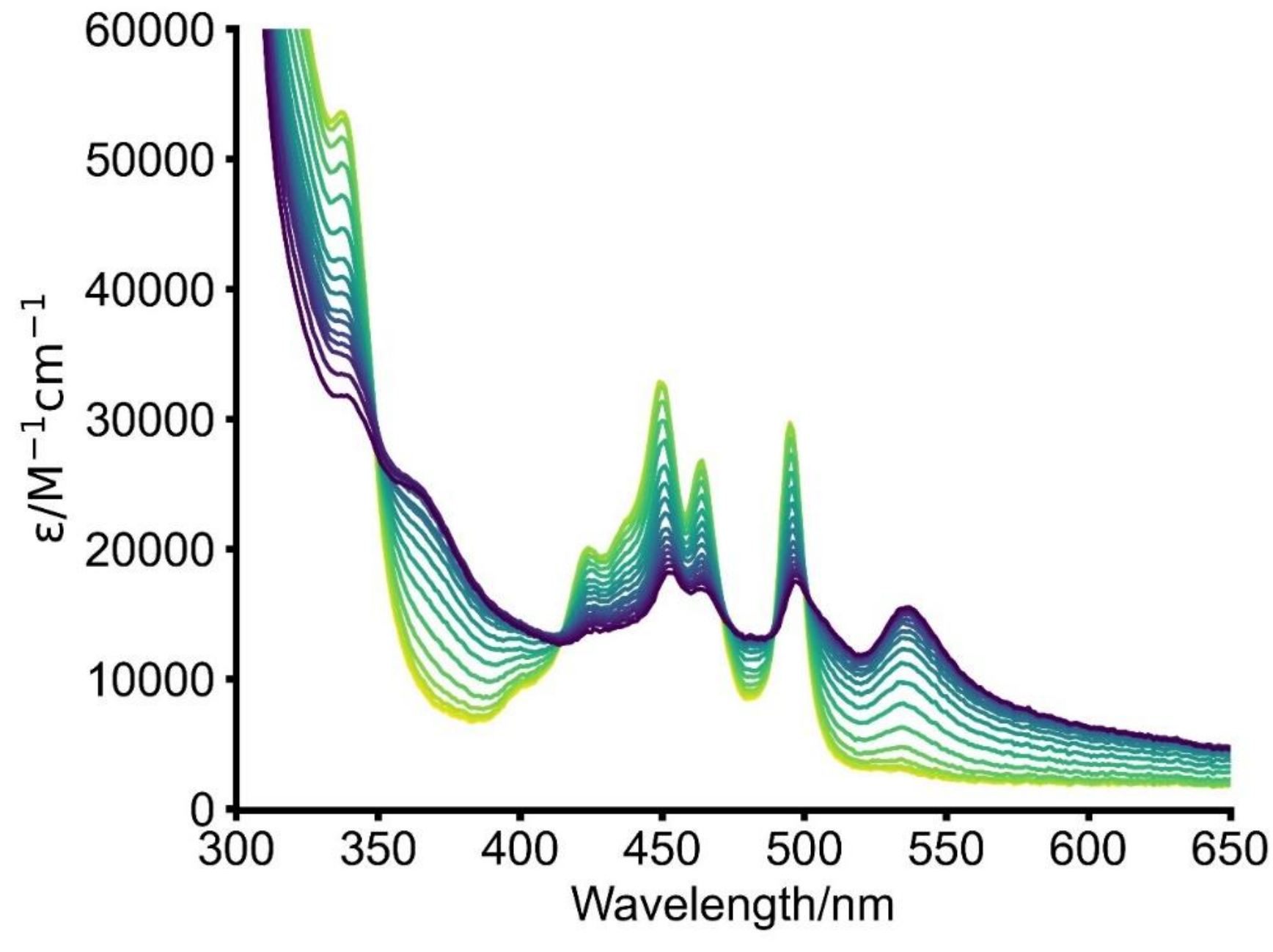

Supplementary Figure 3-17: Changes in absorbance of CDI **4a** in 9:1 NEt_3:$CHCl_3$ (20 μM) after heating (**Supplementary Figure 3-16**) upon decreasing the temperature from 85 °C (yellow) to 0 °C (purple) in 5 °C increments. Sample was prepared by addition of 12 μL of a 5 mM stock solution of compound **4a** in $CHCl_3$ to 2.7 mL of cold NEt_3, then 0.288 mL $CHCl_3$ was added and the sample cooled to 0 °C for 20 minutes before starting the measurement. At each temperature, the sample was left to equilibrate for 1 minute before measuring. Measurement was performed on a Chirascan V100 CD spectrophotometer.

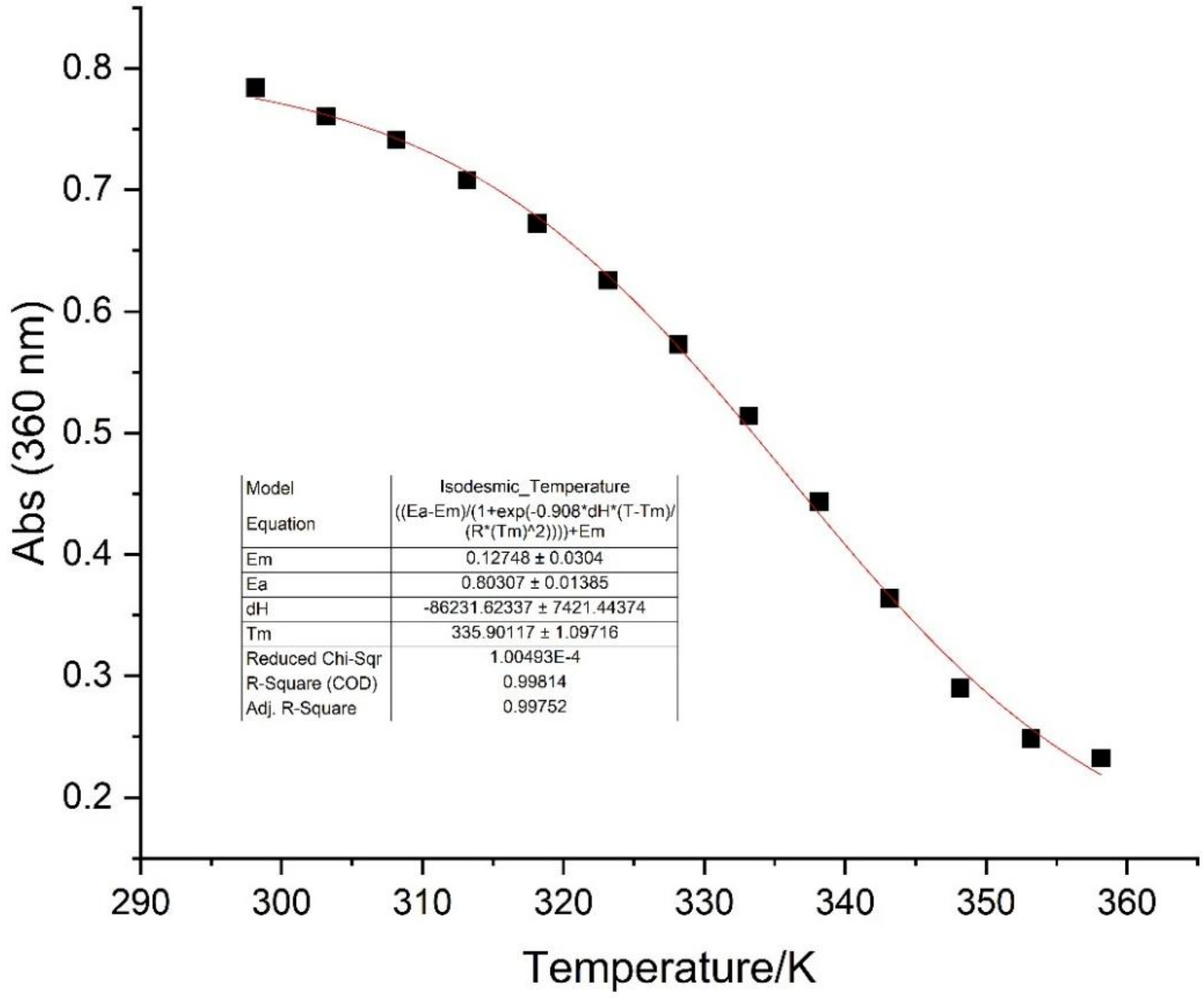

Supplementary Figure 3-18: Fitting of the absorbance at 360 nm from **Supplementary Figure 3-16** (heating) over the range from 298 K to 358 K to a temperature-dependent isodesmic aggregation model.[6]

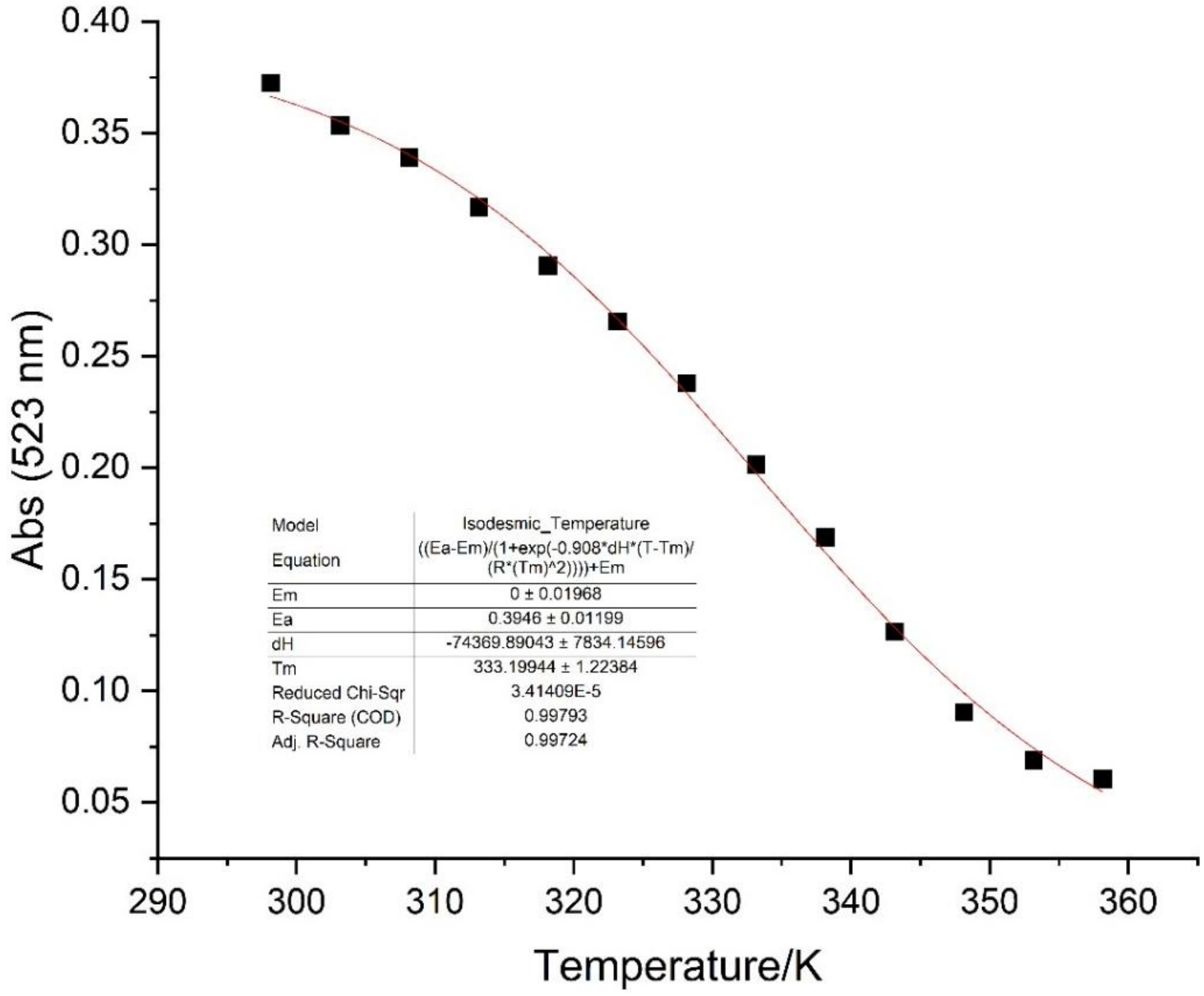

Supplementary Figure 3-19: Fitting of the absorbance at 523 nm from **Supplementary Figure 3-16** (heating) over the range from 298 K to 358 K to a temperature-dependent isodesmic aggregation model.[6]

Supplementary Table 3-3: Thermodynamic parameters computed from the isodesmic fit to the absorbance data (**Supplementary Figure 3-18 and 3-19**). Data reported was calculated for 298 K.

**Index**	**Wavelength (nm)**	**ΔH (kJ mol^{-1})**	**ΔG (kJ mol^{-1})**	**K_e (x10^5 M^{-1})**	**ΔS (J K^{-1} mol^{-1})**	**T_m (K)**	**DP_N**
**1**	360	-86.23	-36.08	20.98	-186.20	335.90	6.00
**2**	523	-74.37	-34.48	10.99	-133.79	333.20	4.22
**3**	Average	-80.30	-35.28	15.99	-160.00	334.55	5.11

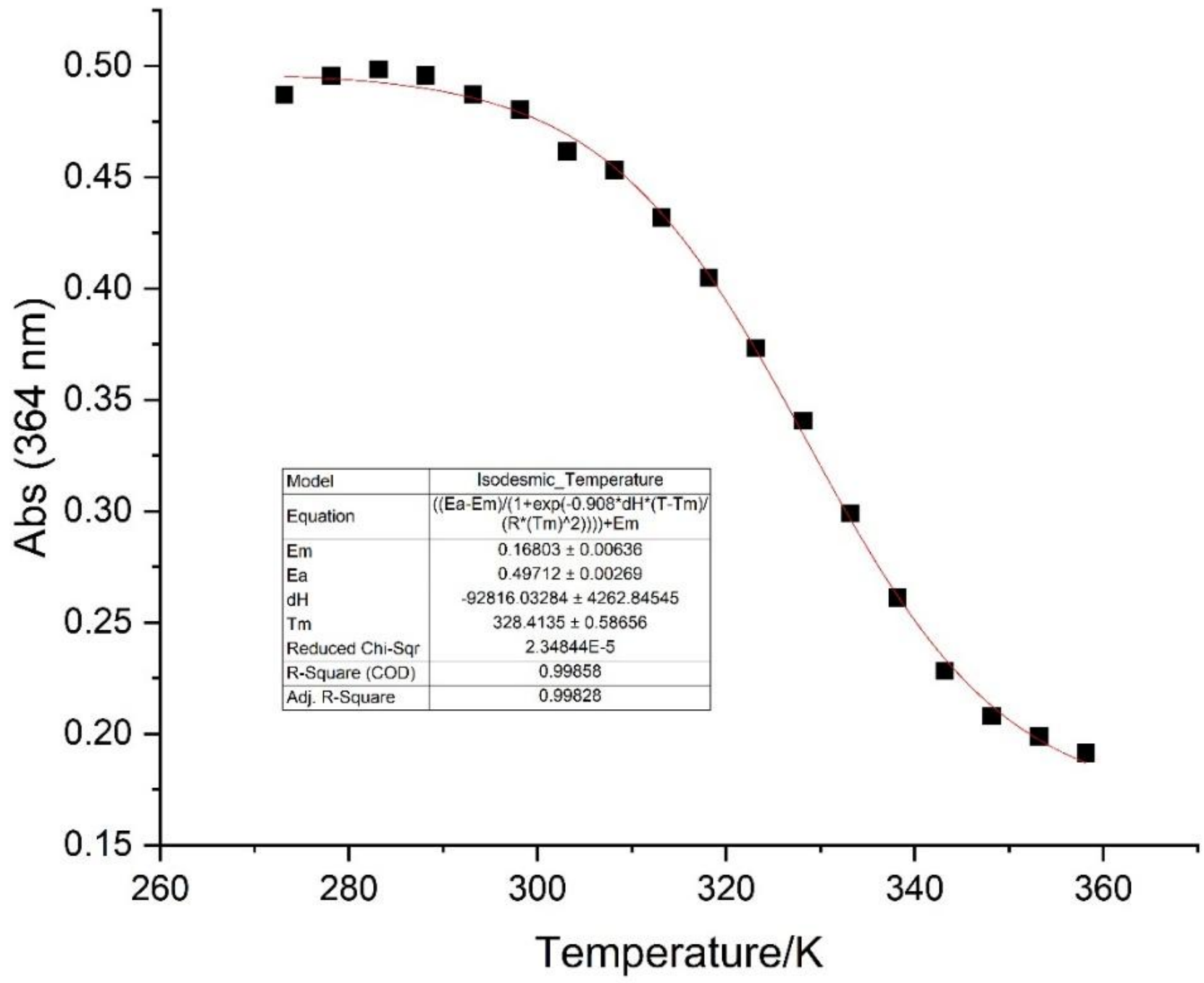

Supplementary Figure 3-20: Fitting of the absorbance at 364 nm from **Supplementary Figure 3-17** (cooling) to a temperature-dependent isodesmic aggregation model.[6]

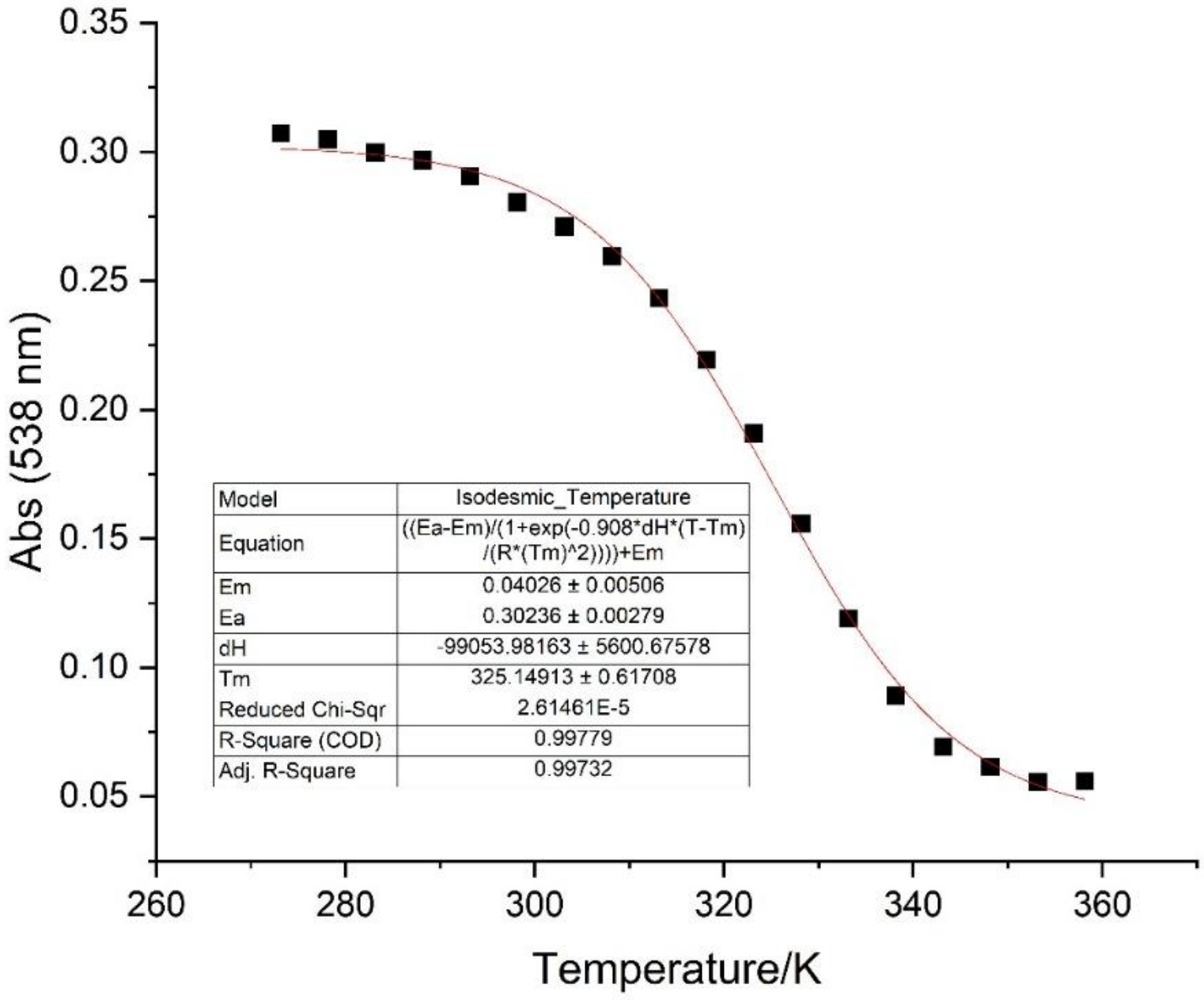

Supplementary Figure 3-21: Fitting of the absorbance at 538 nm from **Supplementary Figure 3-17** (cooling) to a temperature-dependent isodesmic aggregation model.[6]

Supplementary Table 3-4: Thermodynamic parameters computed from the isodesmic fit to the absorbance data (**Supplementary Figures 3-20 and 3-21**). Data reported was calculated for 298 K.

**Index**	**Wavelength (nm)**	**ΔH (kJ mol^{-1})**	**ΔG (kJ mol^{-1})**	**K_e (x10^5 M^{-1})**	**ΔS (J K^{-1} mol^{-1})**	**T_m (K)**	**DP_N**
**1**	364	-92.81	-34.70	12.01	-194.88	328.41	4.43
**2**	538	-99.05	-33.60	7.71	-219.52	325.15	3.46
**3**	Average	-95.93	-34.15	9.86	-207.20	326.78	3.95

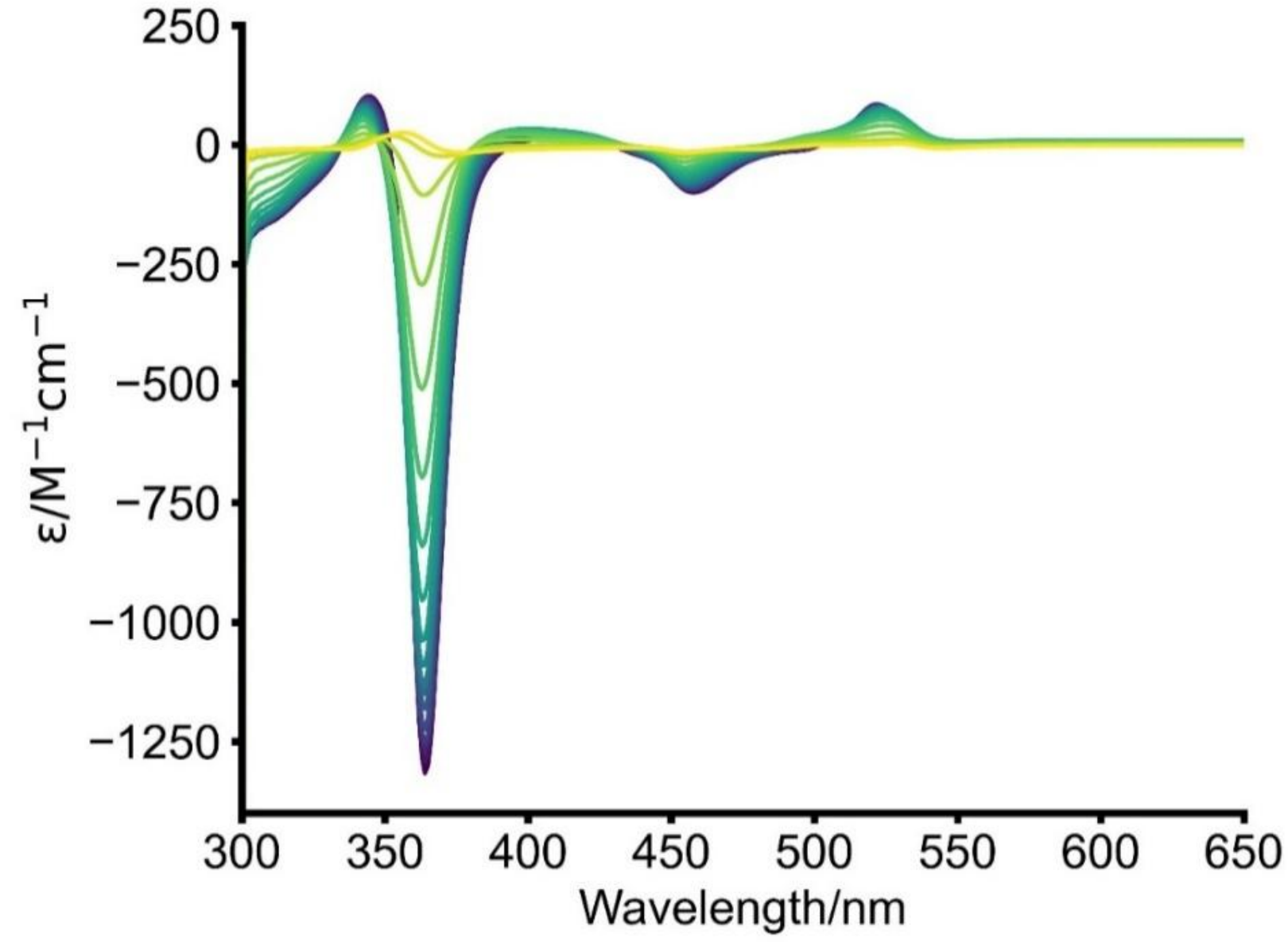

Supplementary Figure 3-22: Changes in circular dichroism of CDI **4a** in 9:1 NEt_3:$CHCl_3$ (20 µM) with varying temperature from 0 °C (purple) to 85 °C (yellow) in 5°C increments. Sample was prepared by addition of 12 µL of a 5 mM stock solution of compound **4a** in $CHCl_3$ to 2.7 mL of cold NEt_3, then 0.288 mL $CHCl_3$ was added and the sample cooled to 0 °C for 15 minutes before starting the measurement. At each temperature, the sample was left to equilibrate for 1 minute before measuring. Measurement was performed on a Chirascan V100 CD spectrophotometer.

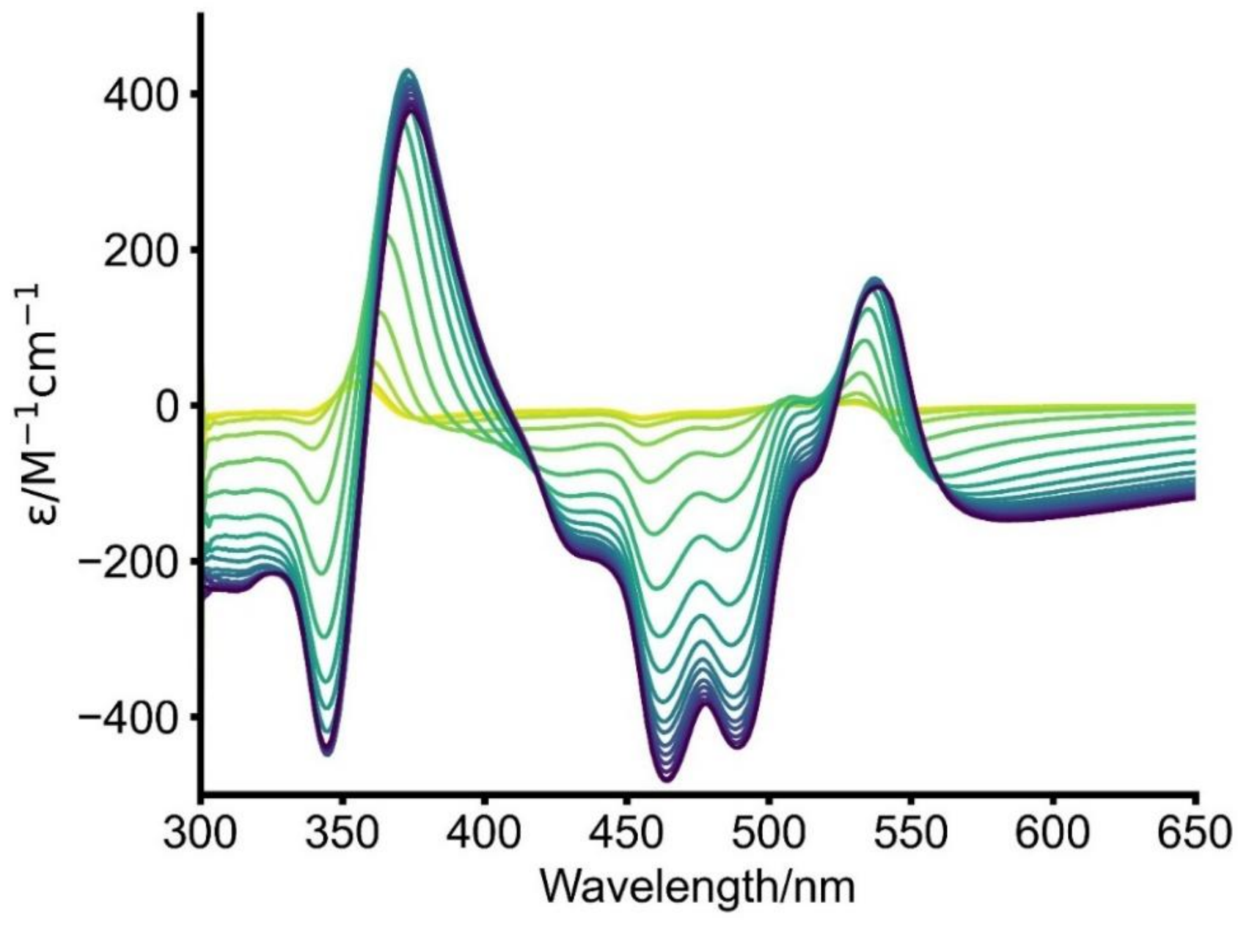

Supplementary Figure 3-23: Changes in circular dichroism of CDI **4a** in 9:1 NEt_3:$CHCl_3$ (20 µM) after heating (**Supplementary Figure 3-22**) upon decreasing the temperature from 85 °C (yellow) to 0 °C (purple) in 5 °C increments. Sample was prepared by addition of 12 µL of a 5 mM stock solution of compound **4a** in $CHCl_3$ to 2.7 mL of cold NEt_3, then 0.288 mL $CHCl_3$ was added and the sample cooled to 0 °C for 20 minutes before starting the measurement. At each temperature, the sample was left to equilibrate for 1 minute before measuring. Measurement was performed on a Chirascan V100 CD spectrophotometer.

Concentration-dependent self-assembly in 9:1 NEt_3:$CHCl_3$

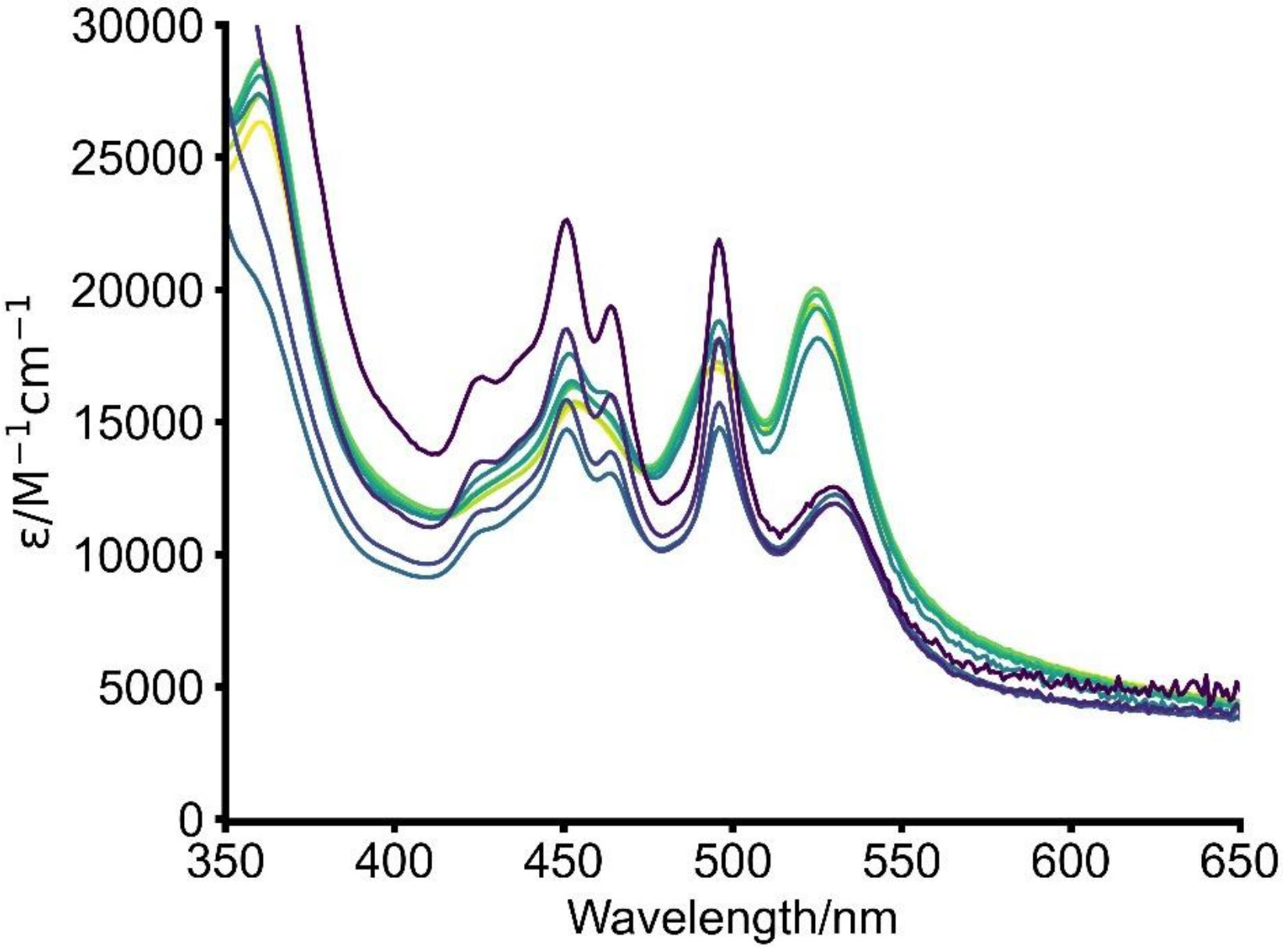

Supplementary Figure 3-24: Changes in absorbance profile over varying concentrations of CDI **4a** in 9:1 NEt_3:$CHCl_3$. Measurement was performed on a Thermo Scientific Genesys 150 spectrophotometer.

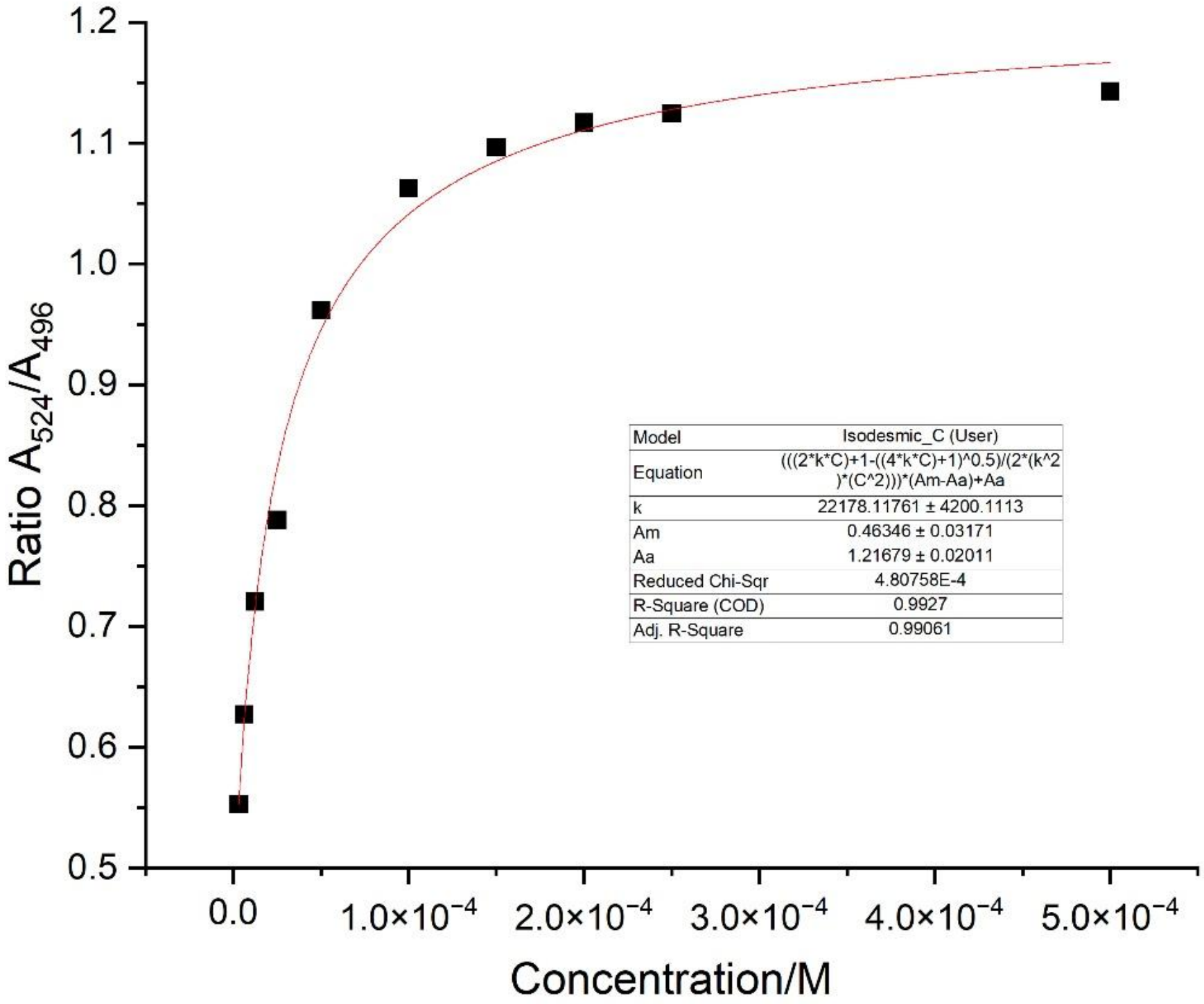

Supplementary Figure 3-25: Fitting of the ratio of absorbance peaks for aggregate and monomer from **Supplementary Figure 3-24** to a concentration-dependent isodesmic model.

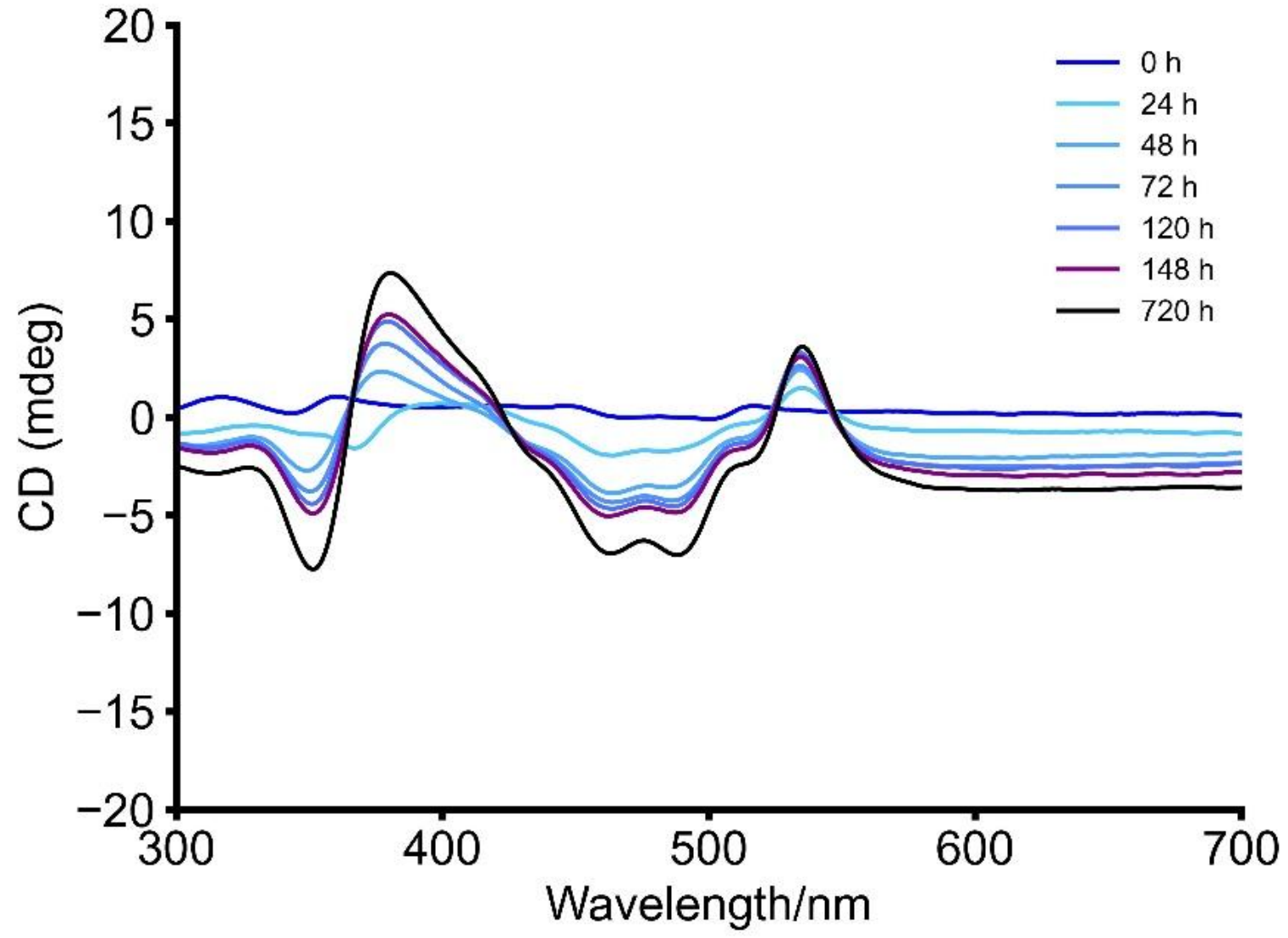

Supplementary Figure 3-26: Changes in circular dichroism of CDI **4a** in 3:1 MeOH:$CHCl_3$ (10 µM) after heating and cooling (**Supplementary Figure 3-10**) over the time course of 1 month.

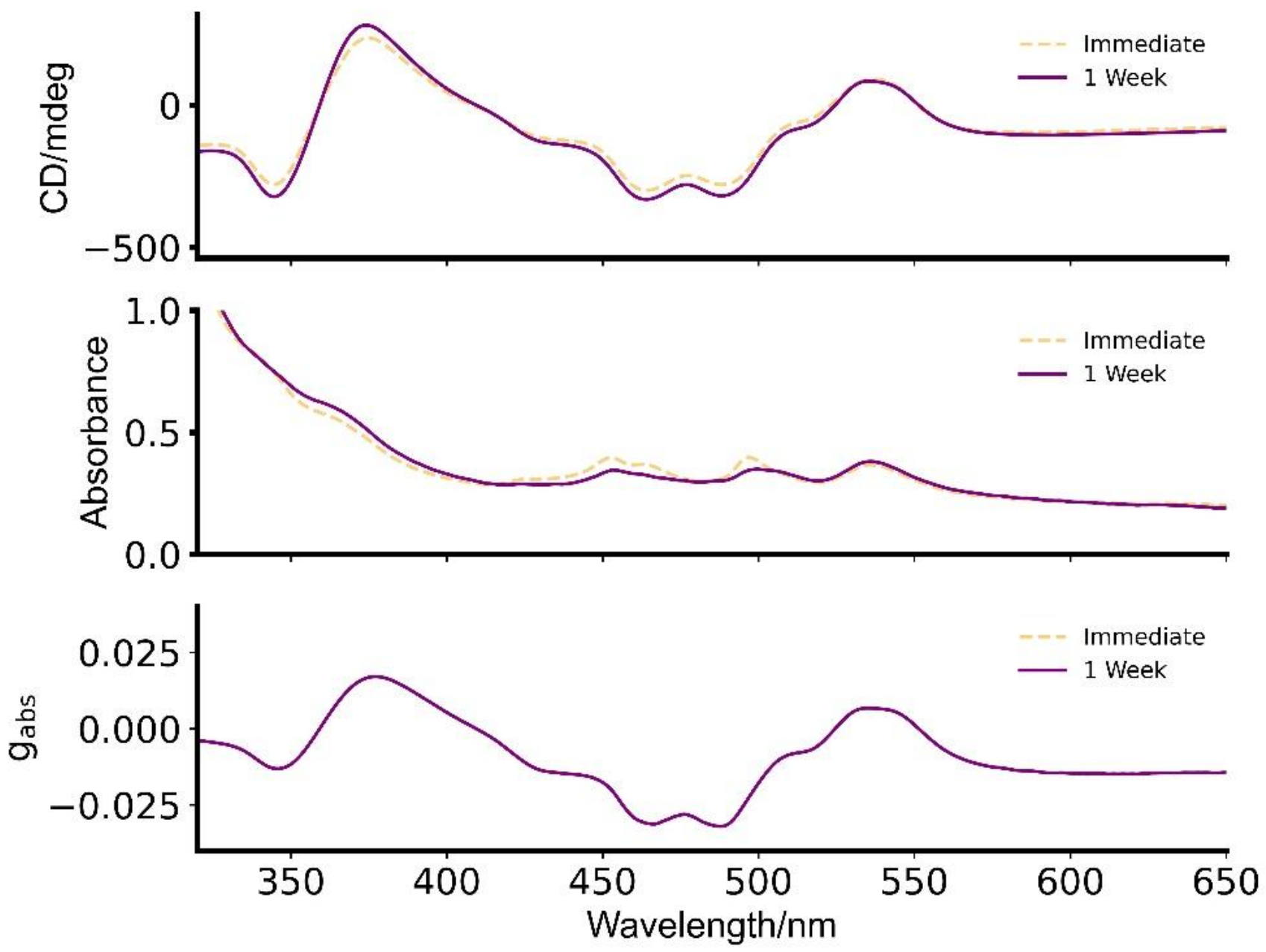

Supplementary Figure 3-27: Changes in circular dichroism of CDI **4a** in 9:1 NEt_3:$CHCl_3$ (20 µM) after heating and cooling (**Supplementary Figure 3-23**) after 1 week.

Isodesmic fits after scatter-correction

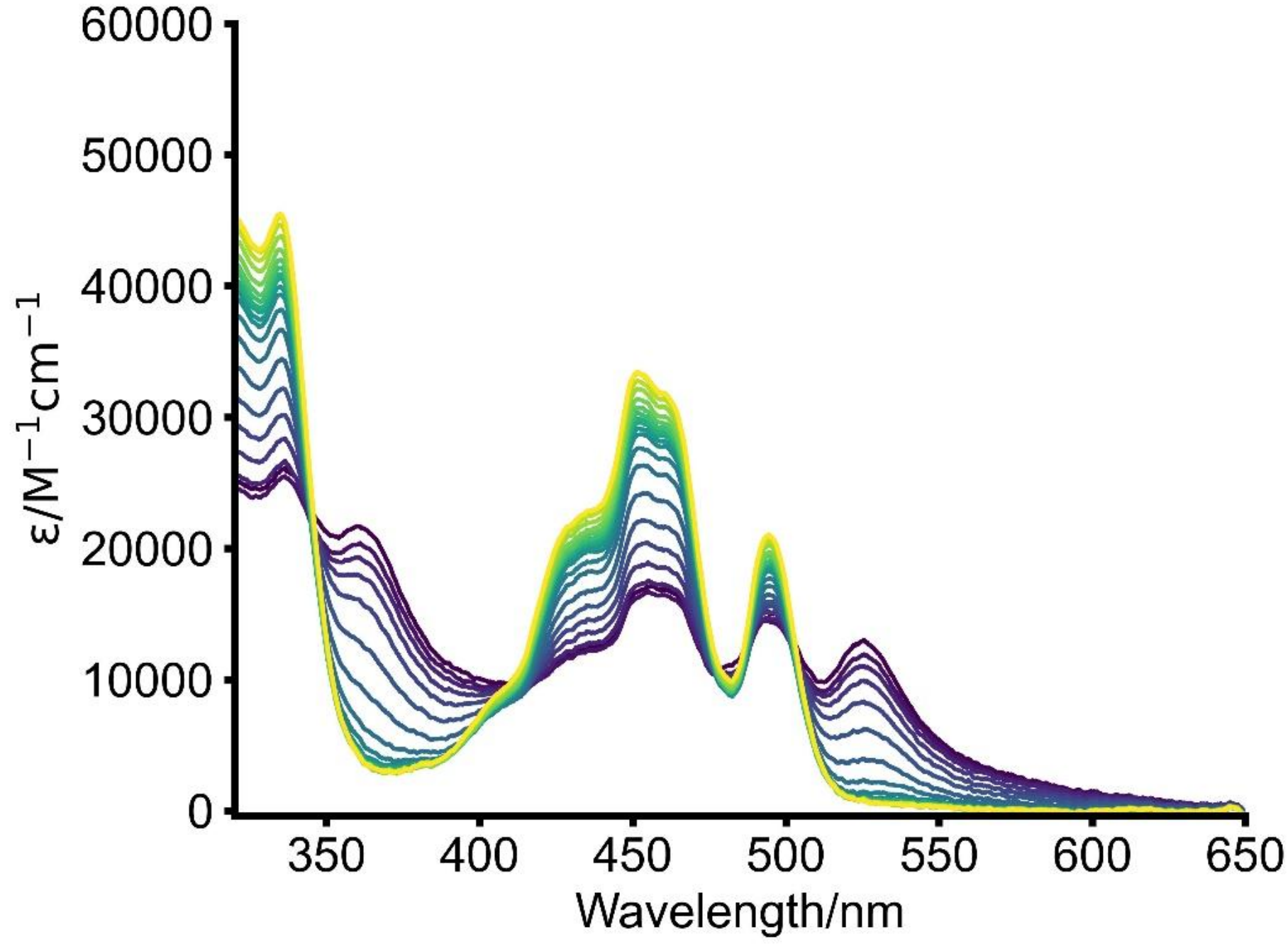

Supplementary Figure 3-28: Changes in absorbance of CDI **4a** in 3:1 MeOH:$CHCl_3$ (10 μM) with varying temperature from 0 °C (purple) to 65 °C (yellow) from **Supplementary Figure 3-5** after correction for scattering using the a|e - UV-Vis-IR Spectral Software by FluorTools. A linear scatter correction was applied by fitting the region from 670-700 nm.

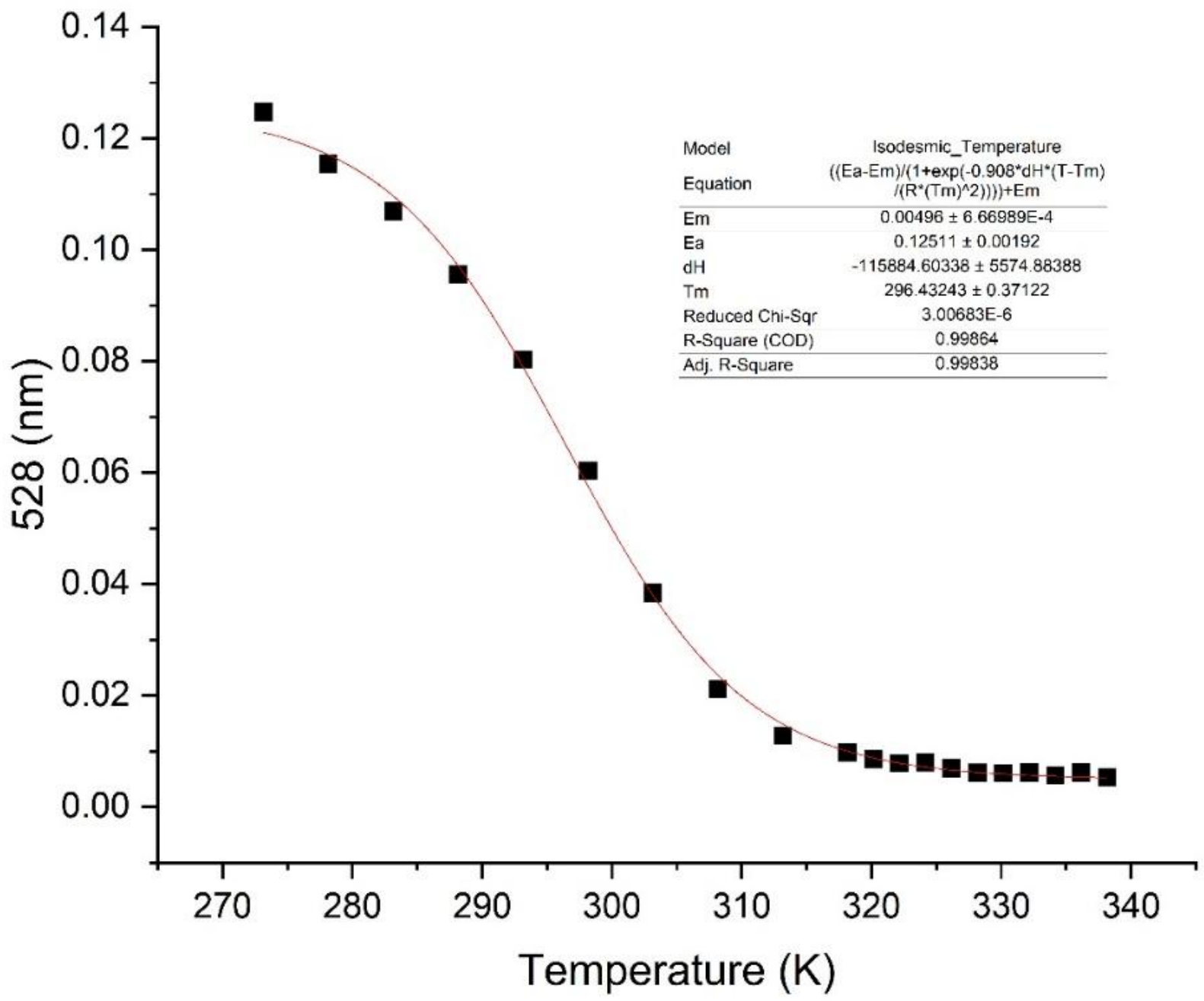

Supplementary Figure 3-29: Fitting of the absorbance at 528 nm from **Supplementary Figure 3-28** to a temperature-dependent isodesmic aggregation model.[6]

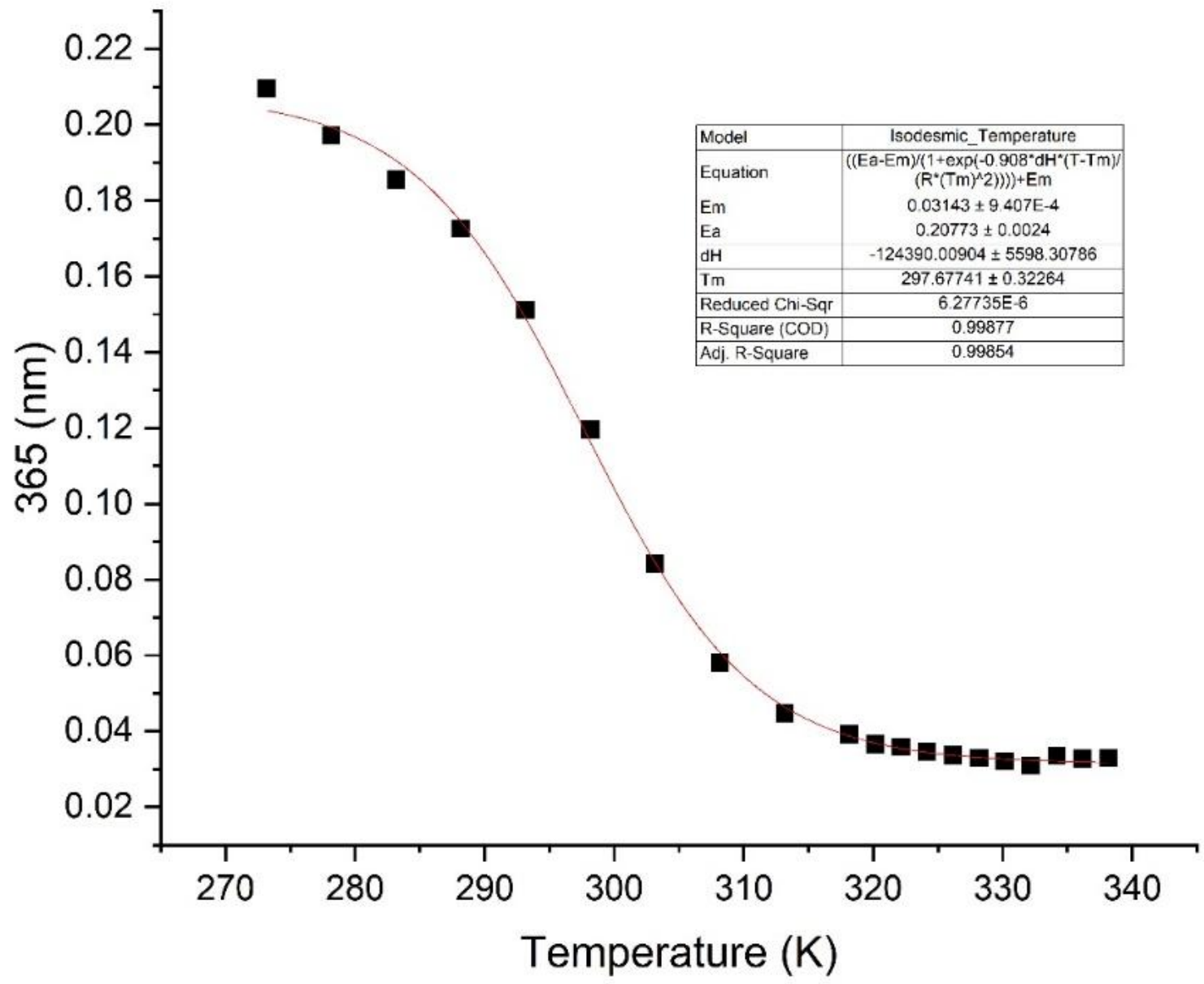

Supplementary Figure 3-30: Fitting of the absorbance at 365 nm from **Supplementary Figure 3-28** to a temperature-dependent isodesmic aggregation model.[6]

Supplementary Table 3-5: Thermodynamic parameters computed from the isodesmic fit to the absorbance data (**Supplementary Figures 3-29** and **3-30**). Data reported was calculated for 298 K.

**Index**	**Wavelength (nm)**	**ΔH (kJ mol^{-1})**	**ΔG (kJ mol^{-1})**	**K_e (x10^5 M^{-1})**	**ΔS (J K^{-1} mol^{-1})**	**T_m (K)**	**DP_N**
**1**	528	–115.88	–31.43	3.22	–283.25	296.43	1.36
**2**	365	–124.39	–31.58	3.42	–311.28	297.67	1.41
**3**	Average	–120.14	–31.51	3.32	–297.27	297.05	1.39

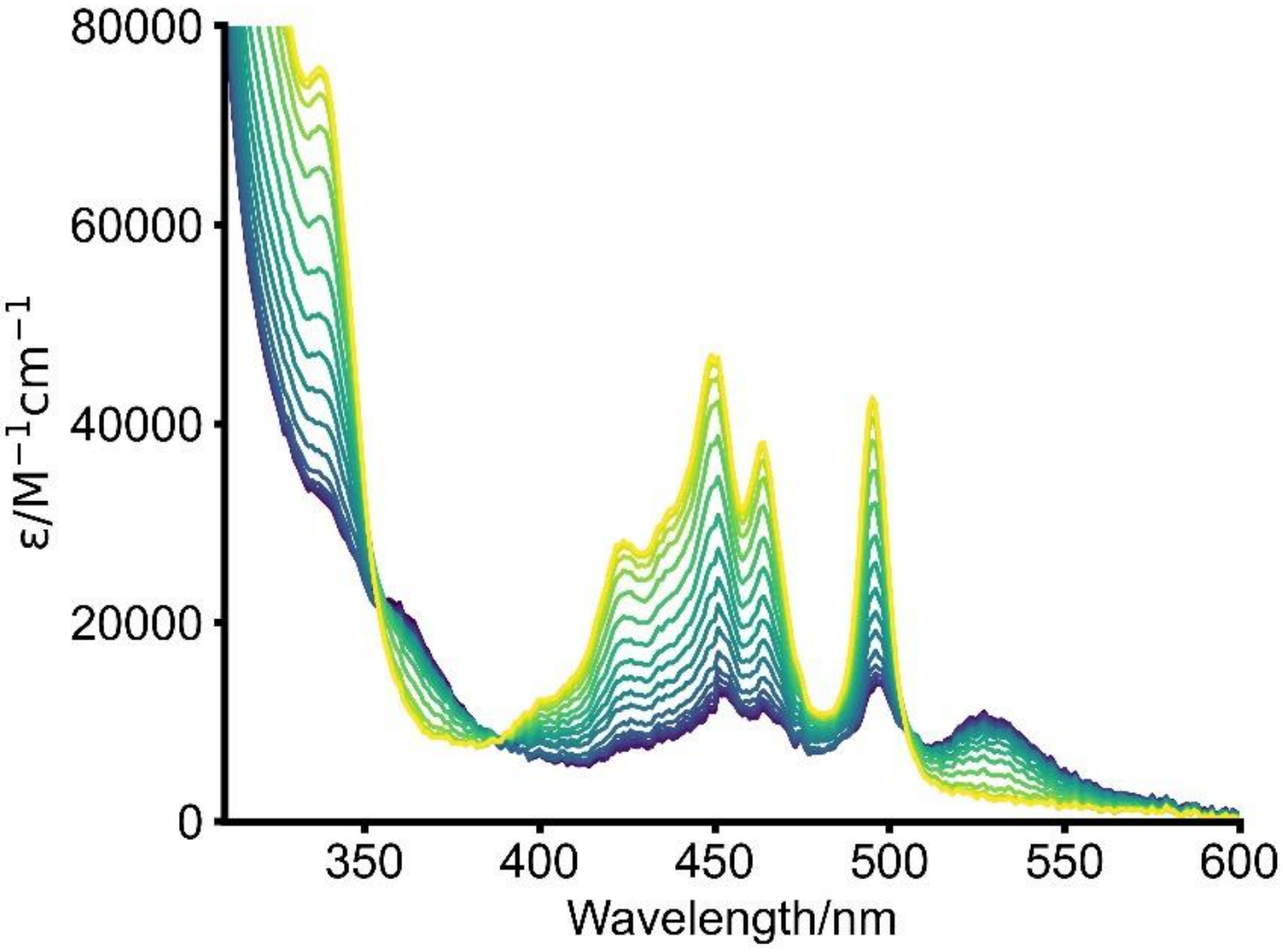

Supplementary Figure 3-31: Changes in absorbance of CDI **4a** in 9:1 NEt_3:$CHCl_3$ (10 μM) with varying temperature from 0 °C (purple) to 85 °C (yellow) from **Supplementary Figure 3-12** after correction for scattering using the a|e - UV-Vis-IR Spectral Software by FluorTools. A linear scatter correction was applied by fitting the region around 620 nm to a linear equation.

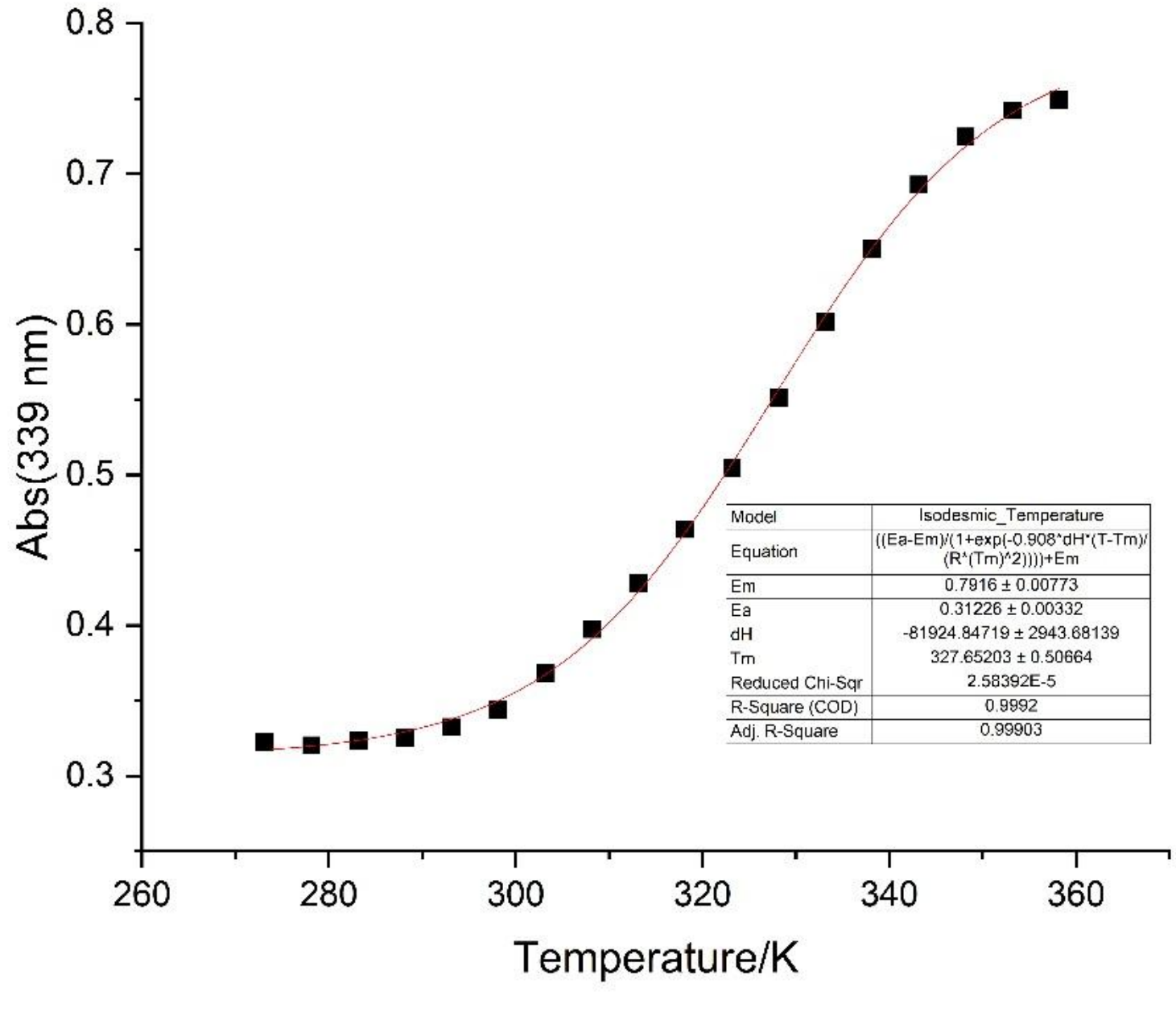

Supplementary Figure 3-32: Fitting of the absorbance at 339 nm from **Supplementary Figure 3-31** to a temperature-dependent isodesmic aggregation model.[6]

Supplementary Table 3-6: Thermodynamic parameters computed from the isodesmic fit to the absorbance data (**Supplementary Figure 3-32**). Data reported was calculated for 298 K.

**Index**	**Wavelength (nm)**	**ΔH (kJ mol⁻¹)**	**ΔG (kJ mol⁻¹)**	***K*$_e$ (x10^5 M^{-1})**	**ΔS (J K⁻¹ mol⁻¹)**	**T$_m$ (K)**	***DP*$_N$**
**1**	339	–81.92	–35.82	18.90	–155.62	327.65	3.88

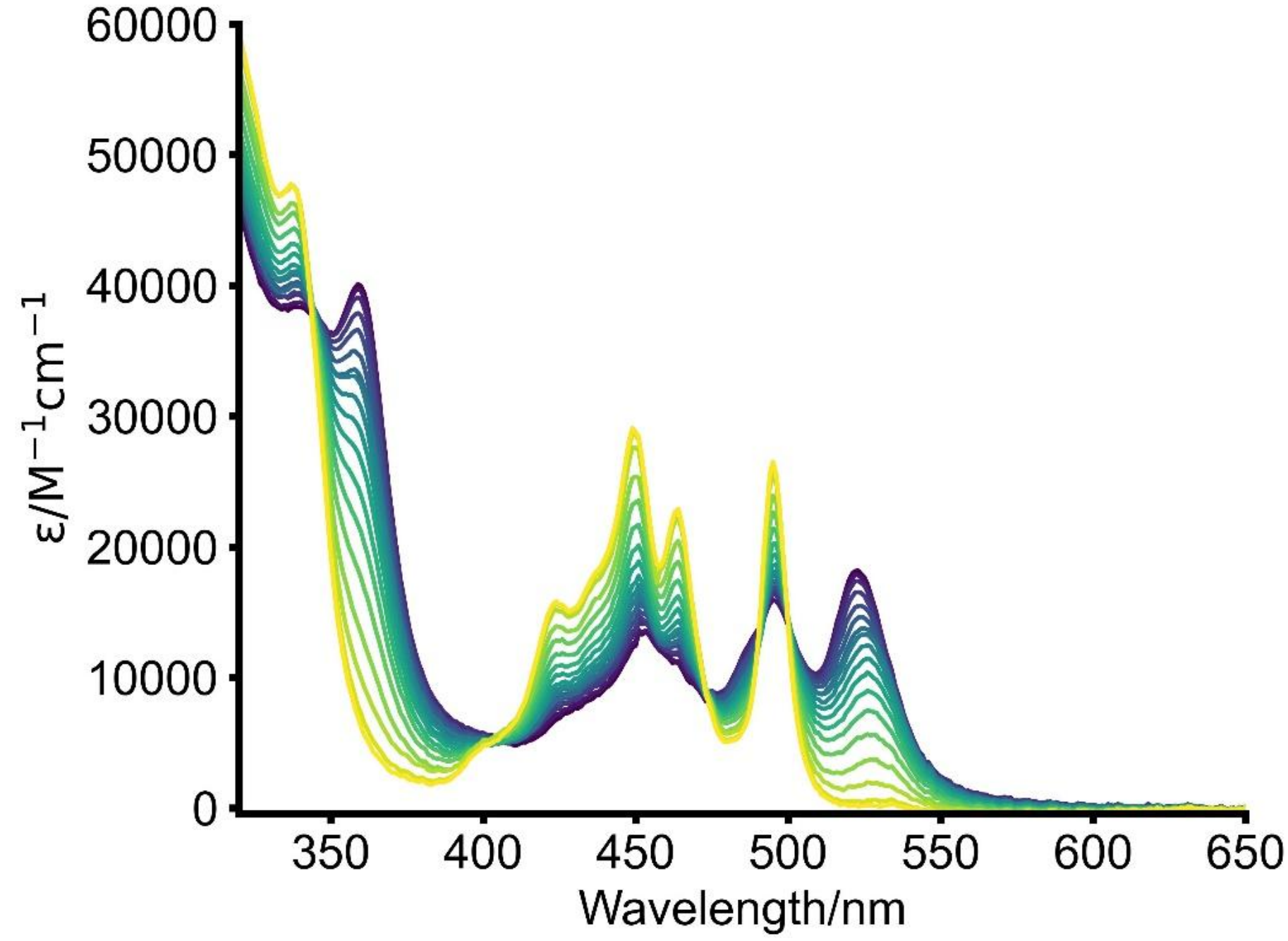

Supplementary Figure 3-33: Changes in absorbance of CDI **4a** in 9:1 NEt_3:$CHCl_3$ (20 μM) with varying temperature from 0 °C (purple) to 85 °C (yellow) from **Supplementary Figure 3-16** after correction for scattering using the a|e - UV-Vis-IR Spectral Software by FluorTools. An alternative scatter correction was applied by fitting the region from 650-750 nm to the equation y = log(1/(1-a/x))+b.

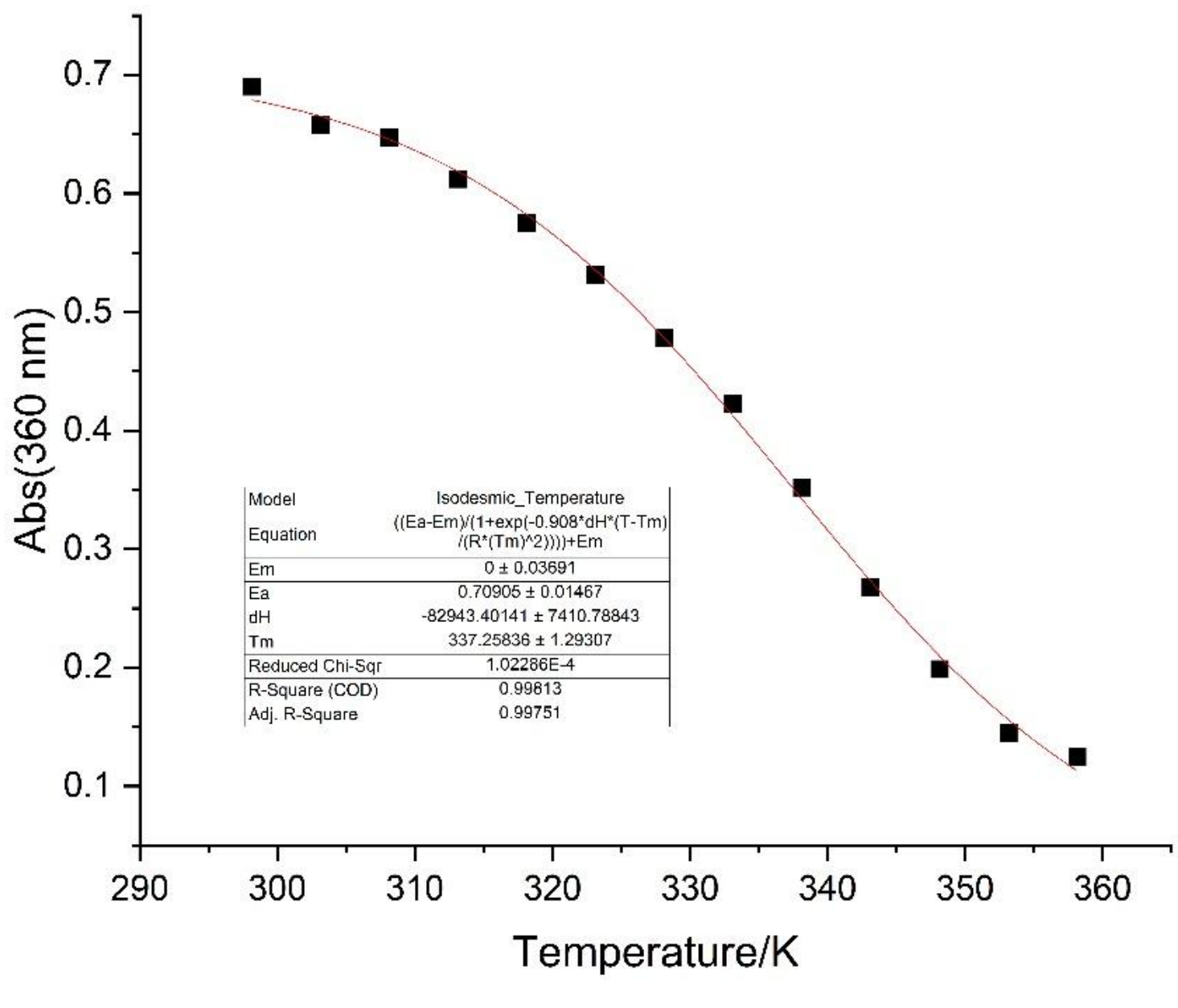

Supplementary Figure 3-34: Fitting of the absorbance at 360 nm from **Supplementary Figure 3-33** to a temperature-dependent isodesmic aggregation model.[6]

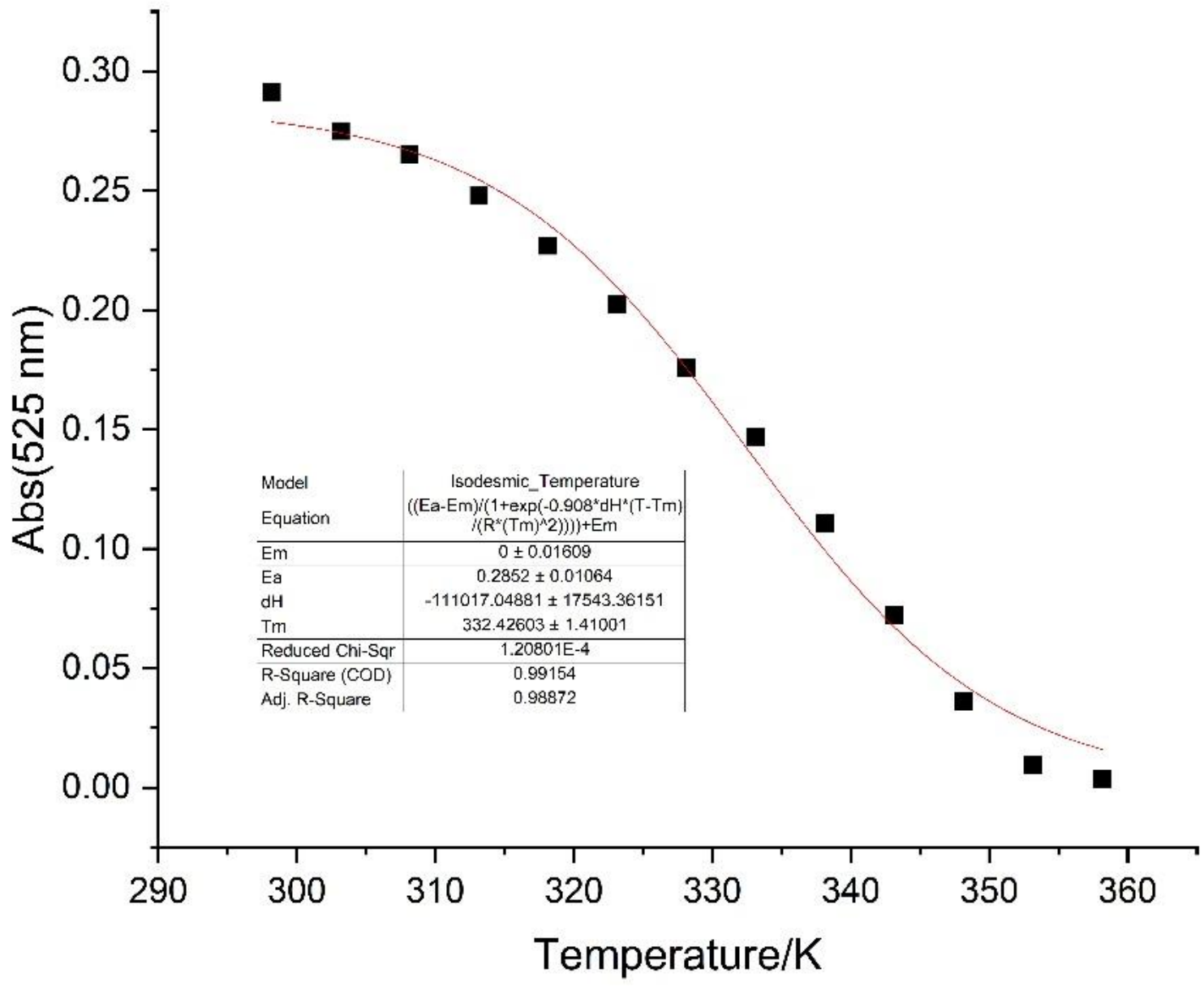

Supplementary Figure 3-35: Fitting of the absorbance at 365 nm from **Supplementary Figure 3-33** to a temperature-dependent isodesmic aggregation model.[6]

Supplementary Table 3-7: Thermodynamic parameters computed from the isodesmic fit to the absorbance data (**Supplementary Figures 3-34** and **3-35**). Data reported was calculated for 298 K.

**Index**	**Wavelength (nm)**	**ΔH (kJ mol^{-1})**	**ΔG (kJ mol^{-1})**	**K_e (x10^5 M^{-1})**	**ΔS (J K^{-1} mol^{-1})**	**T_m (K)**	**DP_N**
**1**	360	–82.94	–36.20	22.03	–156.77	337.26	6.16
**2**	525	–111.02	–35.55	16.95	–253.11	332.43	5.34
**3**	Average	–96.98	–35.88	19.49	–204.94	334.85	5.75

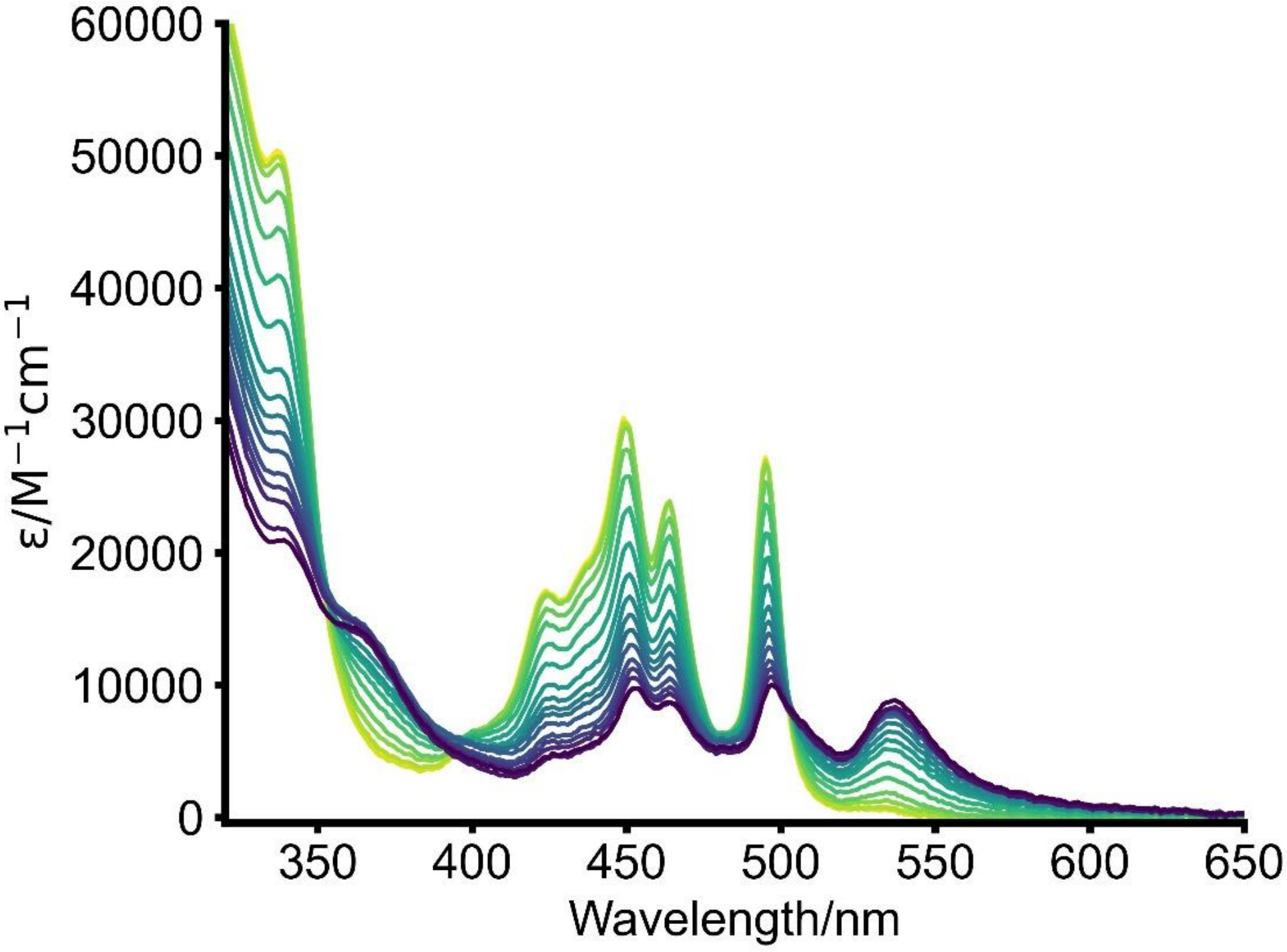

Supplementary Figure 3-36: Changes in absorbance of CDI **4a** in 9:1 NEt_3:$CHCl_3$ (20 µM) upon decreasing the temperature from 85 °C (yellow) to 0 °C (purple) from **Supplementary Figure 3-17** after correction for scattering using the a|e - UV-Vis-IR Spectral Software by FluorTools. A linear scatter correction was applied by fitting a linear equation to datapoints between 700-750 nm.

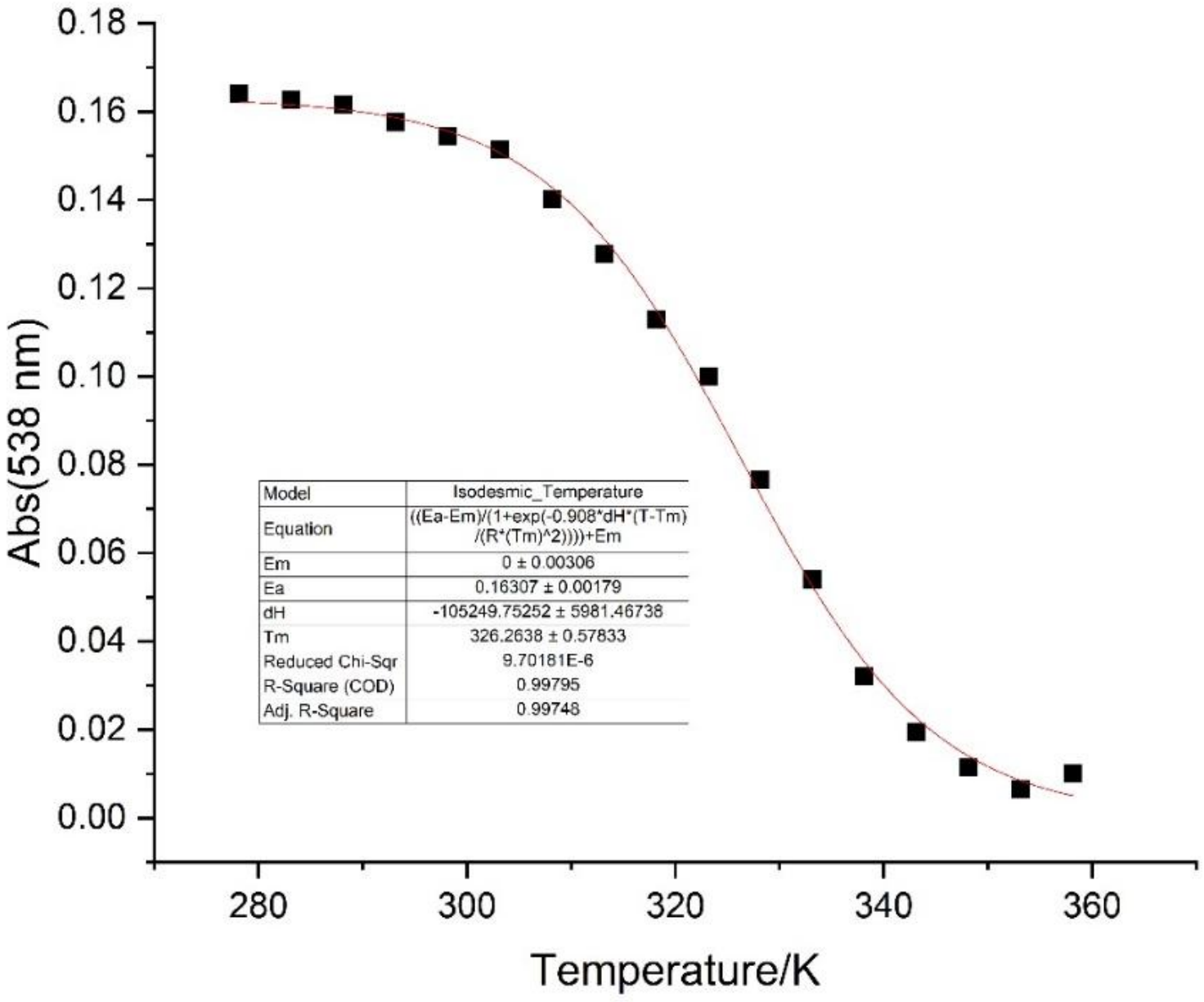

Supplementary Figure 3-37: Fitting of the absorbance at 538 nm from **Supplementary Figure 3-36** to a temperature-dependent isodesmic aggregation model.[6]

Supplementary Table 3-8: Thermodynamic parameters computed from the isodesmic fit to the absorbance data (**Supplementary Figure 3-37**). Data reported was calculated for 298 K.

Index	Wavelength (nm)	ΔH (kJ mol^{-1})	ΔG (kJ mol^{-1})	K_e (x10^5 M^{-1})	ΔS (J K^{-1} mol^{-1})	T_m (K)	DP_N
**1**	538	–105.25	–34.66	11.79	–233.42	326.26	4.38

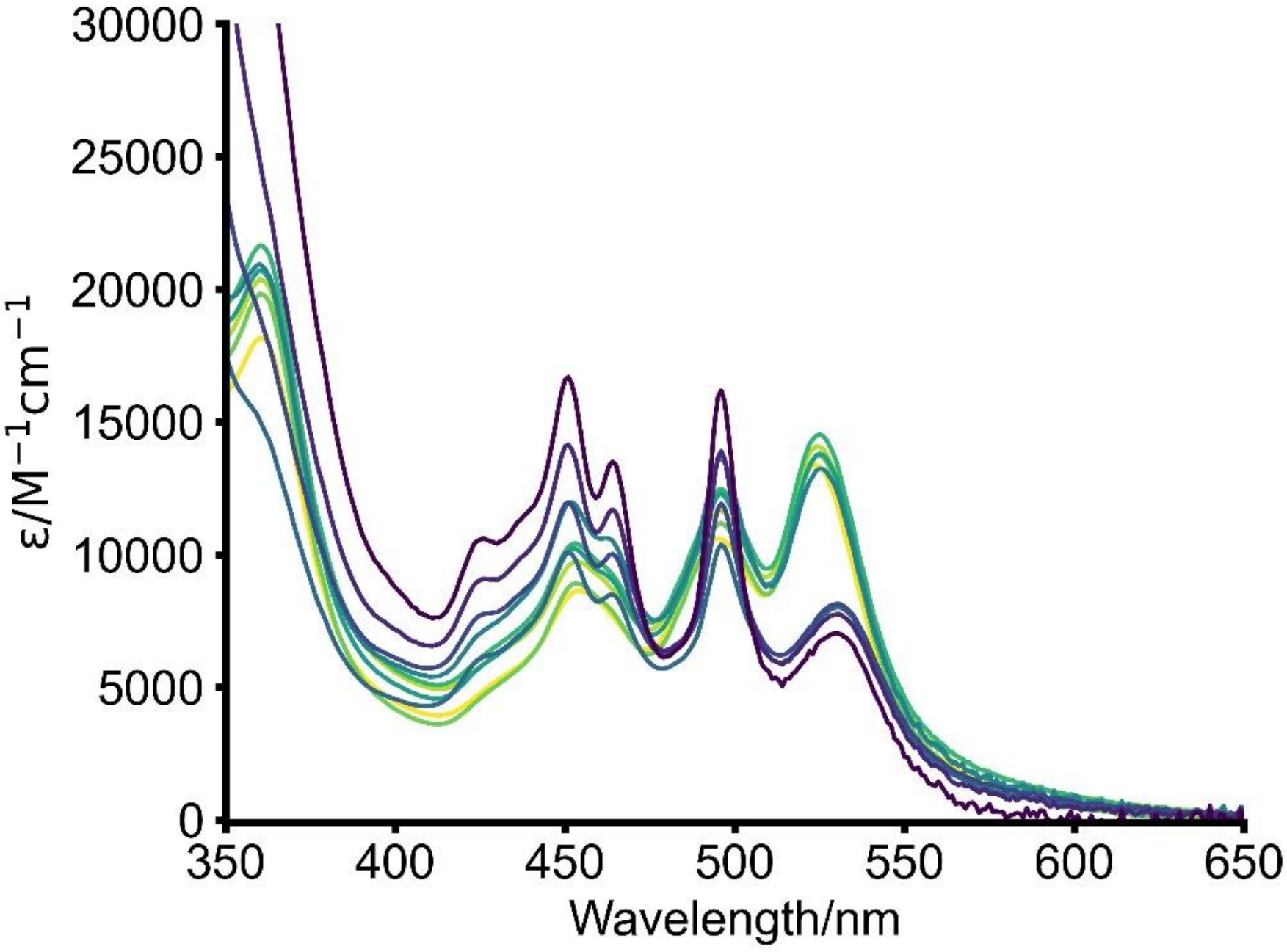

Supplementary Figure 3-38: Changes in absorbance profile over varying concentrations from **Supplementary Figure 3-24** after after correction for scattering using the a|e - UV-Vis-IR Spectral Software by FluorTools. A linear scatter correction was applied by fitting a linear equation to datapoints between 670-750 nm.

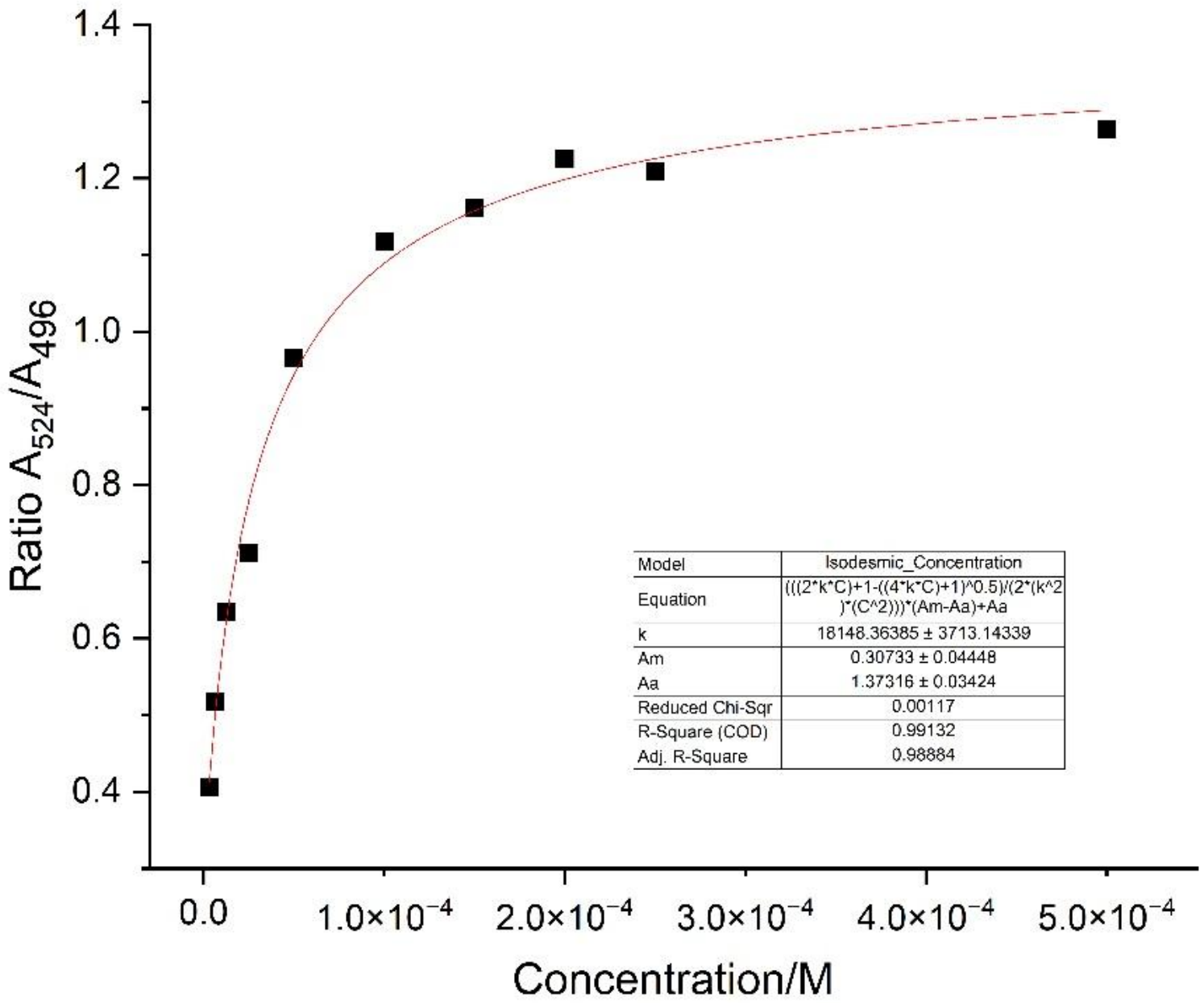

Supplementary Figure 3-39: Fitting of the ratio of absorbance peaks for aggregate and monomer from **Supplementary Figure 3-38** to a concentration-dependent isodesmic model.

Supplementary Table 3-9: A comparison of the association constants (*K*) pre- and post-correction for scattering effects for all experiments in which quantitative analyses were performed.

**Entry**	**Solvent**	**Concentration /μM**	**Method**	***K* pre-correction ($\times 10^5$)**	***K* post-correction ($\times 10^5$)**
**1**	3:1 MeOH:$CHCl_3$	10	Variable Temperature (Heating)	3	3
**2**	9:1 NEt_3:$CHCl_3$	10	Variable Temperature (Heating)	24	19
**3**	9:1 NEt_3:$CHCl_3$	20	Variable Temperature (Heating)	16	20
**4**	9:1 NEt_3:$CHCl_3$	20	Variable Temperature (Cooling). NB: A distinct aggregate to that examined by heating	10	12
**5**	9:1 NEt_3:$CHCl_3$		Variable Concentration	2	2

4) Dynamic Light Scattering

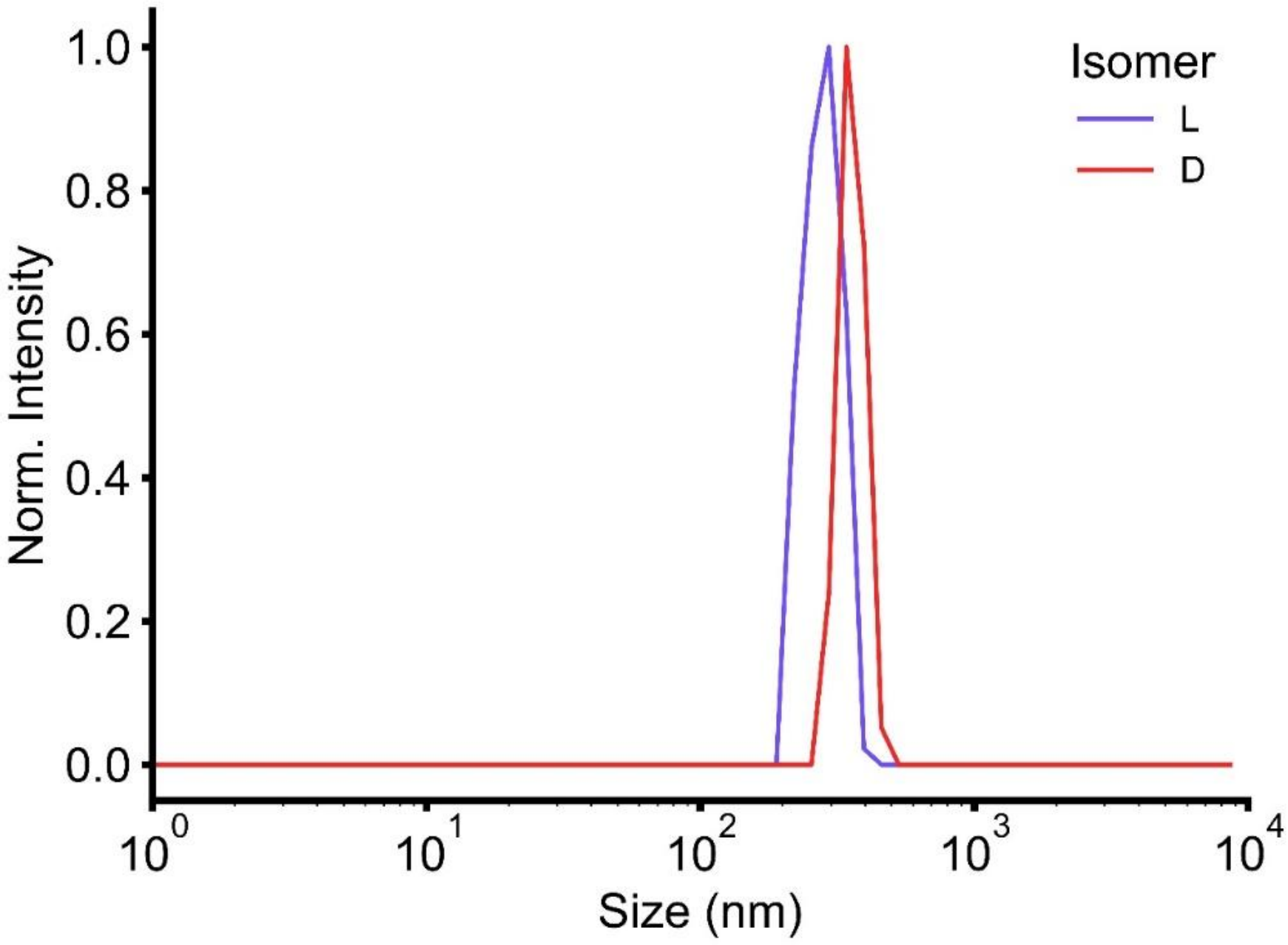

Supplementary Figure 4-1: Dynamic light scattering of **4ab** at 10 µM concentration at 298 K in MeOH. Data recorded on a Malvern Panalytical Zetasizer Nano ZSP. The average of 3 measurements yielded particle sizes for L and D of 473 nm and 602 nm respectively.

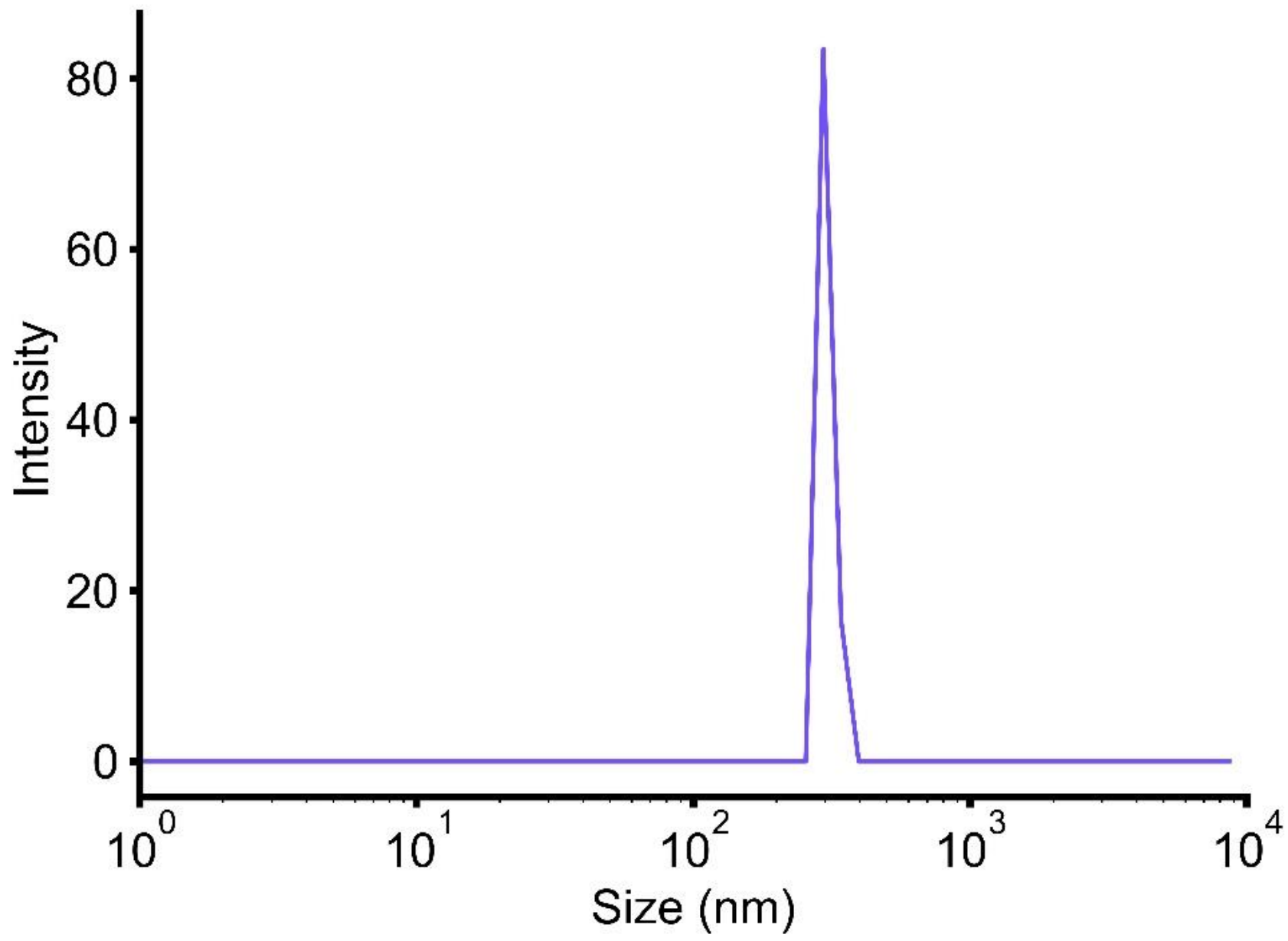

Supplementary Figure 4-2: Dynamic light scattering of **4a** at 10 µM concentration at 298 K in NEt_3. Data recorded on a Malvern Panalytical Zetasizer Nano ZSP. The average of 4 measurements yielded an average particle size of 342 nm.

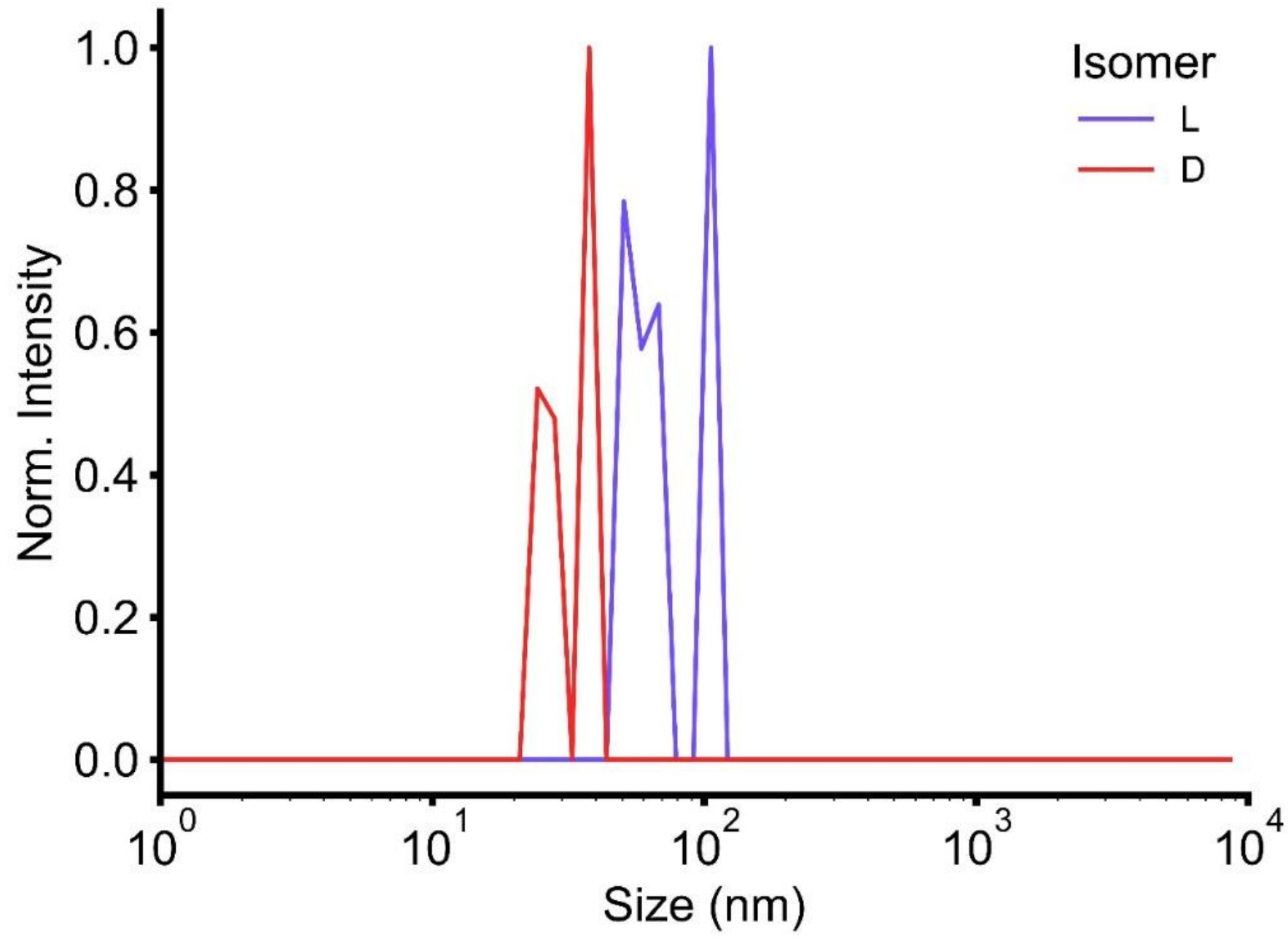

Supplementary Figure 4-3: Dynamic light scattering of **4ab** at 10 µM concentration at 318 K in MeOH. Data recorded on a Malvern Panalytical Zetasizer Nano ZSP.

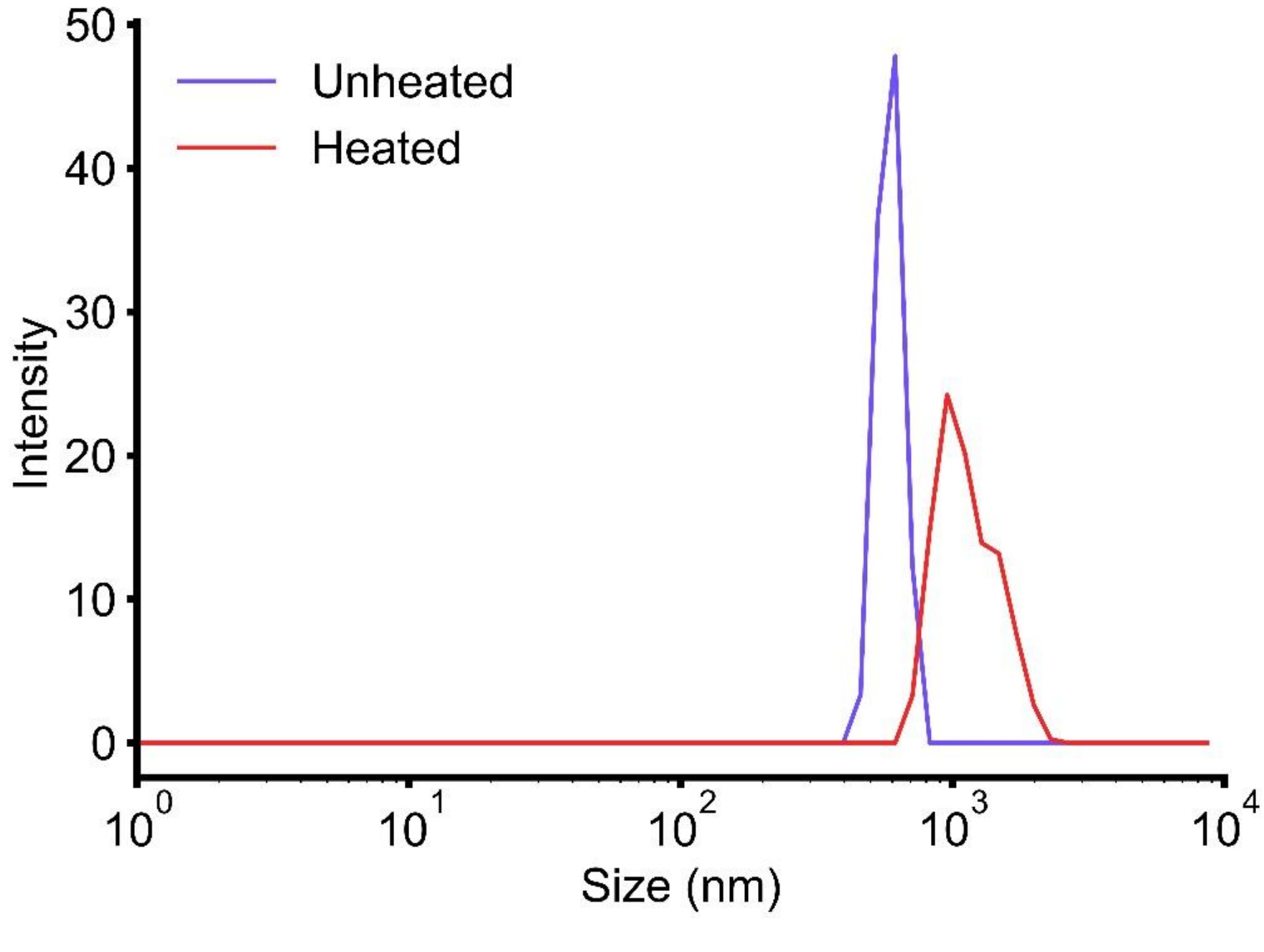

Supplementary Figure 4-4: Dynamic light scattering of **4a** at 20 µM concentration at 298 K in 9:1 NEt_3:$CHCl_3$. Data recorded on a Malvern Panalytical Zetasizer Nano ZSP. The average of 4 measurements yielded an average particle size of 597 nm pre- and an average particle size of 1162 nm post-heating.

5) Thin Film Measurements

Preparation

Thin films were prepared by dropcasting of a solution of the molecule (L- or D-isomer of **4**, or a 1:1 mixture) in $CHCl_3$ (5.83 μM) onto fused silica slides (PFS-1212, UCG Optics). The samples were then annealed by briefly placing them on a hotplate set at 160 °C, for about 30 seconds, until the colour changed. The samples were then immediately removed from the hotplate and left to cool to room temperature before measuring.

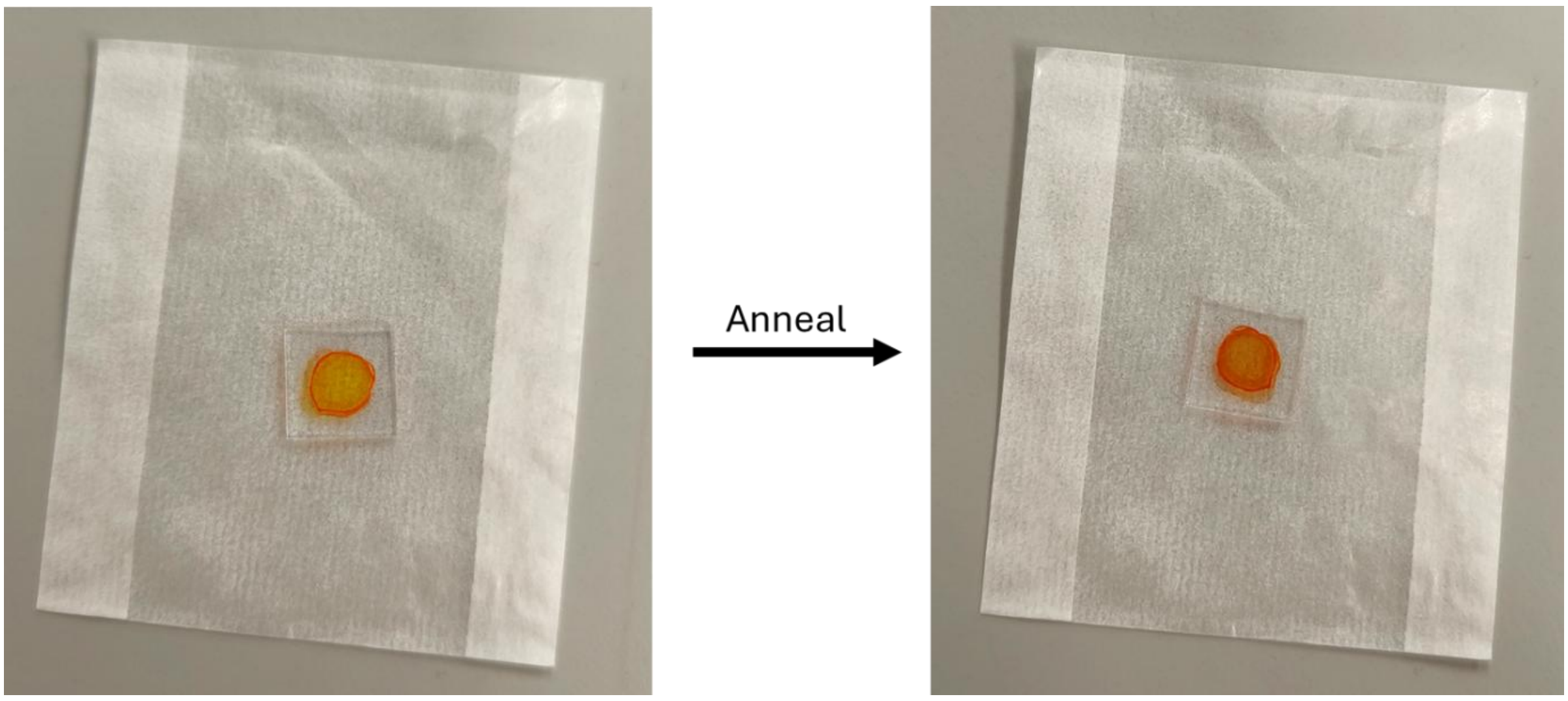

Supplementary Figure 5-1: Images of a dropcast thin film of racemic CDI **4** pre- and post-annealing. The film darkens rapidly, indicating successful annealing.

Conventional Benchtop Spectrometer Measurements

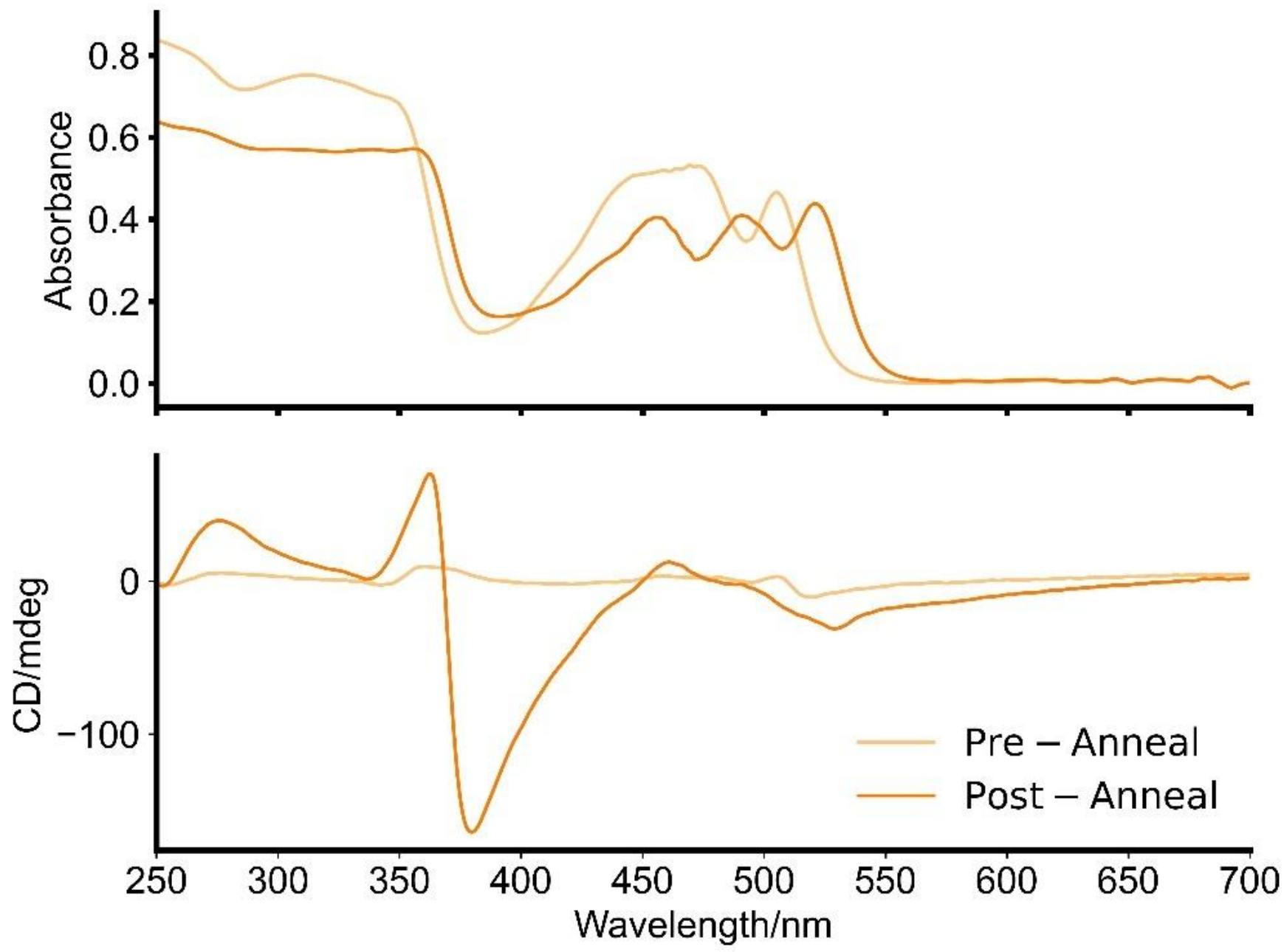

Supplementary Figure 5-2: Example of a thin film of CDI **4b** dropcast from $CHCl_3$ pre- and post-annealing, measured on a conventional benchtop circular dichroism spectrophotometer.

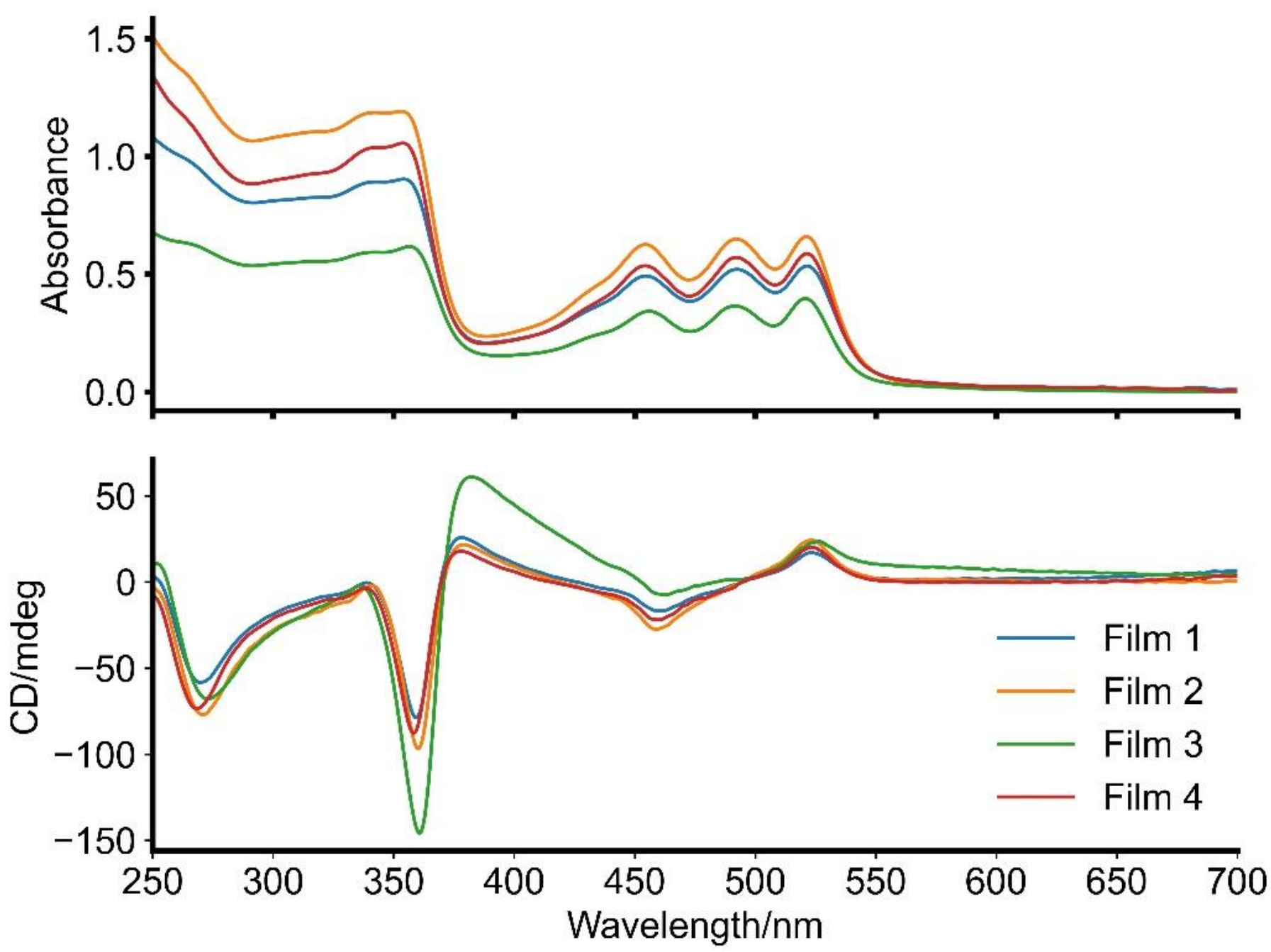

Supplementary Figure 5-3: Circular dichroism of thin films of CDI **4a** recorded on a benchtop spectrophotometer.

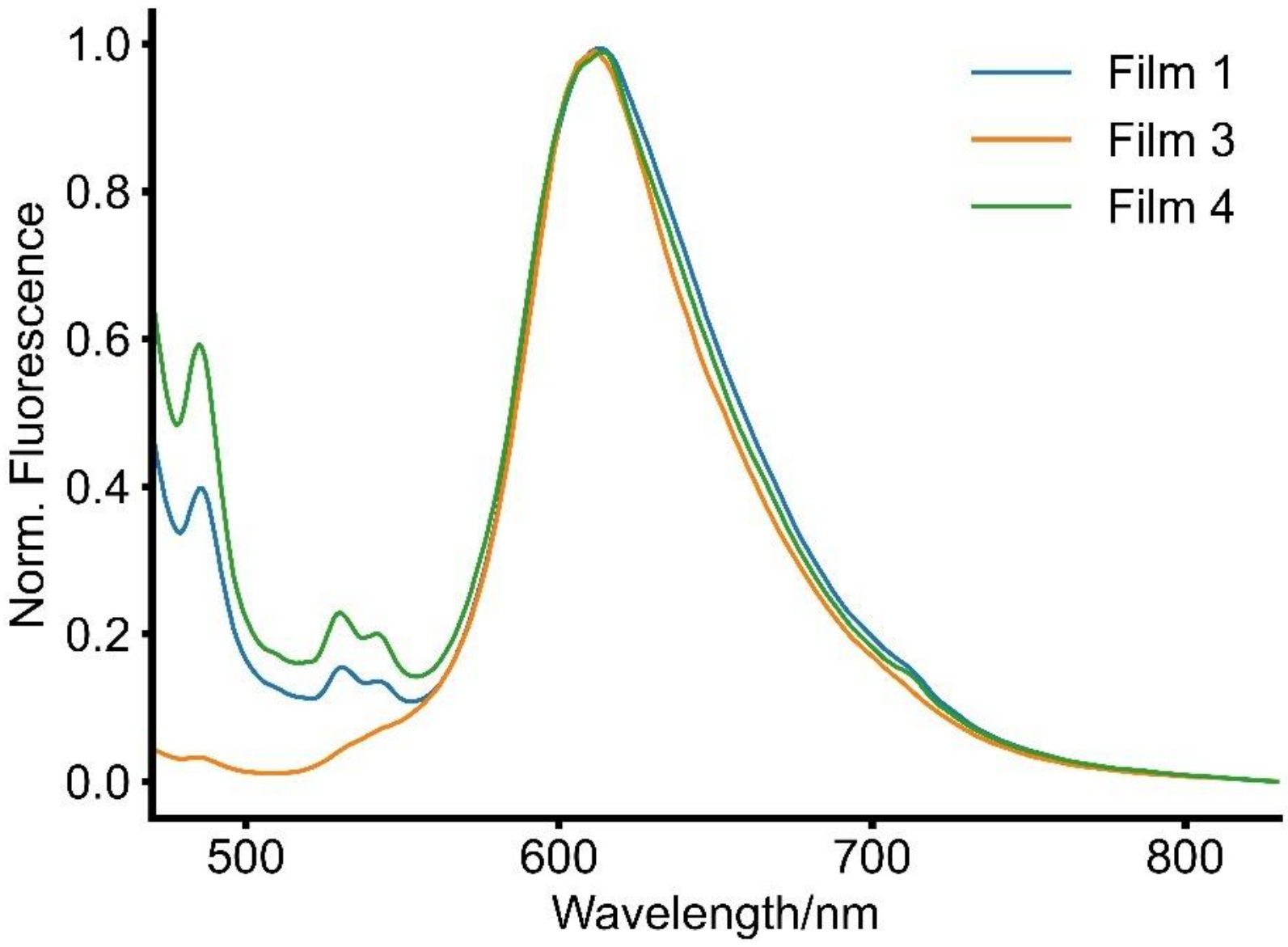

Supplementary Figure 5-4: Emission spectrum of CDI **4a** thin films recorded on a Cary eclipse. λ_{ex} = 450 nm. Gain 500 V.

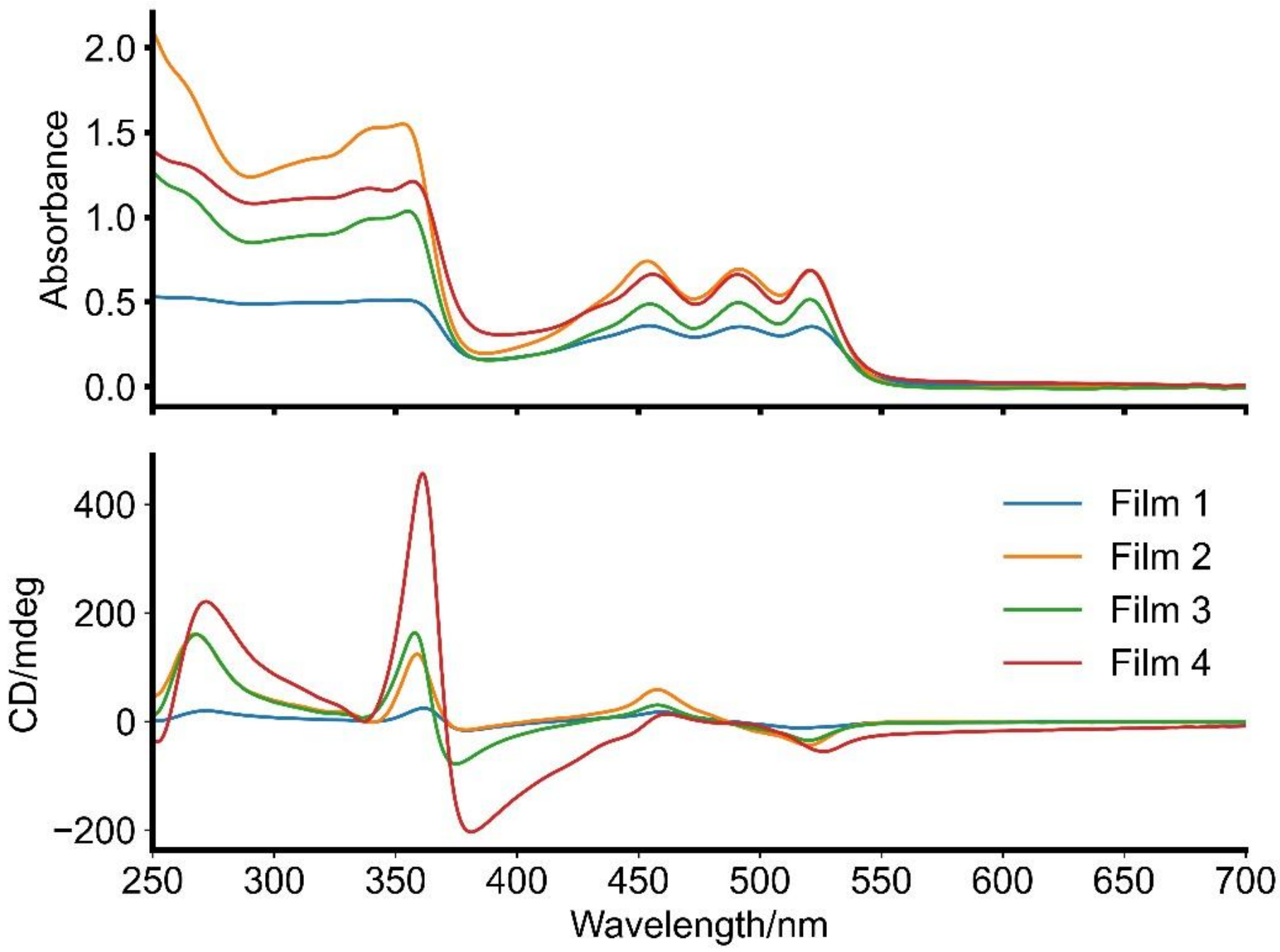

Supplementary Figure 5-5: Circular dichroism of thin films of CDI **4b** recorded on a benchtop spectrophotometer.

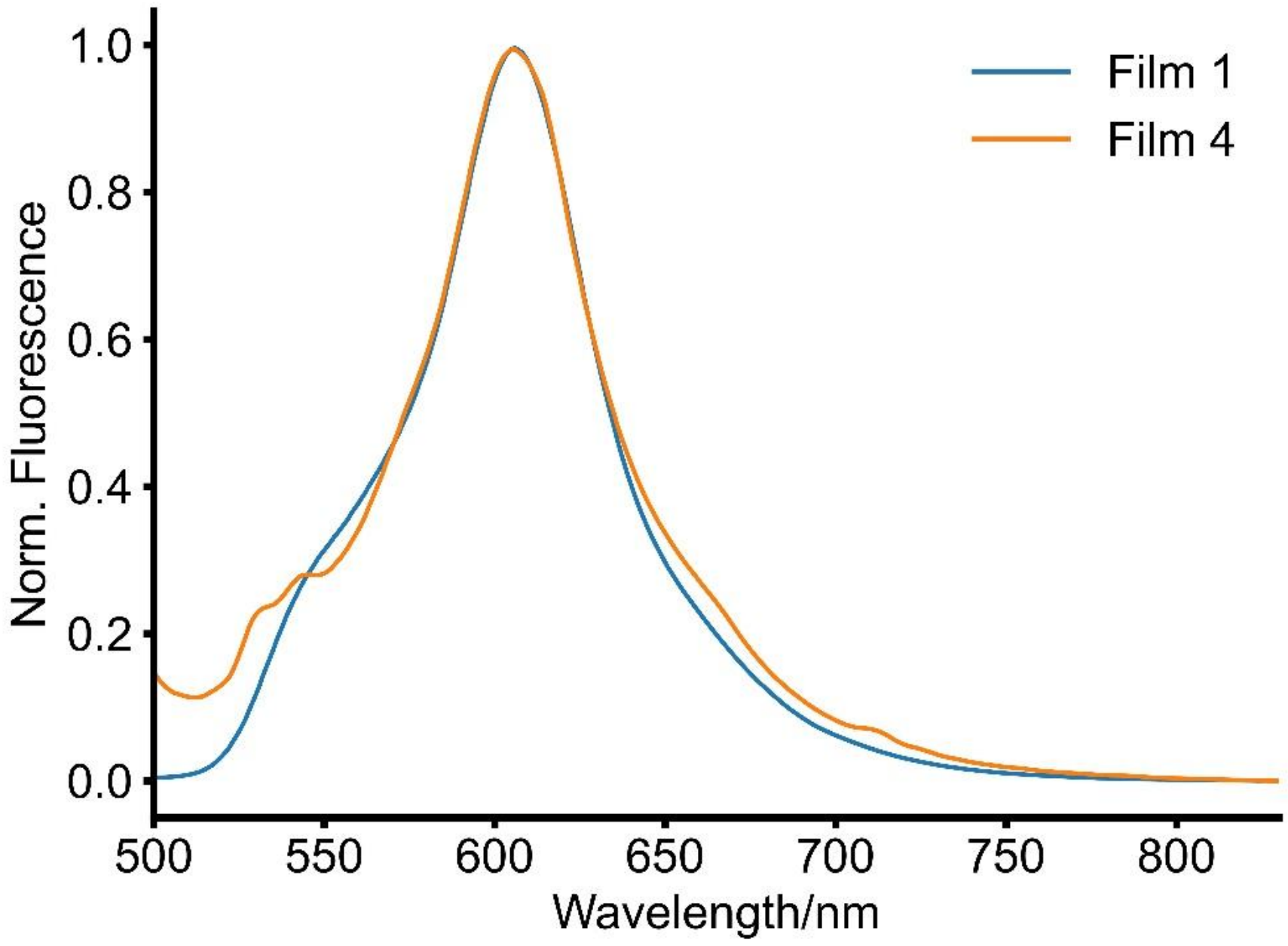

Supplementary Figure 5-6: Emission spectrum of CDI **4b** thin films recorded on a Cary eclipse. λ_{ex} = 450 nm. Gain 500 V.

Polarised Light Microscopy

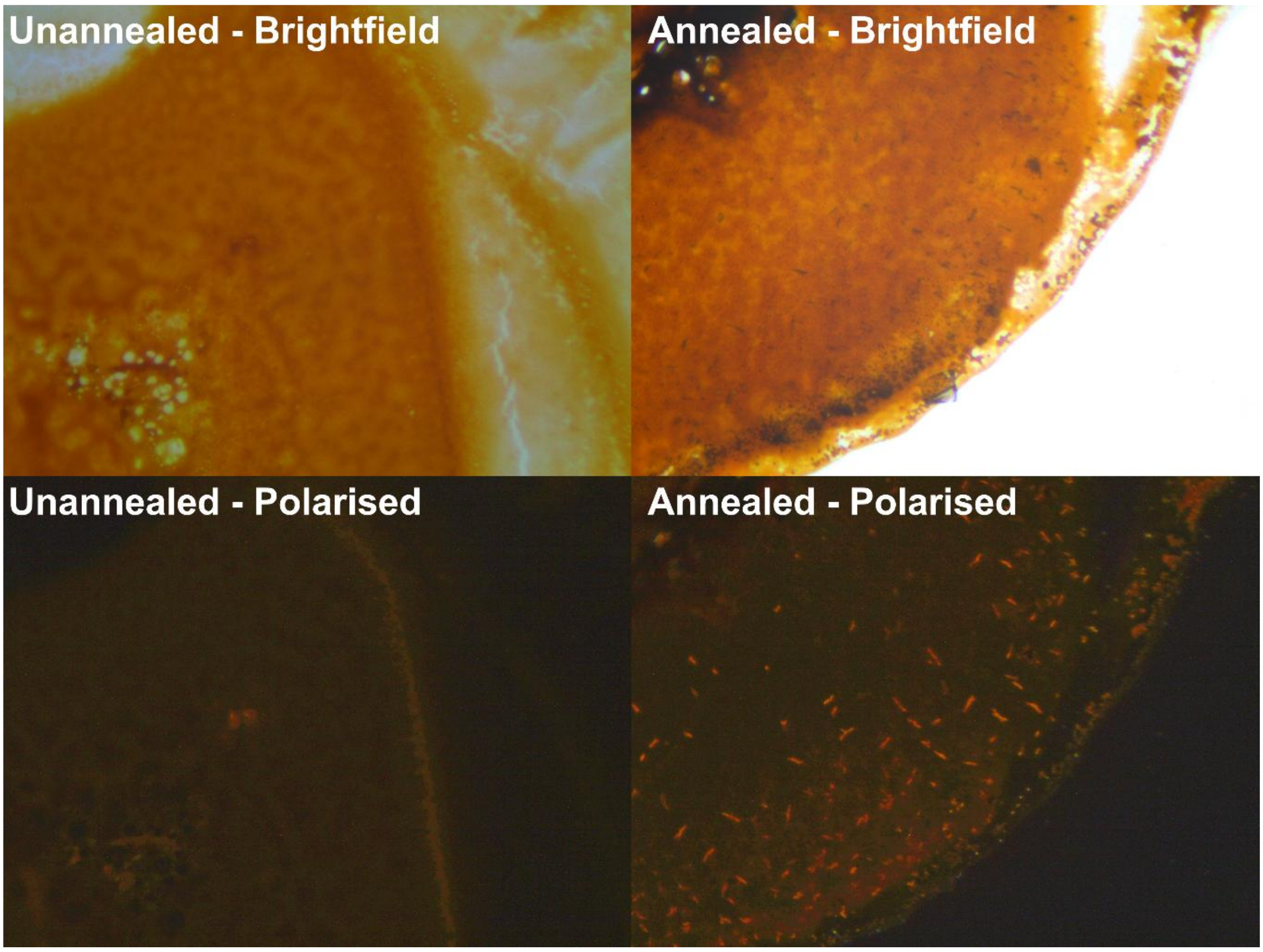

Supplementary Figure 5-7: Polarised light microscopy images of thin films of L-Val-derived CDI **4a** before and after annealing. Upon annealing, small crystallites appear, which show up strongly under cross-polarisation.

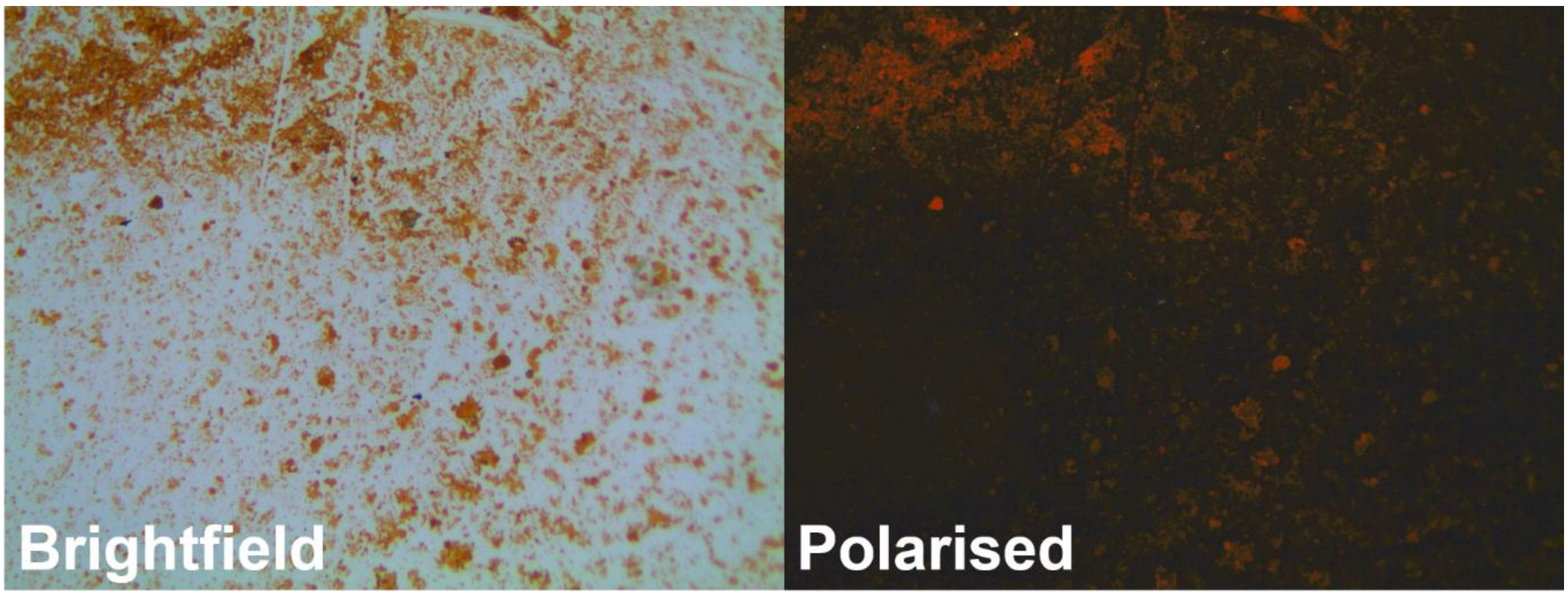

Supplementary Figure 5-8: Dropcast solutions of **4a** in NEt_3 under brightfield and cross-polarisation.

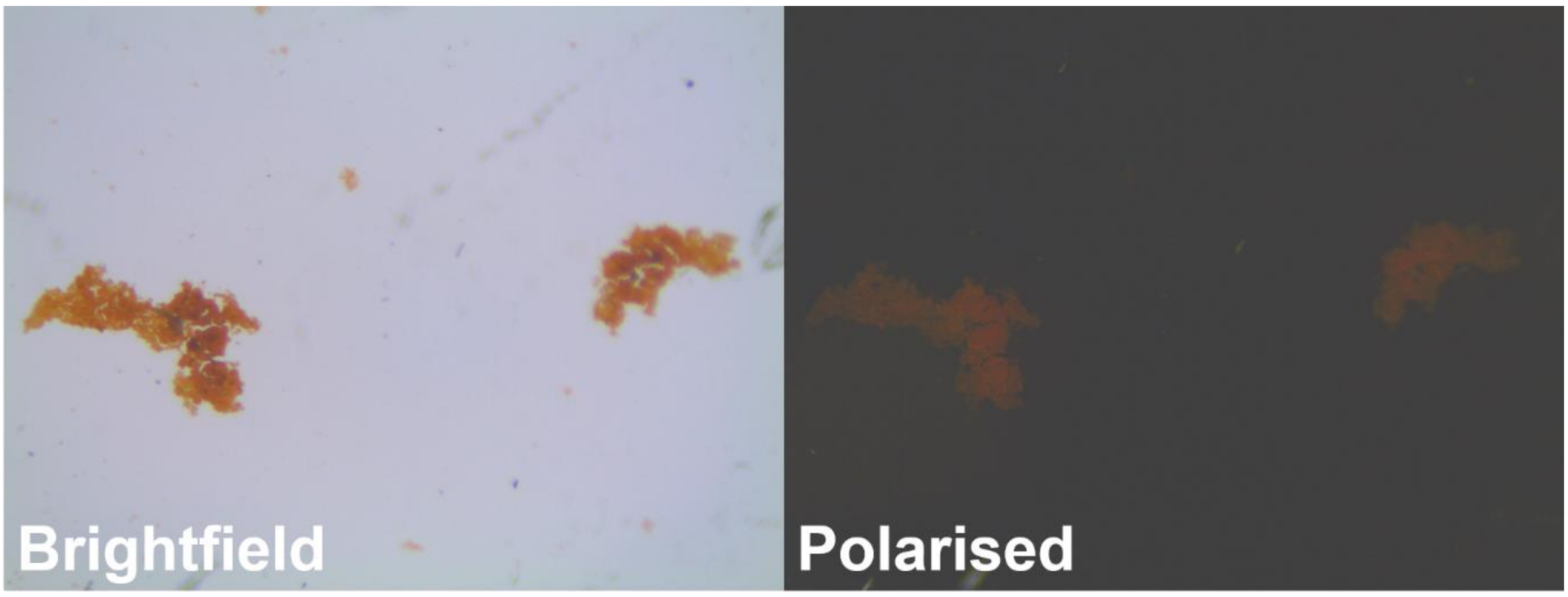

Supplementary Figure 5-9: Dropcast solutions of **4a** in MeOH under brightfield and cross-polarisation.

Height profile measurements of films

Film heights for films measured by MMP were determined on a KLA Filmetrics Profilm 3D optical profilometer and heights plotted represent a horizontal cross-section across the measured area.

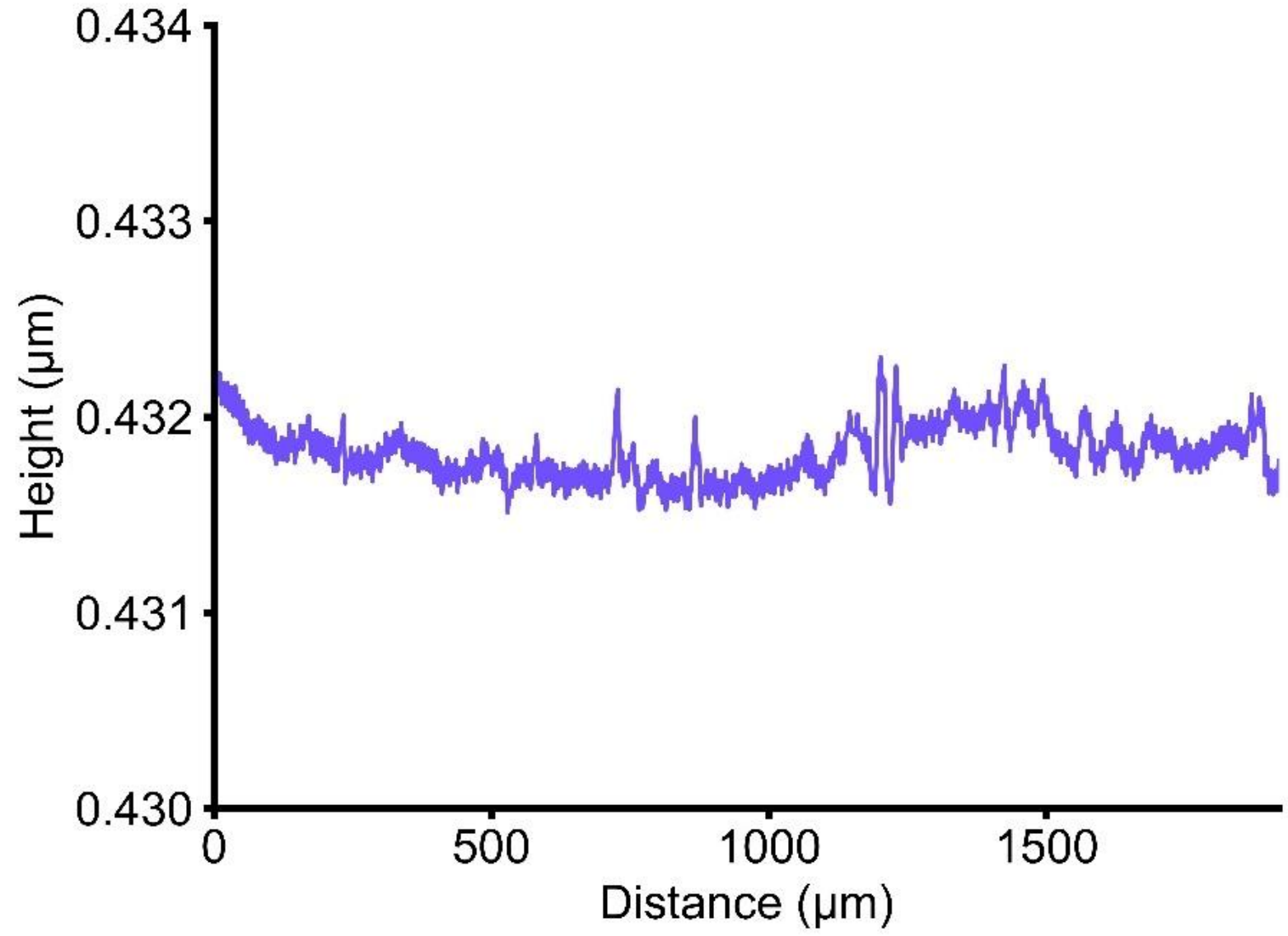

Supplementary Figure 5-10: Thickness of centre of Film 3 (**Supp. Fig. 5-3**) of LVal-derived CDI **4a.**

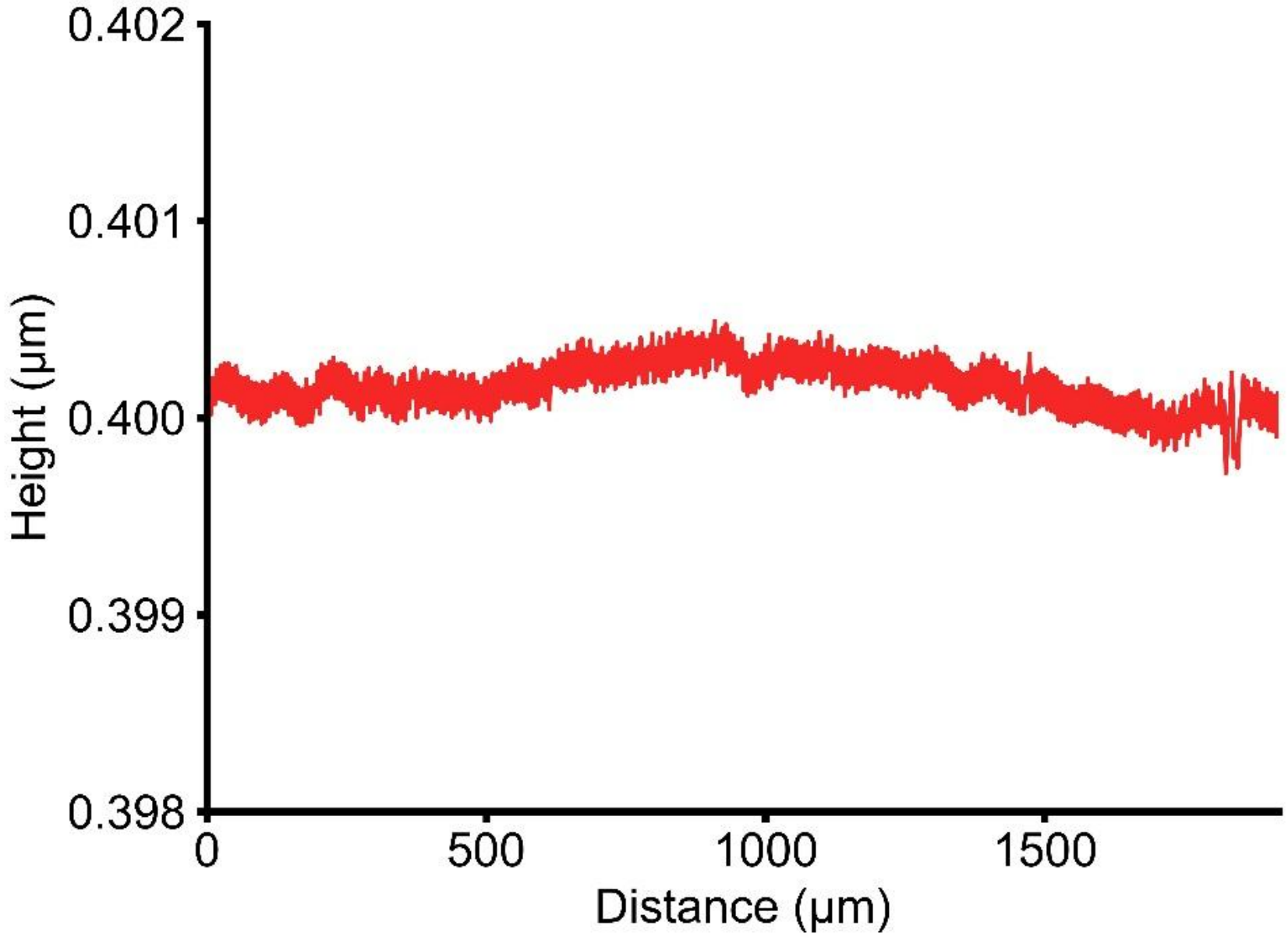

Supplementary Figure 5-11: Thickness of centre of Film 4 (**Supp. Fig. 5-5**) of DVal-derived CDI **4b.**

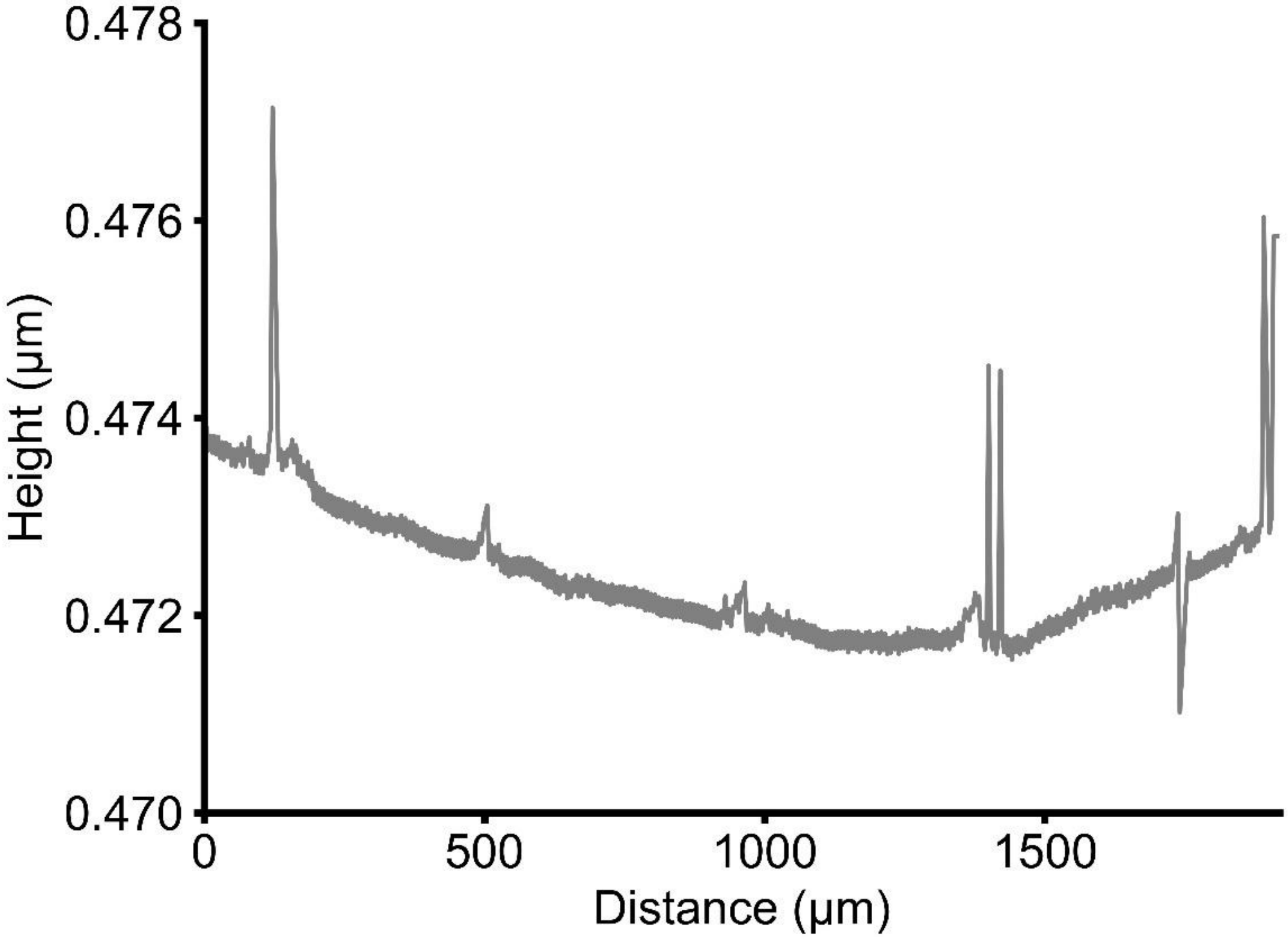

Supplementary Figure 5-12: Thickness of racemic CDI film.

Mueller Matrix polarimetry measurements at B23 at Diamond Light Source
For anisotropic samples such as thin films, linear dichroism (LD), linear birefringence (LB) and circular birefringence (CB) can substantially affect the spectral shape, magnitude, intensity and sign of the circular dichroism (CD) measured with benchtop and synchrotron CD instruments. Also, the residual static strain birefringence of the photoelastic modulator and polarizing prism can generate CD artifacts. For accurate chiroptical characterisation of solid-state chiral materials Mueller Matrix polarity (MMP) has to be employed, as each optical property can be decomposed from the others.[7,8]

The custom-built B23 MMP by Hinds Instruments is composed of four photoelastic modulators in transmission operating at different frequencies, two in the polarization state generator and two in the polarization state analyser[9,10] coupled to a double grating subtractive monochromator (Olis Instruments).[11] The wavelength range of B23 MMP is 190-700nm, which has been successfully employed to measure and quantify the CD of a wide range of chiral materials in the solid-state.[12–17] With benchtop CD instruments, the smallest area that can be measured is about 3 mm in diameter giving an average CD compared to 50 µm using the MMP instrument at B23.[9]

An important measurement derived from MMP is the depolarization index[18] (DI) that varies from 1 for non-depolarising materials to zero for depolarising materials. For non-depolarizing materials, the natural logarithm Matrix[19] method is capable of decomposing the polarization elements CD, LD, LD', CB, LB and LB'. For materials with a depolarization index between 1 and 0.5, the Analytic Inversion method[20] can decompose the polarization properties.

The Analytic Inversion is the method used in this study, enabling the CD to be decomposed from the other optical polarizations. Based on the type of materials investigated, some areas or spectral regions may show $DI > 1$ (due to e.g., light scattering, iridescence and reflections), in which case our interpretation of the data is only carried out in the areas of spectral regions with $0.5 \leq DI \leq 1$, since only these will have a physical meaning. A $DI < 0.5$ is indicative of complex materials for which none of the current methods can decompose their optical properties.

The 16 elements of the 4 × 4 Mueller Matrix (spectroscopic and mapping data) are presented for each sample. All datasets were processed with the Analytic Inversion method (for DI between 0.5 and 1) calculating the CD, CB, LD, LD', LB, and LB' optical properties and presented together with the absorbance (Abs), the dissymmetry factor ($g_{abs} = \Delta A/A$), and the Depolarization Index (DI) to allow for comparison between different samples. The substrate background has been subtracted for all MMP data sets.

MMP spectra were recorded with over a wavelength range from 300 to 600 nm. For each sample, 3 spectra at different locations were taken and averaged to produce representative spectra of the whole sample. MMP mapping was performed at $\lambda = 358$ nm, chosen as this is the maximum observed by CD for the two enantiomer films and are mirror images. Films to be measured were chosen based on the highest CD observed by conventional benchtop spectrometers (**Supp. Figs. 5-3 and 5-5**). This means that for **4a**, film 3 was measured, whereas for **4b**, film 4 was measured. Spectra were taken with an integration time of 0.1 seconds over 16 iterations, whereas maps were taken with an integration time of 0.05 seconds over 16 iterations.

M_{00} T	M_{01} -LD	M_{02} -LD’	M_{03} CD
M_{10} -LD	M_{11} T	M_{12} CB	M_{13} LB’
M_{20} -LD’	M_{21} -CB	M_{22} T	M_{23} -LB
M_{30} CD	M_{31} -LB’	M_{32} LB	M_{33} T

Supplementary Figure 5-13: Optical properties assigned to the 16 elements of the 4x4 Mueller matrix (MM). The principal diagonal represents the Transmission of the investigated thin film. The green elements CD, LD (along 0° and 90°axes) and LD’ (along ±45° axes) are symmetric whereas the red elements CB, LB and LB’, and blue elements -CB, -LB and -LB’ are antisymmetric.

MMP Spectra

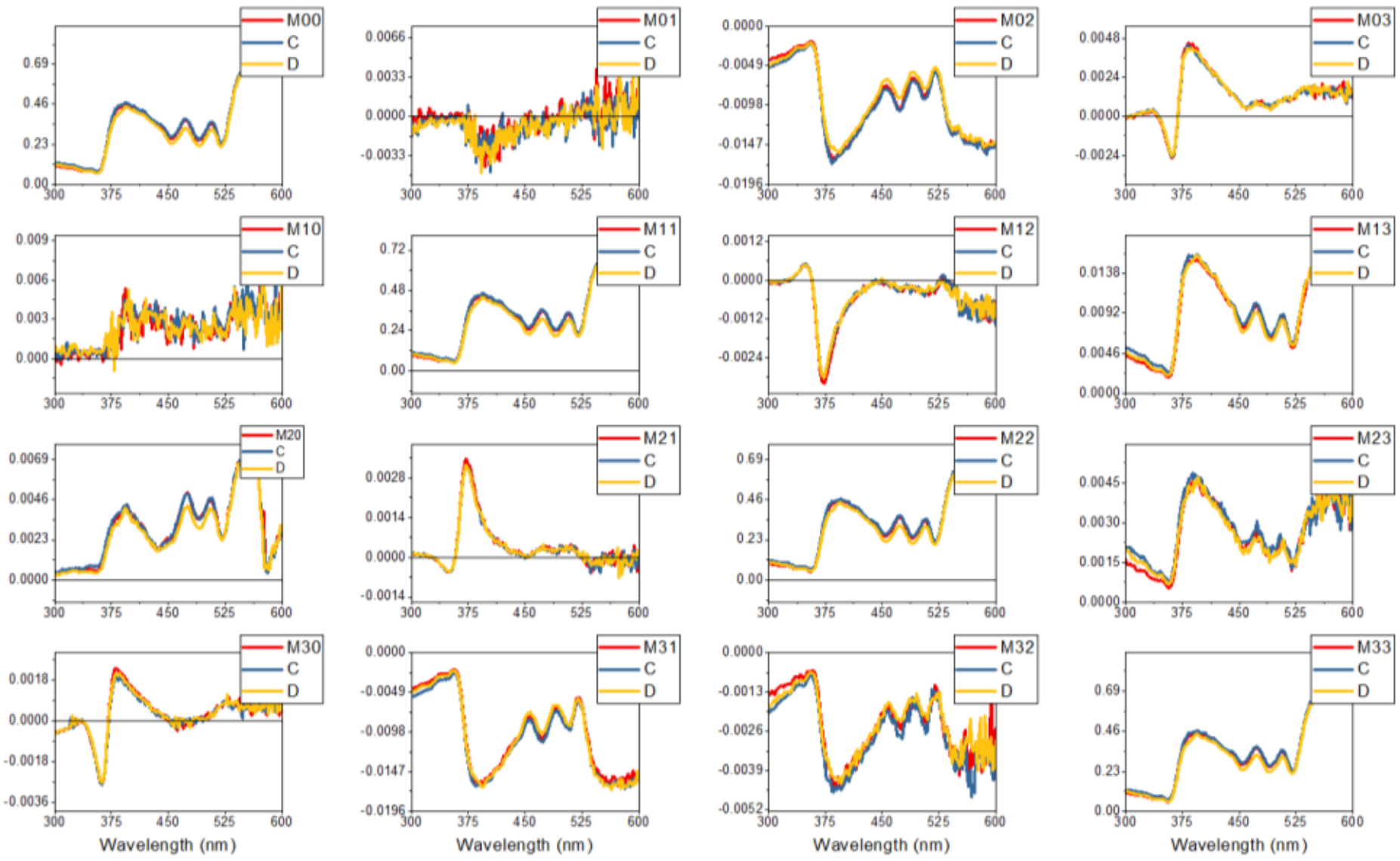

Supplementary Figure 5-14: Raw data of 4x4 Mueller-Matrix of three positions on the CDI **4a** film recorded by the MMP over the range of 300-600 nm. The LD (M10 and M01) and LD' (M20 and M02) elements are not symmetric, indicating that the film samples are bi-anisotropic.

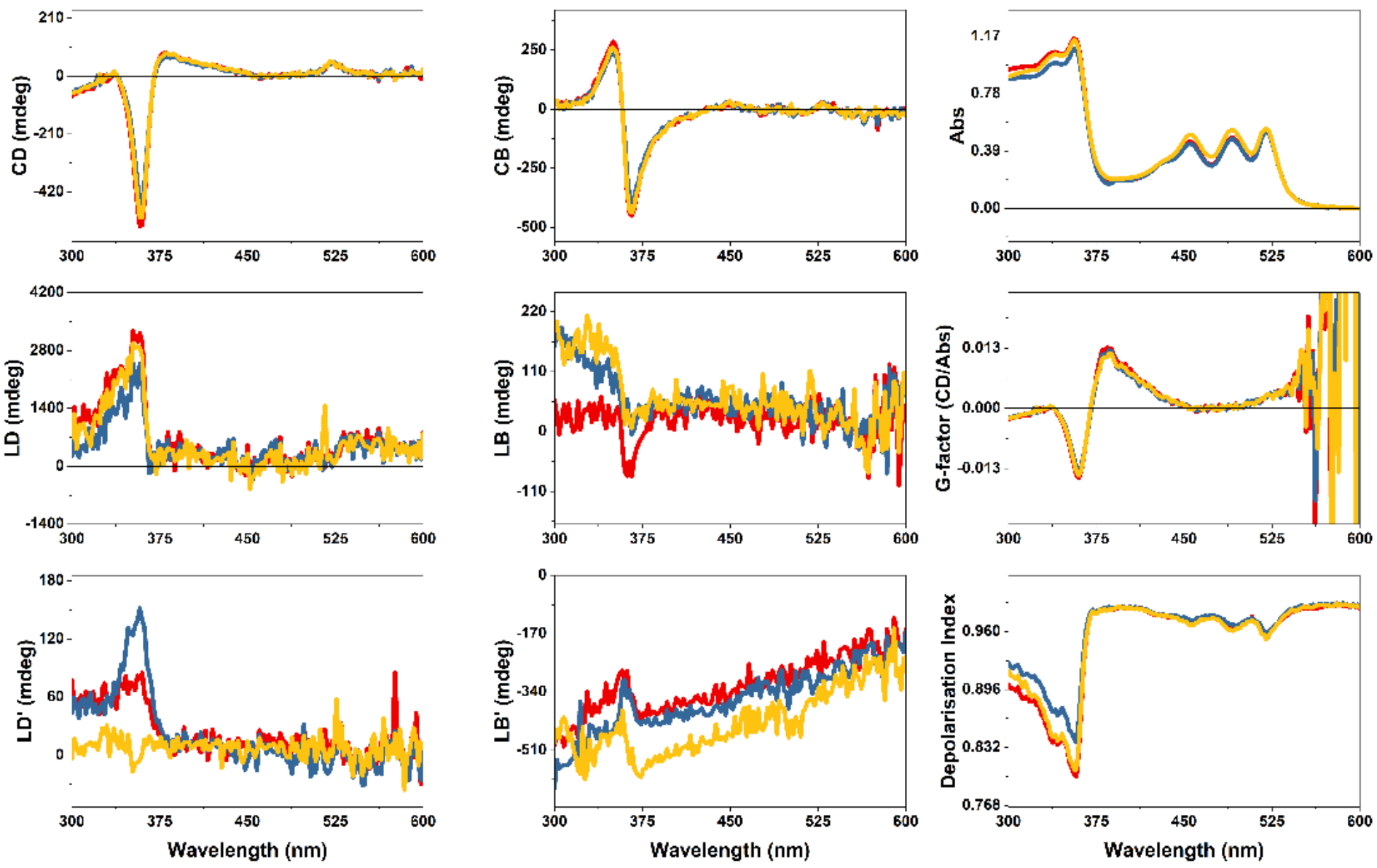

Supplementary Figure 5-15: CD, CB, Abs, LD, LB, g-factor, LD', LB' and DI spectra at 3 locations of the CDI **4a** film as recorded by MMP and processed with the Analytic Inversion method. The g_{abs} values at wavelengths > 530 nm are neglected due to noise.

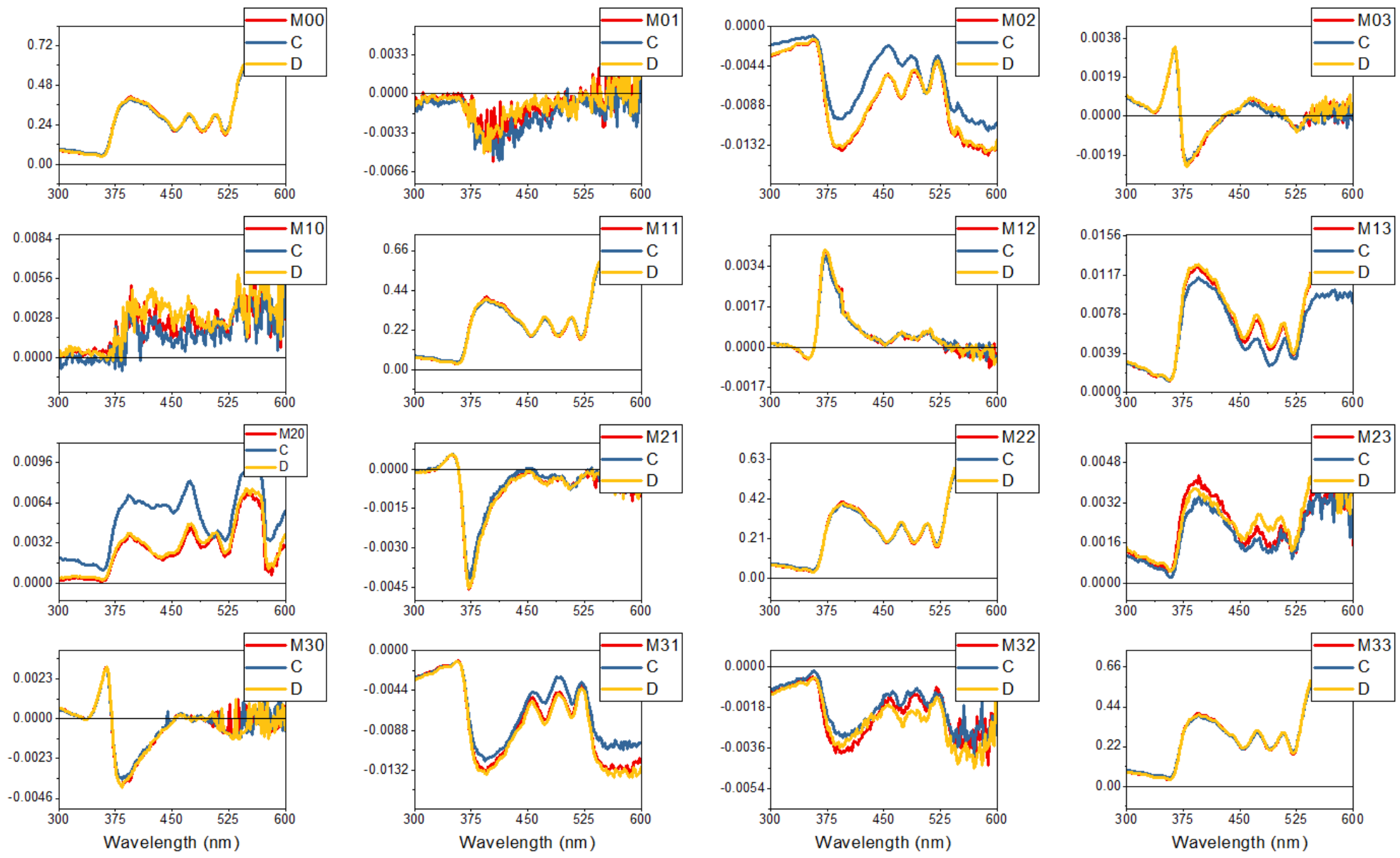

Supplementary Figure 5-16: Raw data of 4x4 Mueller-Matrix of three positions on the CDI **4b** film recorded by the MMP over the range of 300-600 nm. The LD (M10 and M01) and LD' (M20 and M02) elements are not symmetric, indicating that the film samples are bi-anisotropic.

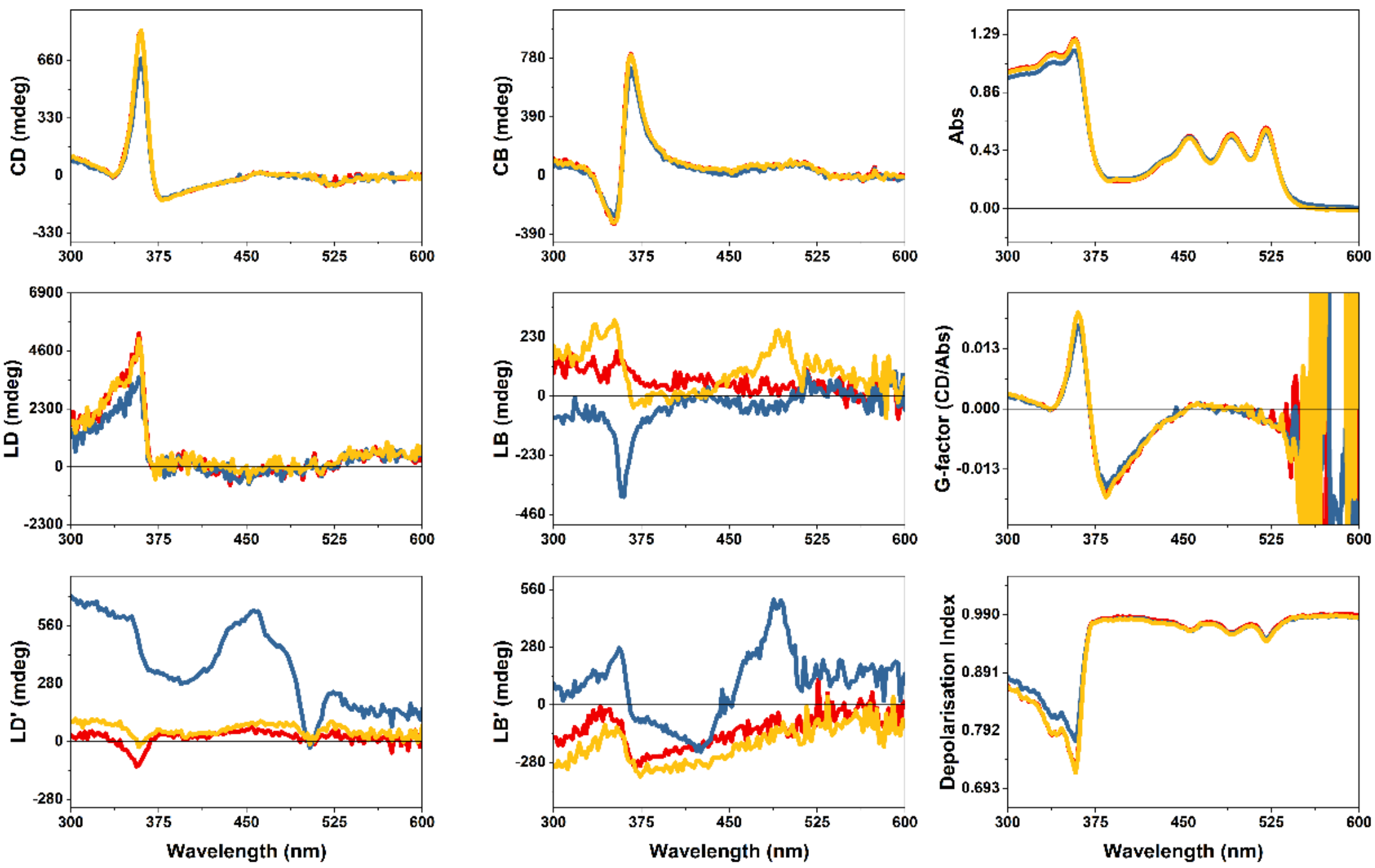

Supplementary Figure 5-17: CD, CB, Abs, LD, LB, g-factor, LD’, LB’ and DI spectra at 3 locations of the CDI **4b** film as recorded by MMP and processed with the Analytic Inversion method. The g_{abs} values at wavelengths > 530 nm are neglected due to noise.

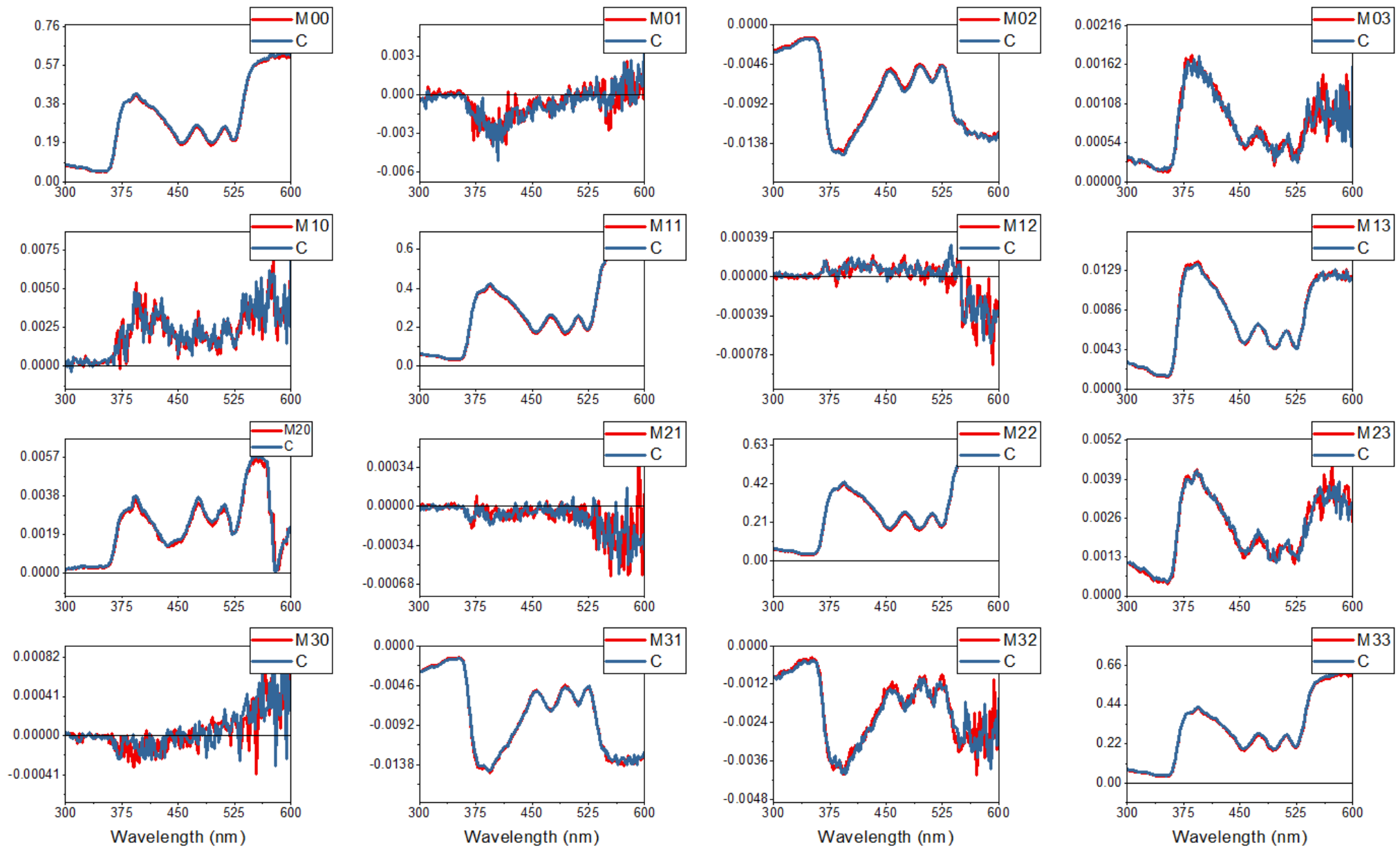

Supplementary Figure 5-18: Raw data of 4x4 Mueller-Matrix of two positions on the racemic CDI **4** film recorded by the MMP over the range of 300-600 nm. The dissymmetry of LD and LD' indicates the sample is anisotropic.

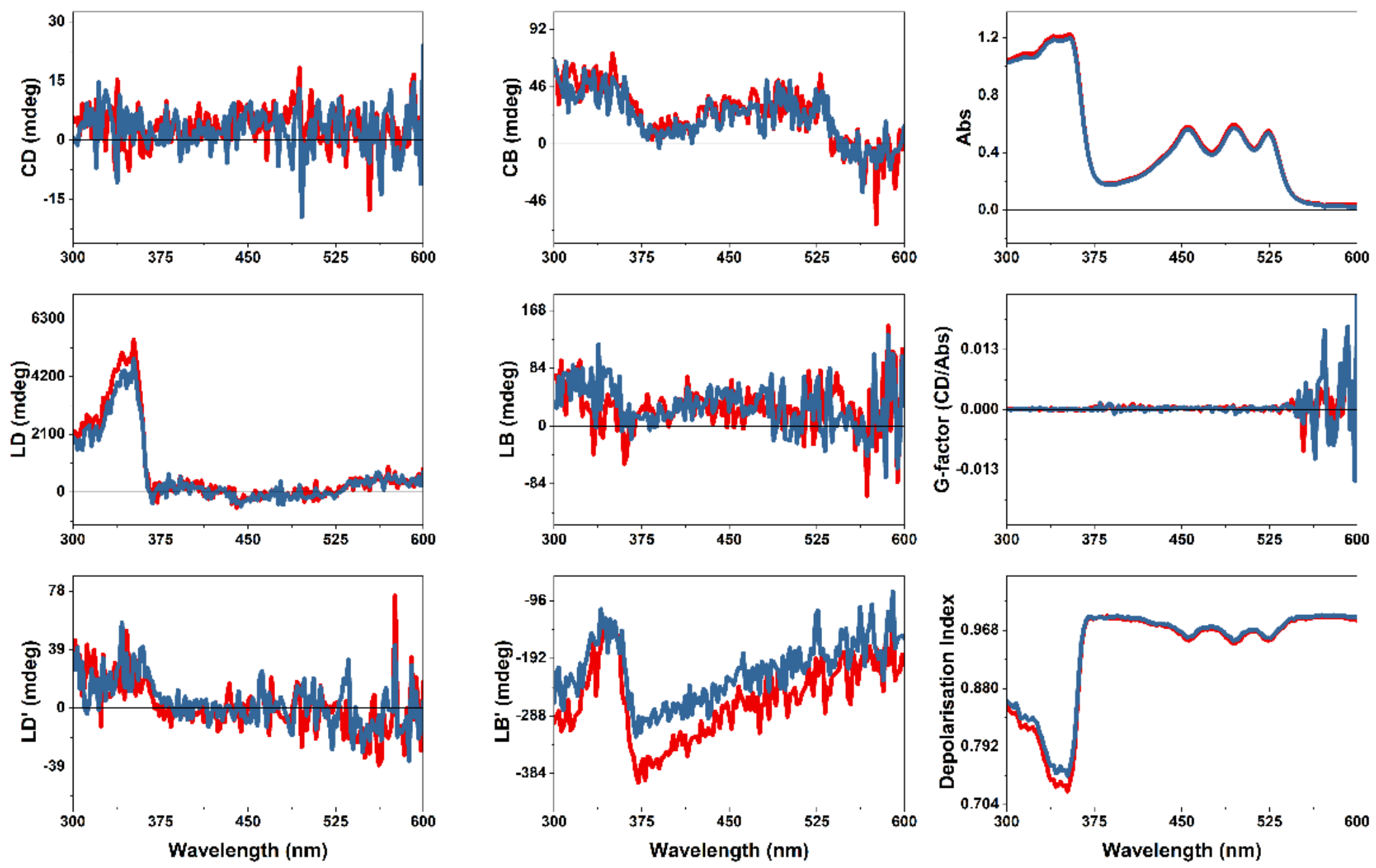

Supplementary Figure 5-19: CD, CB, Abs, LD, LB, g-factor, LD', LB' and DI spectra at 2 locations of the racemic CDI film as recorded by MMP and processed with the Analytic Inversion method.

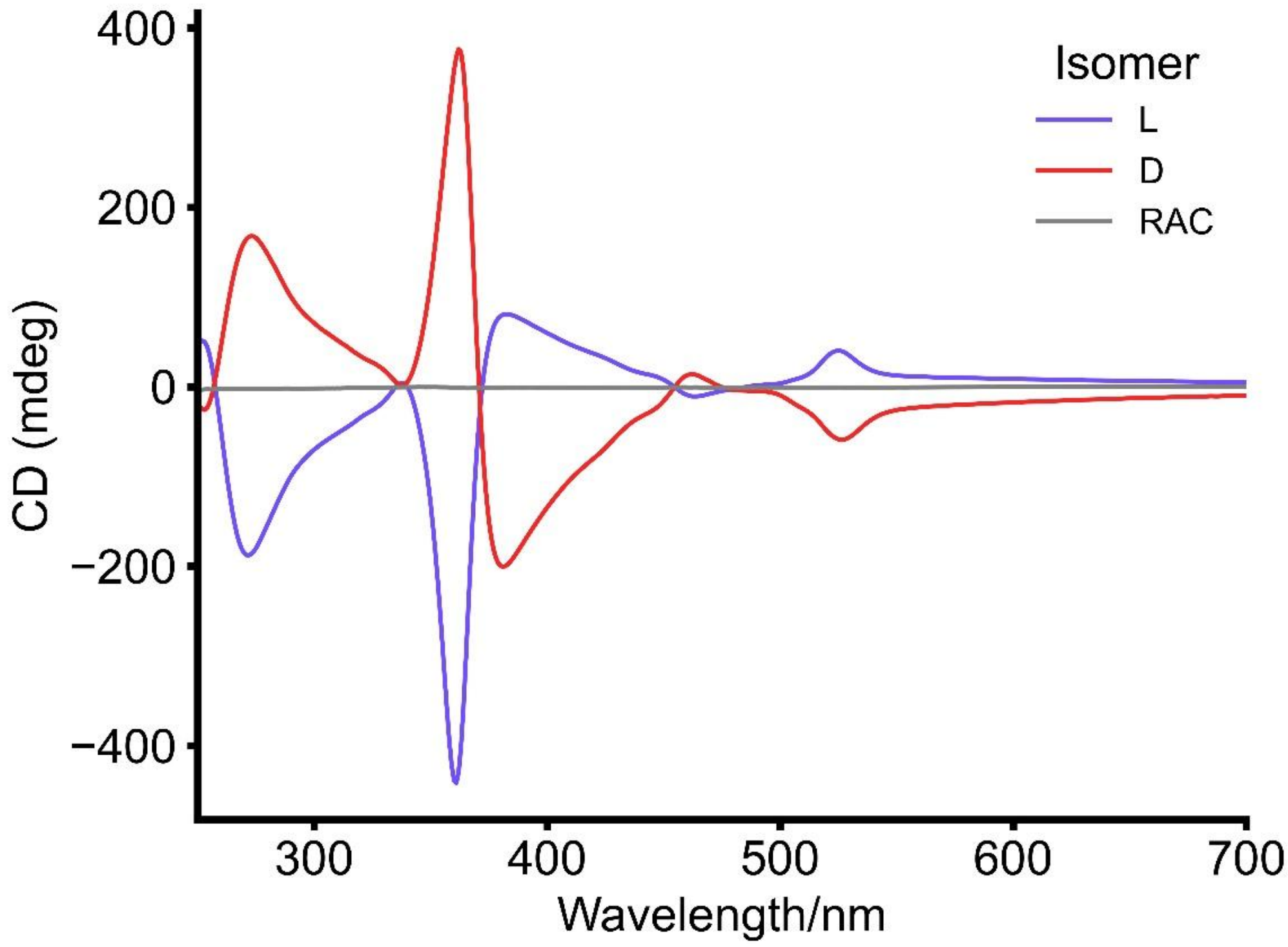

Supplementary Figure 5-20: Averaged and smoothed CD spectra of the L-, D- and racemic films (**Supp. Figs. 5-15, 5-17, 4-19**) recorded on a benchtop spectrophotometer (chirascan).

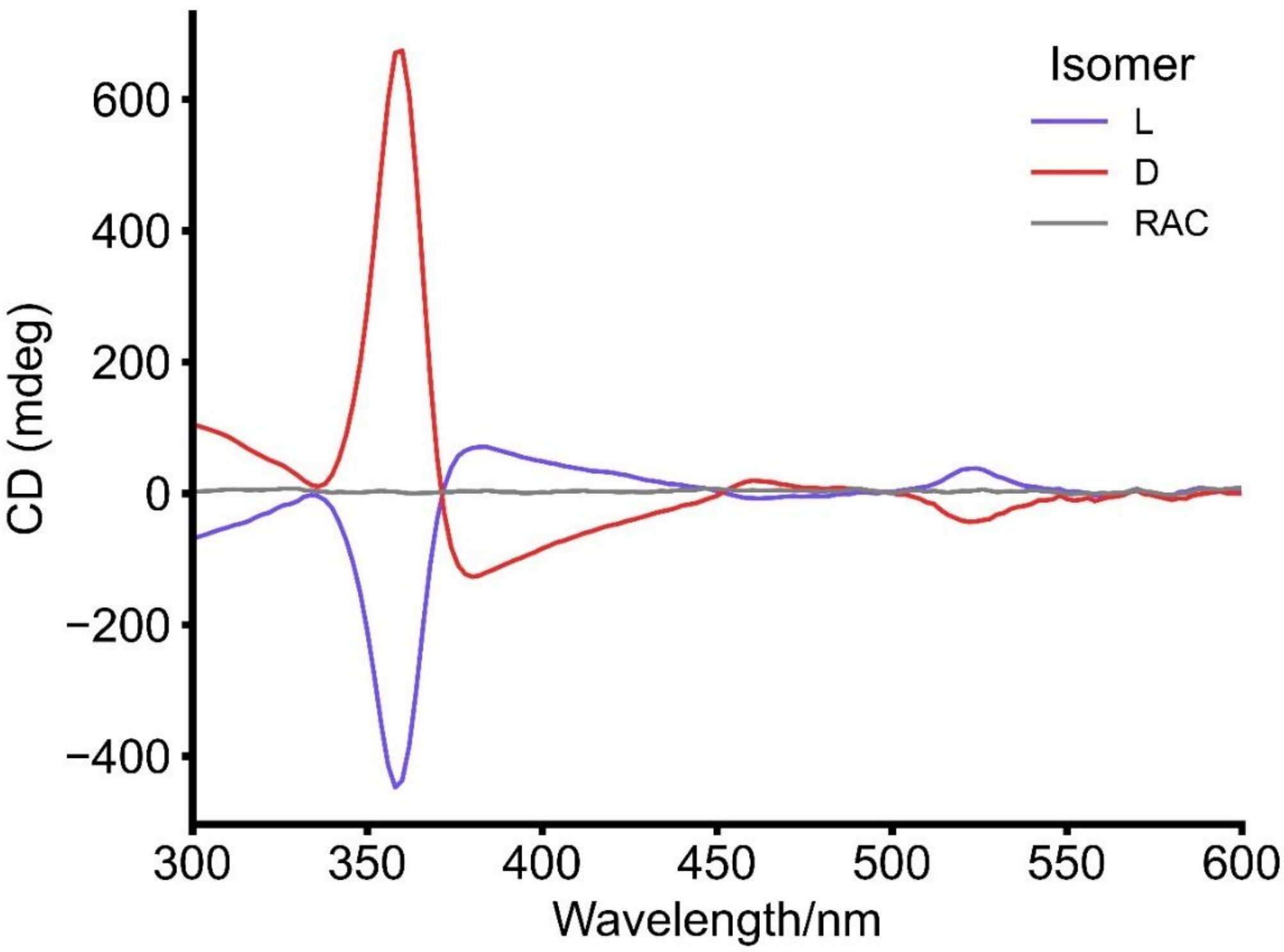

Supplementary Figure 5-21: Averaged and smoothed CD spectra of the L-, D- and racemic films (**Supp. Figs. 5-15, 5-17, 4-19**) as recorded by the MMP.

MMP Maps

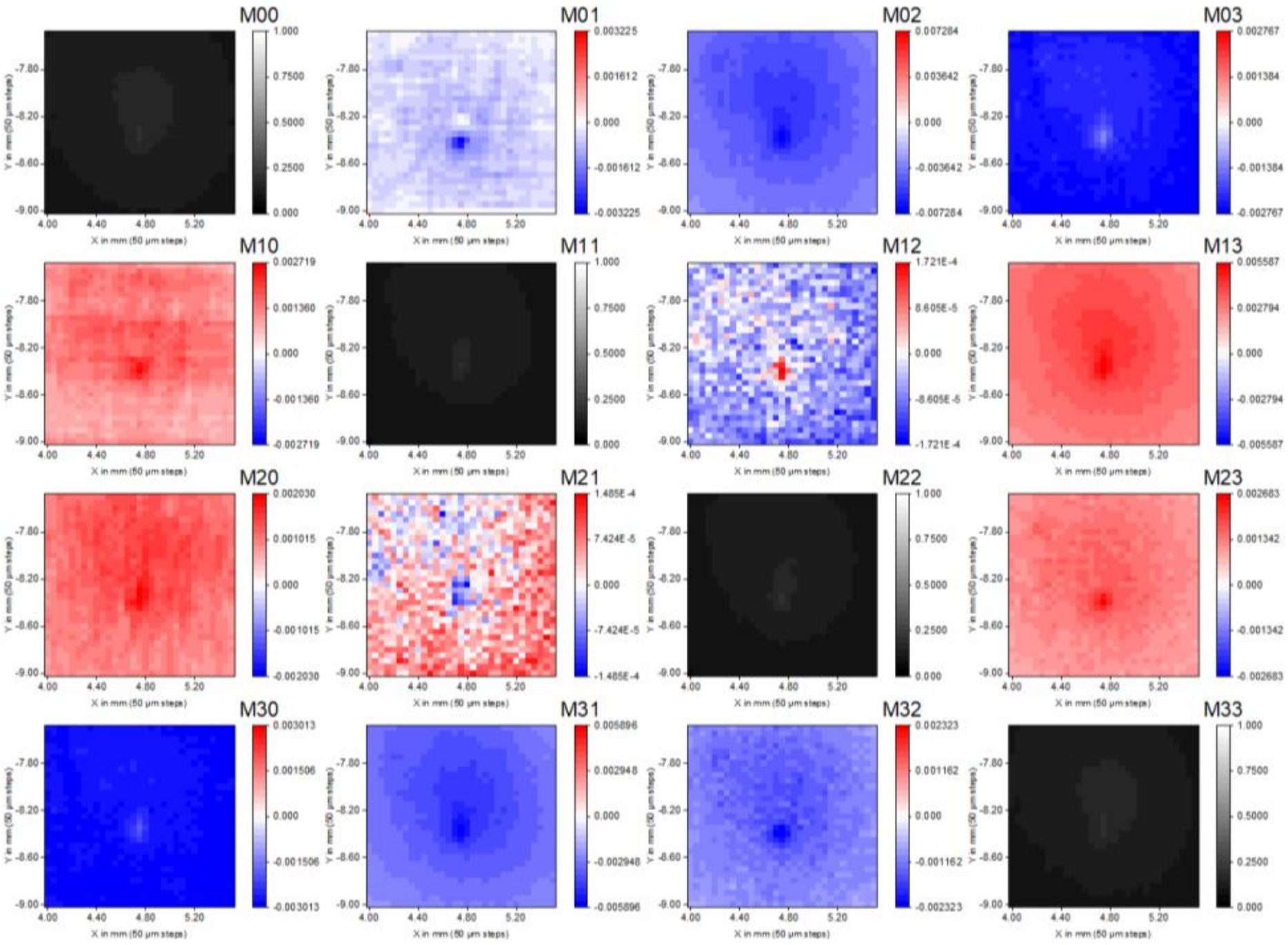

Supplementary Figure 5-22: Raw differential 4x4 Mueller-Matrix at 358 nm of CDI **4a** thin film over an area of 1.5 mm x 1.5 mm with a resolution of 50 µm (31x31 steps).

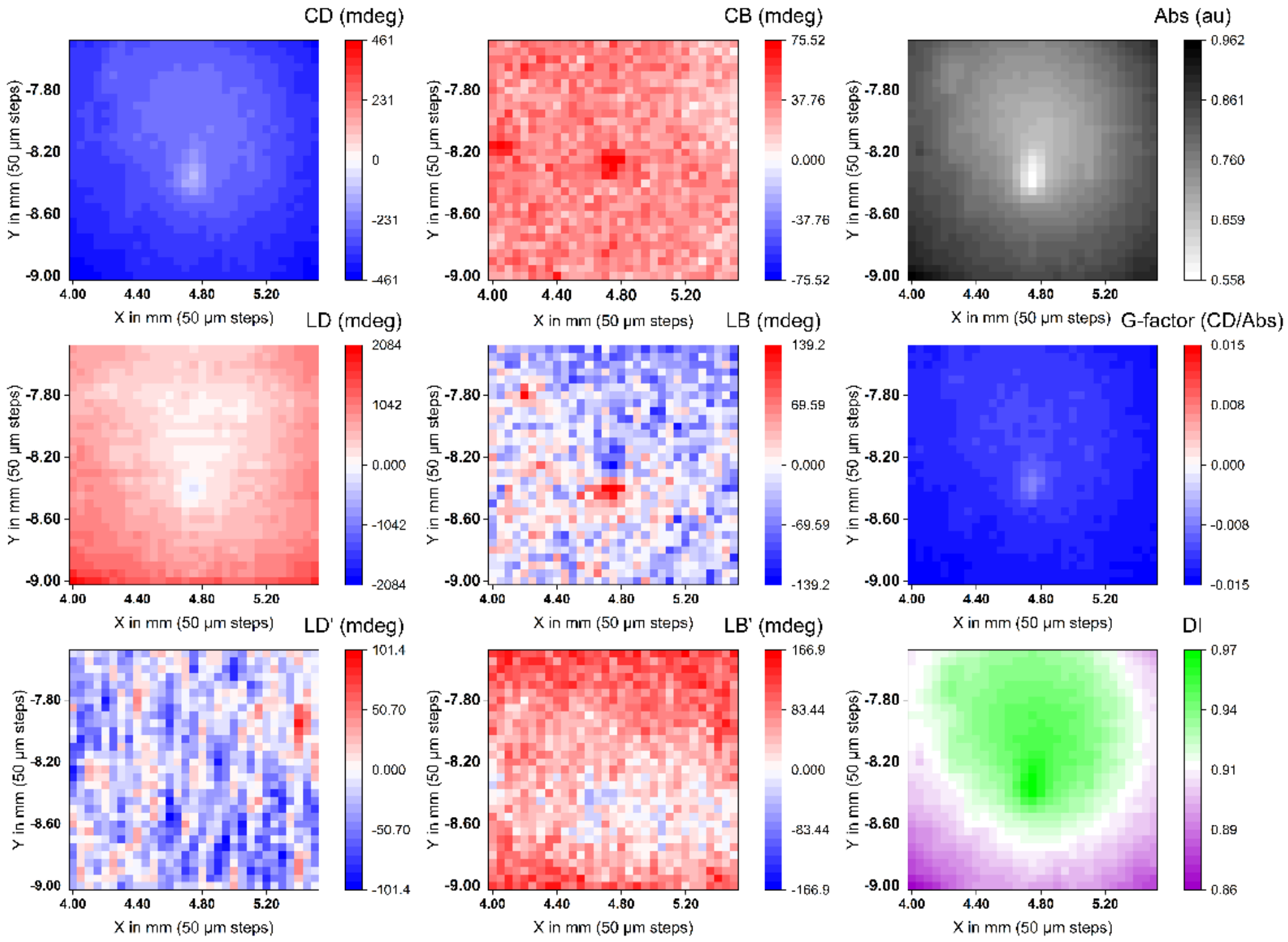

Supplementary Figure 5-23: CD, CB, Abs, LD, LB, g-factor, LD', LB' and DI Map recorded at 358 nm of CDI **4a** thin film with background subtracted and processed with the Analytic Inversion method over an area of 1.5 mm x 1.5 mm with a resolution of 50 µm (31x31 steps).

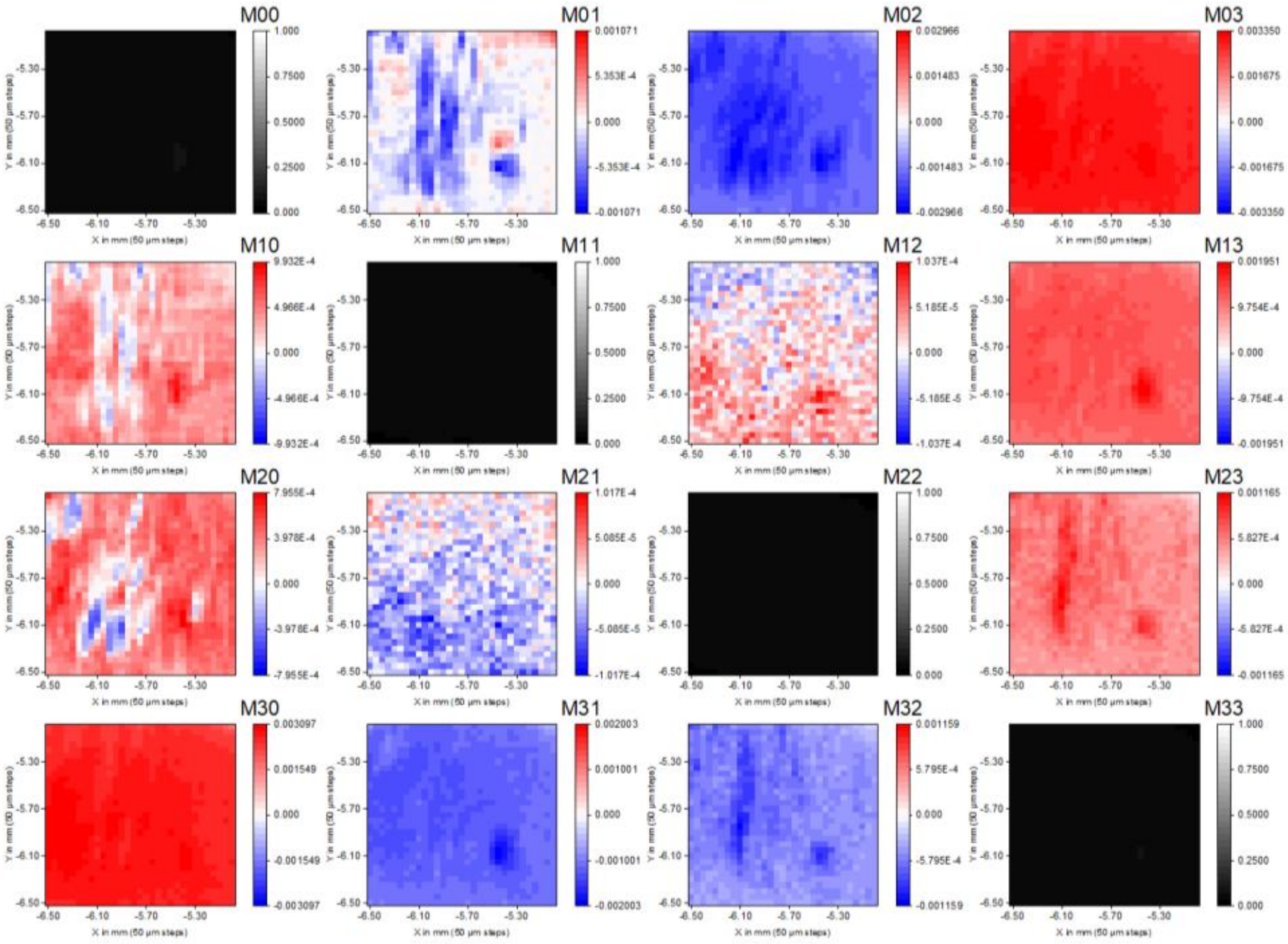

Supplementary Figure 5-24: Raw differential 4x4 Mueller-Matrix at 358 nm of CDI **4b** thin film over an area of 1.5 mm x 1.5 mm with a resolution of 50 µm (31x31 steps).

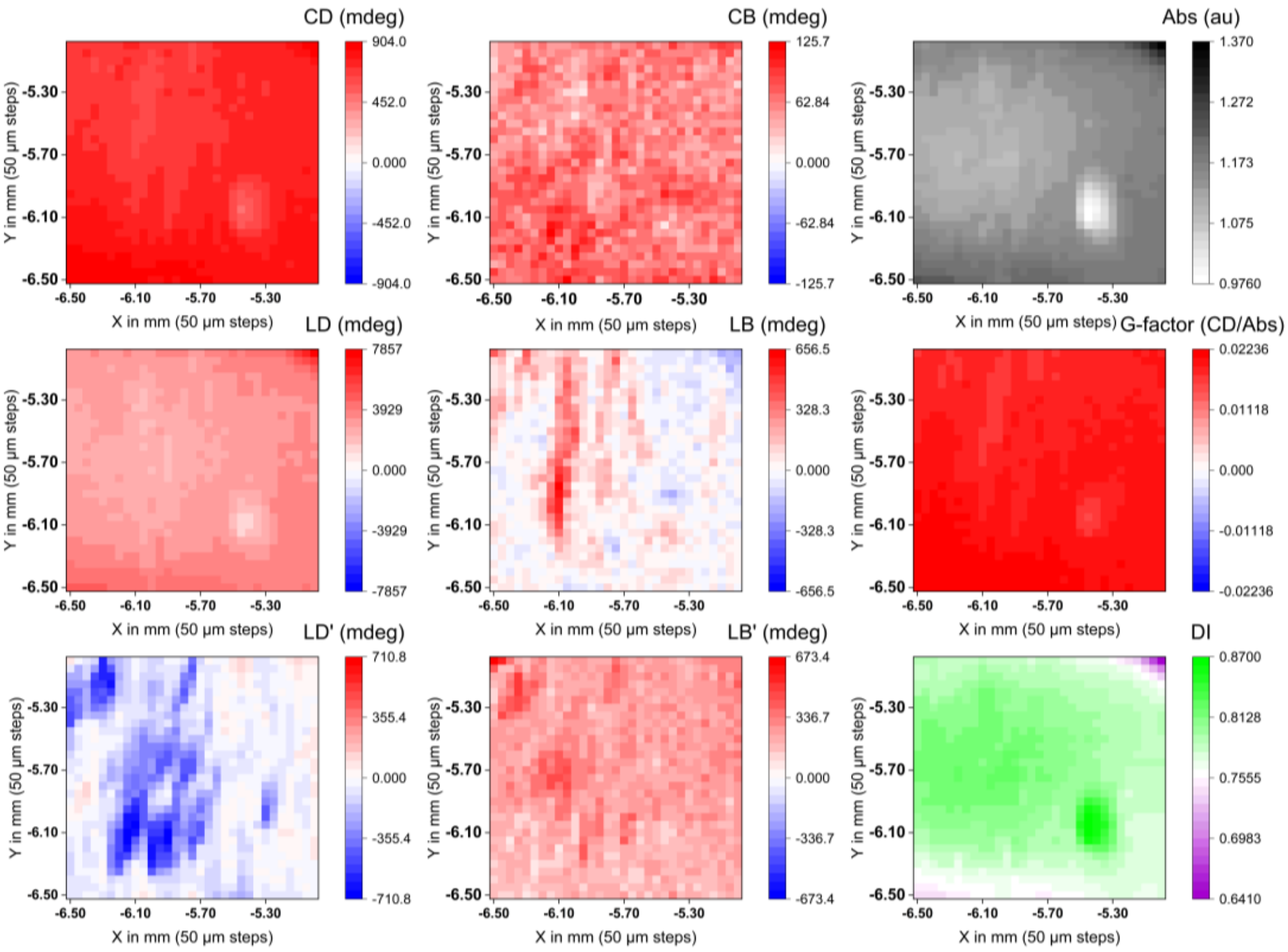

Supplementary Figure 5-25: CD, CB, Abs, LD, LB, g-factor, LD', LB' and DI Map recorded at 358 nm of CDI **4b** thin film with background subtracted and processed with the Analytic Inversion method over an area of 1.5 mm x 1.5 mm with a resolution of 50 µm (31x31 steps).

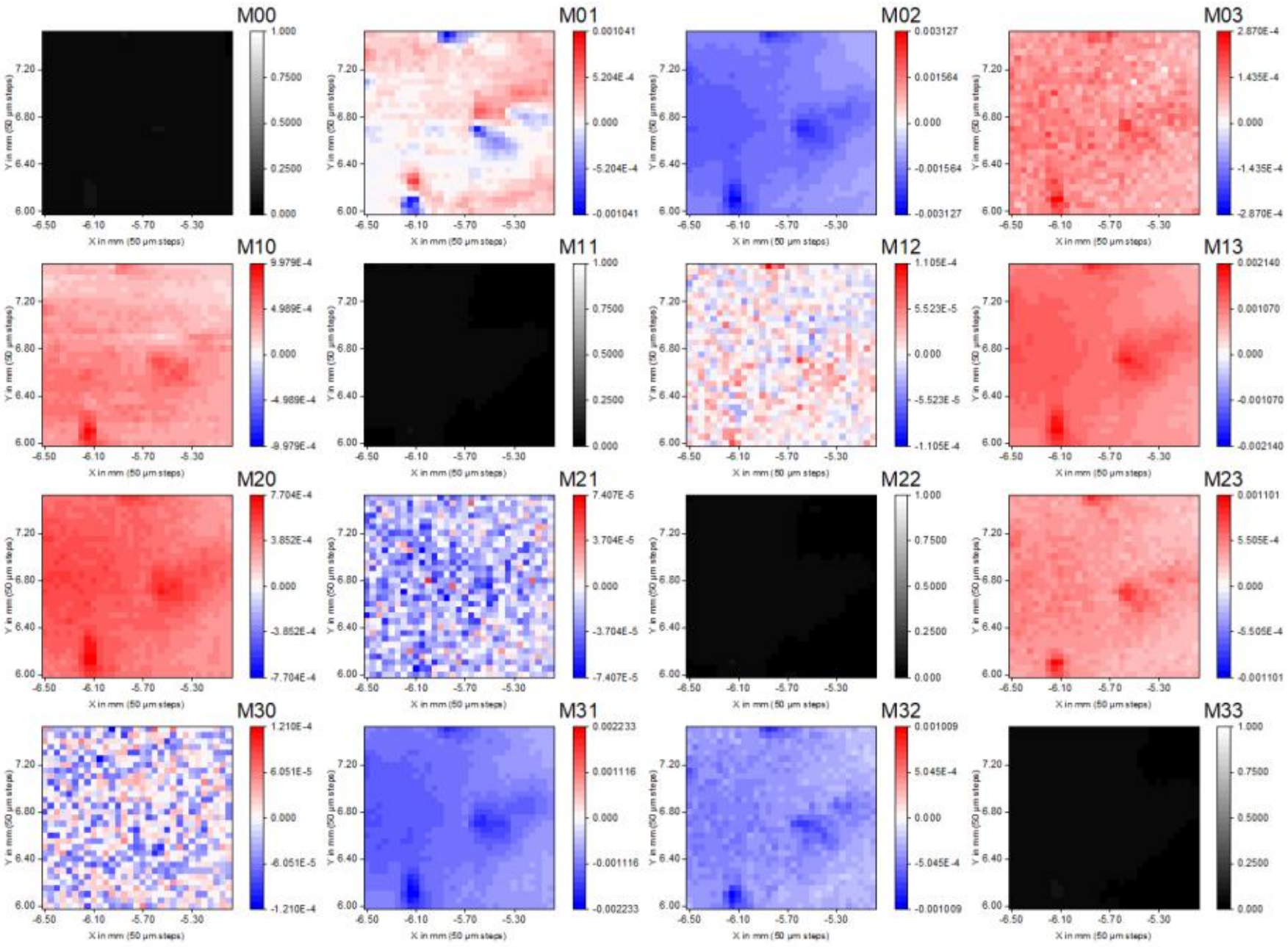

Supplementary Figure 5-26: Raw differential 4x4 Mueller-Matrix at 358 nm of racemic CDI thin film over an area of 1.5 mm x 1.5 mm with a resolution of 50 µm (31x31 steps).

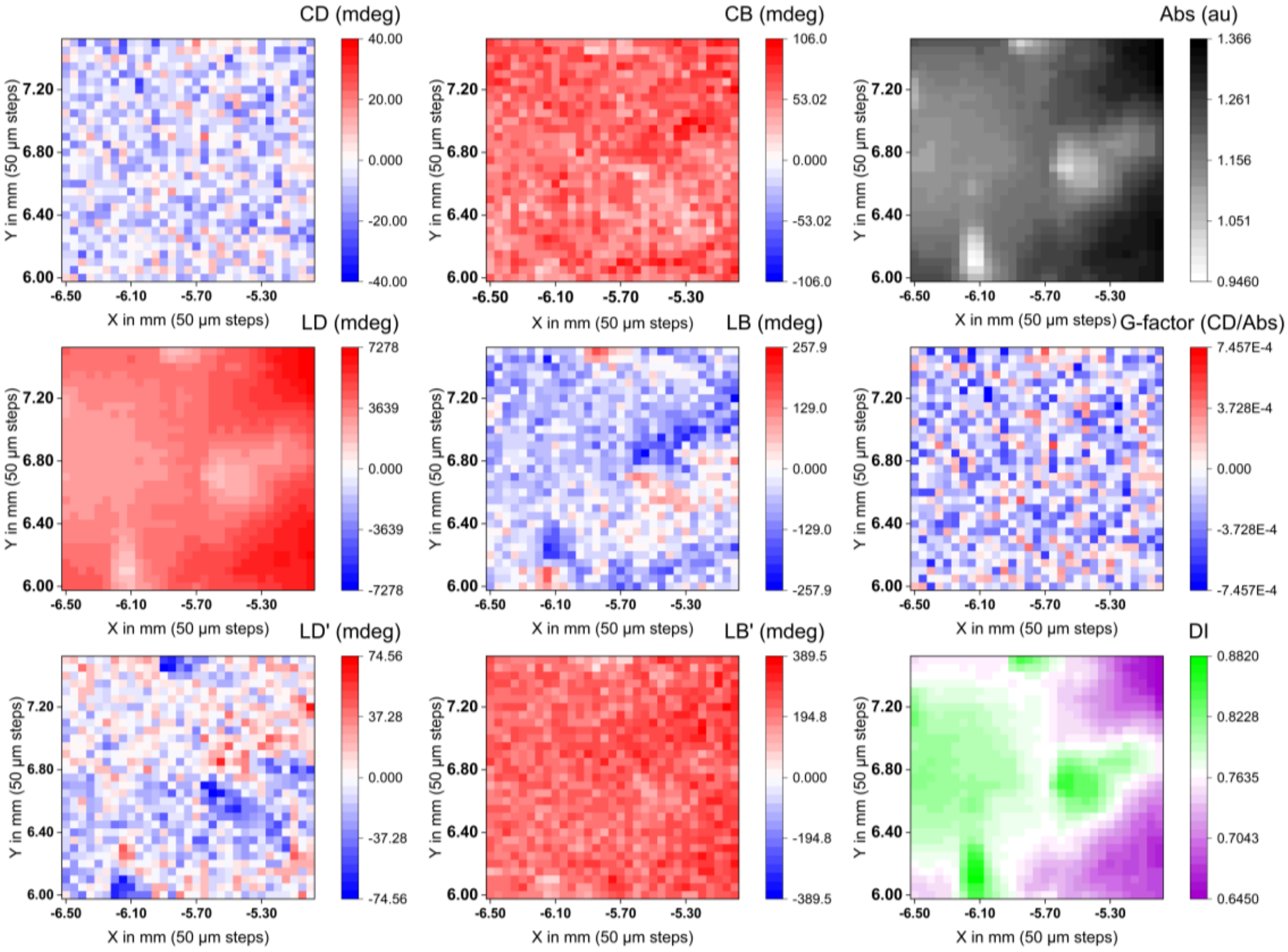

Supplementary Figure 5-27: CD, CB, Abs, LD, LB, g-factor, LD', LB' and DI Map recorded at 358 nm of racemic CDI thin film with background subtracted and processed with the Analytic Inversion method over an area of 1.5 mm x 1.5 mm with a resolution of 50 µm (31x31 steps).

Film Quantum Yield

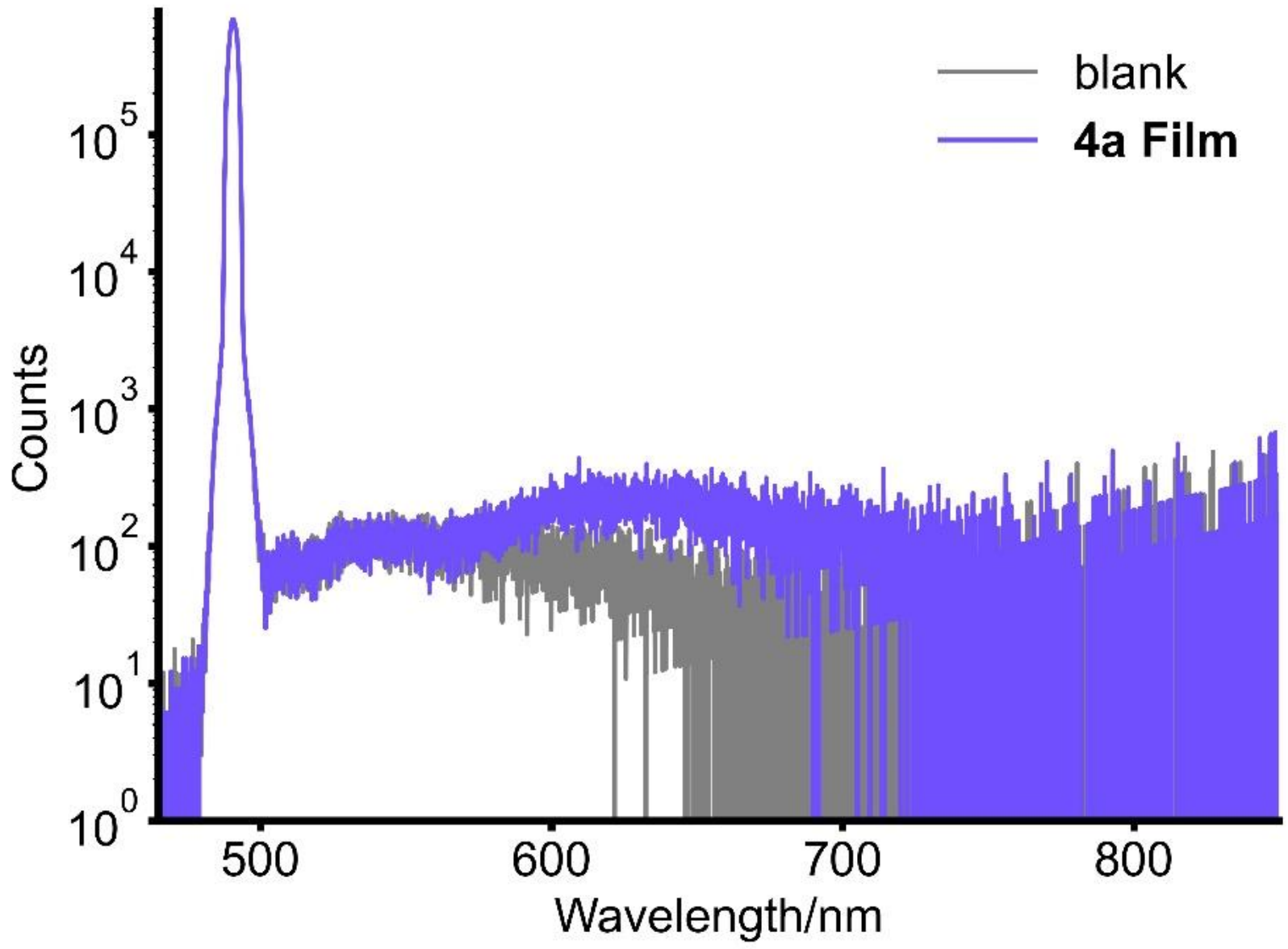

Supplementary Figure 5-28: Fluorescence quantum yield of a thin film of Lval-derived CDI **4a**. Film was excited using indirect excitation to avoid light-guiding. λ_{ex} = 490 nm, slit width (λ_{ex}) = 3 nm, slit width (λ_{em}) = 0.1 nm, step size = 0.1 nm, dwell time = 0.2 s, light intensity = 75%. Quantum yield (Φ) = 28%.

6) X-Ray Diffraction

Crystals of D-Val-PDI **2b** were obtained by slow evaporation from a solution in $CHCl_3$/MeOH. The obtained, kite-shaped plates were measured on beamline I19 at the UK Diamond Light Source on a 3-circle diffractometer (λ = 0.6889 Å).[21–23] A suitable single crystal was selected and mounted using fomblin film on a micromount. Data were collected on a Dectris PILATUS 2M detector. The crystals were kept at 100 K during data collection (single omega sweep). The structure was solved by direct methods using ShelXT[18] and refined with ShelXL[19] using a least squares method. Olex2 software was used as the solution, refinement and analysis program.[24] Hydrogen atoms were placed geometrically and refined using a riding model. Disorder was found in one TBS-group, which could not be accurately modeled due to a lack of residual electron density. The affected atoms were therefore refined isotropically.

Crystal Data for $C_{75}H_{80}N_8O_7Si_2$ (M =1261.65 g/mol): monoclinic, space group $P2_1$ (no. 4), a = 13.1378(3) Å, b = 11.1934(2) Å, c = 23.6687(5) Å, β = 94.993(2)°, V = 3467.43(12) $Å^3$, Z = 2, T = 100 K, μ(synchrotron) = 0.103 mm^{-1}, $Dcalc$ = 1.208 g/cm^3, 66601 reflections measured (3.016° ≤ 2Θ ≤ 71.56°), 30452 unique (R_{int} = 0.0772, R_{sigma} = 0.1573) which were used in all calculations. The final R_1 was 0.0793 (I > 2σ(I)) and wR_2 was 0.2237 (all data).

CCDC accession number: 2560271

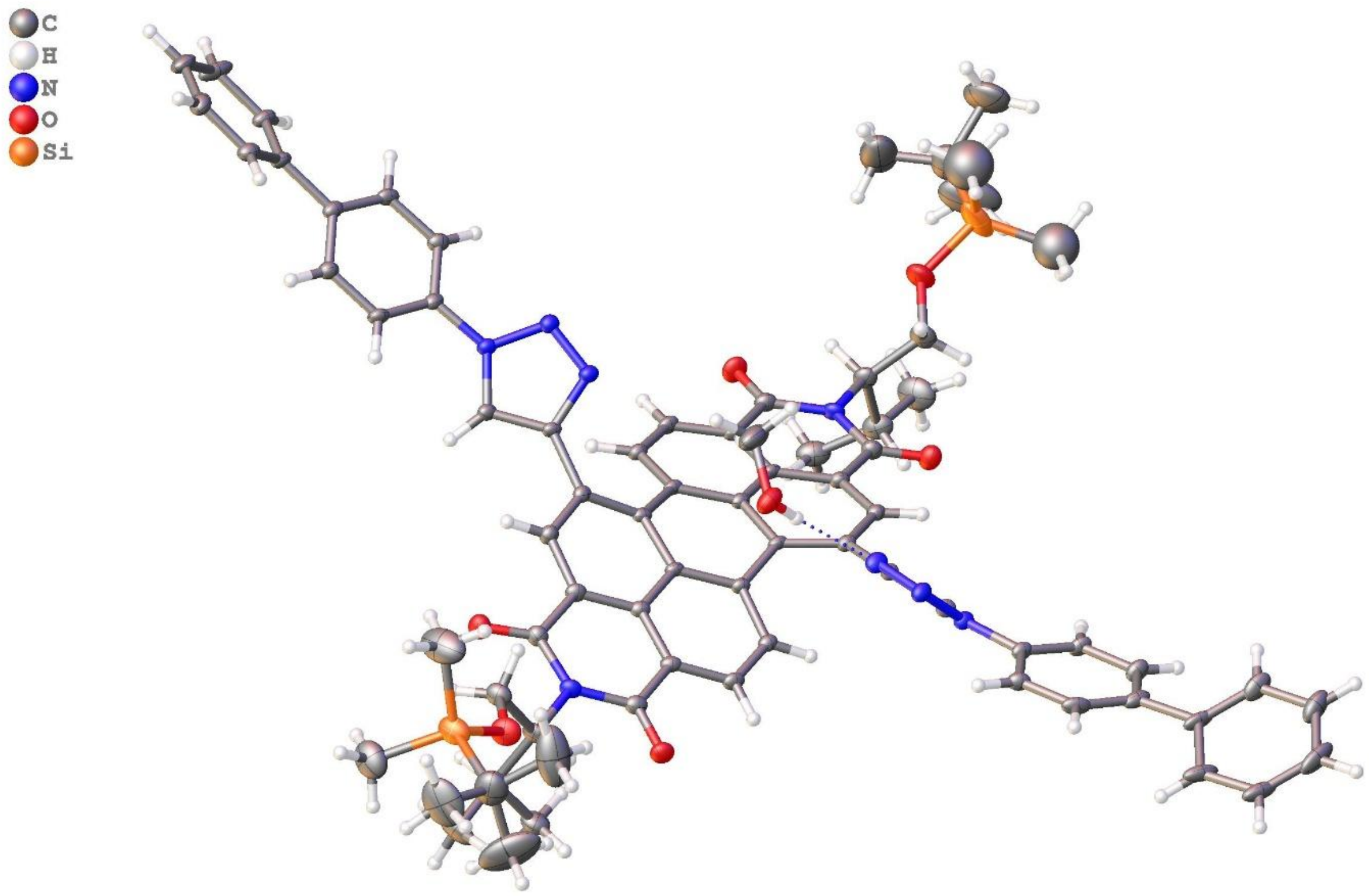

Supplementary Figure 6-1: Asymmetric unit of crystals of **2b**.

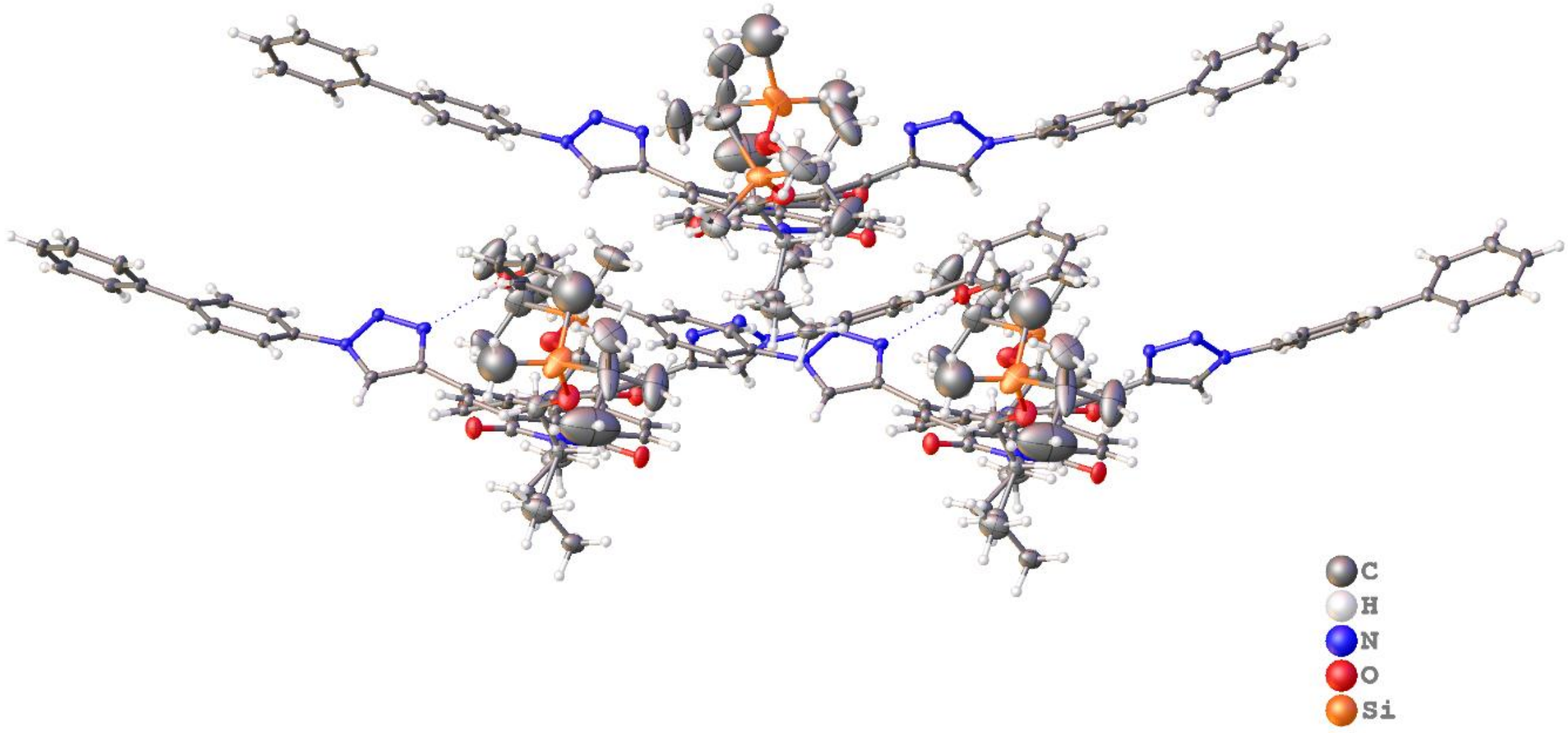

Supplementary Figure 6-2: Stacking of individual PDI-units in crystals of **2b**. Note that the PDIs cores are not directly in contact; instead π-stacking occurs via the biphenyl-arms.

Crystals of L-Val-CDI **3a** were grown from slow evaporation of a solution of $CHCl_3$. The obtained crystals were measured on an Agilent SuperNova single crystal X-ray diffractometer with dual wavelength microfocus X-ray source and an Atlas CCD detector. Data were collected at 100 K through the use of an Oxford Cryosystems cryostream device using Cu K_α radiation. Data were collected and processed using CrysAlisPro 1.171.43. The structure was solved by direct methods using ShelXT[18] and refined with ShelXL[19] using a least squares method. Olex2 software was used as the solution, refinement and analysis program.[24] Non-crystallographically-defined hydrogen atoms were placed geometrically and refined using a riding model.

Crystal Data for $C_{76}H_{74}Cl_6N_8O_6Si_2$ (M =1464.31 g/mol): triclinic, space group P1 (no. 1), a = 9.7509(9) Å, b = 11.5896(10) Å, c = 17.0226(11) Å, α = 108.578(7)°, β = 90.680(7)°, γ = 103.711(8)°, V = 1763.5(3) Å^3, Z = 1, T = 99.99(14) K, μ(Cu Kα) = 3.032 mm^{-1}, $Dcalc$ = 1.379 g/cm^3, 15488 reflections measured (5.502° ≤ 2Θ ≤ 140.142°), 8314 unique (R_{int} = 0.0844, R_{sigma} = 0.1019) which were used in all calculations. The final R_1 was 0.0945 (I > 2σ(I)) and wR_2 was 0.2714 (all data).

CCDC accession number: 2560269

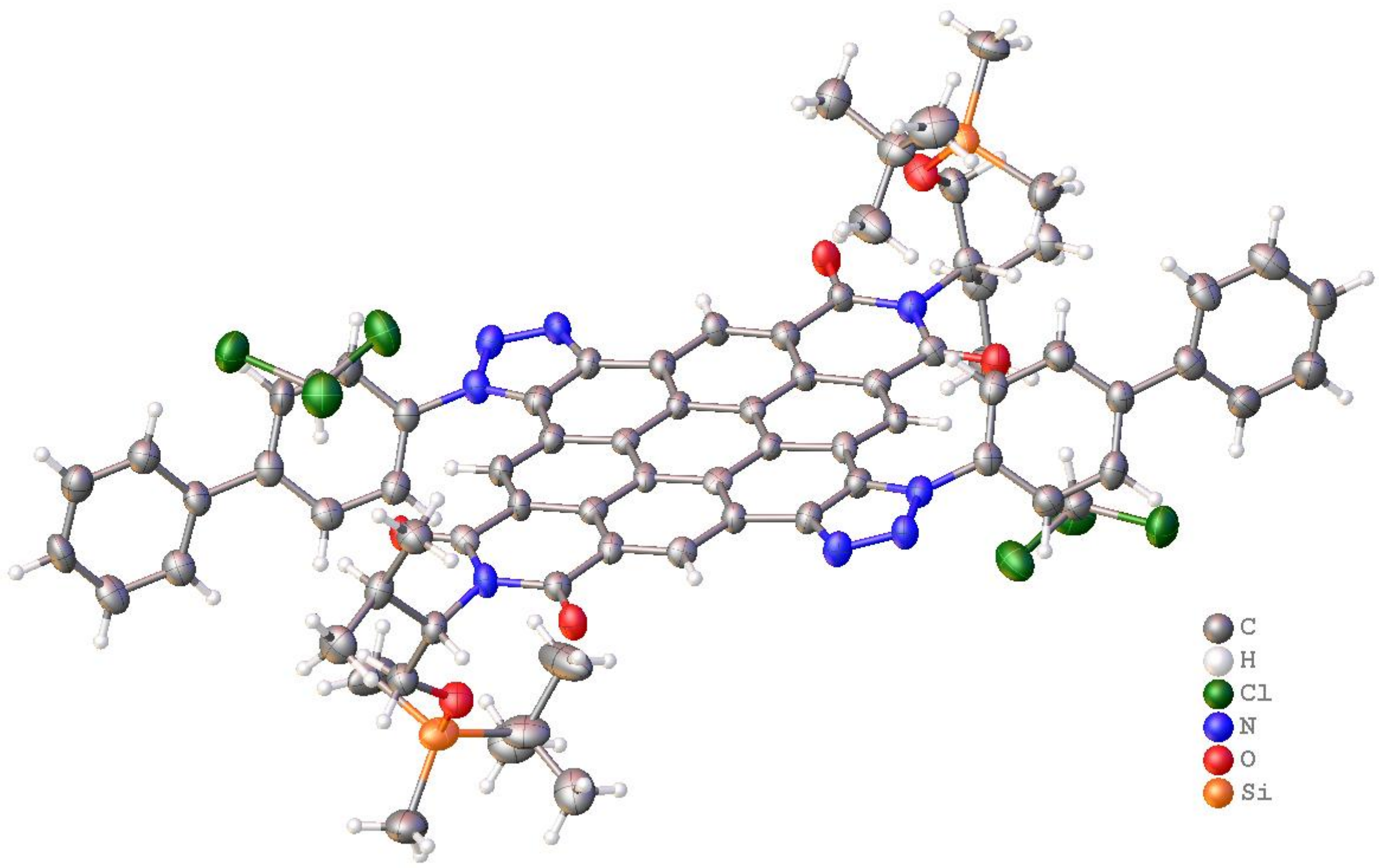

Supplementary Figure 6-3: Asymmetric unit of crystals of the TBS-protected, L-Val-derived CDI **3a**.

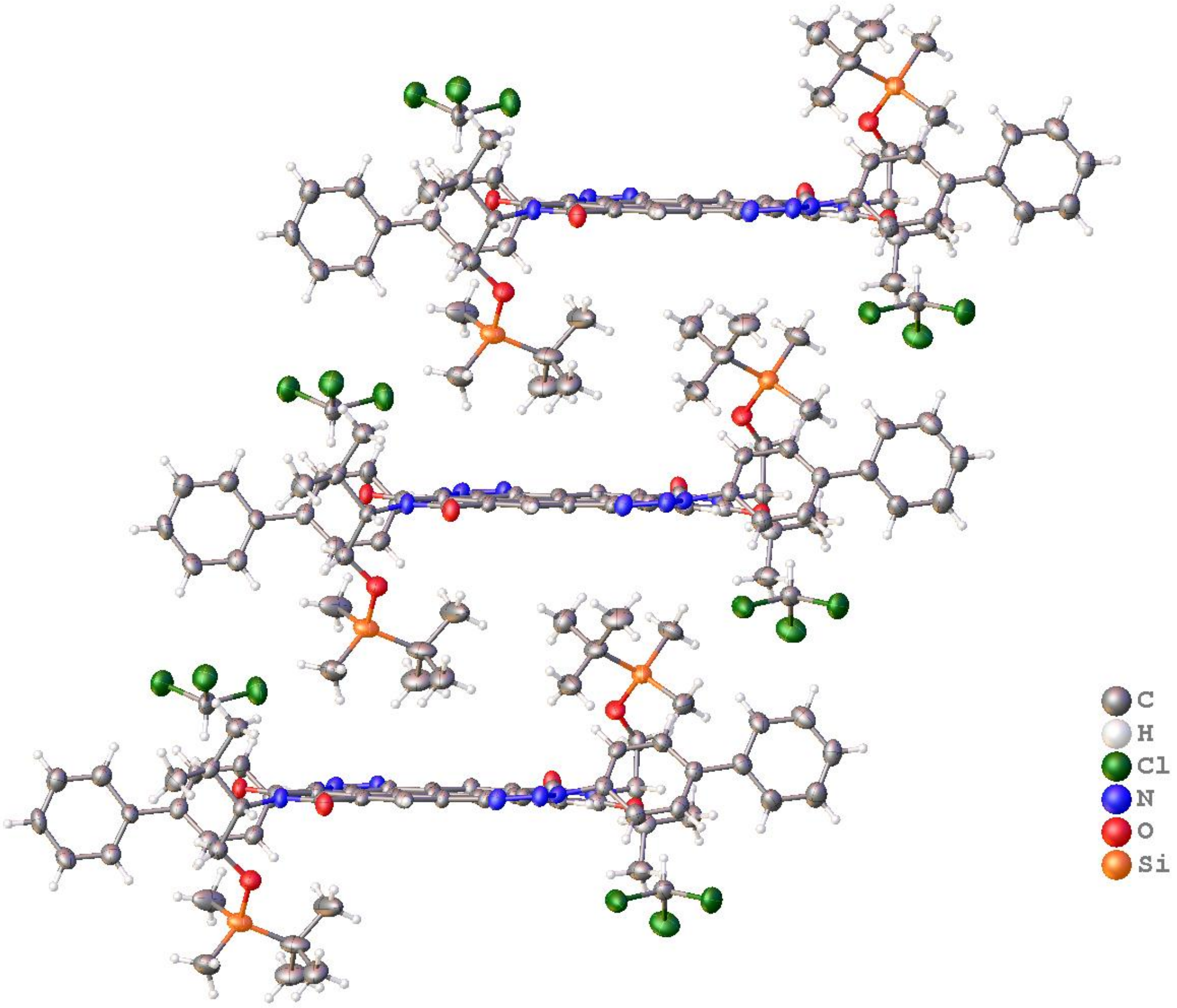

Supplementary Figure 6-4: Crystal packing of the TBS-protected, L-Val-derived CDI **3a** along the π-π direction. TBS groups hinder close approach of the π-surfaces.

Crystals of L-Val-CDI **3b** were grown from slow evaporation of a solution of CH_2Cl_2. The obtained crystals were measured on an Agilent SuperNova single crystal X-ray diffractometer with dual wavelength microfocus X-ray source and an Atlas CCD detector. Data were collected at 100 K through the use of an Oxford Cryosystems cryostream device using Cu K_α radiation. Data were collected and processed using CrysAlisPro 1.171.43. The structure was solved by direct methods using ShelXT[18] and refined with ShelXL[19] using a least squares method. Olex2 software was used as the solution, refinement and analysis program.[24] Non-crystallographically-defined hydrogen atoms were placed geometrically and refined using a riding model.

Crystal Data for $C_{76}H_{76}Cl_4N_8O_6Si_2$ (M =1395.42 g/mol): triclinic, space group P1 (no. 1), a = 9.7108(2) Å, b = 11.2454(3) Å, c = 17.2807(3) Å, α = 108.462(2)°, β = 91.5360(10)°, γ = 104.095(2)°, V = 1724.96(7) Å^3, Z = 1, T = 100.00(11) K, μ(Cu Kα) = 2.376 mm^{-1}, $Dcalc$ = 1.343 g/cm^3, 65272 reflections measured (5.426° ≤ 2Θ ≤ 154.436°), 13408 unique (R_{int} = 0.0302, R_{sigma} = 0.0189) which were used in all calculations. The final R_1 was 0.0329 (I > 2σ(I)) and wR_2 was 0.0907 (all data).

CCDC accession number: 2560270

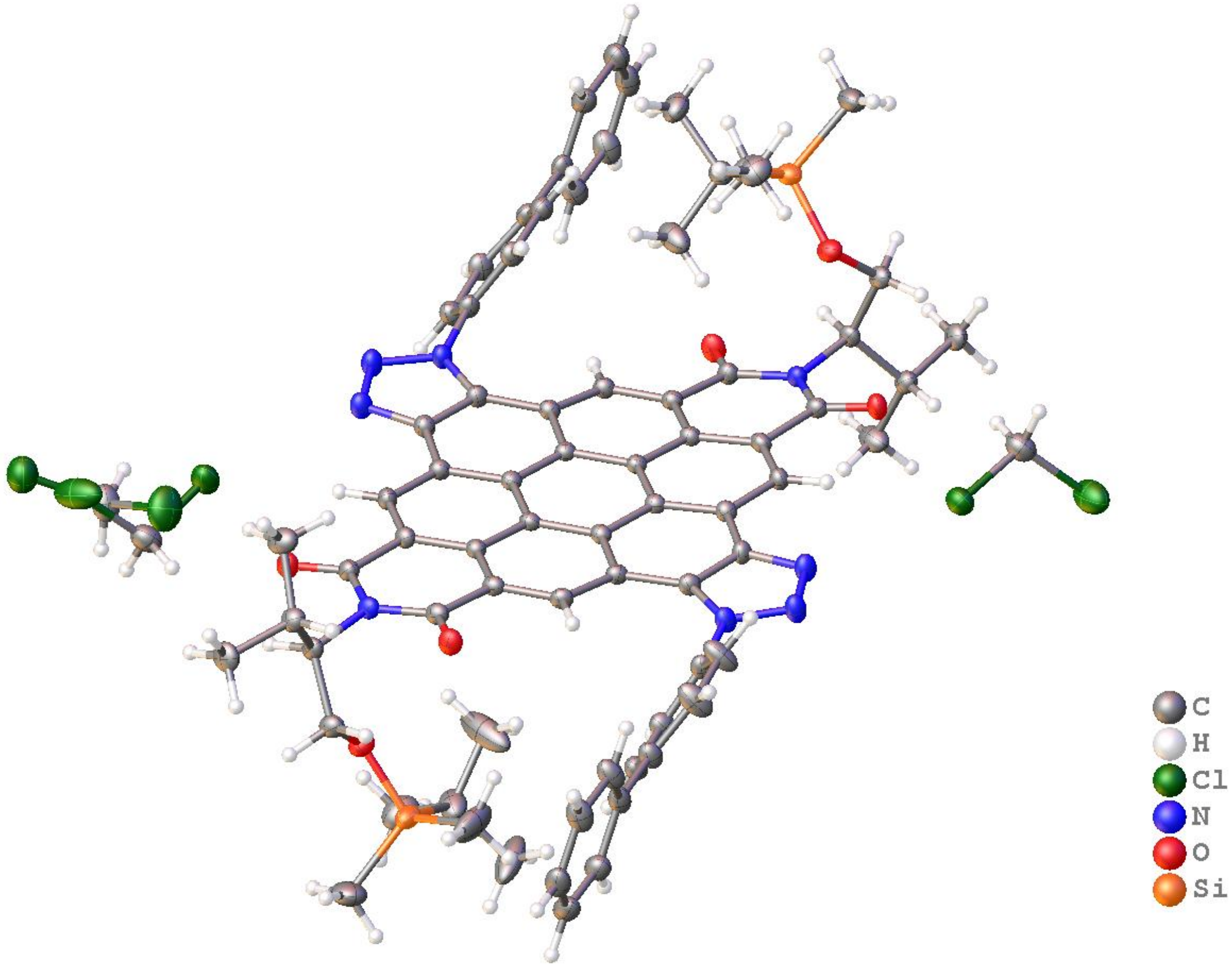

Supplementary Figure 6-5: Asymmetric unit of crystals of the TBS-protected, L-Val-derived CDI **3b**.

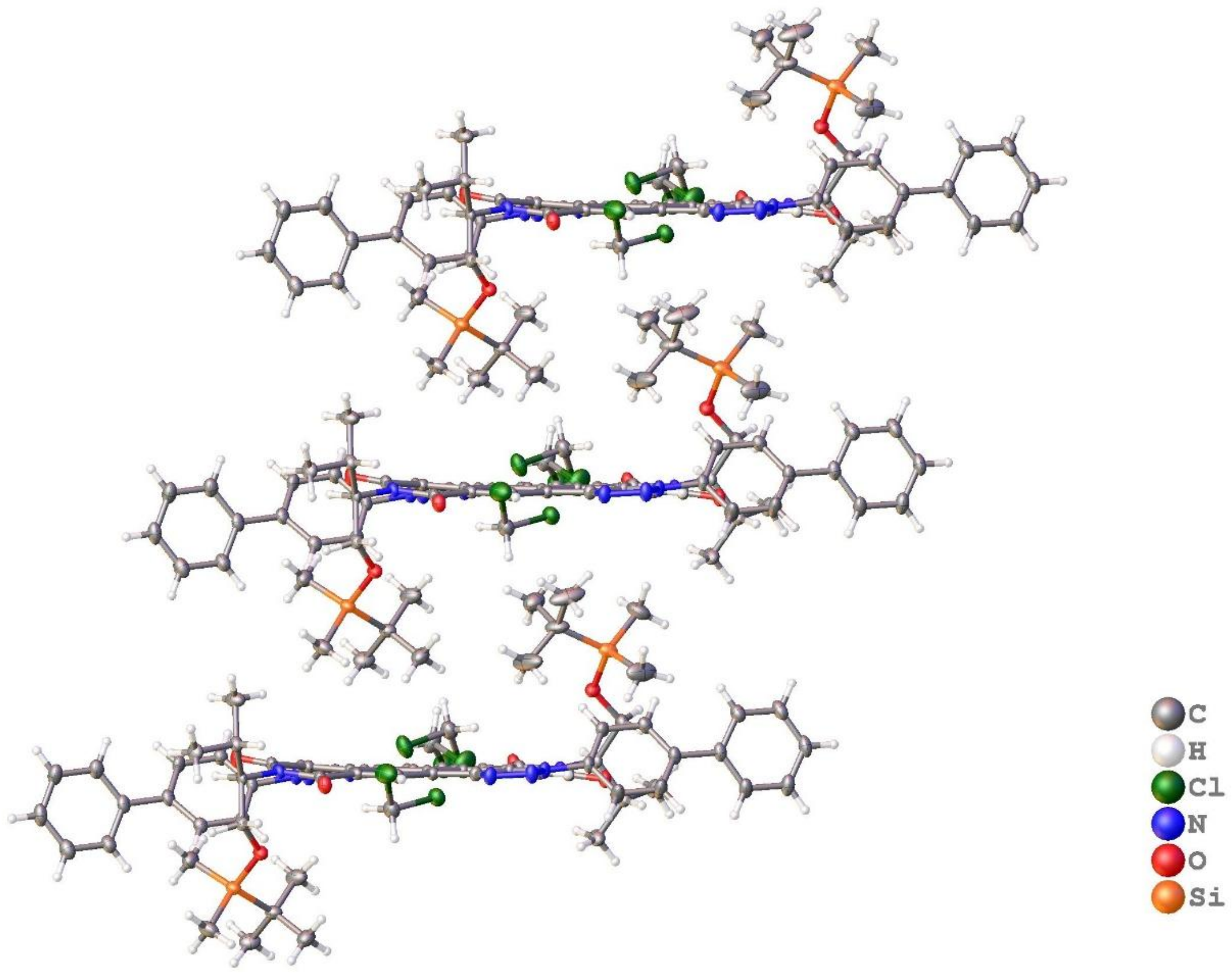

Supplementary Figure 6-6: Crystal packing of the TBS-protected, D-Val-derived CDI **3b** along the π-π direction. TBS groups hinder close approach of the π-surfaces.

Powder x-ray diffraction

Powder samples were analysed on a Rigaku XtaLAB Synergy R equipped with a HyPix-Arc 100 detector at 1 mR beam divergence over a scan range of 0-42 ° 2θ and a detector distance of 200 mm using the Gandolfi measurement method for powders. The solution sample was prepared by drying from MeOH solution, whereas the films were prepared as previous and a small amount of sample scraped off for measurement. The resulting powders were mounted on a MiTeGen micromesh (150 μm) for analysis.

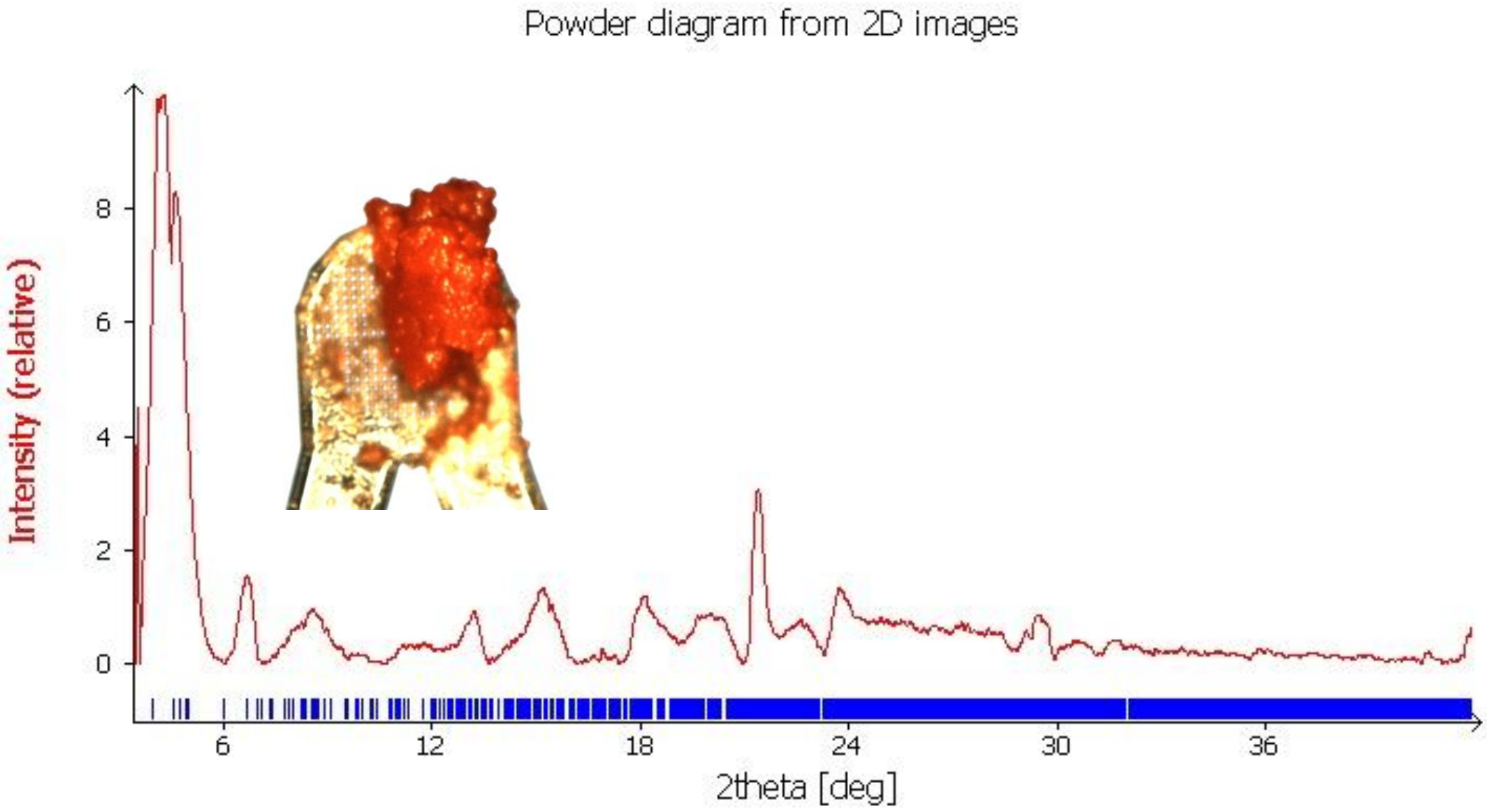

Supplementary Figure 6-7: Powder x-ray diffraction pattern (3-42° 2θ) obtained from scrapings of unannealed thin film of **4a** dropcast from $CHCl_3$ measured at 298 K.

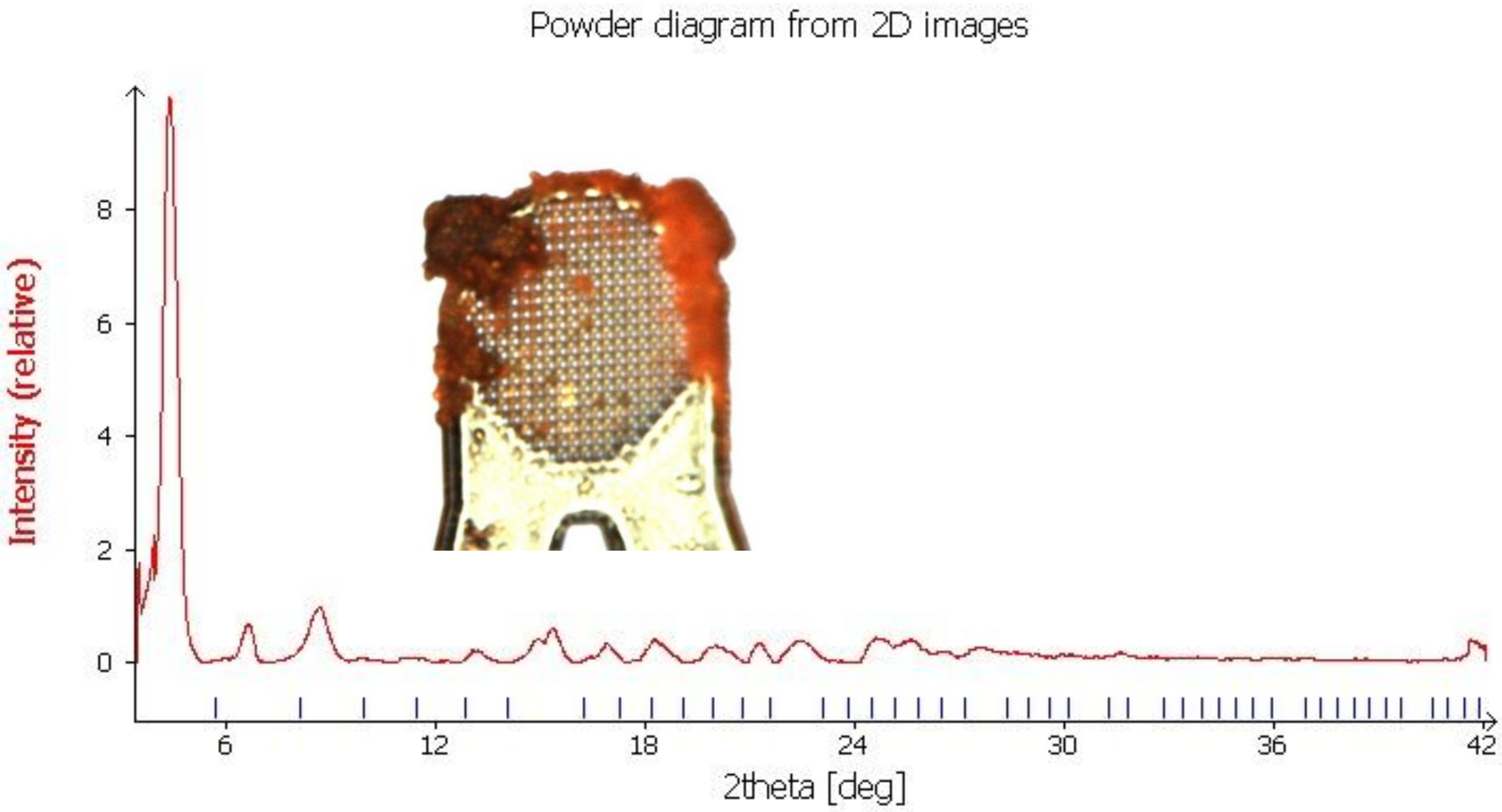

Supplementary Figure 6-8: Powder x-ray diffraction pattern (3-42° 2θ) obtained from scrapings of a thermally annealed thin film of **4a** dropcast from $CHCl_3$ measured at 298 K.

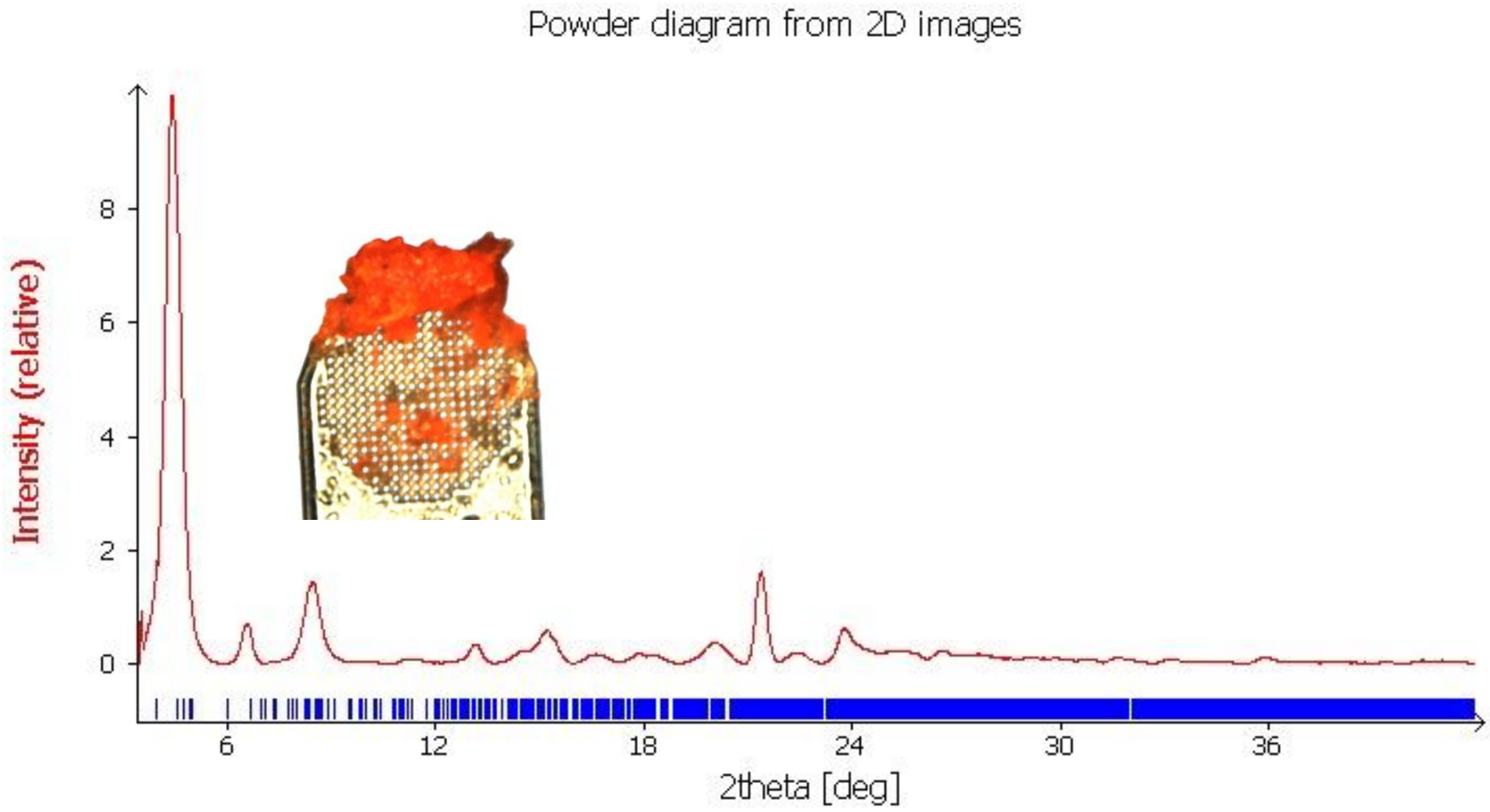

Supplementary Figure 6-9: Powder x-ray diffraction pattern (3-42° 2θ) obtained from the dried residue of a solution of **4a** in MeOH measured at 298 K.

7) Computational Chemistry

General

Density functional theory (DFT) calculations were performed in ORCA 6.0[25] unless otherwise indicated. All functionals and basis sets were chosen following the best-practice DFT protocols published by Grimme.[26]

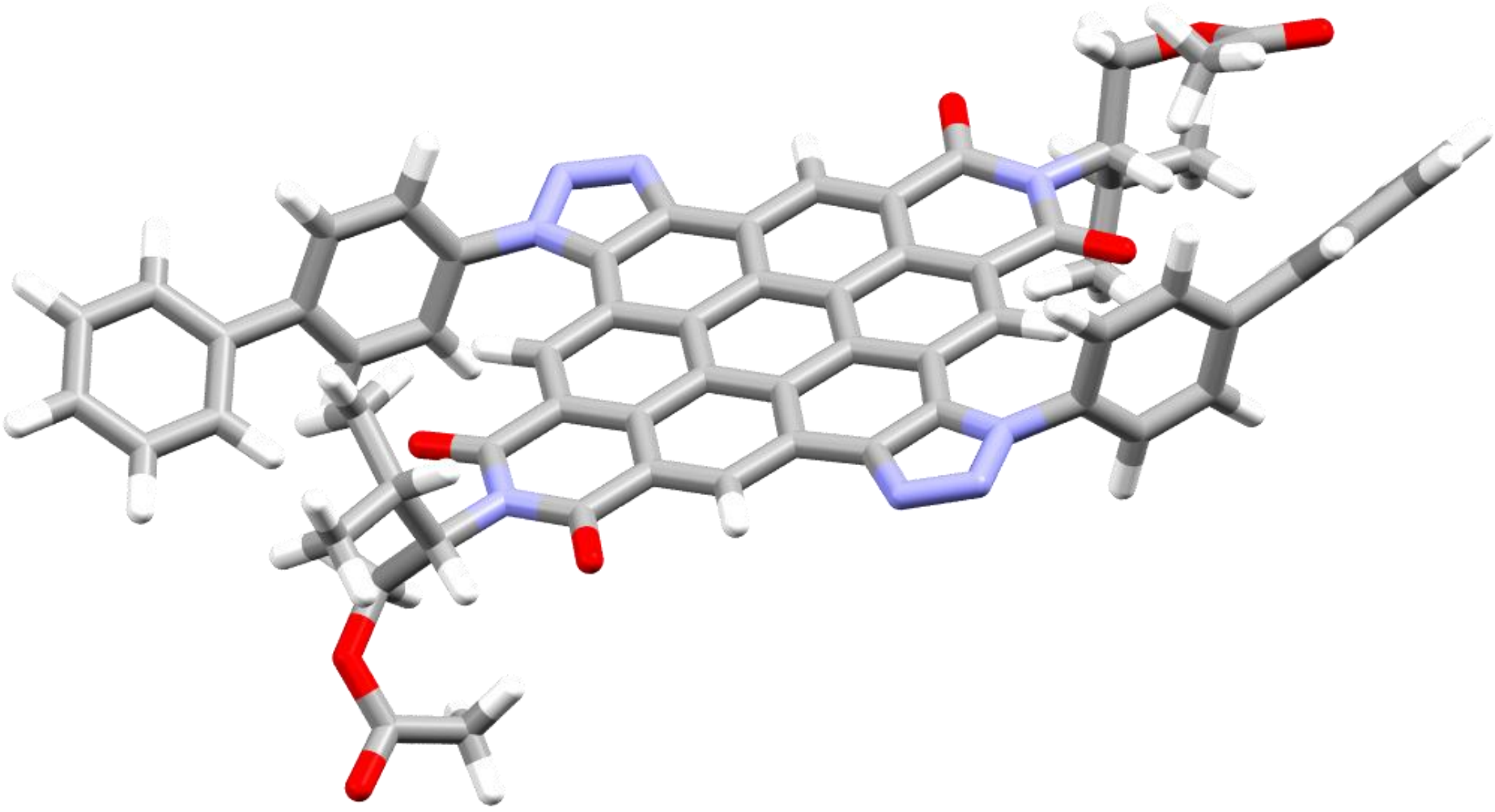

Supplementary Figure 7-1: Structure of DFT-optimised monomer CDI (B97-3c, def2-TZVP) with L-valinol-imide groups. Octanoyl groups were simplified to acetate.

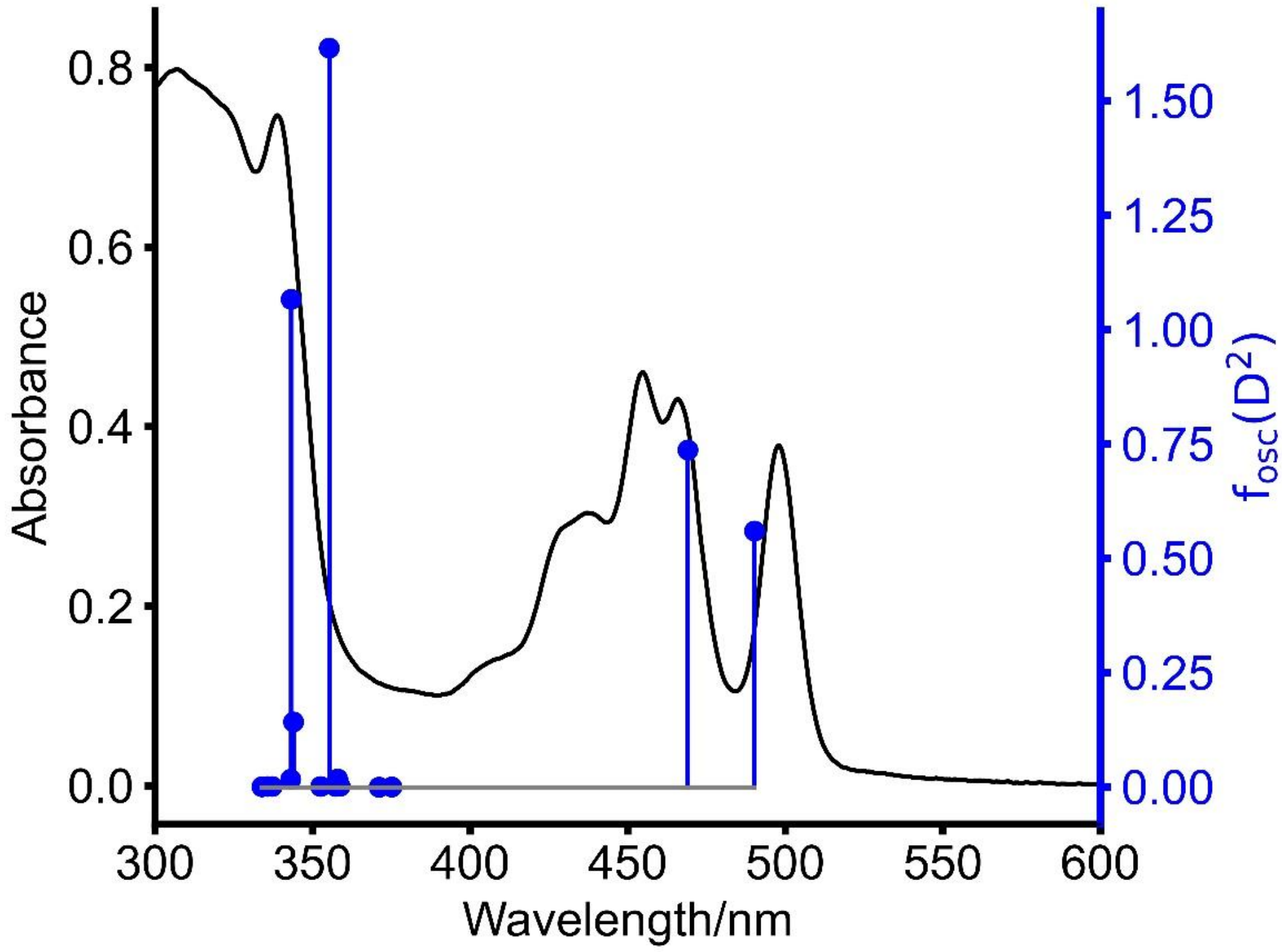

Supplementary Figure 7-2: Predicted absorption spectrum by TD-DFT (ωB97x, def2-SVP, CPCM(Chloroform)) of monomeric CDI with simplified imide groups from **Supplementary Figure 7-1** (blue) overlaid with the measured absorption spectrum of **4a** in $CHCl_3$. Predicted spectrum was shifted by +85 nm to account for the blue shift of the range-separated functional used.

Supplementary Table 7-1: Predicted transitions of monomeric CDI from **Supplementary Figure 7-1** by TD-DFT (ωB97x, def2-SVP, CPCM(Chloroform)).

Transition	Wavelength (nm)	$f_{osc}(D^2)$
**1**	405.2	0.559739
**2**	384.1	0.736866
**3**	290.1	4.42E-05
**4**	286.1	3.01E-06
**5**	273.7	0.001907
**6**	272.9	0.017482
**7**	272.3	0.001343
**8**	270.3	1.615578
**9**	267.5	0.001432
**10**	258.9	0.142434
**11**	258.2	1.066003
**12**	258	0.017221
**13**	252.2	0.001178
**14**	250.7	0.001505
**15**	248.9	0.000608

8) Atomic Force Microscopy

General

Samples were measured on a Bruker MultiMode 8, equipped with a SCANASYST-AIR probe, using a ScanAsyst in air at 2 kHz Peak Force Tapping Frequency. Size measurements were performed in ImageJ.

AFM images of thin film samples

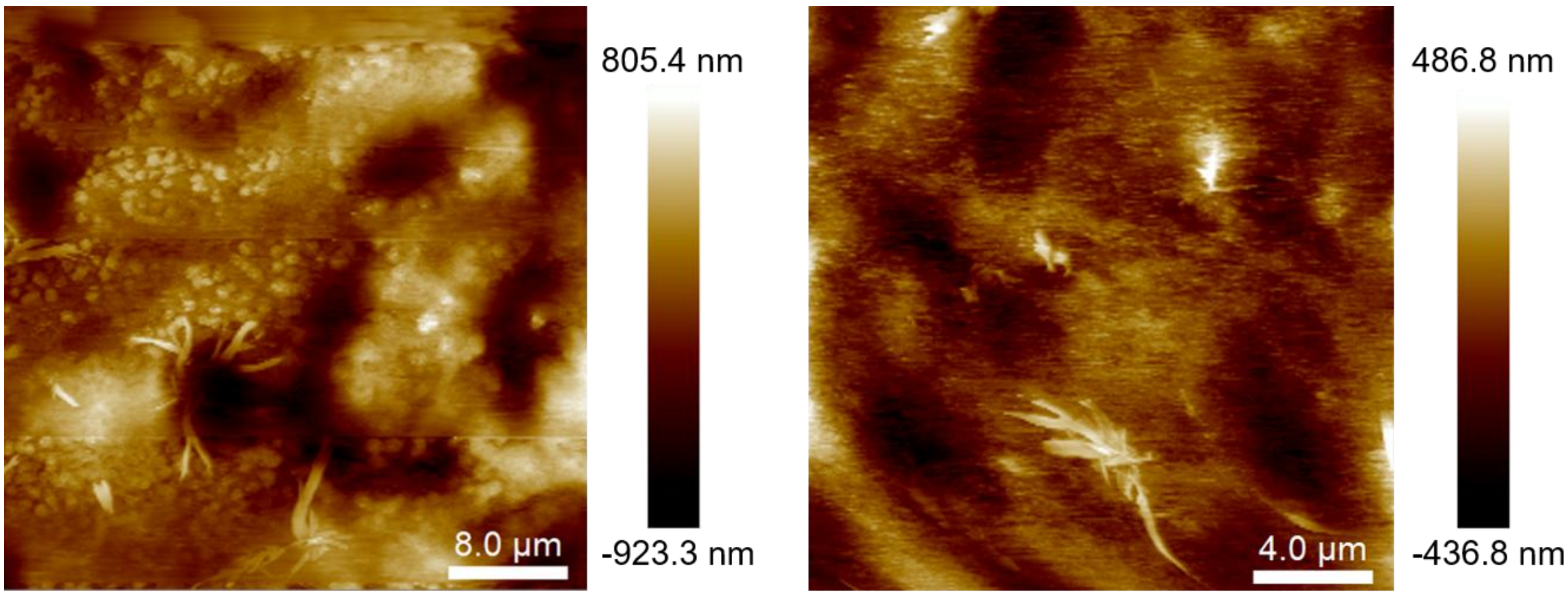

Supplementary Figure 8-1: AFM images of two different annealed films of CDI **4a**.

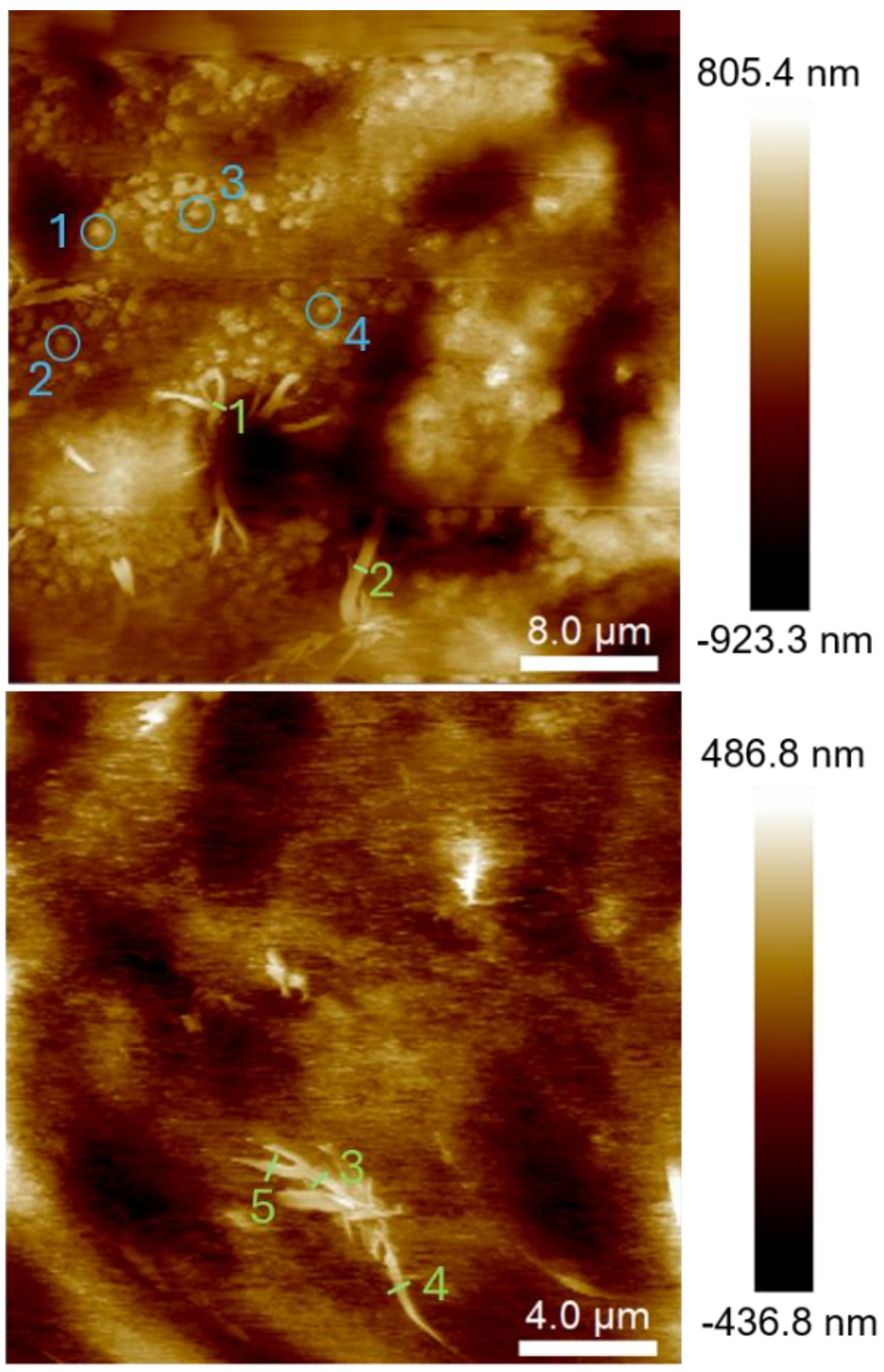

Spot	Diameter (µm)
1	1.06
2	0.97
3	0.60
4	0.78

Fibre	Diameter (µm)
1	0.62
2	0.78
3	0.31
4	0.37
5	0.36

Supplementary Figure 8-2: Measurements of features found in AFM images of annealed films of CDI **4a**.

9) NMR Spectra

OH-D-Valinol-Br₂-PDI S1

^{1}H NMR (400 MHz, $CDCl_3$)

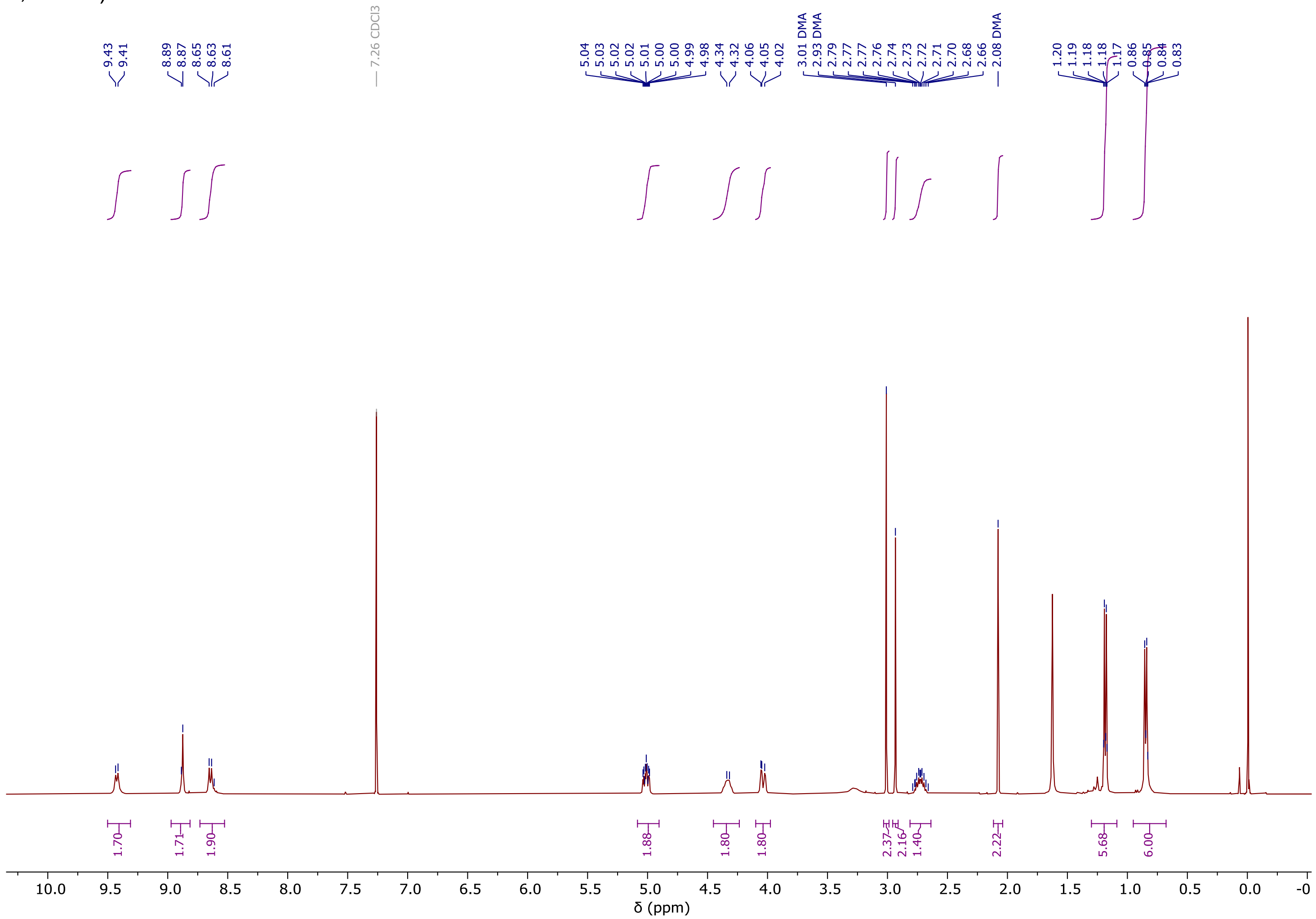

^{13}C NMR (101 MHz, $CDCl_3$)

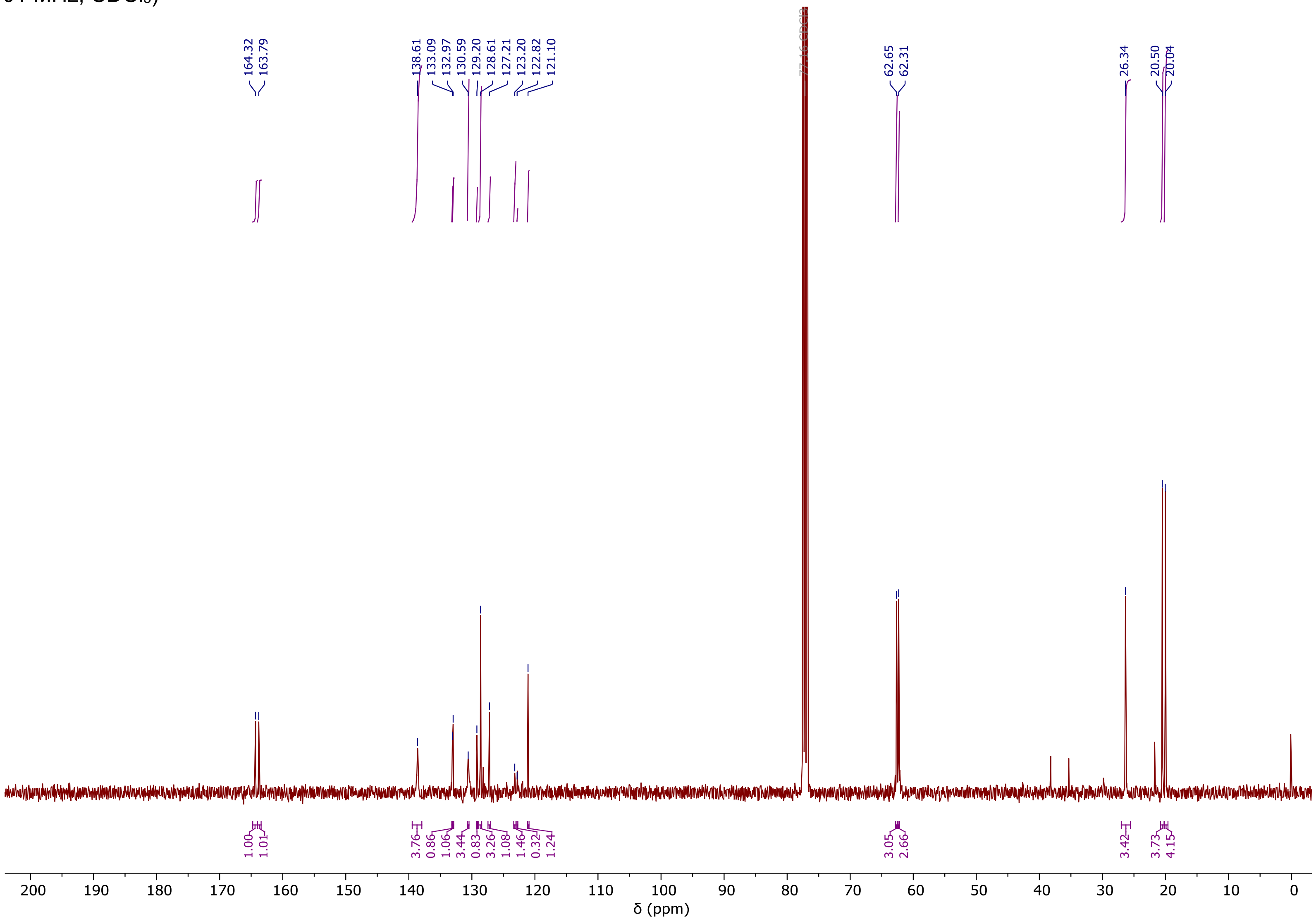

TBSO-D-Valinol-Br$_2$-PDI S2

^{1}H NMR (400 MHz, $CDCl_3$)

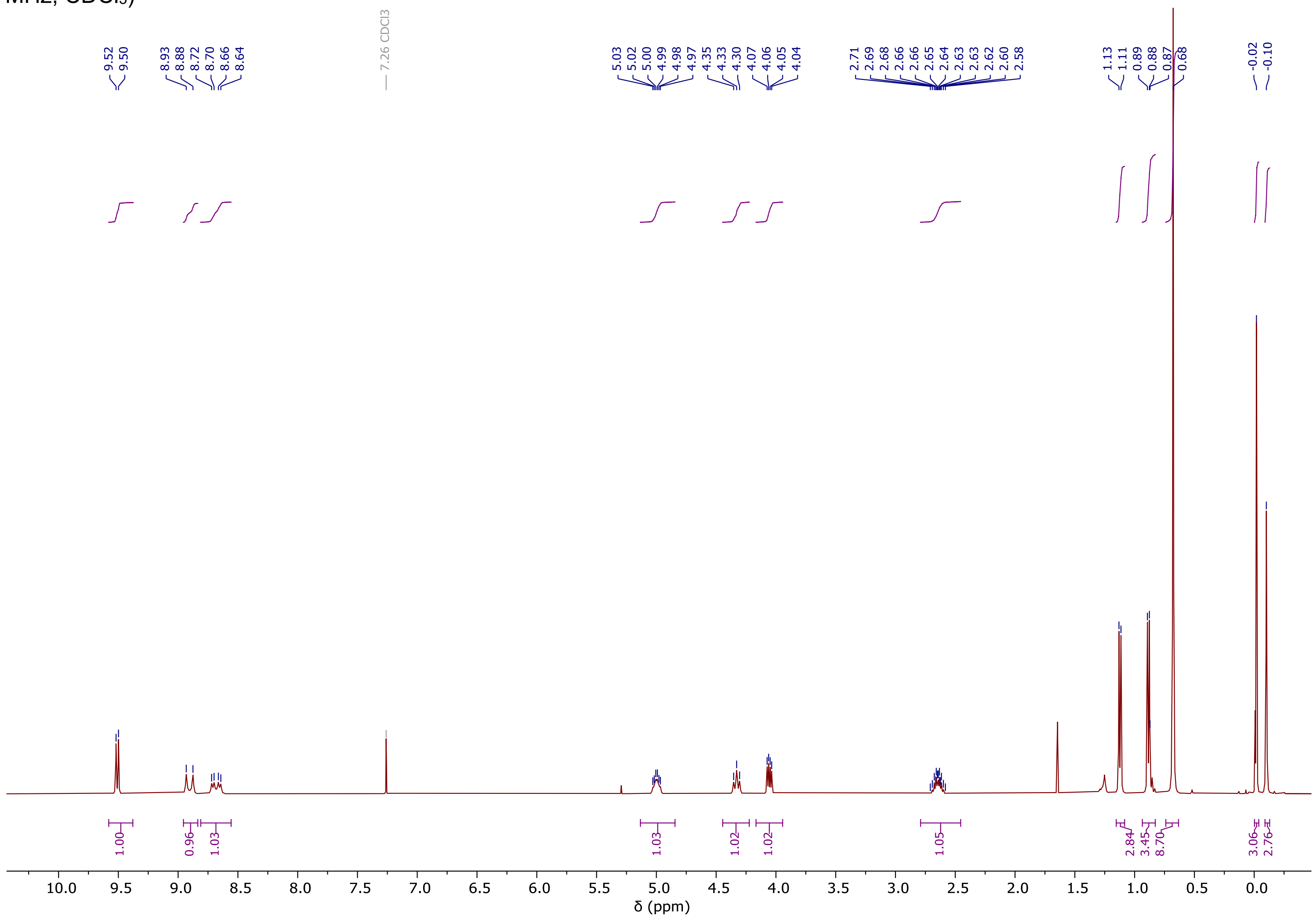

¹³C NMR (101 MHz, CDCl₃)

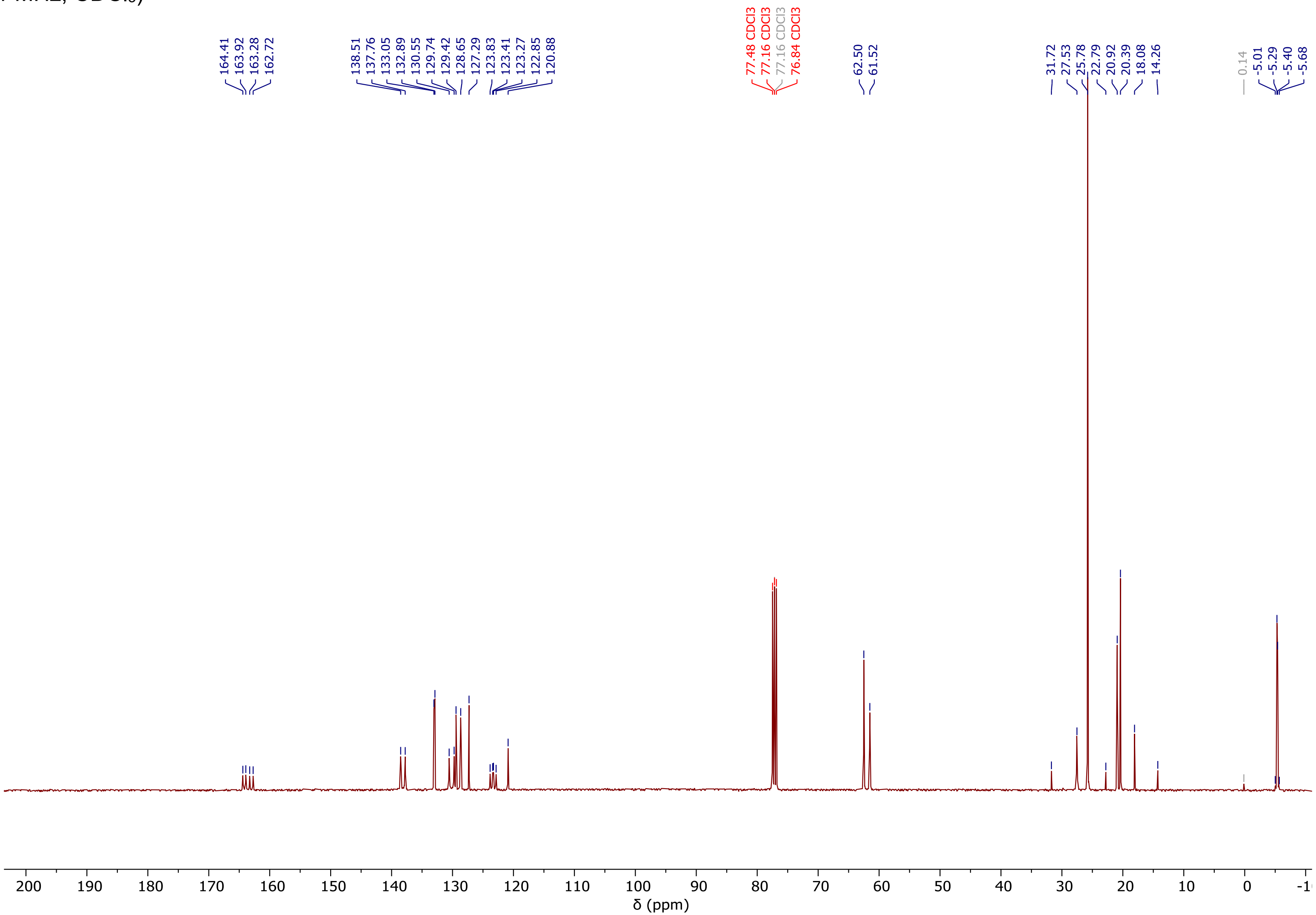

TBSO-L-Valinol-bis-TMS-acetylene-PDI 1a

^{1}H NMR (400 MHz, $CDCl_3$)

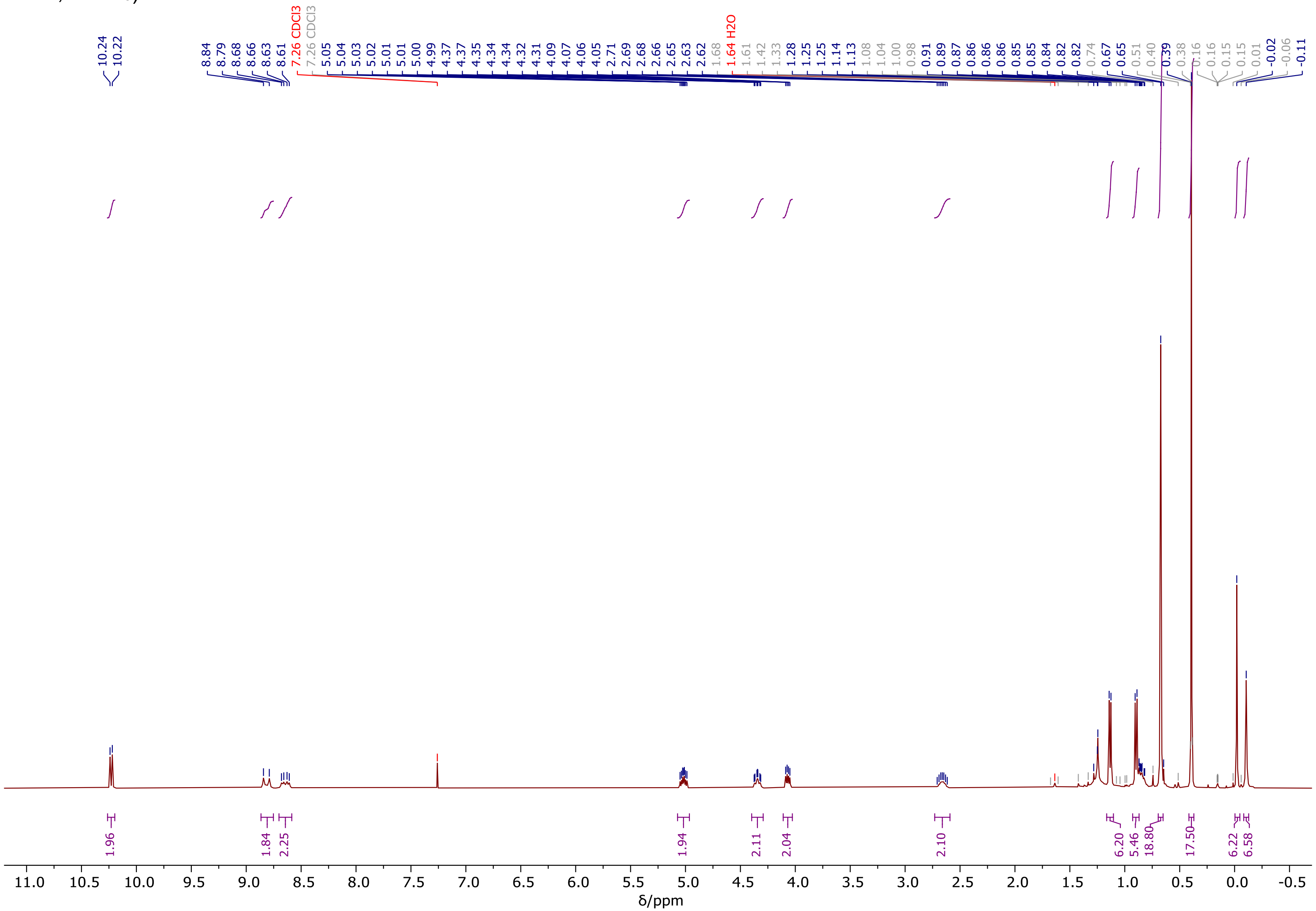

^{13}C NMR (101 MHz, $CDCl_3$)

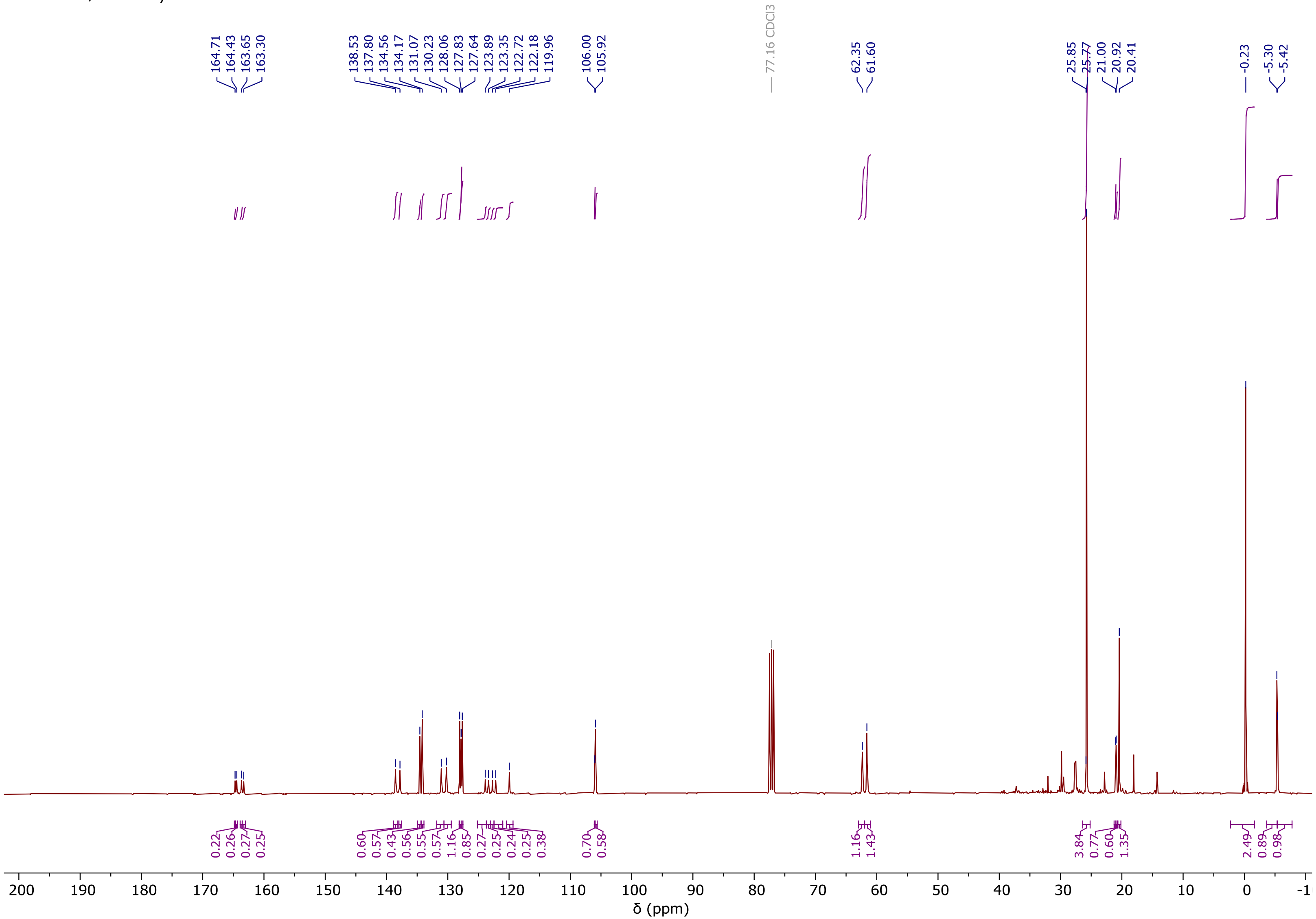

TBSO-D-Valinol-bis-TMS-acetylene-PDI 1b

^{1}H NMR (400 MHz, $CDCl_3$)

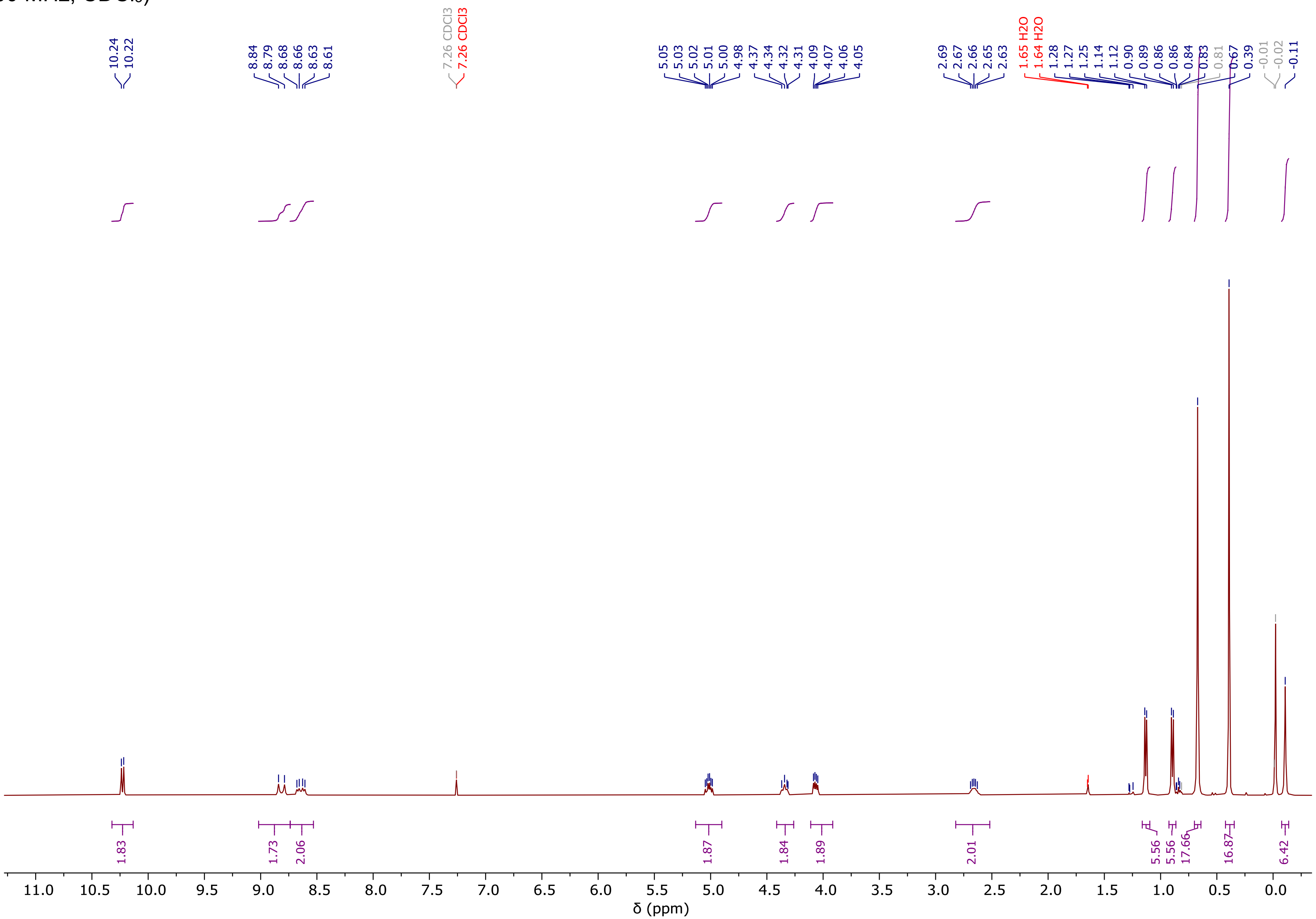

13C NMR (101 MHz, $CDCl_3$)

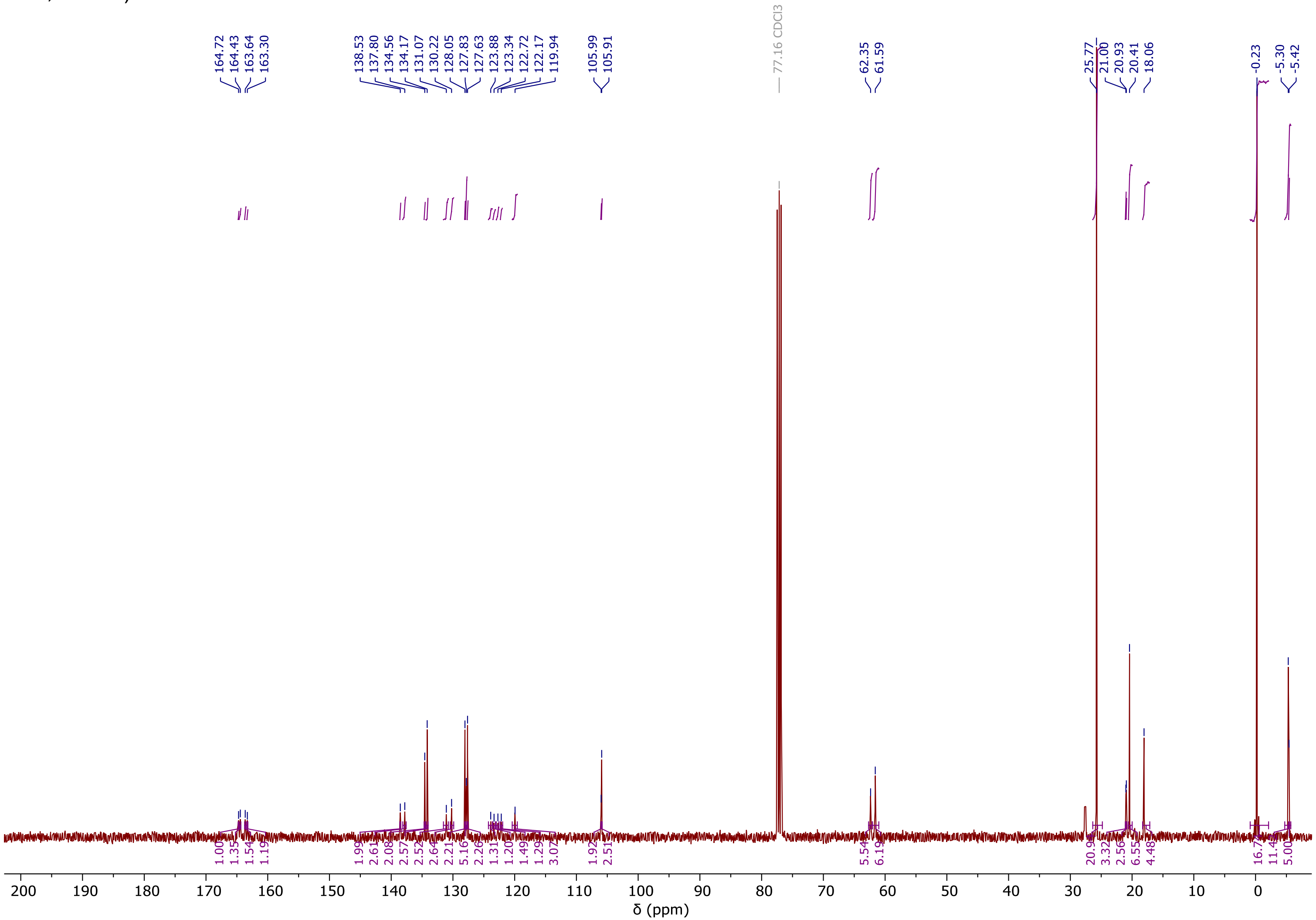

Biphenyl Azide S3

^{1}H NMR (300 MHz, $CDCl_3$)

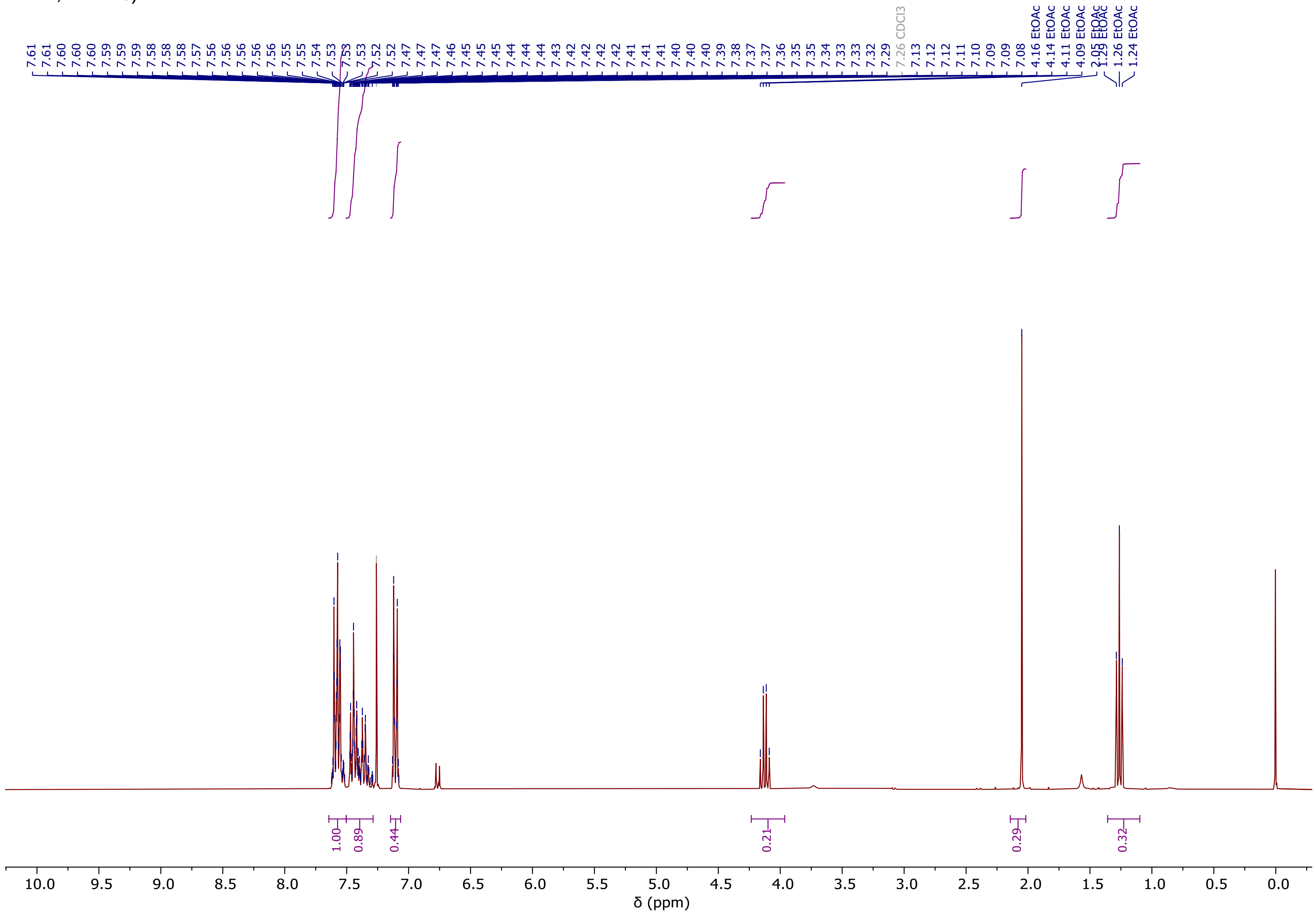

TBSO-LVal-biphenyl triazole-PDI 2a

^{1}H NMR (400 MHz, $CDCl_3$)

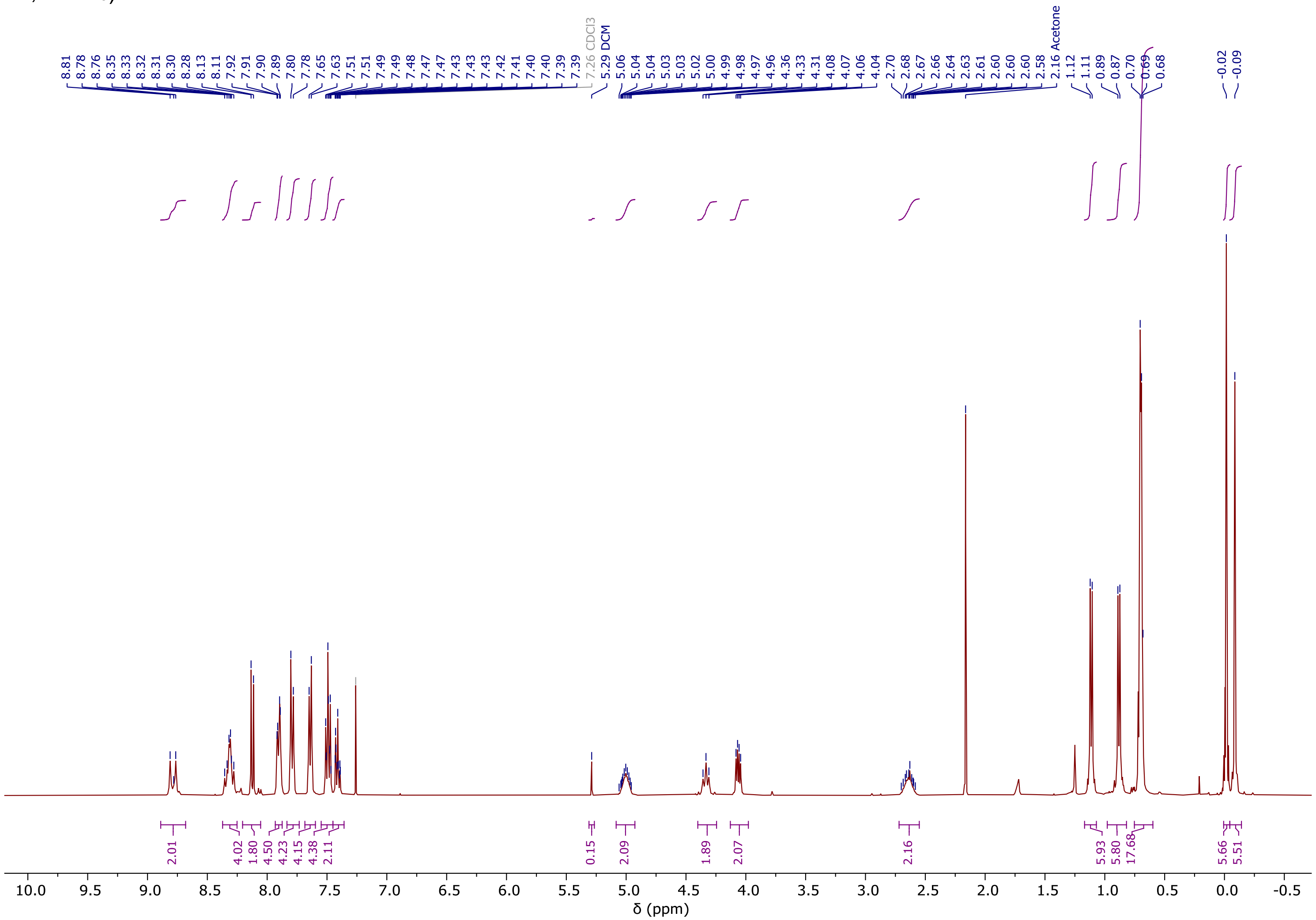

^{13}C NMR (101 MHz, $CDCl_3$)

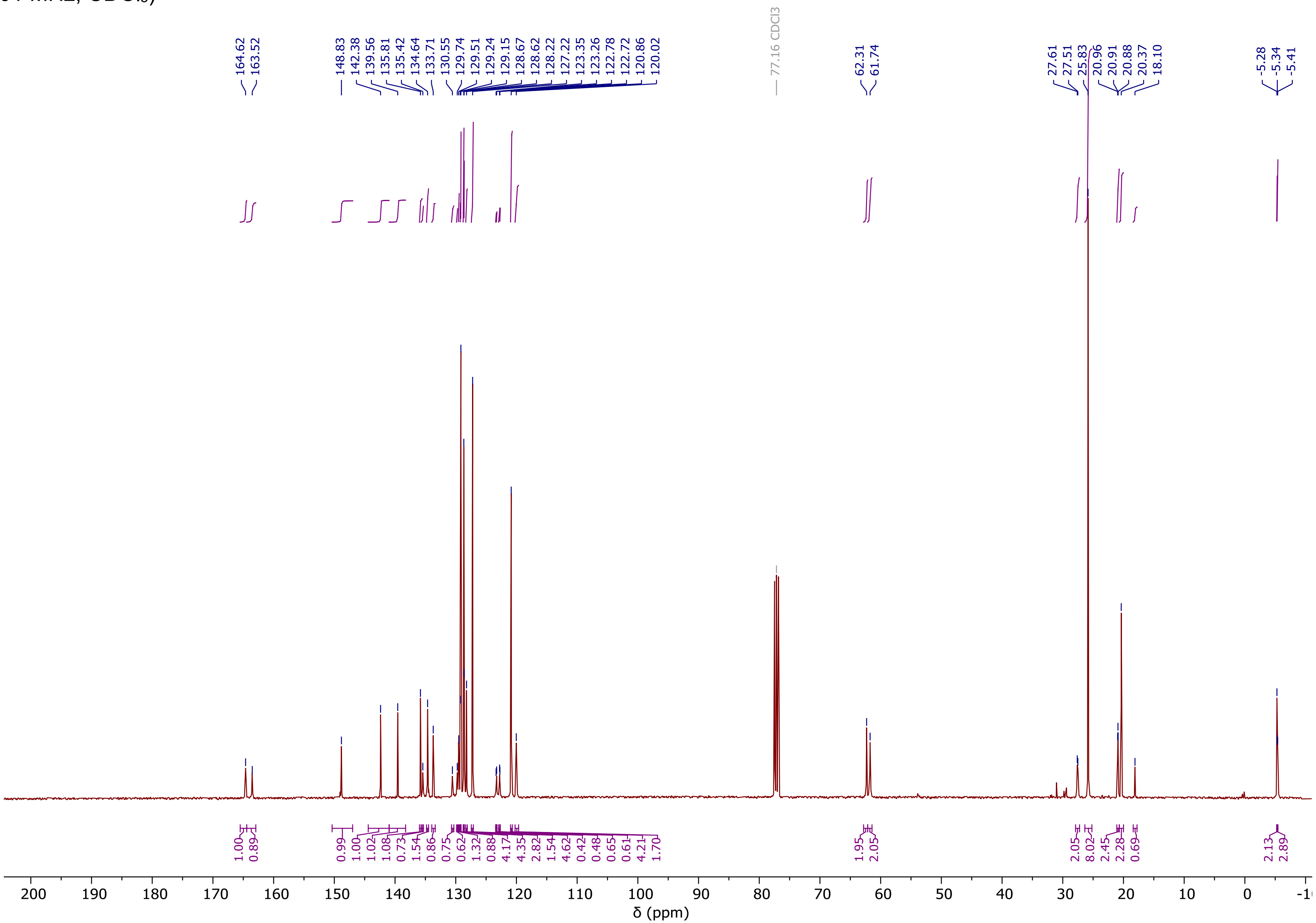

TBSO-DVal-biphenyl triazole-PDI 2b

^1H NMR (400 MHz, $CDCl_3$)

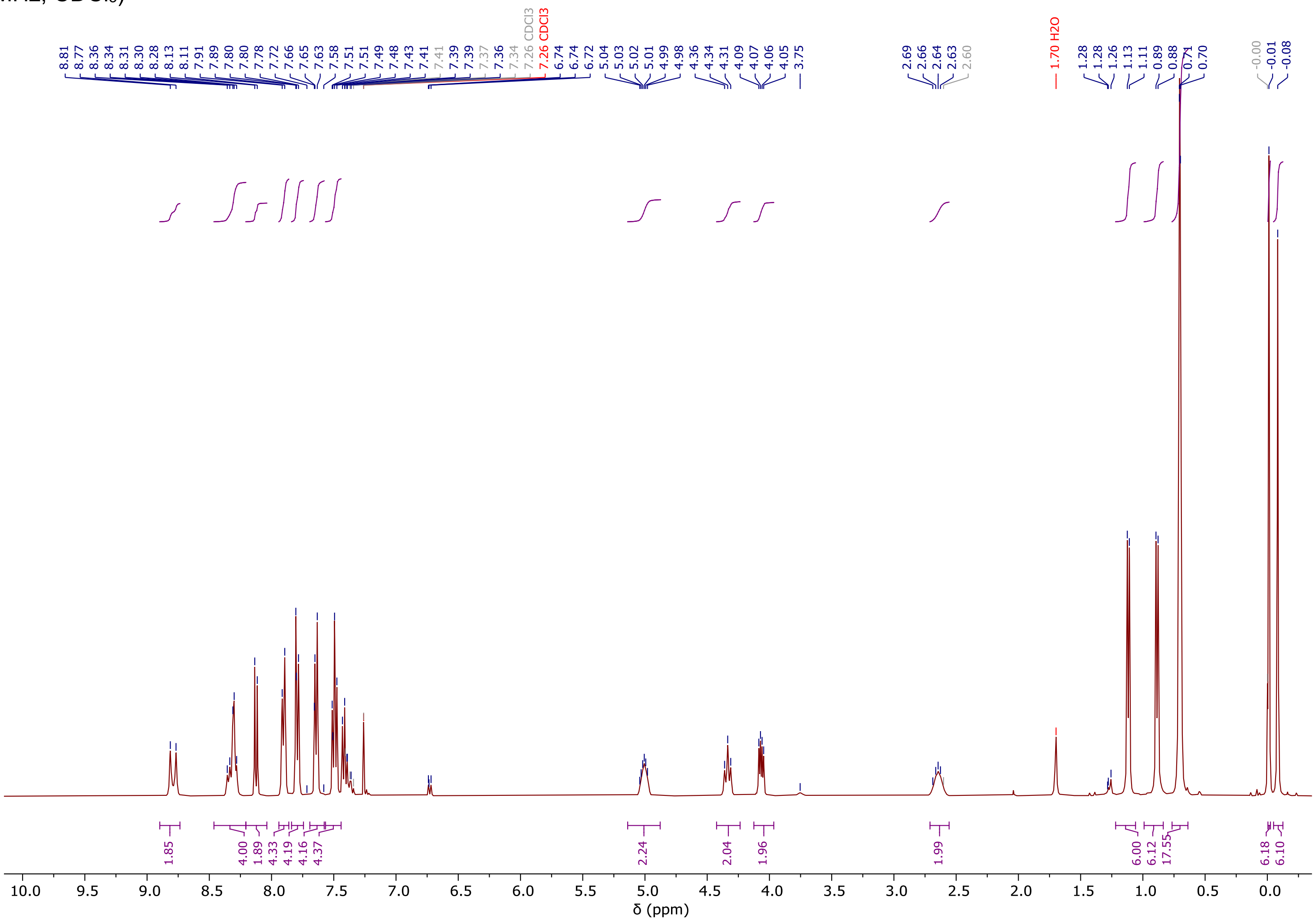

^{13}C NMR (101 MHz, $CDCl_3$)

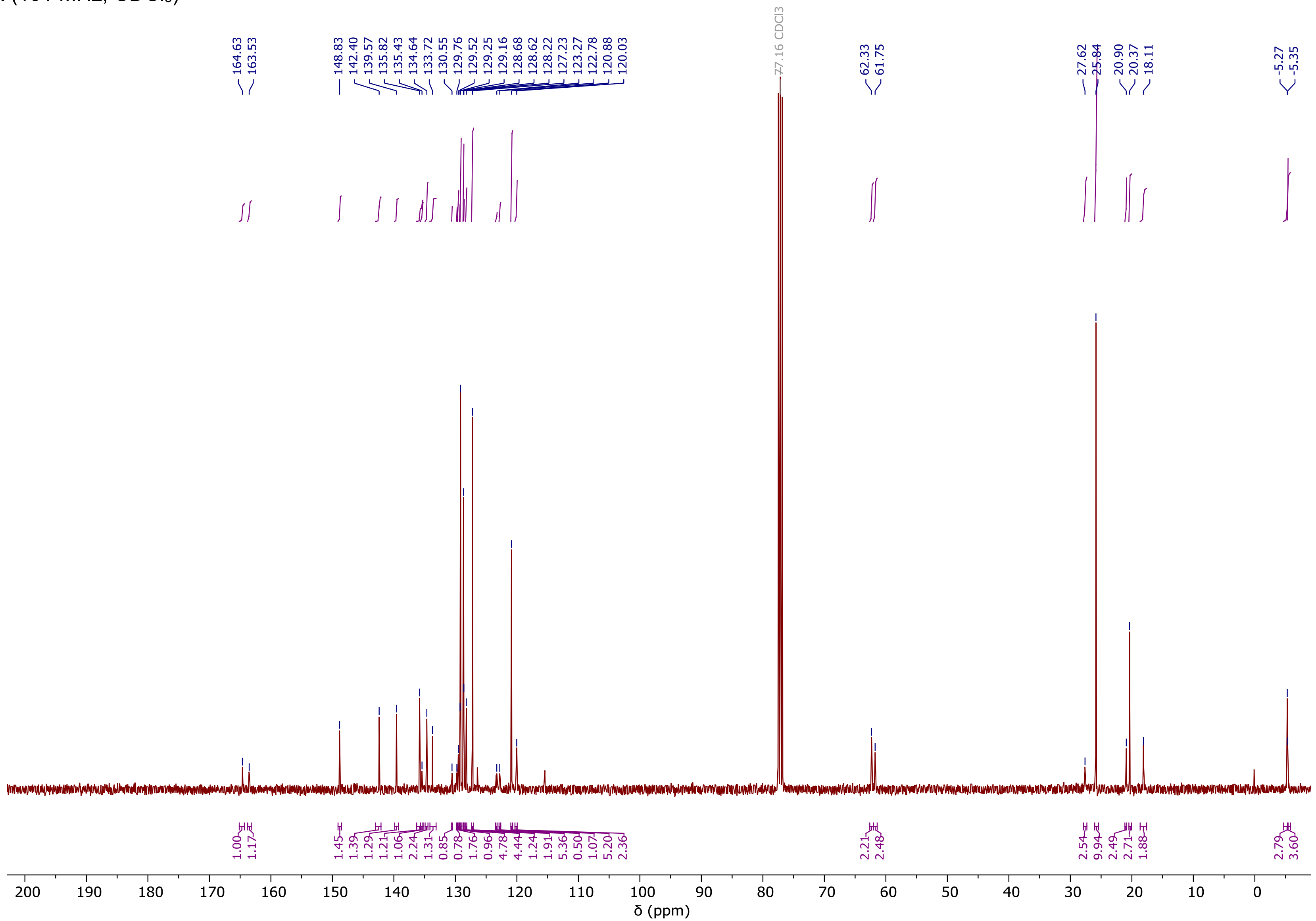

TBSO-LVal-biphenyl triazole-CDI 3a

^{1}H NMR (400 MHz, TCE-d_2, 373 K)

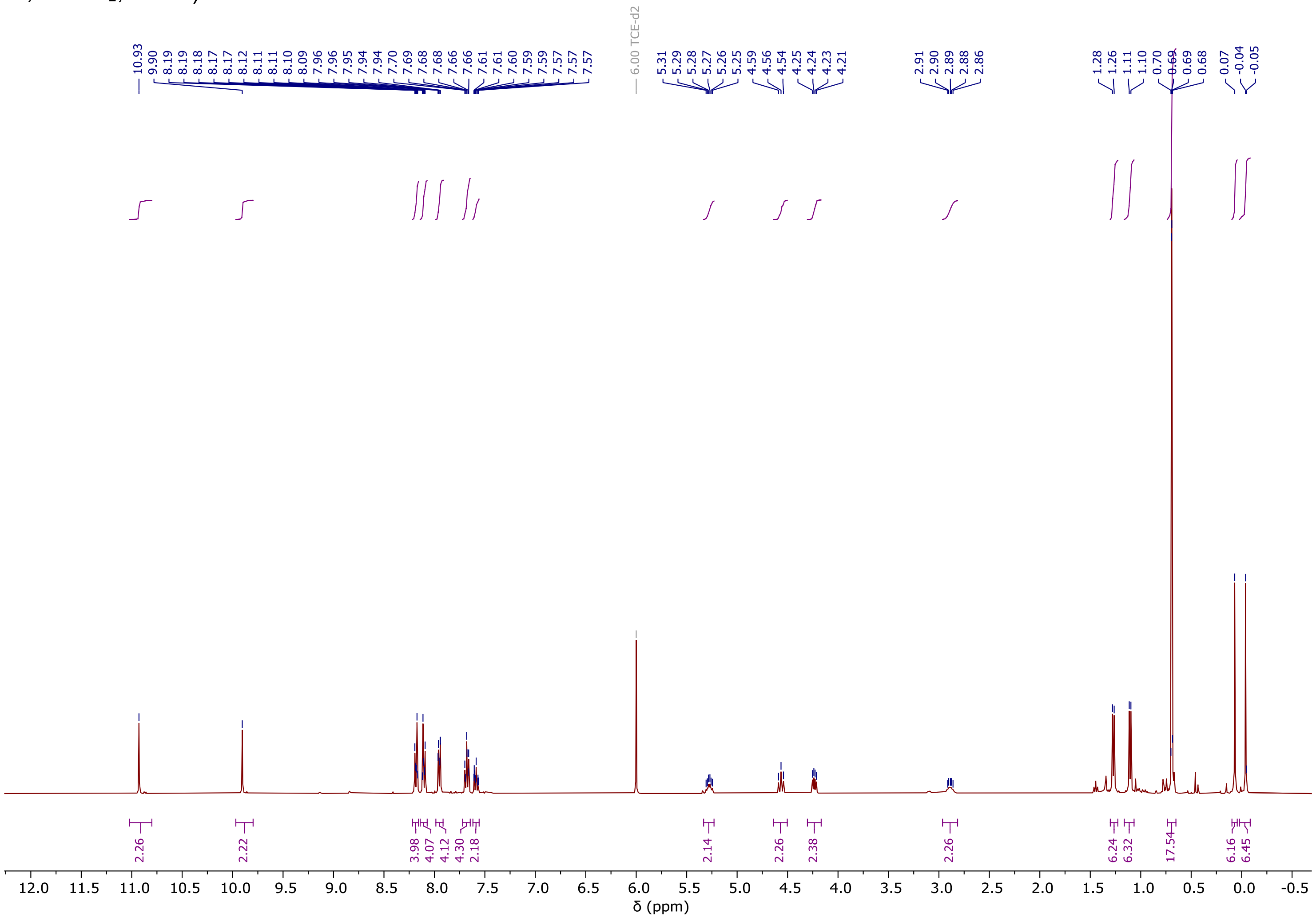

^{13}C NMR (400 MHz, TCE-d_2, 373 K)

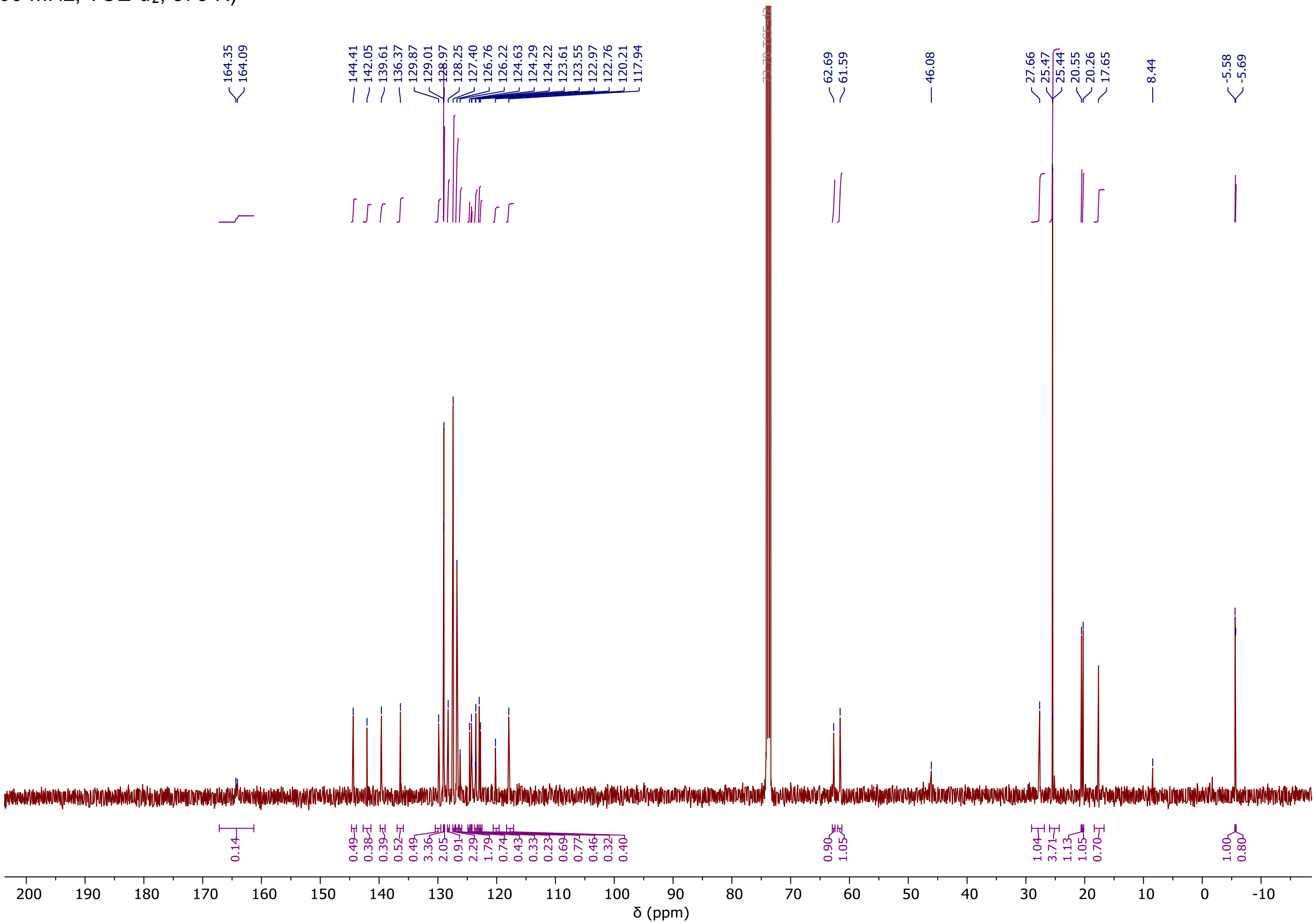

^1H NMR (400 MHz, $CDCl_3$, 298 K)

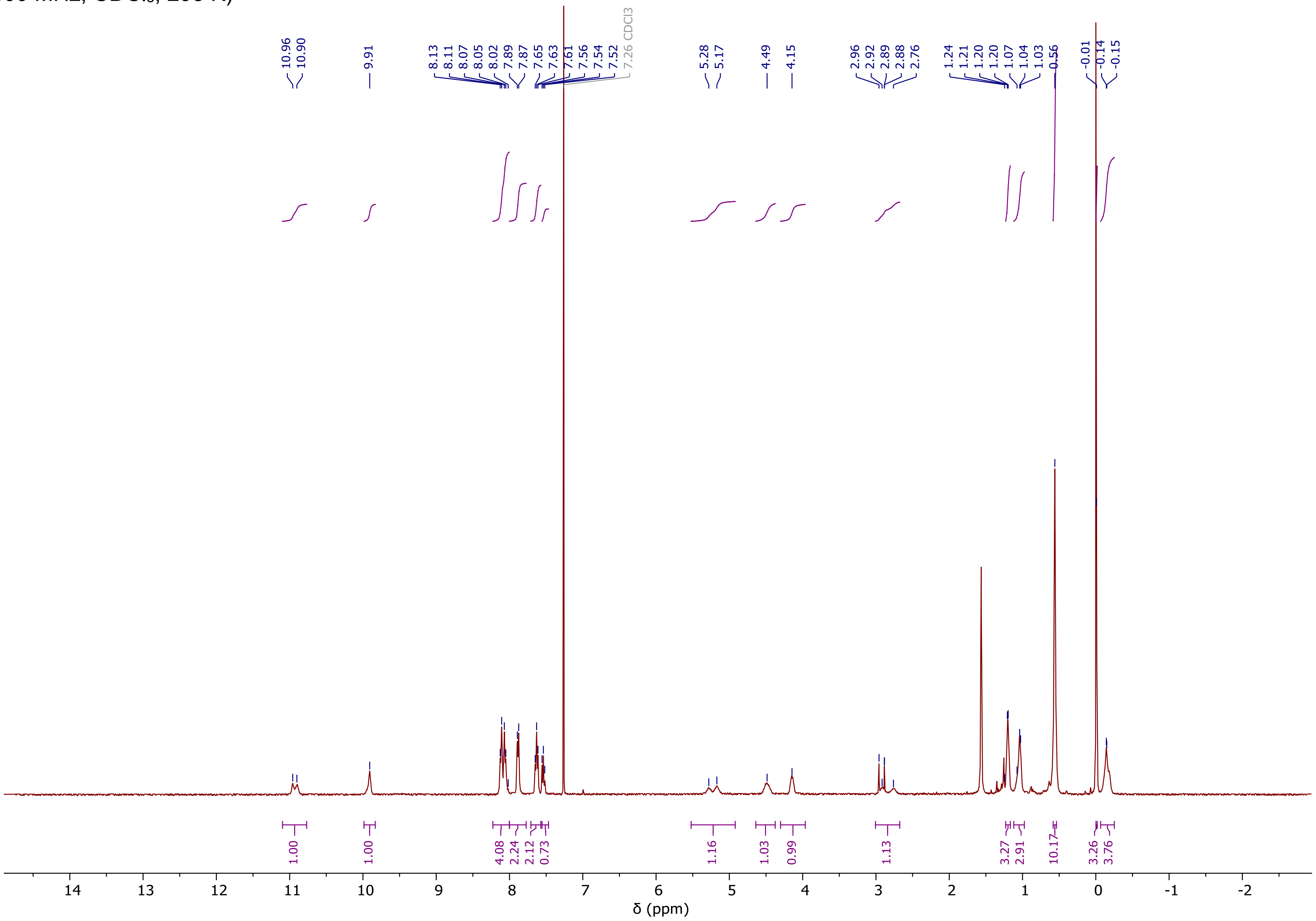

TBSO-DVal-biphenyl triazole-CDI 3b

^{1}H NMR (400 MHz, TCE-d_2, 298 K)

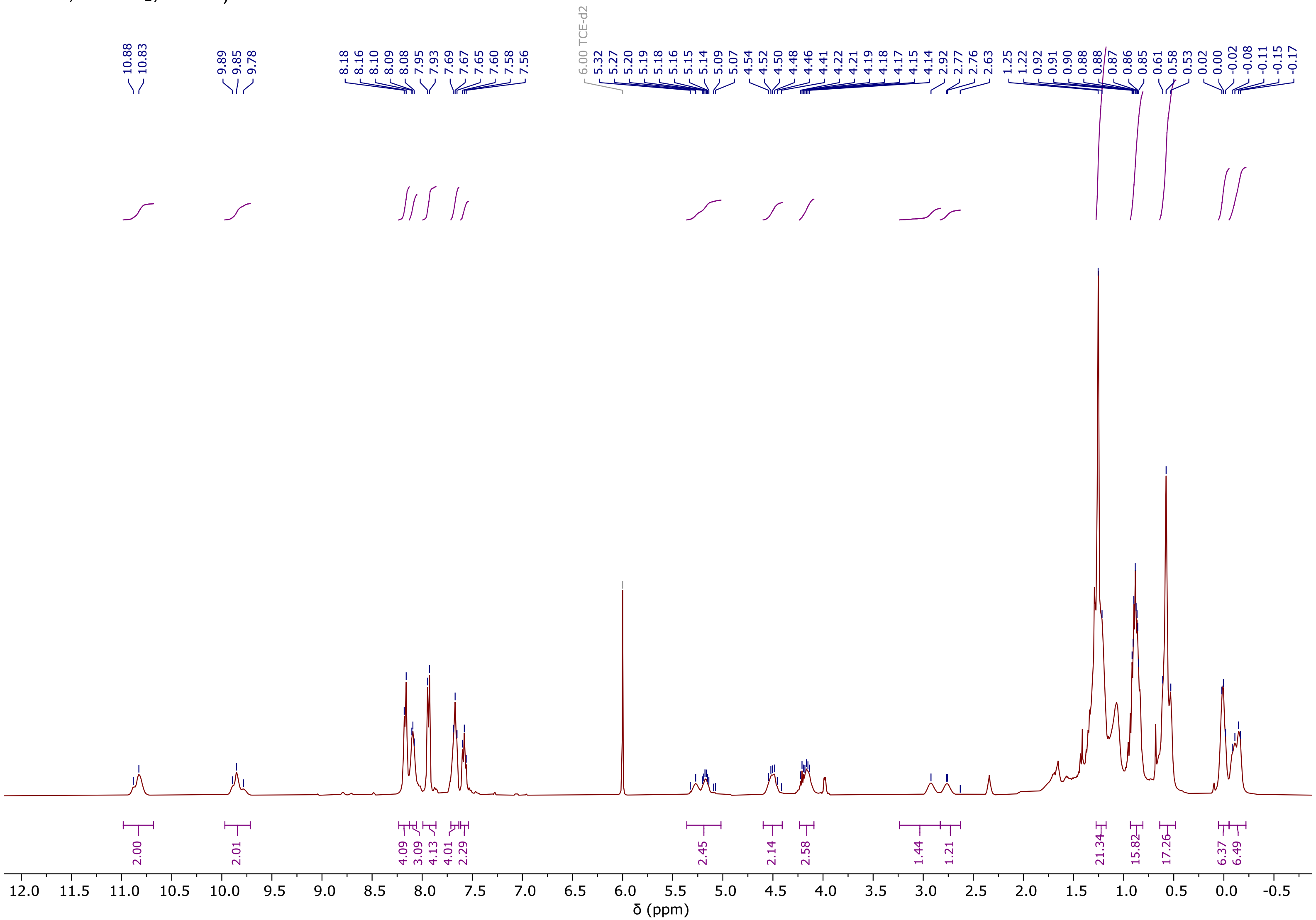

^{13}C NMR (101 MHz, TCE-d_2, 298 K)

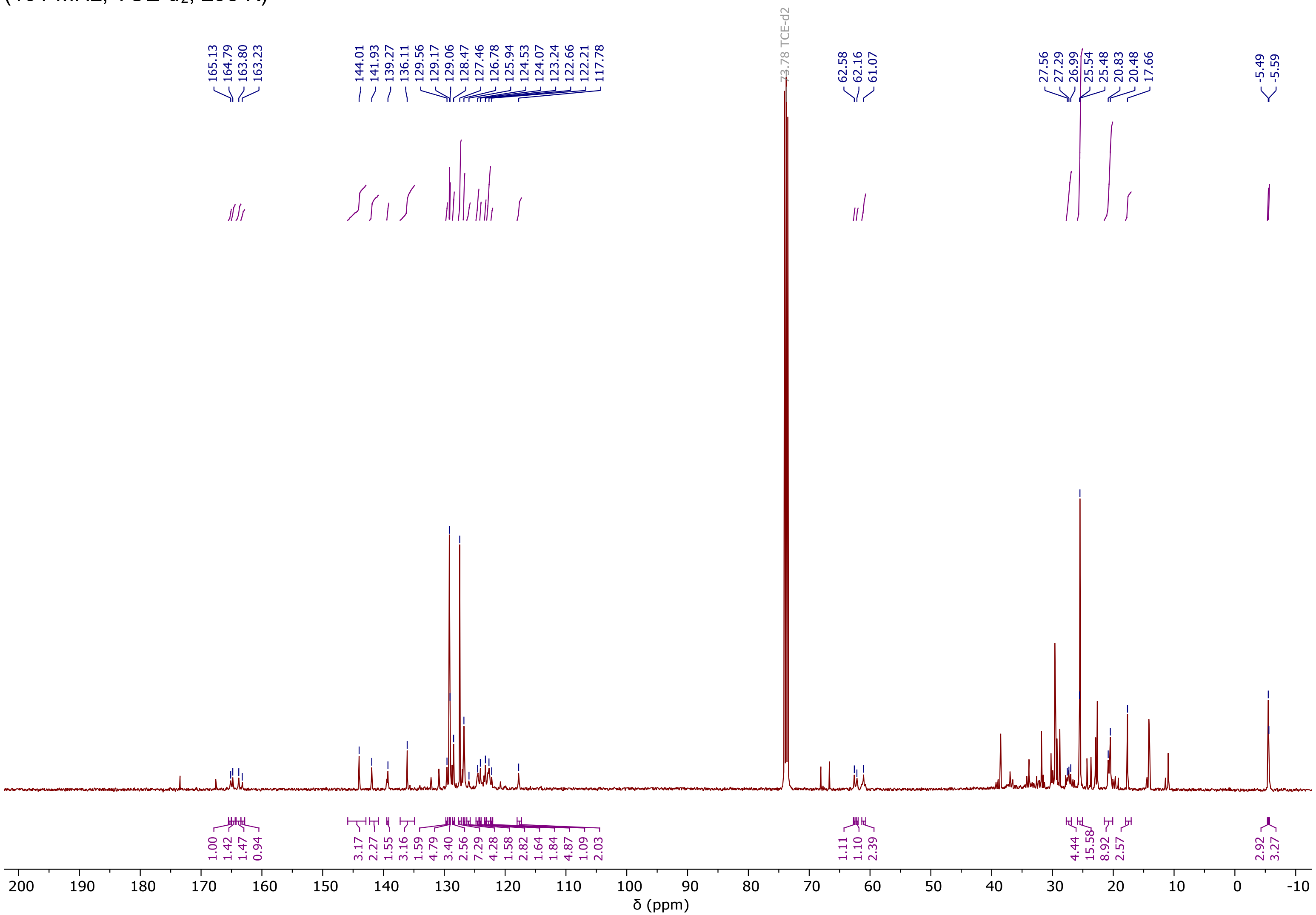

OH-LVal-biphenyl triazole-CDI S4a

^1H NMR (400 MHz, TCE-d_2)

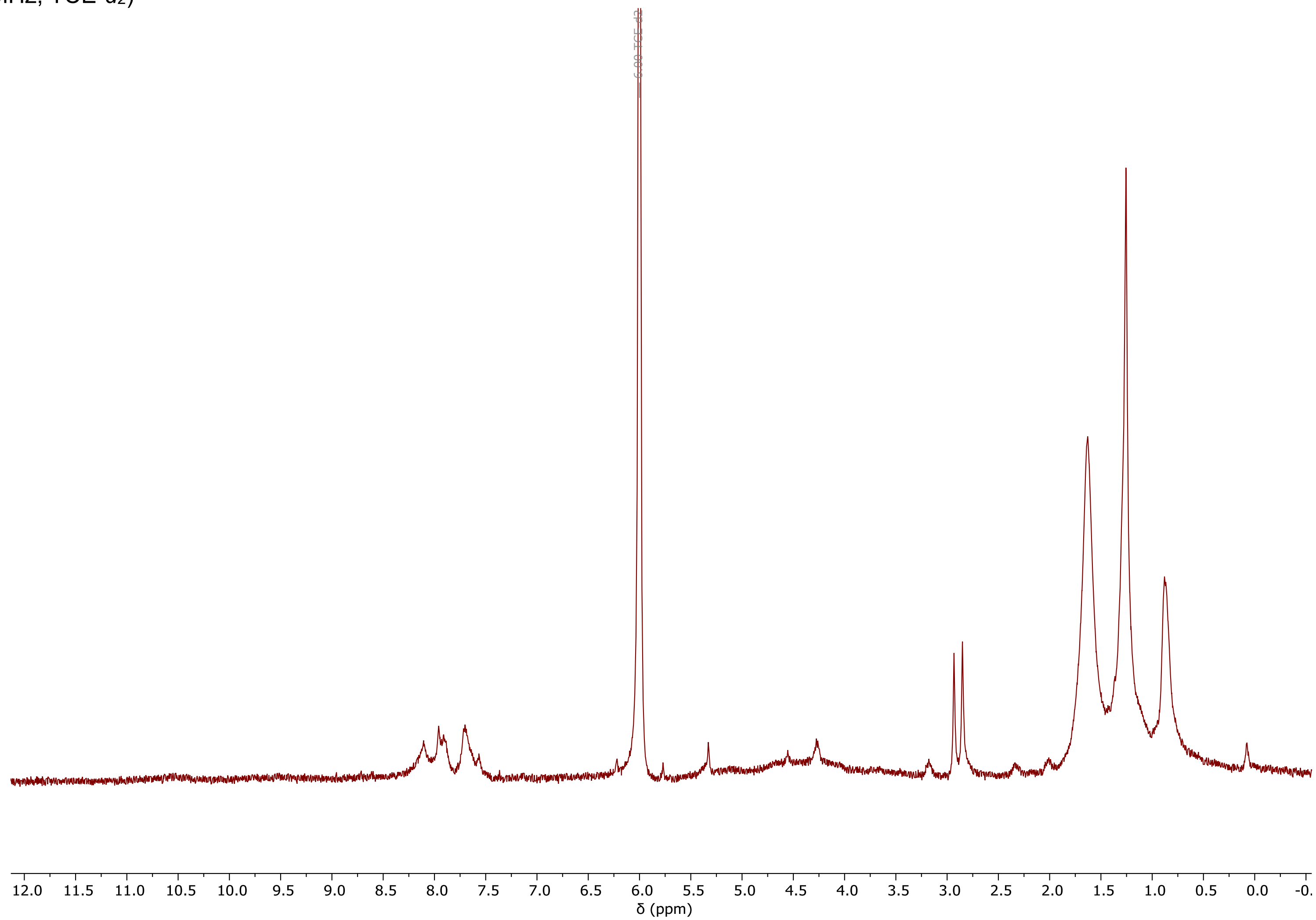

Octanoyl-LVal-CDI 4a

^{1}H NMR (400 MHz, $CDCl_3$)

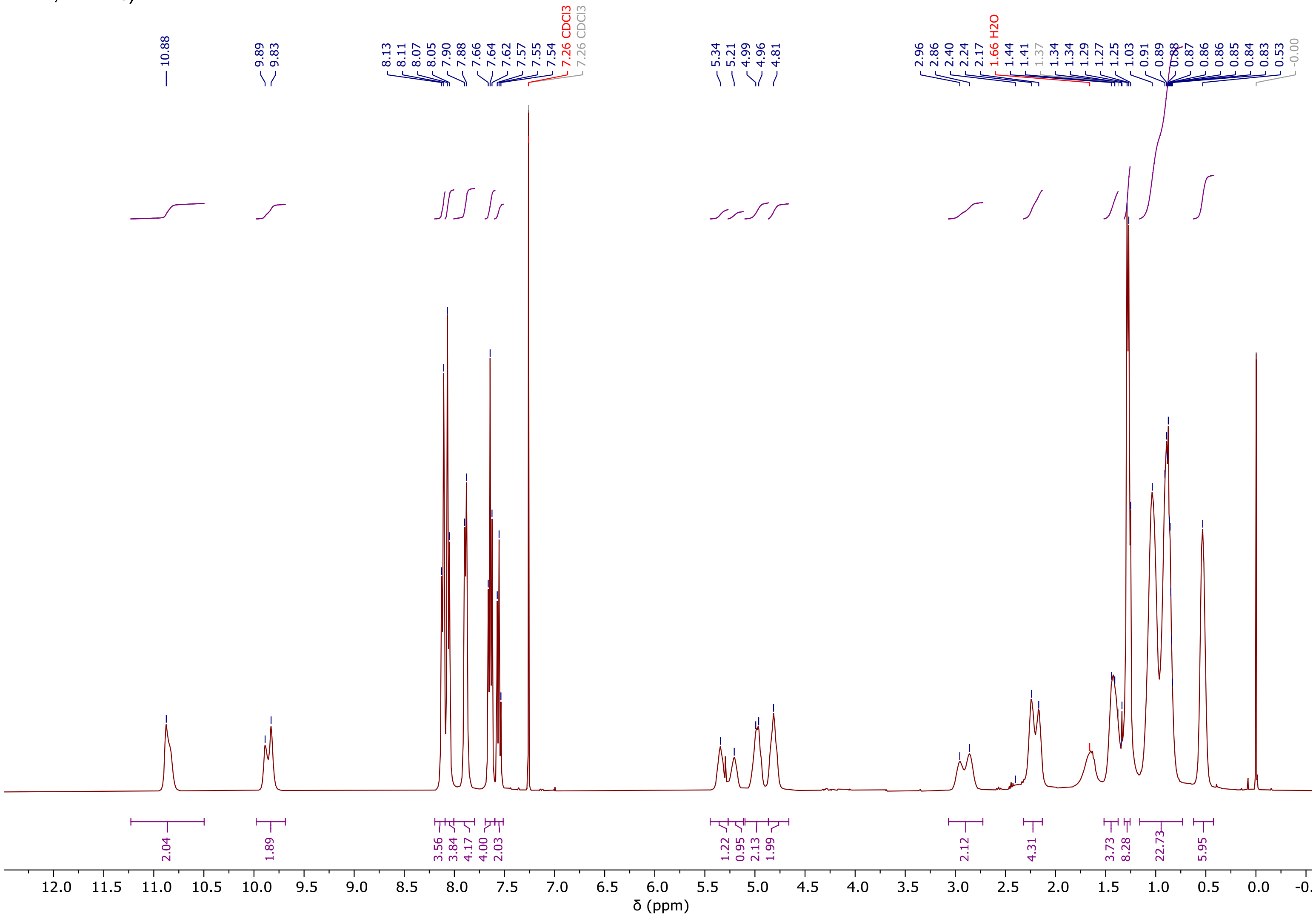

^{13}C NMR (101 MHz, $CDCl_3$)

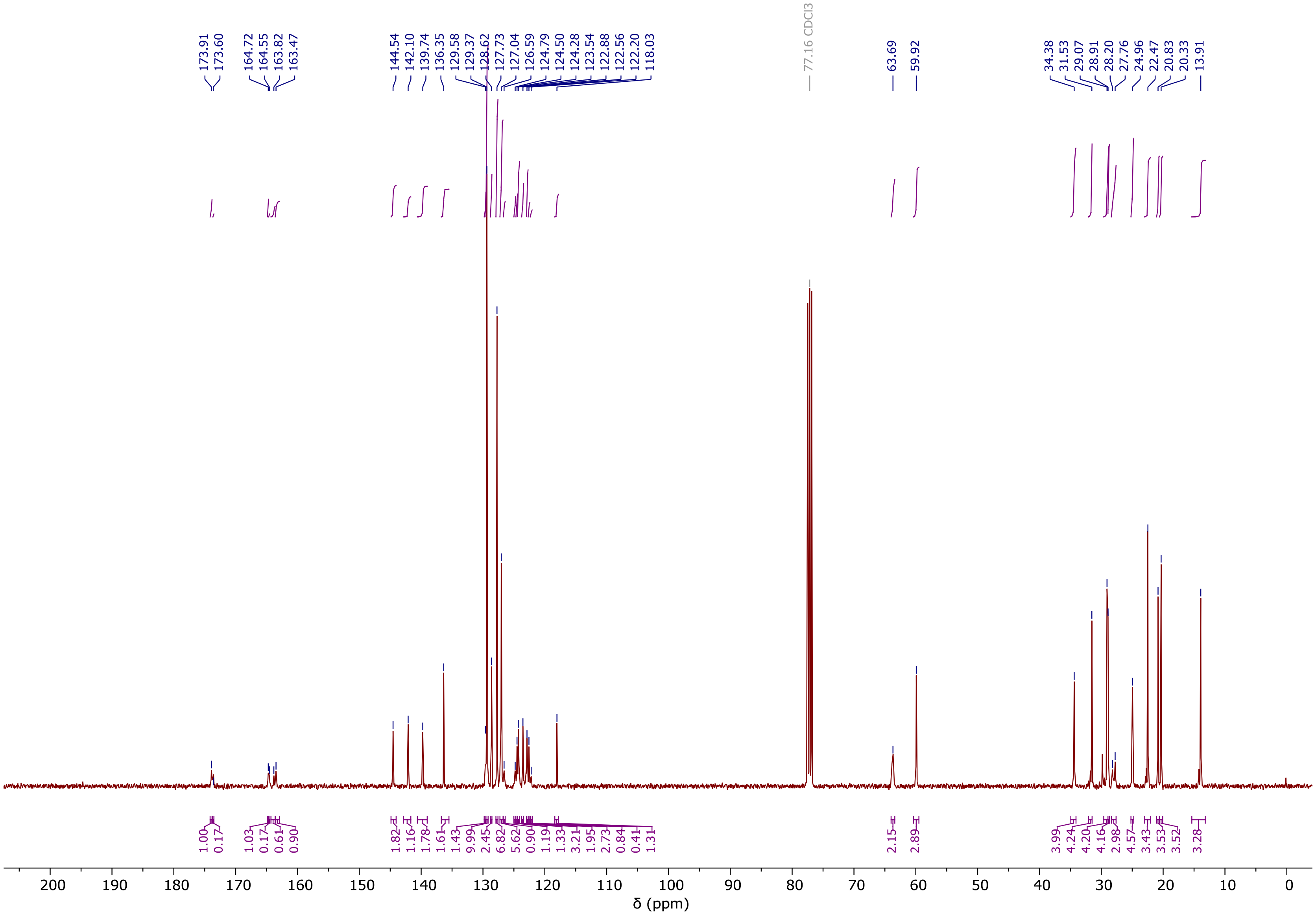
173.91
173.60
164.72
164.55
163.82
163.47
144.54
142.10
139.74
136.35
129.58
129.37
128.62
127.73
127.04
126.59
124.79
124.50
124.28
123.54
122.88
122.56
122.20
118.03
77.16 CDCl3
63.69
59.92
34.38
31.53
29.07
28.91
28.20
27.76
24.96
22.47
20.83
20.33
13.91
1.00
0.17
1.03
0.17
0.61
0.90
1.82
1.16
1.78
1.61
1.43
9.99
2.45
6.82
5.62
0.90
1.19
1.33
3.21
1.95
2.73
0.84
0.41
1.31
2.15
2.89
3.99
4.24
4.20
4.16
2.98
4.57
3.43
3.53
3.52
3.28
200
190
180
170
160
150
140
130
120
110
100
90
80
70
60
50
40
30
20
10
0
δ (ppm)

Octanoyl-DVal-CDI 4b

^{1}H NMR (400 MHz, $CDCl_3$)

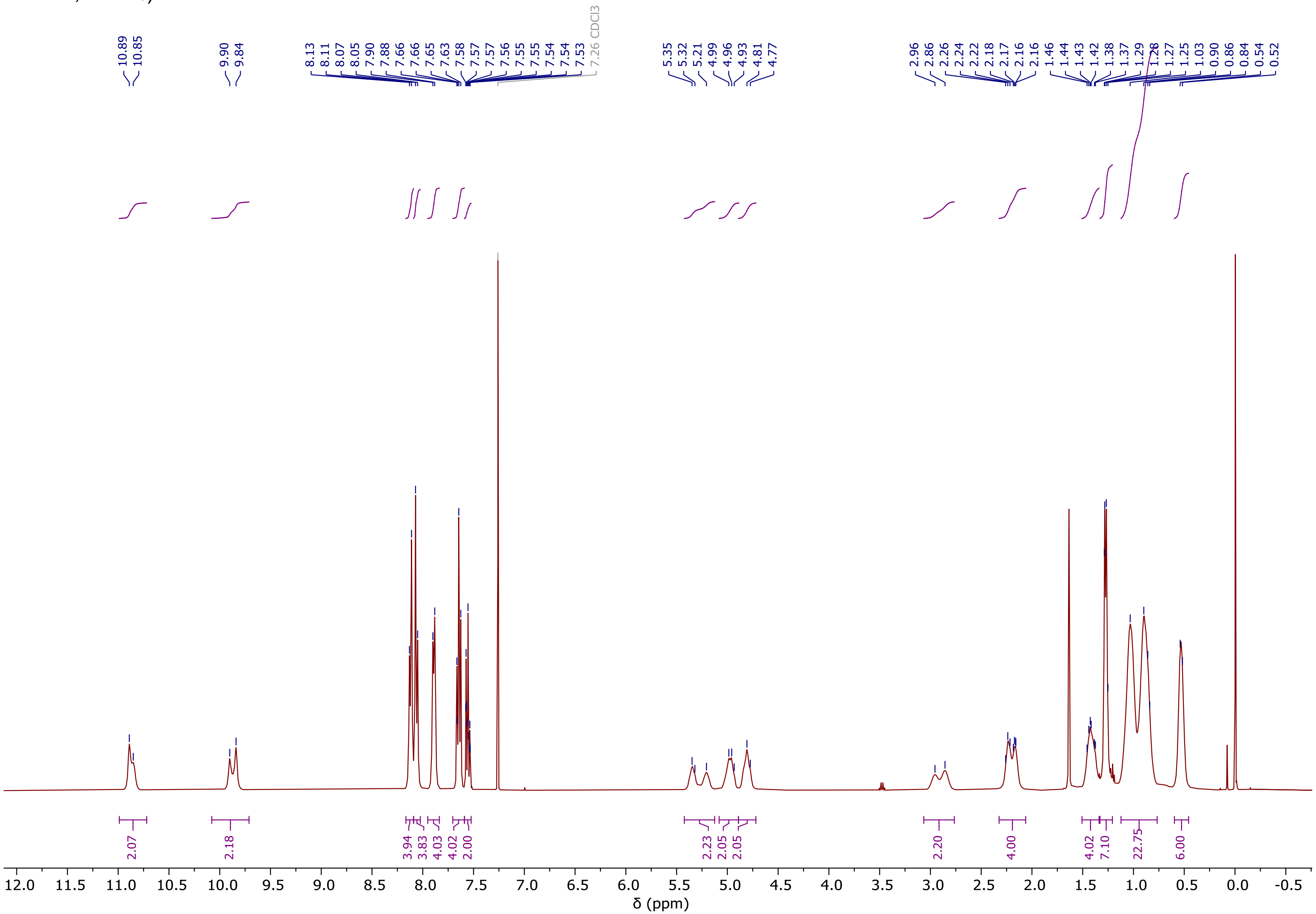

^{13}C NMR (101 MHz, $CDCl_3$)

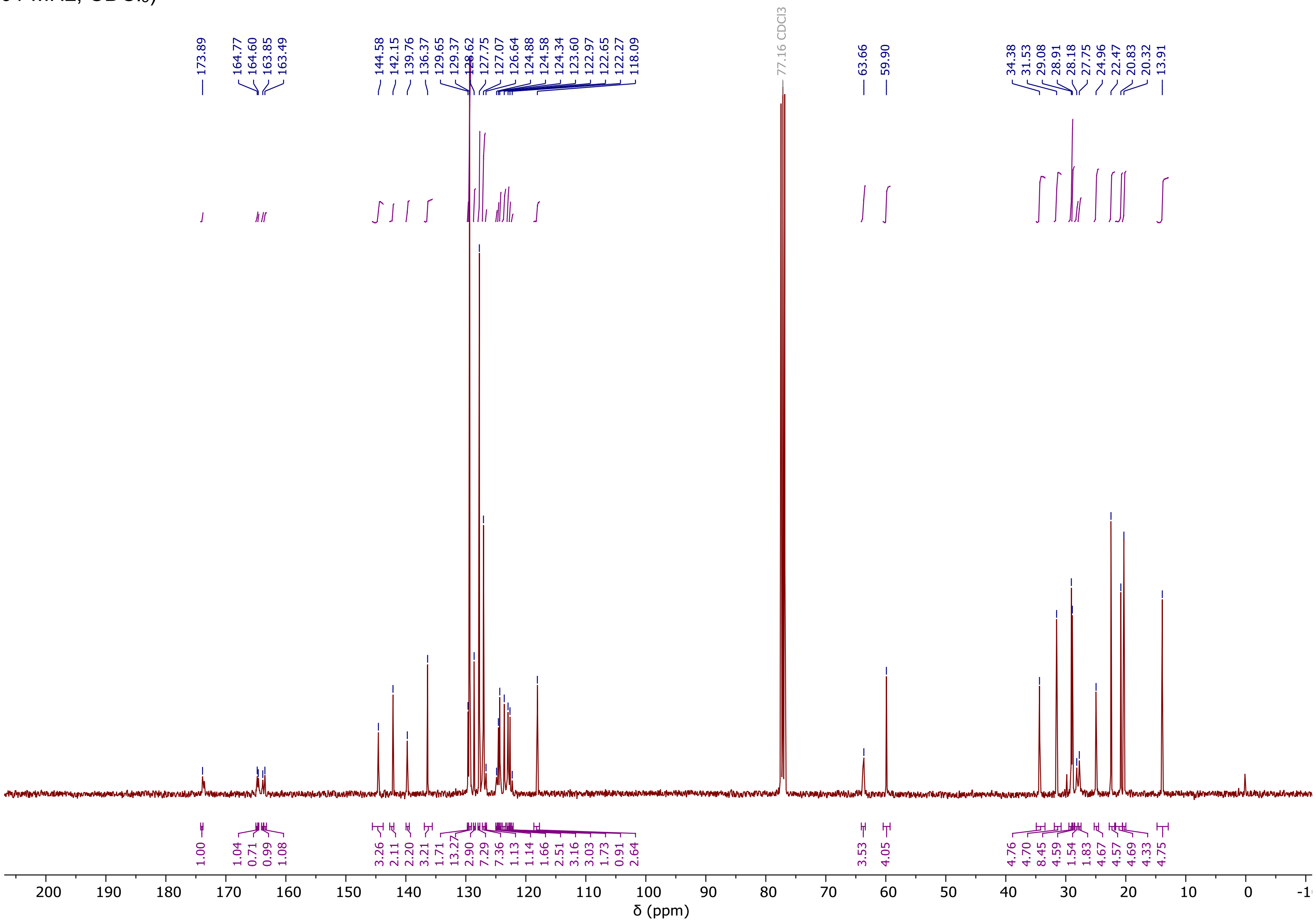

CDI 3a Variable Temperature NMR:

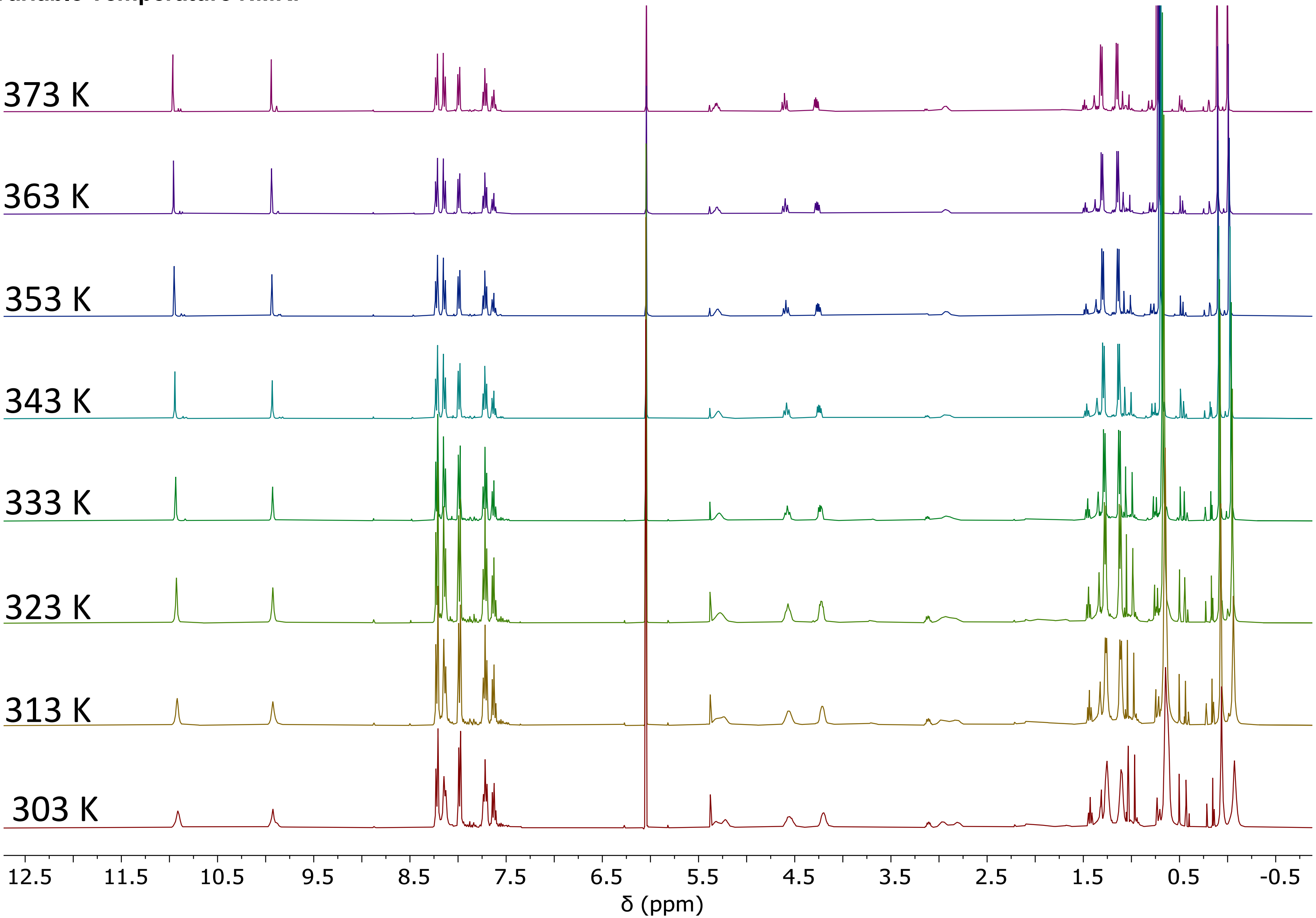

Supplementary Figure 9-1: Variable Temperature NMR of TBS-protected L-Valinol-CDI **3a** in TCE-d_2 from 303 K (red) to 373 K (magenta) in 10 °C increments.

Calculation of $\Delta G^{\ddagger}$ of interconversion from variable temperature NMR data

Barrier heights of interconversion between syn- and anti- imide rotamers were calculated using the Eyring equation as follows:

$$\Delta G^{\ddagger}_{eT_c} = \mathrm{R}T_c\left[\ln\left(\frac{k_B T_c}{h}\right) - \ln(k_{eT_c})\right]$$

With T_c being the temperature of coalescence (in K), and the rate constant of interconversion at the coalescence point k_{eT_c} being defined as

$$k_{eT_c} = \frac{\pi\Delta\nu}{\sqrt{2}}$$

With $\Delta\nu$ being the difference in chemical shifts of the two rotameric protons in Hz.

Supplementary Table 9-1: Interconversion barrier of valinol imide groups of **3a** in TCE-d_2.

Index	Compound	Proton Environment/ppm	T_c / K	$\Delta\nu$ / ppm	$\Delta G^{\ddagger}_{eT_c}$ / kJ mol^{-1}
**1**	**3a**	2.88	333	0.15	68.32
**2**	**3a**	5.27	323	0.10	67.27
**3**	**3a**	9.90	313	0.04	67.49

Which yields an average $\Delta G^{\ddagger}$ for these three proton environments of 67.69 kJ mol^{-1}.

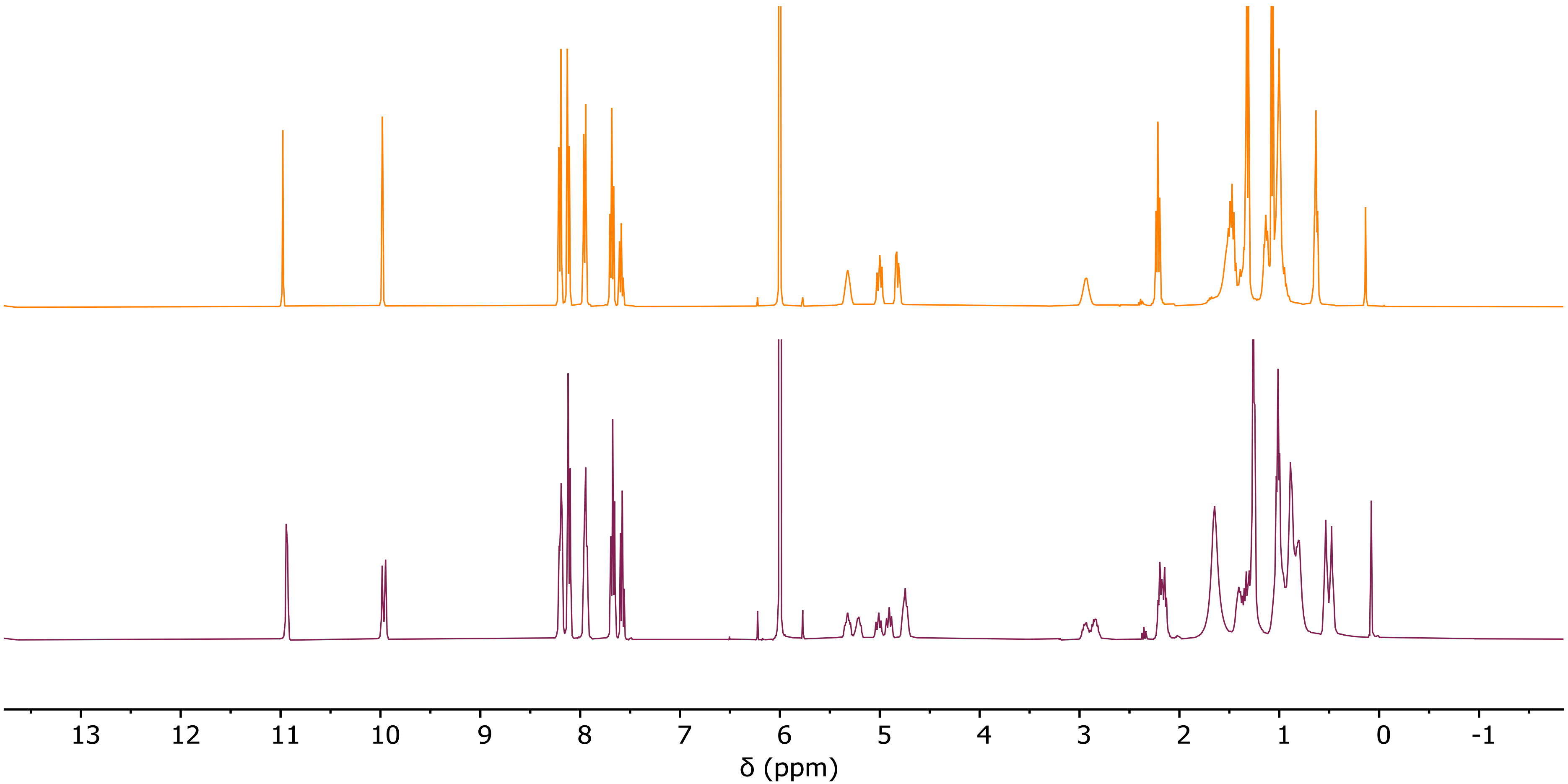

Supplementary Figure 9-2: Full variable temperature ^{1}H NMR of octanoylated L-valinol-CDI **4a** in TCE-d_2 at 298 K (purple) to 373 K (orange) shown in figure 2c.

10)Mass Spectra

OH-D-Valinol-Br2-PDI S1

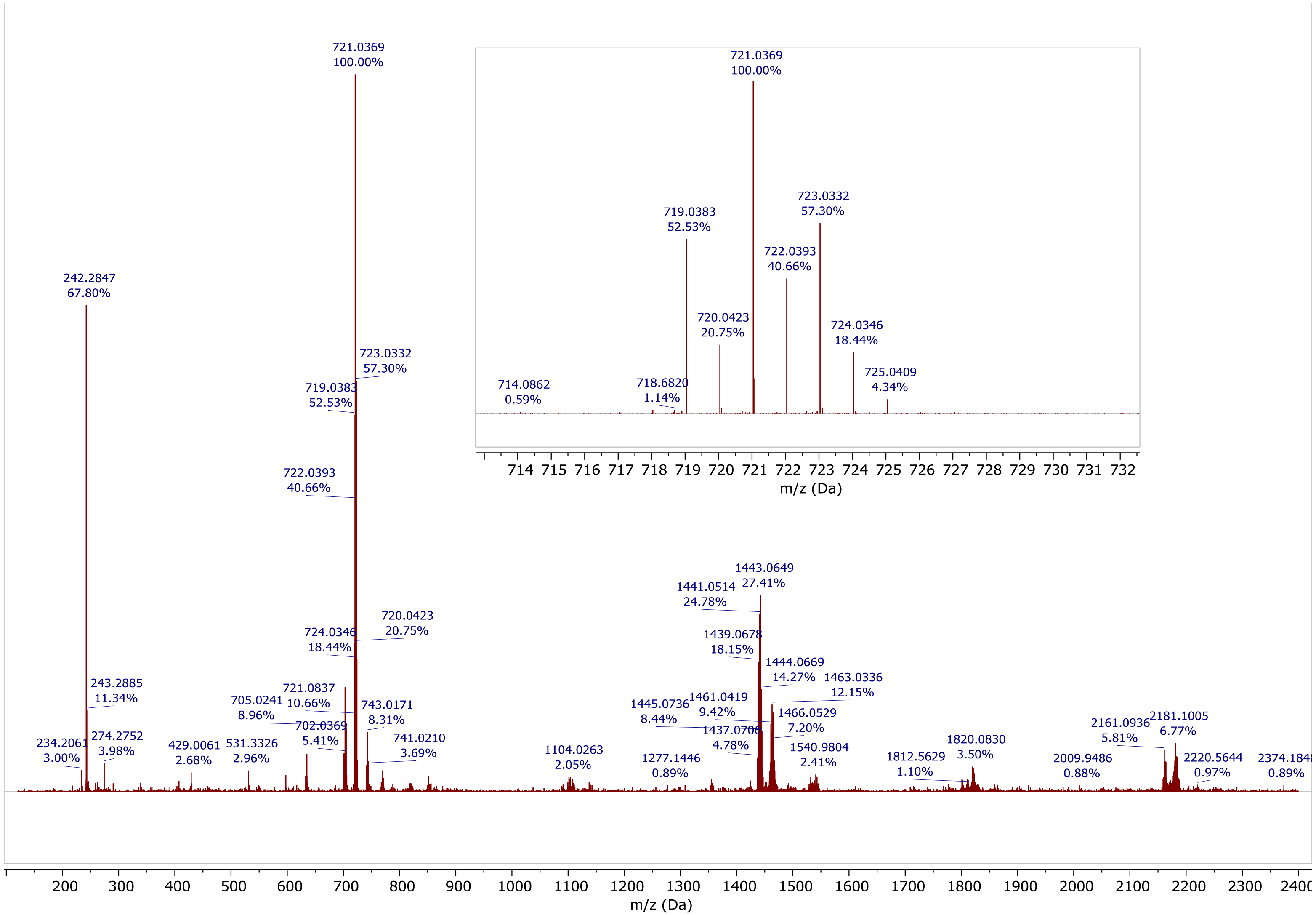

TBSO-D-Valinol-Br2-PDI S2

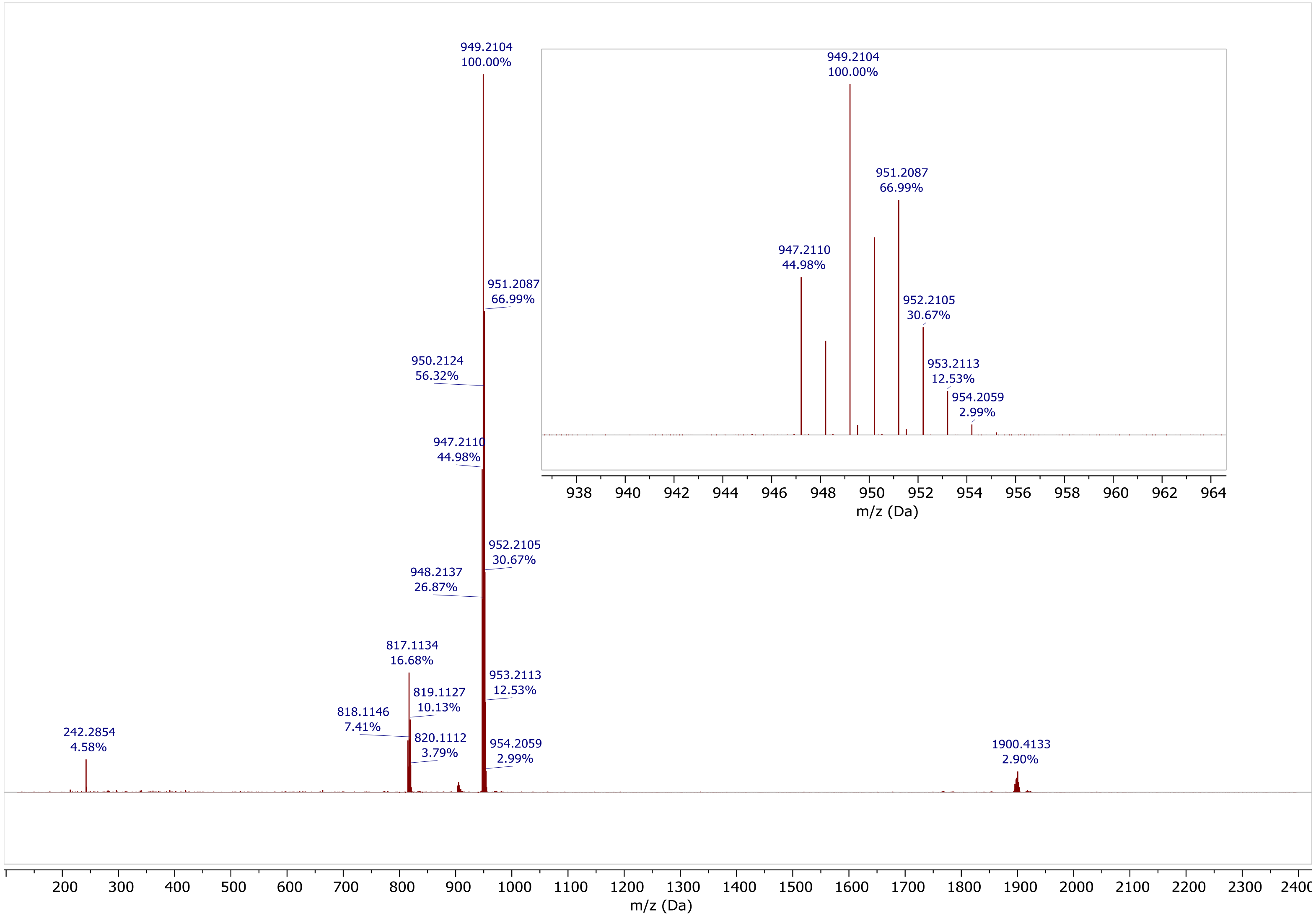

TBSO-L-Valinol-bis-TMS-acetylene-PDI 1a

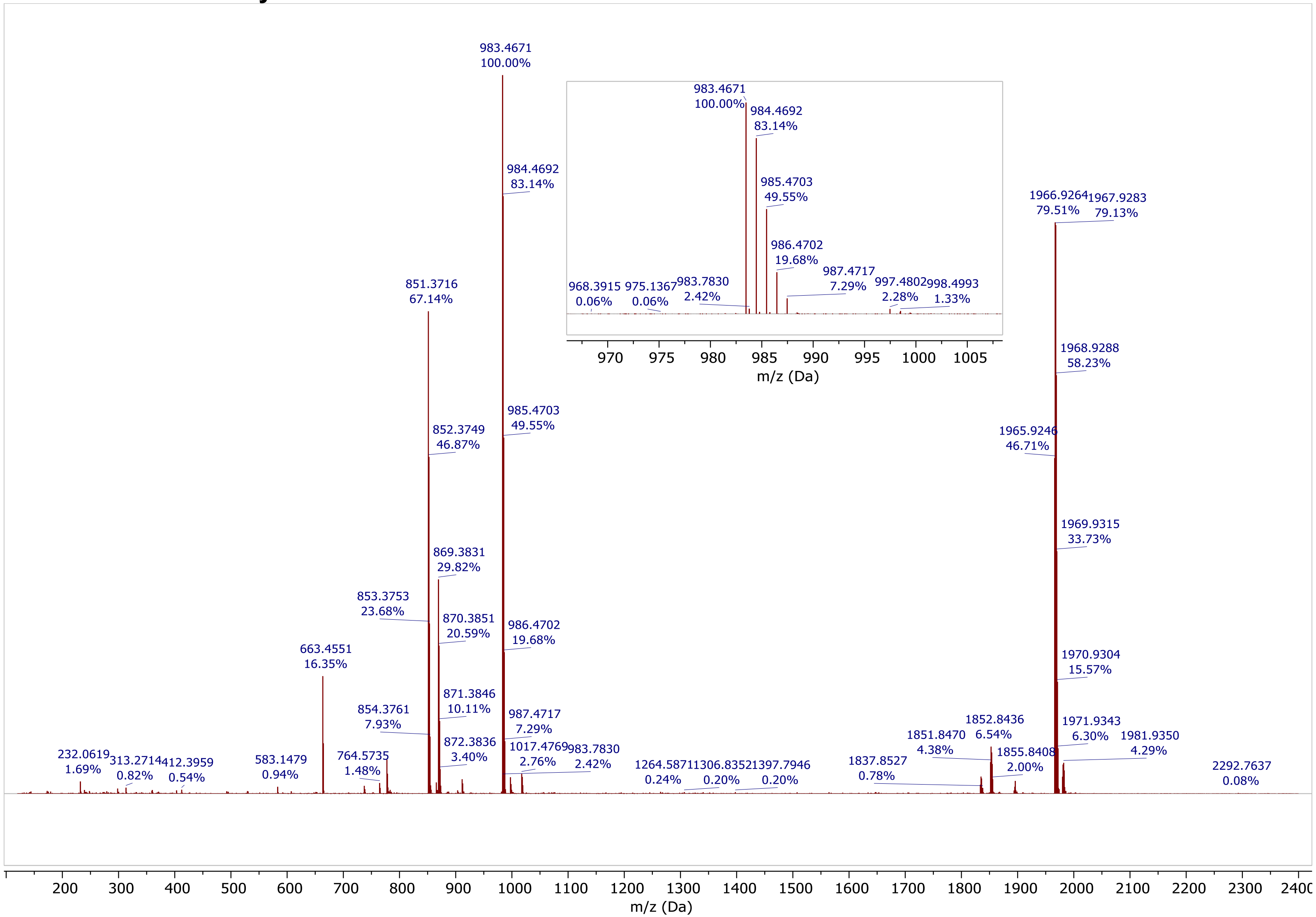

TBSO-D-Valinol-bis-TMS-acetylene-PDI 1b

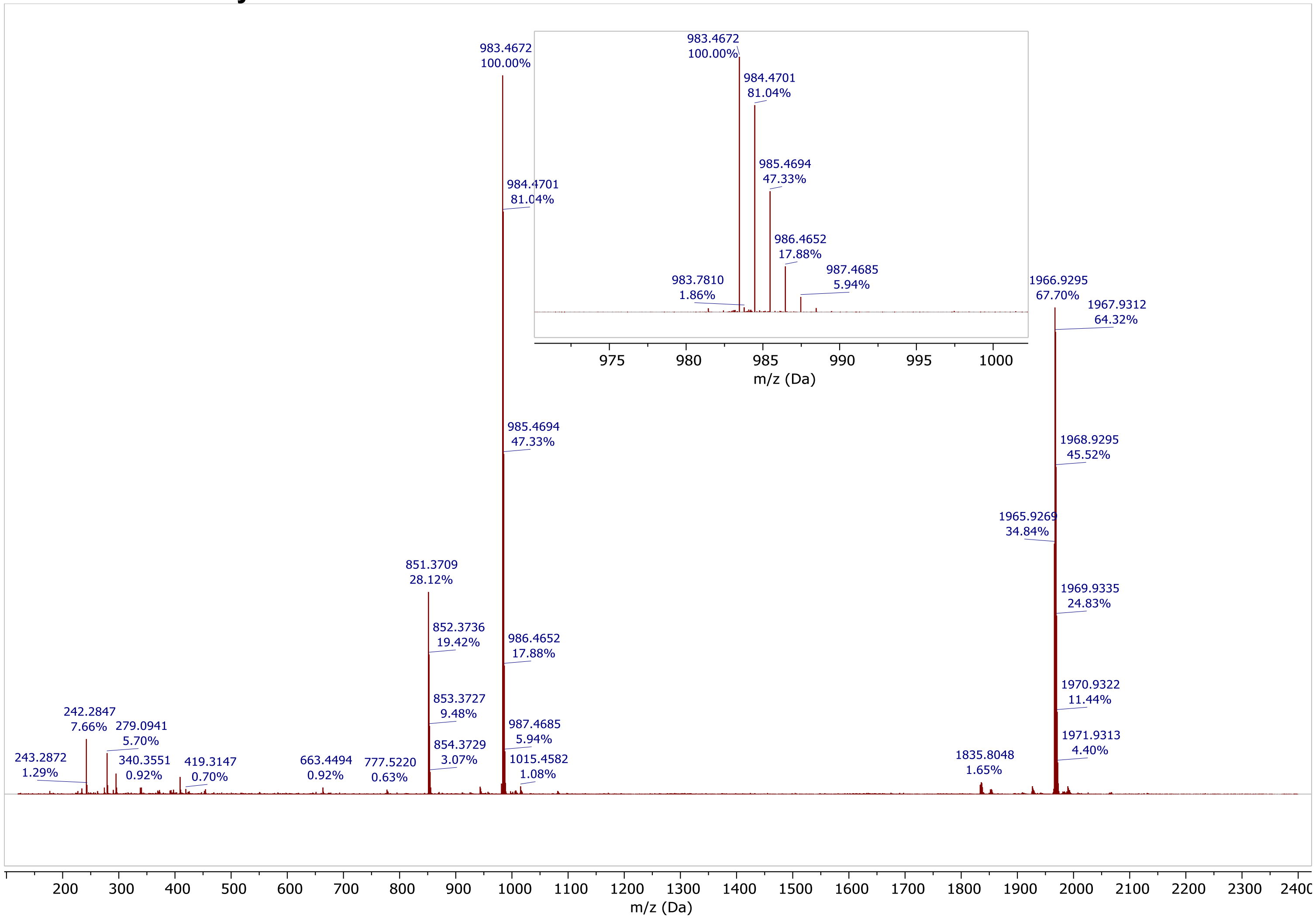

TBSO-LVal-biphenyl triazole-PDI 2a

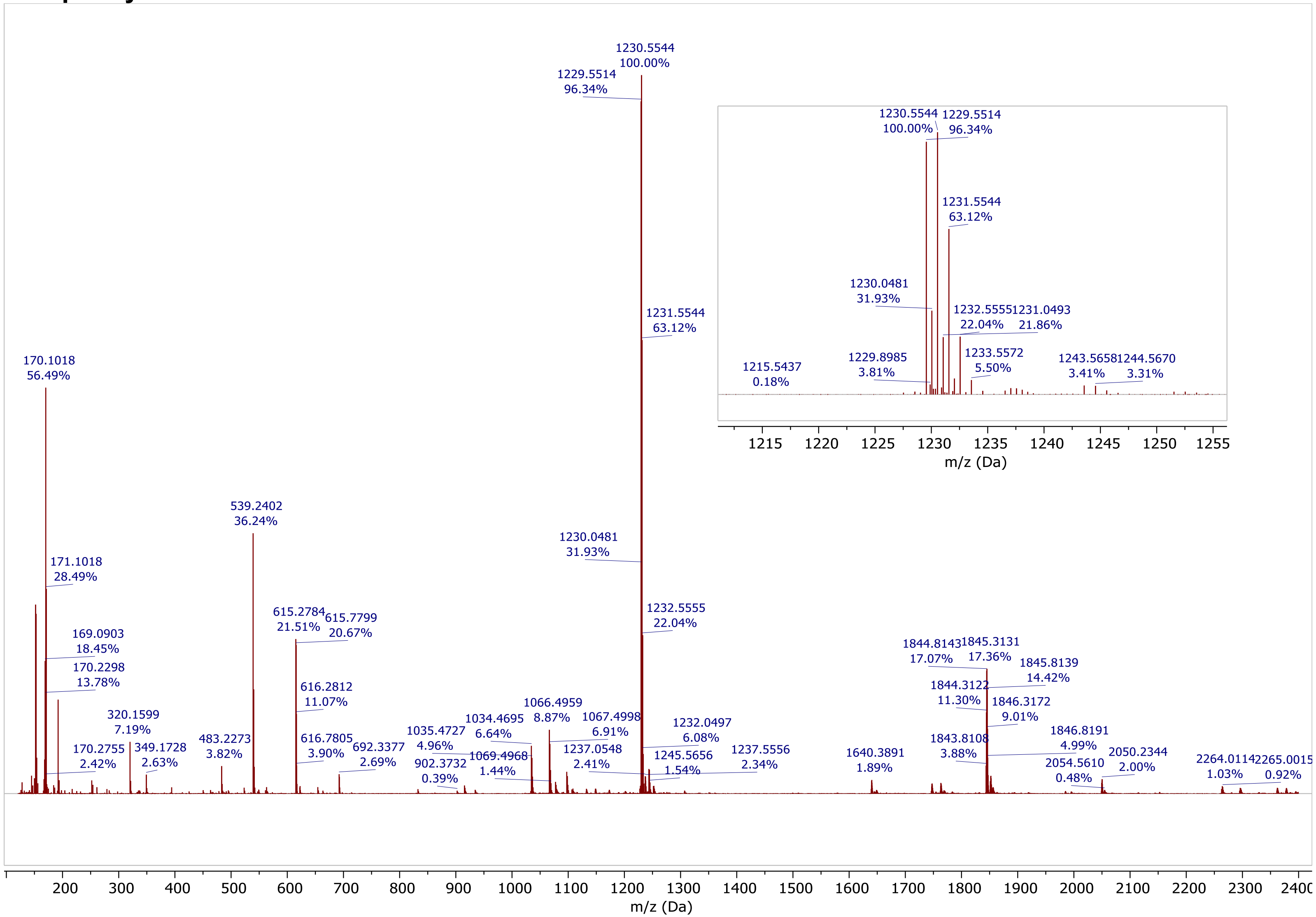

TBSO-DVal-biphenyl triazole-PDI 2b

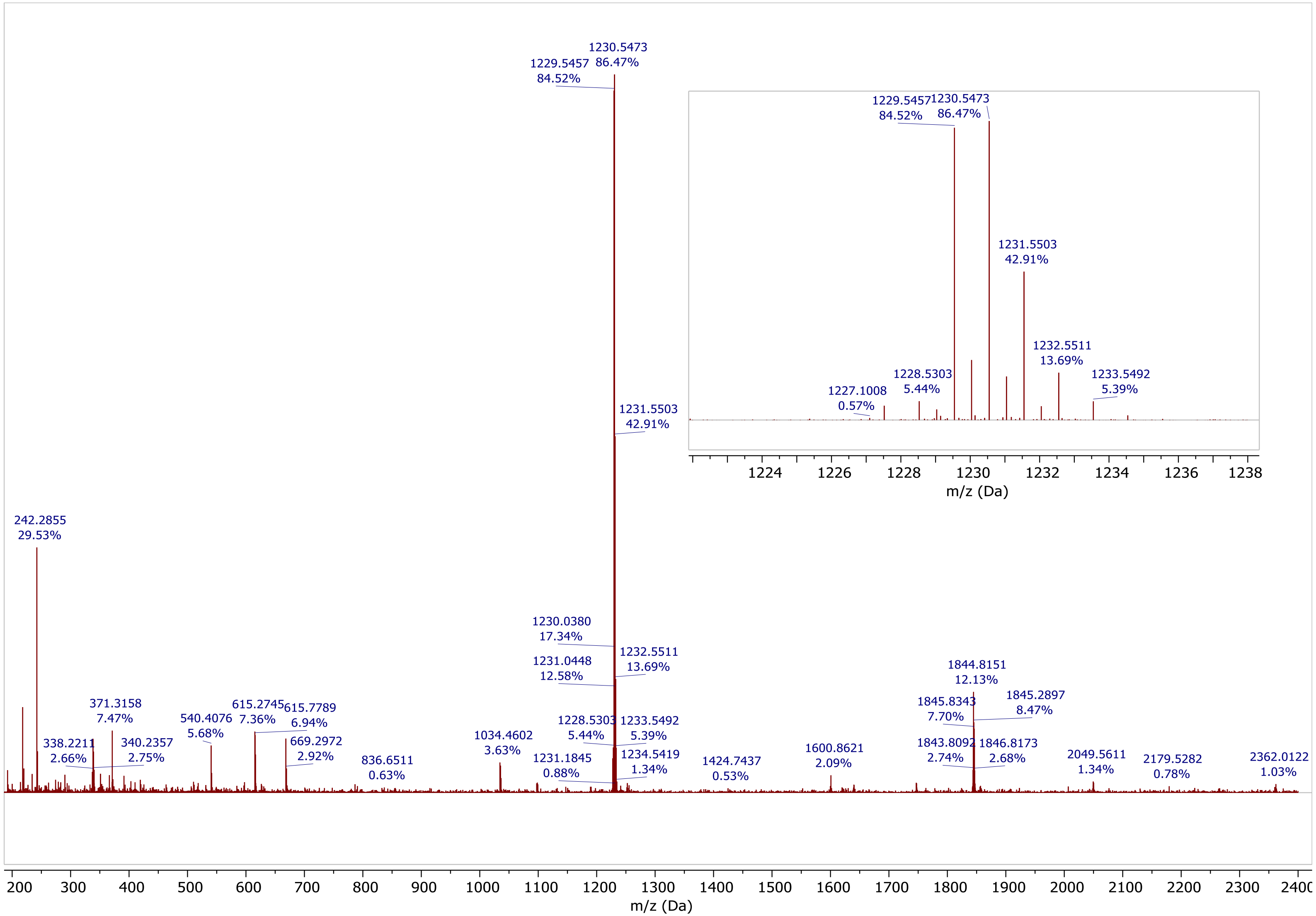

TBSO-LVal-biphenyl triazole-CDI 3a

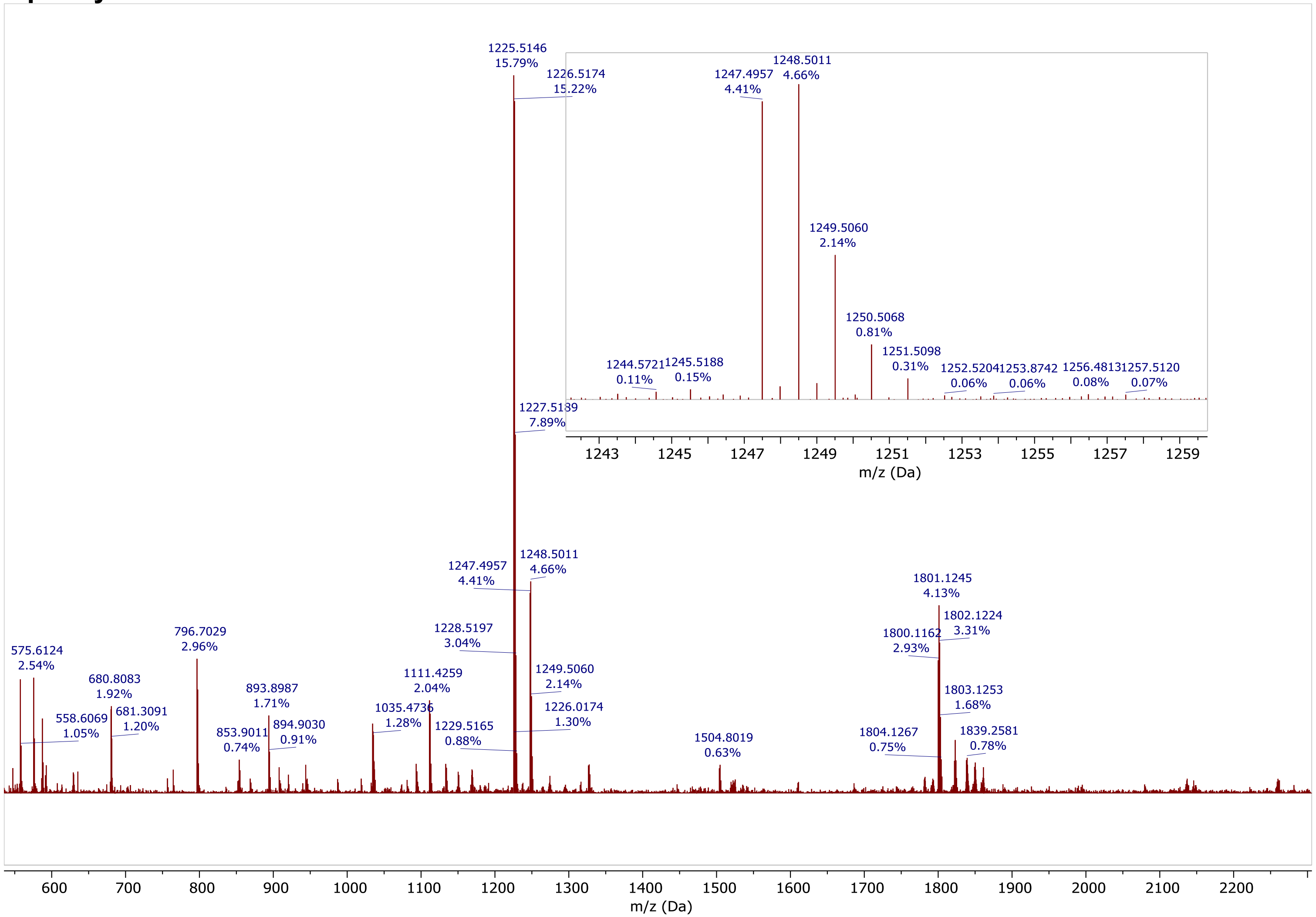

TBSO-DVal-biphenyl triazole-CDI 3b

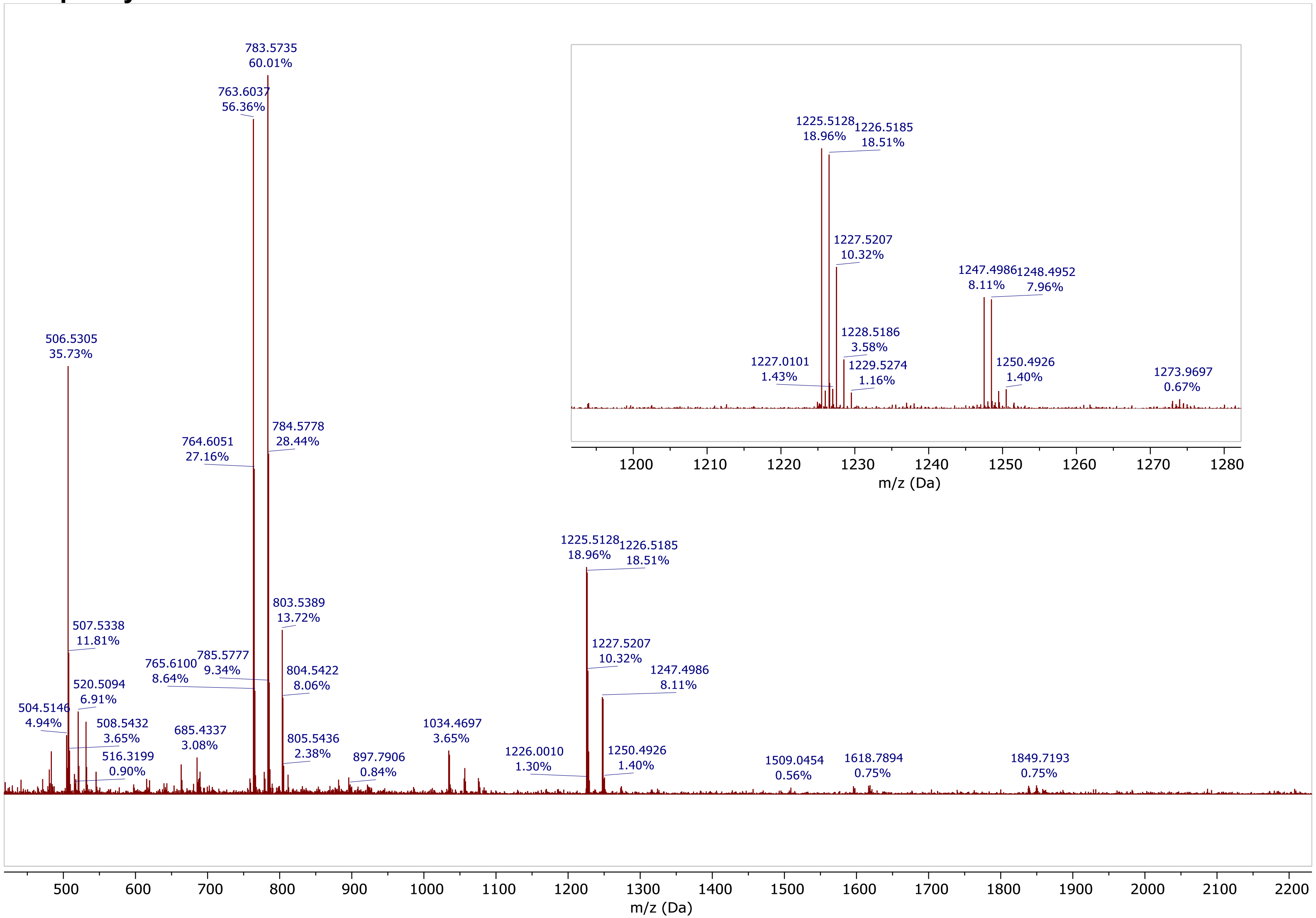

OH-LVal-biphenyl triazole-CDI S4a

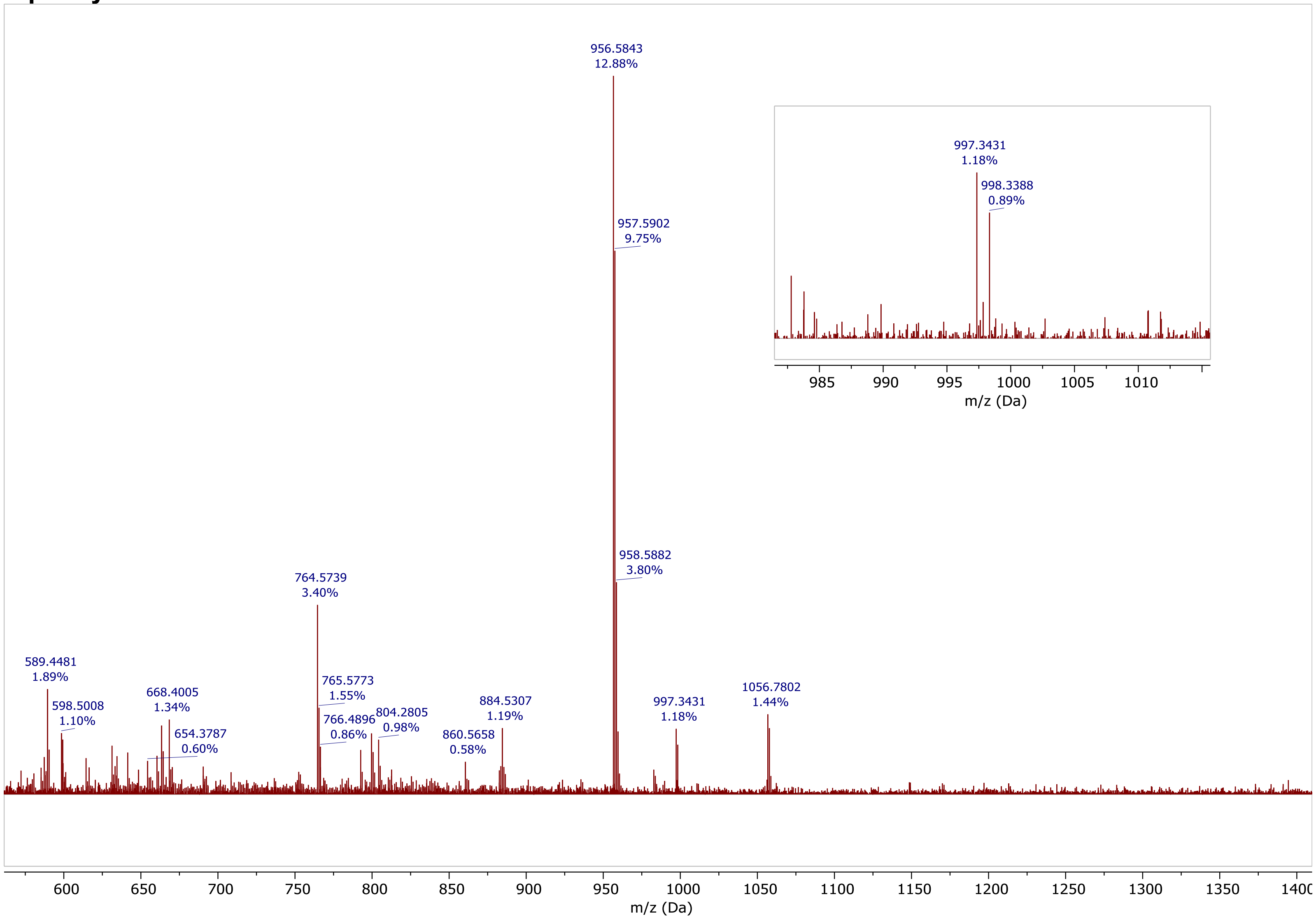

OH-DVal-biphenyl triazole-CDI S4b

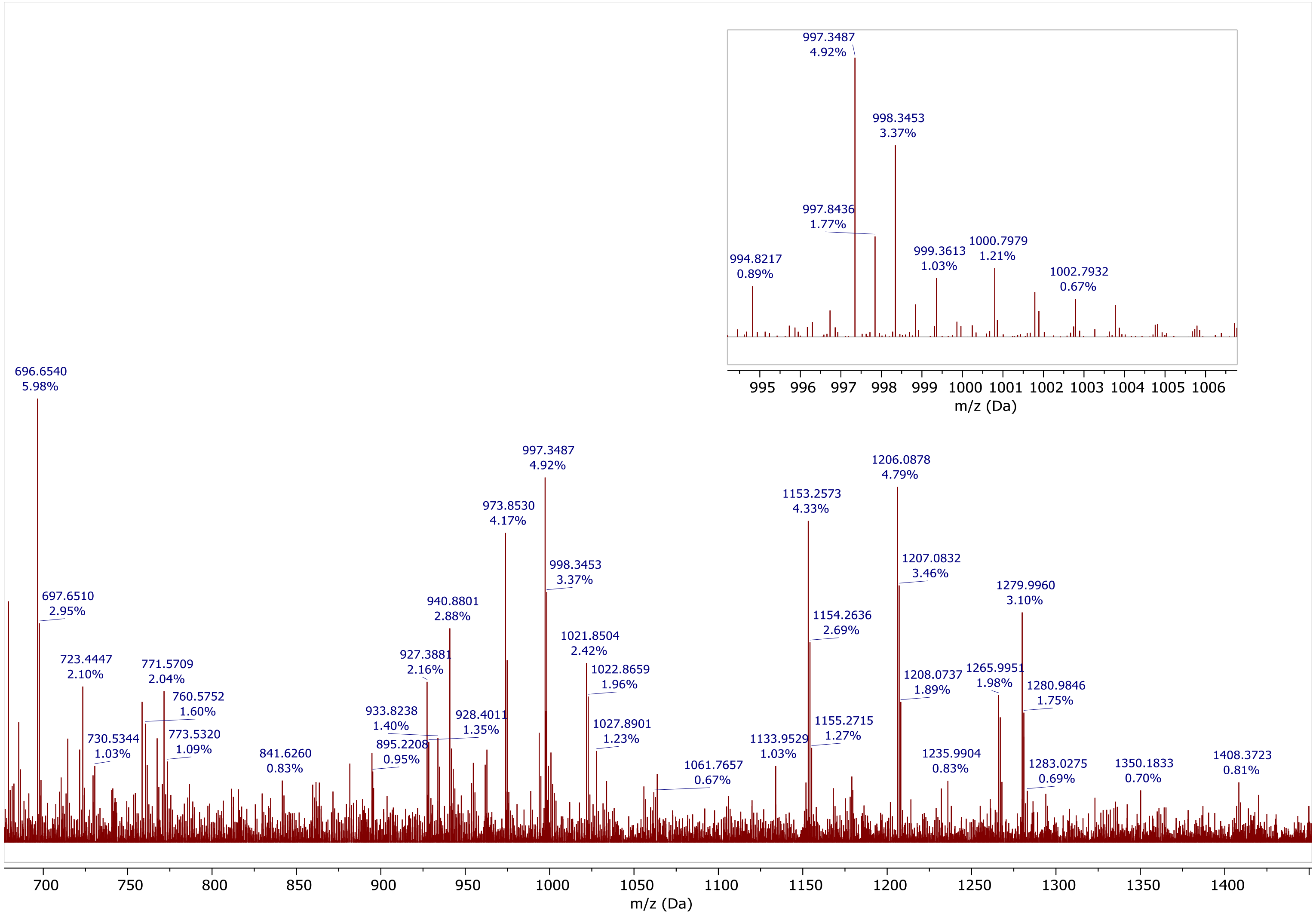

Octanoyl-LVal-biphenyl triazole-CDI 4a

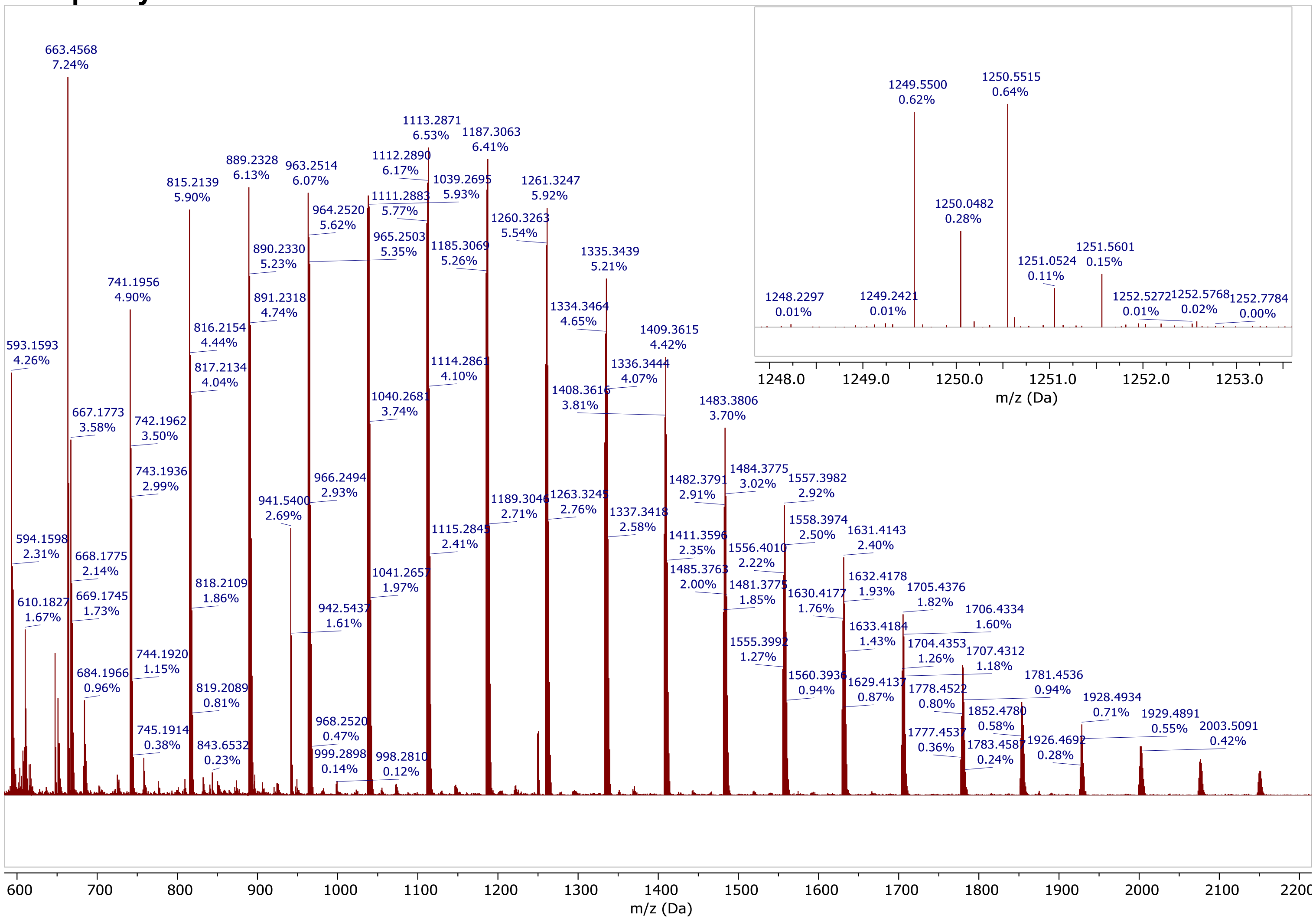

Octanoyl-DVal-biphenyl triazole-CDI 4b

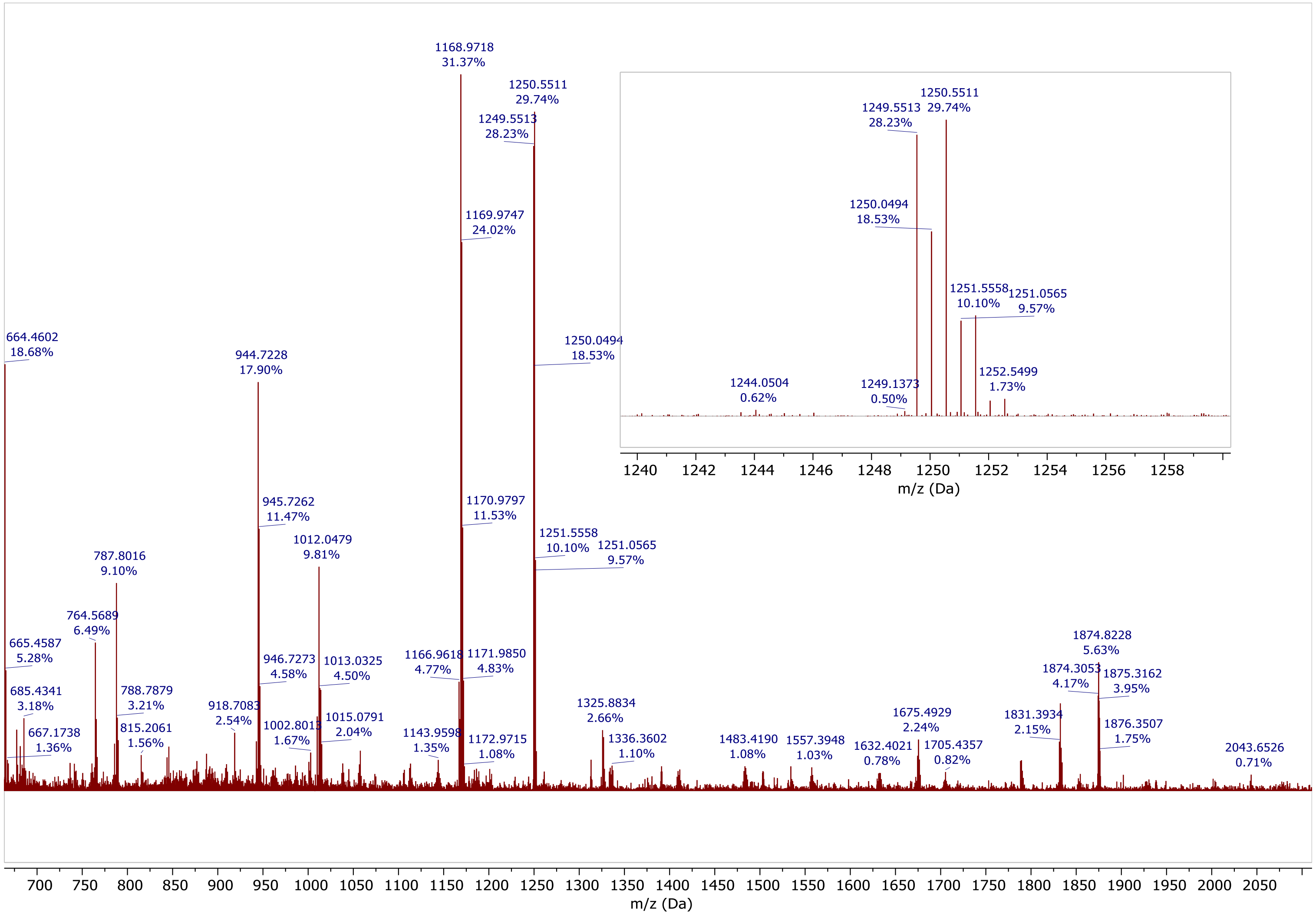

11) References

(1) Hartmann, D.; Penty, S. E.; Pal, R.; Barendt, T. A. Chirally Locked and Dynamic Bis-Perylene Diimide Macrocycles with Multiple Sources of Chirality. *Commun Chem* **2026**. https://doi.org/10.1038/s42004-026-01904-z.

(2) Roemer, M.; Luck, I.; Proschogo, N. Cu(I) Mediated Azidation of Halobenzenes, and Cu Catalysed Selective Azide Reduction to Corresponding Amines. *Advanced Synthesis & Catalysis* **2022**, *364* (17), 2957–2971. https://doi.org/10.1002/adsc.202200594.

(3) Liu, H.; Xu, B. Gold-Catalyzed Azidation, Cyanation, and Alkynylation of Aryl Iodides from Readily Available TMS-N3, TMS-CN, and Alkynyl-TMS. *Chem. Commun.* **2025**, *61* (82), 16006–16009. https://doi.org/10.1039/D5CC02290H.

(4) Carr, R.; Puckrin, R.; McMahon, B. K.; Pal, R.; Parker, D.; Pålsson, L.-O. Induced Circularly Polarized Luminescence Arising from Anion or Protein Binding to Racemic Emissive Lanthanide Complexes. *Methods Appl. Fluoresc.* **2014**, *2* (2), 024007. https://doi.org/10.1088/2050-6120/2/2/024007.

(5) Würthner, F. Solvent Effects in Supramolecular Chemistry: Linear Free Energy Relationships for Common Intermolecular Interactions. *J. Org. Chem.* **2022**, *87* (3), 1602–1615. https://doi.org/10.1021/acs.joc.1c00625.

(6) Smulders, M. M. J.; Nieuwenhuizen, M. M. L.; de Greef, T. F. A.; van der Schoot, P.; Schenning, A. P. H. J.; Meijer, E. W. How to Distinguish Isodesmic from Cooperative Supramolecular Polymerisation. *Chemistry – A European Journal* **2010**, *16* (1), 362–367. https://doi.org/10.1002/chem.200902415.

(7) Ugras, T. J.; Yao, Y.; Robinson, R. D. Can We Still Measure Circular Dichroism with Circular Dichroism Spectrometers: The Dangers of Anisotropic Artifacts. *Chirality* **2023**, *35* (11), 846–855. https://doi.org/10.1002/chir.23597.

(8) Harada, T.; Moriyama, H. Solid-State Circular Dichroism Spectroscopy. In *Encyclopedia of Polymer Science and Technology*; John Wiley & Sons, Ltd, 2013. https://doi.org/10.1002/0471440264.pst587.

(9) Hussain, R.; Jávorfi, T.; Siligardi, G. CD Imaging at High Spatial Resolution at Diamond B23 Beamline: Evolution and Applications. *Front. Chem.* **2021**, *9*. https://doi.org/10.3389/fchem.2021.616928.

(10) Martin, A. T.; Nichols, S. M.; Murphy, V. L.; Kahr, B. Chiroptical Anisotropy of Crystals and Molecules. *Chem. Commun.* **2021**, *57* (66), 8107–8120. https://doi.org/10.1039/D1CC00991E.

(11) Hussain, R.; Krumpa, N.; Strachan, J.; Clarke, D.; Wagner, U.; Macdonald, B.; Reading, D.; Cobb, T.; Gillingham, I.; Price, A.; Smith, M.; Siligardi, G. Design of B23 Circular Dichroism Beamline at Diamond Light Source. *Adv. Syn. Rad.* **2008**, *01* (02), 265–270. https://doi.org/10.1142/S1793617908000173.

(12) Gedeon, C.; Del Rio, N.; Furlan, F.; Taddeucci, A.; Vanthuyne, N.; Gregoriou, V. G.; Fuchter, M. J.; Siligardi, G.; Gasparini, N.; Crassous, J.; Chochos, C. L. Rational Design of New Conjugated Polymers with Main Chain Chirality for Efficient Optoelectronic Devices: Carbo[6]Helicene and Indacenodithiophene Copolymers as Model Compounds. *Advanced Materials* **2024**, *36* (25), 2314337. https://doi.org/10.1002/adma.202314337.

(13) Chowdhury, R.; Preuss, M. D.; Cho, H.-H.; Thompson, J. J. P.; Sen, S.; K. Baikie, T.; Ghosh, P.; Boeije, Y.; Chua, X. W.; Chang, K.-W.; Guo, E.; van der Tol, J.; van den Bersselaar, B. W. L.; Taddeucci, A.; Daub, N.; Dekker, D. M.; Keene, S. T.; Vantomme, G.; Ehrler, B.; Meskers, S. C. J.; Rao, A.; Monserrat, B.; Meijer, E. W.; Friend, R. H. Circularly Polarized Electroluminescence from Chiral Supramolecular Semiconductor Thin Films. *Science* **2025**, *387* (6739), 1175–1181. https://doi.org/10.1126/science.adt3011.

(14) Pancotti, G.; Killalea, C. E.; Rees, T. W.; Liirò-Peluso, L.; Riera-Galindo, S.; Beton, P. H.; Campoy-Quiles, M.; Siligardi, G.; Amabilino, D. B. Film Thickness Dependence of Nanoscale Arrangement of a Chiral Electron Donor in Its Blends with an Achiral Electron Acceptor. *Nanoscale* **2025**, *17* (6), 3133–3144. https://doi.org/10.1039/D4NR04269G.

(15) Wasiluk, M.; Goldmann, C.; Bagiński, M.; Pawlak, M.; Majewski, PawełW.; Abramowicz, J.; Roszkowski, P.; Rebholz, L.; Rockstuhl, C.; Hamon, C.; Lewandowski, W. From Low Symmetry to High Dissymmetry: Chiral Plasmonic Films of Binary and Nanobipyramid Assemblies. *Advanced Functional Materials n/a* (n/a), 2500933. https://doi.org/10.1002/adfm.202500933.

(16) Ugras, T. J.; Carson, R. B.; Lynch, R. P.; Li, H.; Yao, Y.; Cupellini, L.; Page, K. A.; Wang, D.; Arbe, A.; Bals, S.; Smieska, L.; Woll, A. R.; Arteaga, O.; Jávorfi, T.; Siligardi, G.; Pescitelli, G.; Weinstein, S. J.; Robinson, R. D. Transforming Achiral Semiconductors into Chiral Domains with

Exceptional Circular Dichroism. *Science* **2025**, *387* (6733), eado7201. https://doi.org/10.1126/science.ado7201.
(17) Albano, G.; Bertuolo, M.; Zinna, F.; Taddeucci, A.; Jávorfi, T.; Hussain, R.; Farinola, G. M.; Pescitelli, G.; Punzi, A.; Siligardi, G.; Bari, L. D. Unravelling the Origin of Strong Non-Reciprocal Chiroptical Features in Thin Films of a Chiral Diketopyrrolo[3,4-c]Pyrrole Dye. *Nanoscale* **2025**, *17* (9), 5128–5140. https://doi.org/10.1039/D4NR04956J.
(18) Gil, J. J.; and Bernabeu, E. Depolarization and Polarization Indices of an Optical System. *Optica Acta: International Journal of Optics* **1986**, *33* (2), 185–189. https://doi.org/10.1080/713821924.
(19) Azzam, R. M. A. Propagation of Partially Polarized Light through Anisotropic Media with or without Depolarization: A Differential 4 × 4 Matrix Calculus. *J. Opt. Soc. Am., JOSA* **1978**, *68* (12), 1756–1767. https://doi.org/10.1364/JOSA.68.001756.
(20) Arteaga, O.; Canillas, A. Analytic Inversion of the Mueller-Jones Polarization Matrices for Homogeneous Media. *Opt. Lett., OL* **2010**, *35* (4), 559–561. https://doi.org/10.1364/OL.35.000559.
(21) Winter, G.; Waterman, D. G.; Parkhurst, J. M.; Brewster, A. S.; Gildea, R. J.; Gerstel, M.; Fuentes-Montero, L.; Vollmar, M.; Michels-Clark, T.; Young, I. D.; Sauter, N. K.; Evans, G. DIALS: Implementation and Evaluation of a New Integration Package. *Acta Cryst D* **2018**, *74* (2), 85–97. https://doi.org/10.1107/S2059798317017235.
(22) Beilsten-Edmands, J.; Winter, G.; Gildea, R.; Parkhurst, J.; Waterman, D.; Evans, G. Scaling Diffraction Data in the DIALS Software Package: Algorithms and New Approaches for Multi-Crystal Scaling. *Acta Cryst D* **2020**, *76* (4), 385–399. https://doi.org/10.1107/S2059798320003198.
(23) Winter, G. Xia2: An Expert System for Macromolecular Crystallography Data Reduction. *J Appl Cryst* **2010**, *43* (1), 186–190. https://doi.org/10.1107/S0021889809045701.
(24) Dolomanov, O. V.; Bourhis, L. J.; Gildea, R. J.; Howard, J. a. K.; Puschmann, H. OLEX2: A Complete Structure Solution, Refinement and Analysis Program. *J Appl Cryst* **2009**, *42* (2), 339–341. https://doi.org/10.1107/S0021889808042726.
(25) Neese, F. Software Update: The ORCA Program System—Version 5.0. *WIREs Computational Molecular Science* **2022**, *12* (5), e1606. https://doi.org/10.1002/wcms.1606.
(26) Bursch, M.; Mewes, J.-M.; Hansen, A.; Grimme, S. Best-Practice DFT Protocols for Basic Molecular Computational Chemistry. *Angewandte Chemie International Edition* **2022**, *61* (42), e202205735. https://doi.org/10.1002/anie.202205735.
